# Optical coherence tomography for early detection of crop infection

**DOI:** 10.1186/s13007-025-01411-7

**Published:** 2025-07-06

**Authors:** Ghada Salem Sasi, Stephen J. Matcher, Adrien Alexis Paul Chauvet

**Affiliations:** 1 School of Mathematic and Physical Sciences, University of Sheffield, Sheffield, S3 7HF UK; 2 School of Electrical and Electronic Engineering, The University of Sheffield, 3 Solly Street, Sheffield, S1 4DE UK

**Keywords:** Optical coherence tomography, Wheat, *Septoria*, Machine learning

## Abstract

**Background:**

Fungal diseases are among the most significant threats to global crop production, often leading to substantial yield losses. Early detection of crop infection by fungus is the very first step to deploying a timely and effective treatment. Early and reliable detection is thus key to improving yields, sustainability, and achieving food security. Conventional diagnostic methods are however often destructive, slow, or requiring visible symptoms which appear late in the infection process. To overcome these challenges, we propose using optical coherence tomography (OCT) as an innovative imaging tool to provide cross-sectional and three-dimensional images of the plant internal microstructure non-invasively, in vivo, and in real-time.

**Results:**

We demonstrate the use of low-cost OCT to monitoring wheat (cultivar AxC 169) when infected by *Septoria tritici*. We show that OCT analysis can effectively detect signs of infection before any external symptoms appear. Although OCT cannot directly visualize fungal hyphae, OCT reveals apparent morphological changes of the mesophyll where the fungal filaments are expected to develop. This study thus focuses on monitoring and correlating changes within the mesophyll structural organisation with the state of infection. It results in distinct statistical difference between intact and infected wheat plants two days only after infection. We then demonstrate the use of machine learning (ML) for high throughput segmentation of OCT scans, providing a foundation for future automated fungus-detection analysis.

**Conclusions:**

This work highlights the potential of OCT, combined with ML tools, to enable rapid, non-invasive, and early diagnosis of crop fungal infections, opening new avenues for precision agriculture and sustainable disease management.

**Supplementary Information:**

The online version contains supplementary material available at 10.1186/s13007-025-01411-7.

## Introduction

Wheat is cultivated in about 122 countries, with China, India, and the USA being major producers [1–3]. In the UK, wheat constitutes 58% of crops grown and yields approximately 15 million tons annually [4–6]. Its rich nutritional content makes it a key source of protein, carbohydrates, and fibers, forming the basis of foods like bread, pastries, and pasta [2, 7–10]. Wheat is, however, susceptible to various diseases amongst which the most potent are the wheat blast, *Fusarium* Head Blight, and *Zymoseptoria tritici* [11–14]. The latter especially is a devastating fungus which can cause up to 40% yield loss in wheat crops [15]. *Septoria* is thus a major concern for agriculture in the UK and Europe. Furthermore, this fungus propagates rapidly under favorable humid conditions, which is specifically relevant to the UK and continental Europe. Global efforts to help farmers anticipate *Septoria* outbreaks are being actively developed. These measures focus on both prophylactic and curative strategies. For example, the Agriculture and Horticulture Development Board (AHDB) [16] and UK Crop Science [17], which conducts thorough research on *Septoria*, already provides with clear guidelines on how to prevent and how to treat *Septoria* outbreak. Such outbreaks are commonly controlled using fungicides [14]. However, the key to successful fungicide treatment is the timeliness of the treatment. Delaying the treatments until external symptoms are visible can significantly decrease their efficacy [18]. Yield recovery may be limited to 10–30% compared to preventative treatment, which can save up to 70–90% of potential yield [19–21]. With respect to preventive treatments, it has been shown that triazole-based products, for example, are sustainable and effective when applied before infection. But the overuse of such prophylactic strategies nevertheless results in a decline in treatments efficacy from 60 to 90% [20]. It is then critical to detect and treat the infection as early as possible so as to limit the overuse of fungicides while preserving crop yields [22].

**Fig. 1:**
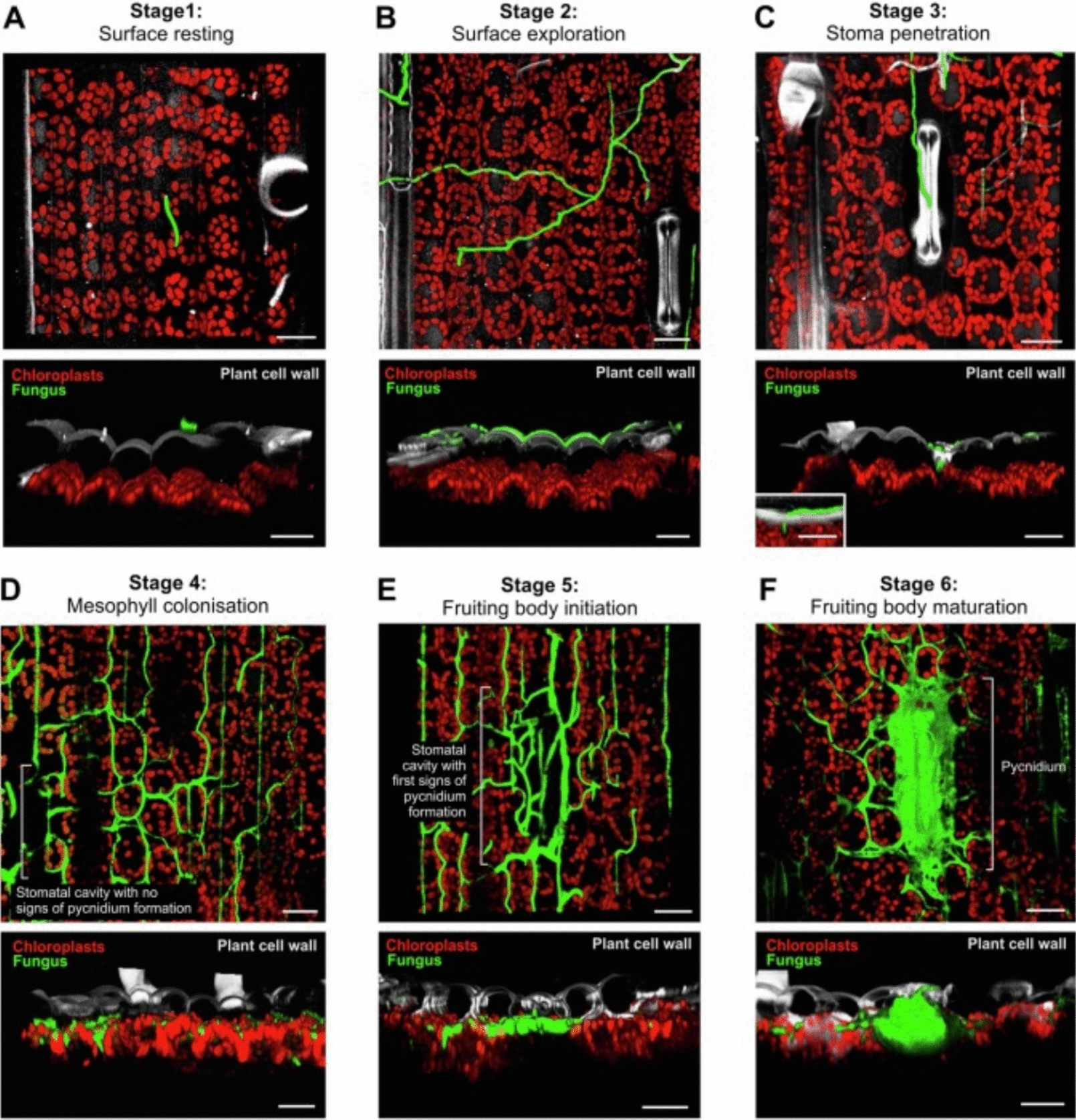
Confocal image stacks of infection process of *Septoria tritici* at different stages in wheat plants. The plants’ epidermis (grey) and chloroplasts (red) are detected by their auto-fluorescence. The green fluorescence is an effect of cytoplasmic eGFP expression in the cells of the fungus. Scale bars: 20 µm. Figure reproduced from [26]. **A** Stage 1,"Surface Resting": Spores settle on the surface of leaves. **B** Stage 2,"Surface Exploration": Spores form an infectious hypha to infect leaves through stomata. **C** Stage 3,"Stoma Penetration": Penetration of the host by the hyphae through the stomata apertures. **D** Stage 4,"Mesophyll Colonization": Colonization of mesophyll by fungus, but with no visible symptoms of infection. **E** Stage 5,"Fruiting Body Initiation": The hyphae grow and fills the inner space. This is a necrotrophic phase, where signs of infection on the leaf can be seen. **F** Stage 6,"Fruiting Body Maturation": The substomatal cavity fills with filaments, fruiting body, and pycnidium, to initiate spore production

The fungus of concern in this study is formerly known as *Mycosphaerella graminicola* also known as *Zymoseptoria tritici* is the pathogen causing *Septoria tritici blotch* (STB), and results in yellow necrotic spots on the leaves [23]. STB life cycle is expected to last about three to four weeks in open-air fields [24, 25]; When infection occurs, spores develop into hyphae which enter through the leaves stomata and proliferate within the mesophyll as depicted in Fig. 1.

After colonizing the whole leaf, STB grows into fruiting bodies (pycnidia), through an asexual sporulation, to give fungal spores at the tip of hyphae (conidia) [27]. The symptoms, e.g. yellow spots that turn brown, usually appear on the leaves within two to three weeks only after infection [28, 29]. It is the subsequent necrosis of the leaves and the plant that causes significant yield loss every year [30]. It is important to note that the different stages coexist [26]. Since a single hyphae penetration (stage 3) suffice to initiate colonisation of the mesophyll (stage 4), it is expected that surface exploration (stage 2) of the majority of the hyphae, which have not yet “found” stomata to enter, progresses concomitantly with the first colonisation event.

There exist already various techniques to help detect and quantify STB. On the one hand, the most accurate includes imaging techniques, such as high-resolution microscopy [54], and molecular testing, such as polymerase chain reaction (PCR) [31]. However, these techniques are also the most cumbersome given the necessity to process the sample beforehand and the need to access large-scale facilities. On the other hand, more practical and field-applicable techniques often suffer from lower precision. For example, RGB Imaging [32] can be useful for plant health studies but is generally insensitive to early-stage infections [33]. In another instance, multi- or hyperspectral imaging (HSI) is increasingly used in field [34–36]. These techniques are non-invasive and field-deployable, which makes them ideal for remote evaluation of a crop’s health [37, 38]. These techniques rely on the spectral changes that are either intrinsic to the plant (e.g. via changes in fluorescence [39]) or surface level (e.g. via changes in pigmentation [33]). However, spectral changes are direct consequences of molecular alteration, and thus, they occur when the plant is already prone to severe stresses, and oftentimes, already damaged [40]. However convenient and reliable, HSI thus detects the spectral signature associated with the chlorosis of wheat leaves [41]. It thus assesses the extent of infection within an already damaged crop. The same impediment is true when using other indices such as temperature and humidity, since they are primarily based of spectroscopic data [42]. Ideally, we require a technique capable of detecting early stages of infection, before the plant shows any external signs of stresses. To this end, we suggest using OCT instead, to benefit from its non-invasiveness, its real-time imaging, and potential field applicability. OCT is commonly used in the medical field, and more specifically in ophthalmology. However, given the advantages of OCT, i.e. non-invasive, in-vivo, 3D rendering, and real-time imaging [43], it is equally suitable for plants [44]. Given the practicality of the technique, OCT is increasingly used in plant imaging for various purposes. It is for example used for straightforward non-invasive assessment of plant’s internal structure [45] as well as for investigation of plant’s response to biotic and abiotic stressors [46, 47]. OCT can even be used for live responses to stressors, with a temporal resolution ranging from several days [48] down to hours [49, 50] and even seconds [51]. Furthermore, benefitting from a simple technical layout and robust optical components, OCT suitable to multimodal imaging [44]. Multimodal OCT variant includes for instance, polarization-sensitive OCT [52], spectroscopic OCT[53, 54], biospeckle or dynamic OCT [55, 56], and inverse spectroscopic OCT [57, 58]. In each case, the acquired data is further processed to provide an added layer of contrast, which can help differentiate structural elements that would otherwise remain indistinguishable.

We here demonstrate the suitability of standard OCT by using a low-cost compact commercial system to acquired cross-sectional images of leaves (~ 6 × 2 mm) with a ~ 10 µm resolution. This integrated system is considered low-cost (< £10 k) [59] compared to the better performing ones which starts at £40 k onward [60]. And although the system only resolves the first 3–5 cell layers, this resolution is sufficient to monitor internal structural differences between intact and infected leaves. The project thus consists of examining the internal structure of leaves through cross-sectional OCT images. The hypothesis is to indirectly monitor the growth of the fungus within the mesophyll, which is expected to push apart the different cell layers. And although the fungus filaments are too small (~ 2 µm in diameter [61]) to be seen with the current OCT resolution, the overall structure of the mesophyll is readily monitored.

In this work, we suggest analysing differences in mesophyll structure between control and infected leaves to provide insights about the state of infection and tissue integrity. In healthy control leaves, thinner and more uniform cell layers typically indicate intact tissue structure. Conversely, in infected leaves, the monitored increase in layer thickness and irregularity of the cell layers may suggest structural degradation, possibly due to the accumulation of fungal material between the cells [62, 63].

## Material and methods

The wheat for this proof-of-concept experiment belongs to the *Avalon* and *Cadenza* (AxC) 169 variety. This variety lacks a resistance gene against *Septoria*, making it more susceptible to *Septoria*, and ensuring effective pathogenesis. The seeds are grown in M3 compost supplemented with 0.5 g osmocote. Six plants were grown for this specific experimental run: three control plants and three plants destined to be infected. The plants were incubated at 20 °C, in a 14-h:10-h a light–dark cycle, at 61% of relative humidity, and levels of carbon dioxide was kept at 455 ppm (i.e. 55 ppm above the usual ~ 400 ppm outdoors level, which enhances plant growth [64]), these adjustments were made as part of the experimental conditions set for all growth chambers. The inoculation was performed when the plants were 21-day old.

Regarding the inoculum, *Zymoseptoria tritici* IPO323 [65] was incubated during 45–60 days on potato dextrose broth (PDB) media composed of 24 g/L of PDB and 15 g/L f Agar mixed with 1000 ml of ultrapure water (resistivity 18 MΩ·cm, Type I). Inoculation was performed via spray to mimic the natural spread of spores in high humidity atmosphere. The spray solution is prepared by adding 10 mL of 0.01% Tween 20 in water to a petri dish containing black heads (pycnidia spores). The spores are then gently scraped off using a sterile spatula and poured into a Falcon tube [17, 65]. Twenty µL of the supernatant was placed onto a counting chamber, ensuring the liquid spread evenly between the chamber and the cover slip. Excess liquid was removed using tissue paper. Spores were observed at 20X magnification using a Leica microscope. After allowing the spores to settle, they were counted, and the inoculum was adjusted accordingly, using sterile Tween 20 water, to achieve a concentration of 1 × 10⁶ spores/mL [36].

For inoculation, the plants were taken out of the growth chamber and placed inside a laminar airflow. The inoculum was sprayed on the top sides of the second newest leaf (GS31, following Zadoks system [66]). After inoculation, plants were covered with propagator lids (to maintain high humidity). The plants were then watered and placed in a sealed propagator and placed back in the growth chamber for 24 h recovery [67]. Although the control plants were left untreated, both the inoculated and controlled plants were regularly sprayed with purified water, via the control system of the growth chamber, so as to keep a high (61%) humidity level.

Scanning electron microscope (SEM) is used for high resolution imagining of surface morphology[68, 69]. In this study, it was used specifically to verify the state of infection at later stages. This study made use of a Hitachi TM3030Plus benchtop SEM.

**Fig. 2:**
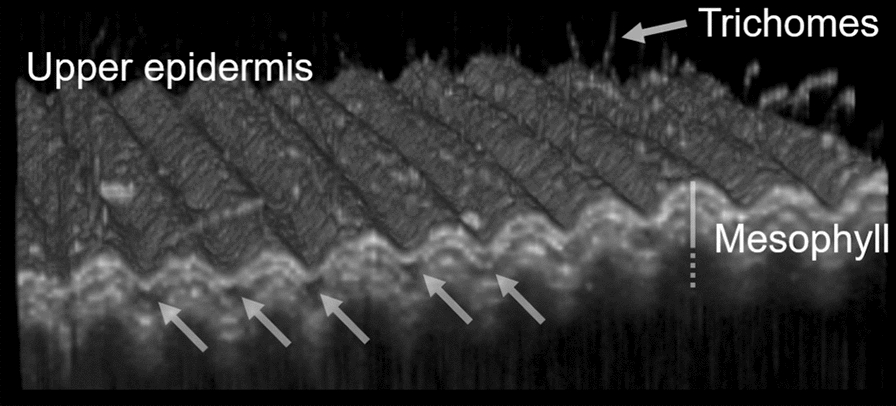
3D OCT images (C-scan) of a control wheat leaf. Each spike above the upper epidermis represents a trichome. Only the first few cell layers of the mesophyll are distinguishable. The arrows point to the “gaps” discussed subsequently. The image has a range of 5 mm x 5 mm and is generated using 300 consecutive b-scans, each separated by ~ 0.017 mm

The OCT system used is an OQ LabScope, version 2.0, from Lumedica using a superluminescent diode with central wavelength at 840 nm. The system generates 512 × 512-pixel images, with axial resolution of ~ 6 μm, and a lateral resolution of 15 μm. Daily OCT scans were collected to monitor the progression of the infection for a 14-day period after inoculation. Readings were taken from the three infected plants and from the three control plants. Scanning was performed midway along the leaf’s length, beside the main vein. Incomplete scans (i.e. in which the edge of the leaf appear) are dismissed for the automated analysis. A typical volumetric (c-scan) is shown in Fig. 2.

This study focusses on analysing the extend of the dark regions appearing within the mesophyll, called “gaps”, as shown in Fig. 2 (marked by arrows), by first manually processing the OCT scans using the FIJI image analysis software. Building upon the encouraging results from the manual analysis, a machine learning (ML) algorithm was developed in collaboration with Cyber Infrastructure Systems (CIS, http://www.cisin.com) to automatically segment these apparent gaps and classify the leaves. The Python code designed for OCT segmentation is a PyQt5-based GUI application that uses OpenCV, TensorFlow, NumPy, and Pandas for image processing and ML-based analysis. After training, the U-Net model (unet_masking3.keras) is used for generating segmentation masks via MaskThread class. The code is provided in supplementary information (SI), and the software is made available for download following this link:

https://drive.google.com/drive/folders/1DJm3OZHfK-P-XSRXGMtpxgSx51WnVNsF?usp=sharing

In both the manual and the automated procedure, the analysis focuses on the thickness of these apparent gaps between the second and third upper layers of the mesophyll.

## Results

**Fig. 3:**
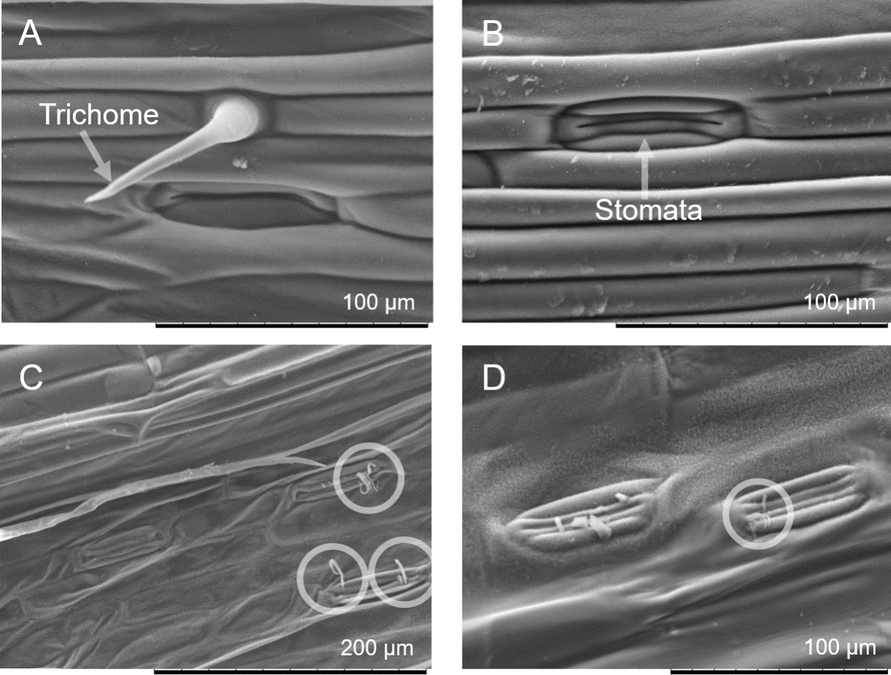
SEM images showing control wheat plant (**A**, **B**), and infected wheat plant 12 days after inoculation by *Septoria* (**B**, **C**). The circled hyphae emerging from the stomata pores illustrate an advanced colonisation (stage 4) with signs of necrosis (deflated cells, stage 5)

The effectiveness of the inoculation procedure is demonstrated in Fig. 3, where filaments can be seen emerging from the stomatal pores of infected leaves.

**Fig. 4:**
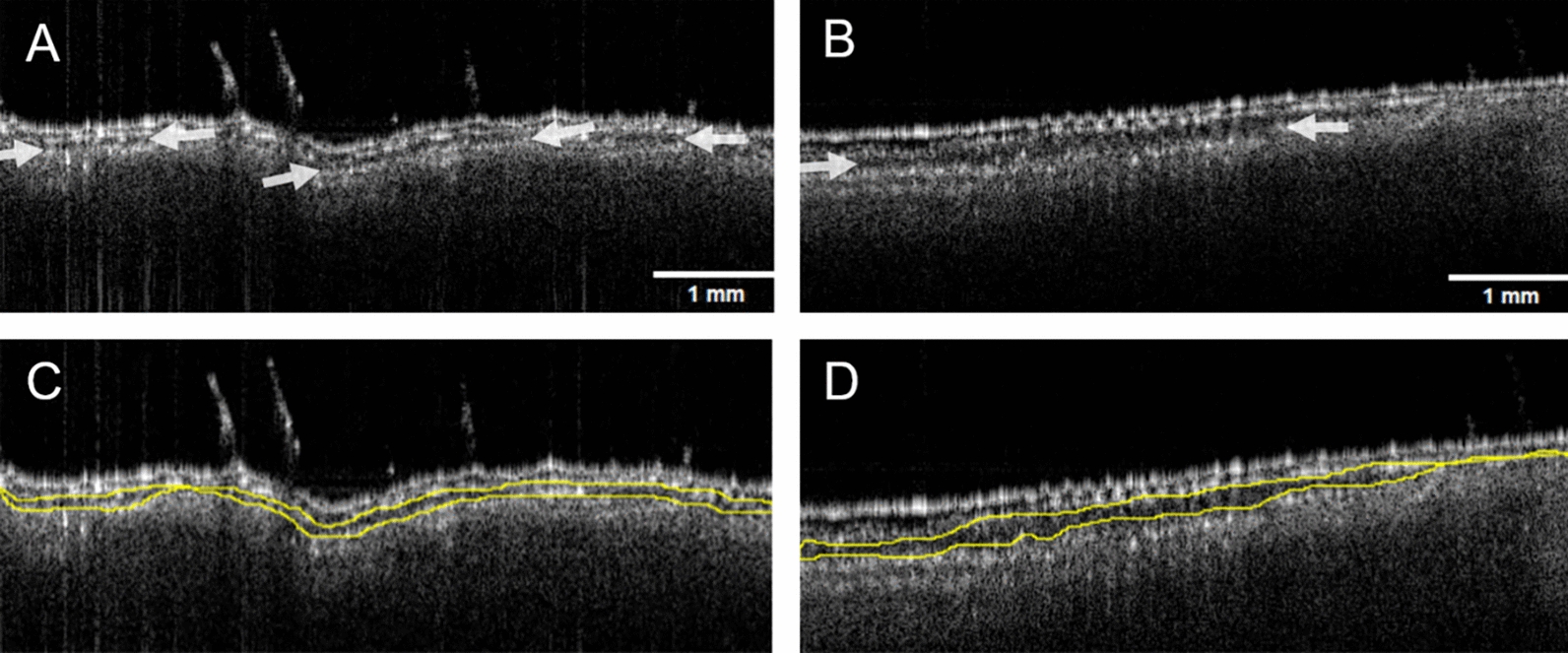
Individual OCT cross section (B-scan) of control wheat leaf (**A**, **C**) and infected wheat leaf taken 3 days after inoculation (**B**, **D**). The arrows in A and B indicate the gap between the second and third cell layers. Scale bars represent 1 mm. The yellow contour in C and D are examples of manual segmentation. Scale bar represents 1 mm.

It is also worth noting that healthy leaves already have air gaps within their mesophyll (as shown in Fig. 2) used to facilitate gas exchange [70]. These air gaps, which are expected to appear dark in OCT B-scan, are thus indistinguishable with the low-density components of the cells (e.g. the cytoplasm). All what OCT scans shows are regions of high density (e.g. cell’s nucleus and vacuole). The apparent gaps monitored in OCT images, shown in Fig. 4, thus correspond to low-density regions, which includes the air network, the surrounding of the plant cells’ nucleus and vacuole, and possibly the fungus hyphae. These apparent gaps are however highly heterogeneous and not easily distinguished given the uneven leaf morphology. As such it was decided to restrict the analysis to the thickness (or height) of the apparent gap.

These apparent gaps notably increase when the plant is infected, while the leaves are still green and seemingly healthy, as shown in the subsequent Table 1. Table 1 shows the average thickness of the gaps present in a selection of 20 OCT B-scans out of each volumetric reading, for both control and infected leaves, ranging from day 0 (i.e. right before inoculation) to day 1 (i.e. 24 h after inoculation) and up to day 7. Every day, one reading was performed per plant, on the three different controlled and on the three different infected plants.

**Table 1:** Mean thickness of the gaps from day 0 (right before inoculation) to day 7 (after inoculation) of control and *Septoria*-infected wheat leaves (One reading per plant, on three different controlled and three different infected plants)

	Control			Infected		
	R1	R2	R3	R1	R2	R3
Day 0	0.055 ± 0.018	0.046 ± 0.016	0.048 ± 0.017	0.045 ± 0.015	0.0463 ± 0.015	0.049 ± 0.017
	
Day 1	0.044 ± 0.015	0.038 ± 0.015	0.041 ± 0.014	0.05 ± 0.014	0.0543 ± 0.009	0.05 ± 0.015
	
Day 2	0.039 ± 0.014	0.041 ± 0.014	0.043 ± 0.016	0.051 ± 0.019	0.068 ± 0.026	0.067 ± 0.024
	
Day 3	0.039 ± 0.014	0.039 ± 0.013	0.042 ± 0.014	0.067 ± 0.024	0.075 ± 0.027	0.082 ± 0.028
	
Day 4	0.042 ± 0.015	0.049 ± 0.015	0.046 ± 0.013	0.076 ± 0.024	0.082 ± 0.03	0.073 ± 0.024
	
Day 5	0.049 ± 0.02	0.049 ± 0.02	0.054 ± 0.02	0.068 ± 0.03	0.069 ± 0.03	0.083 ± 0.03
	
Day 6	0.05 ± 0.017	0.047 ± 0.018	0.053 ± 0.02	0.082 ± 0.04	0.063 ± 0.024	0.067 ± 0.02
	
Day 7	0.044 ± 0.02	0.04 ± 0.017	0.046 ± 0.02	0.073 ± 0.024	0.083 ± 0.03	0.07 ± 0.024
	

Every mean value is an average of 250 individual thickness measurements taken manually from a selection of 20 OCT B-scans out of each volumetric reading (C-scan). For each day, a single image of the leaf (out of the three available control and infected plants) is shown to appreciate the lack of external symptoms until day 7.

**Fig. 5:**
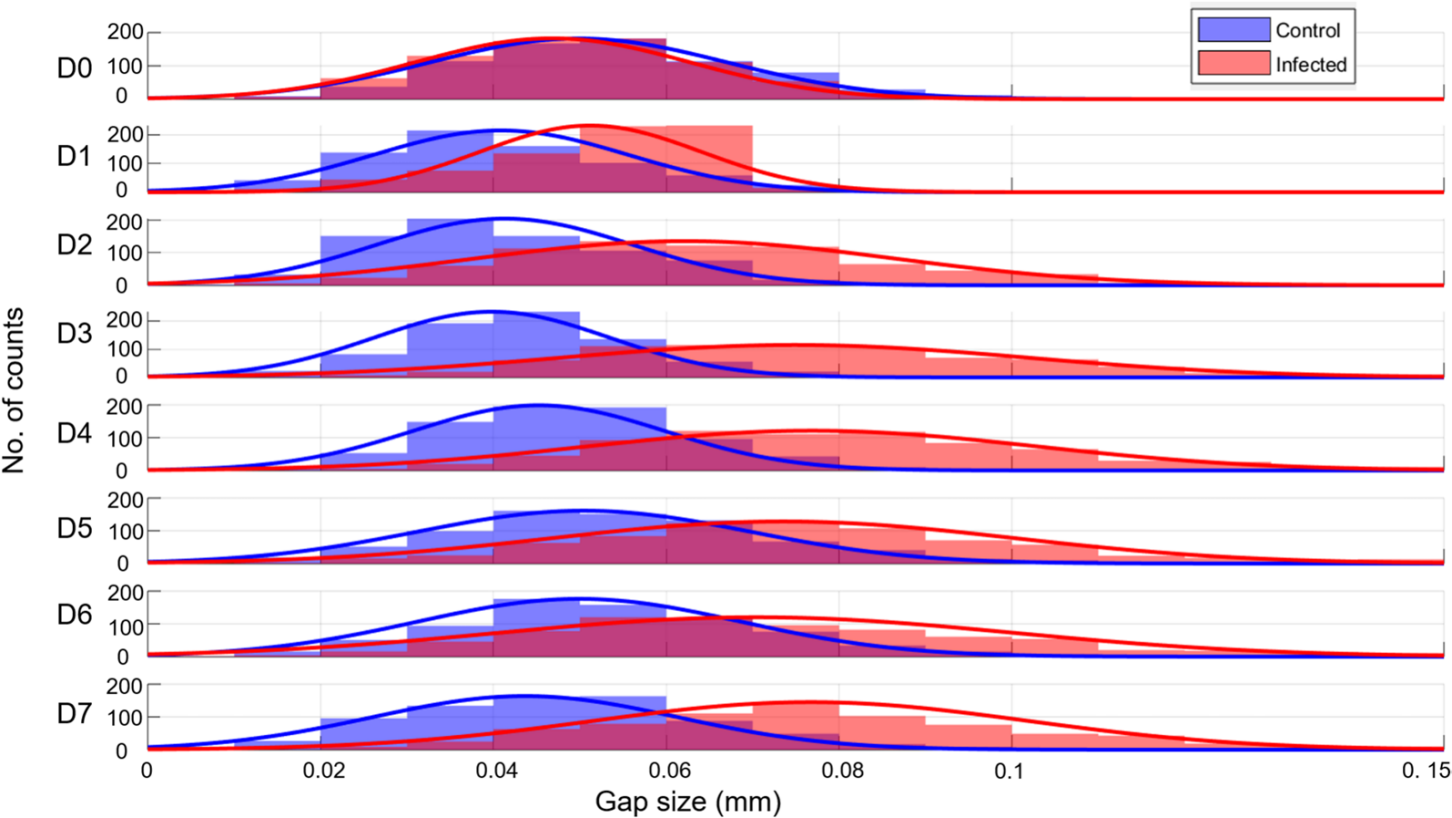
Gap size distribution for manual thickness measurements from day 0 (D0) to day 7 (D7). Superimposed histograms of control (blue) and infected leaves (red) groups with their Gaussian fits. The histograms used a bin width of 0.01 mm.

Only the first 7 days are here presented, while the leaves do not show any visual signs of infection. Images of the subsequent chlorosis and necrotic stages, from day 8 to 14, can be found in SI. When analysing the individual measurements used to compute the averages shown in the above table, the measured gap thicknesses reveal distinct trend between control and infected leaves, as depicted in Fig. 5. From day 2 after inoculation onward, infected leaves exhibit consistently larger gap thicknesses, exceeding 0.05 mm in average, while measurements on control leaves remains below or around that value. Furthermore, the width of the Gaussian-fit for the infected leaves group is typically broader (FWHM ~ 0.2 compared to that of the control group (FWHM ~ 0.15). Accordingly, the apparent gaps in infected leaves are more heterogenous than those in control leaves.

The superimposed histograms from the control and infected groups reveal distinct statistical differences in the gap size and distribution. From the average gap values, we could theoretically set a threshold value for the average gap’s thickness of 0.05 mm, above which the leaf is classified as infected. If such was the case, effective assessment of infection could already be made from day 1 and affirmed from day 2 after inoculation.

**Fig. 6:**
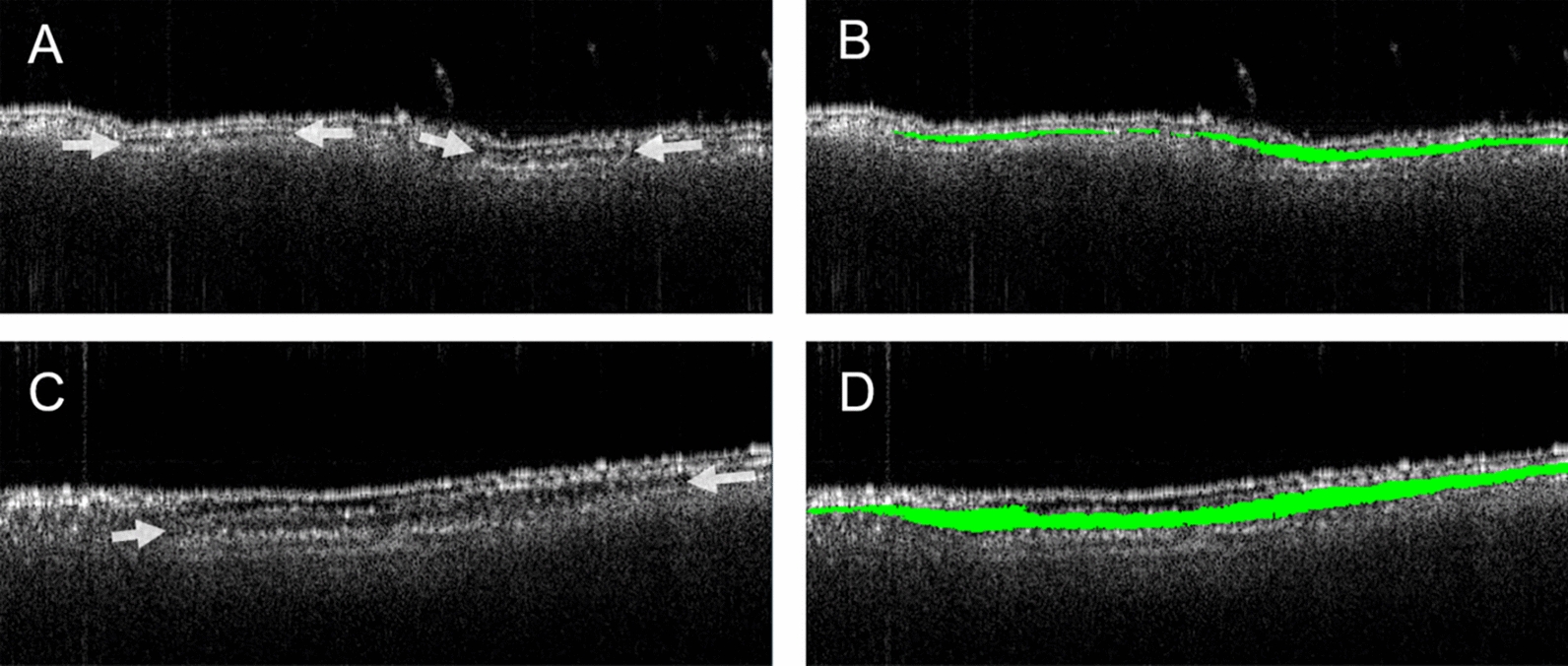
Examples of automated segmentation using OCT image analysis to classify the spacing between the second and third cell layers in a control wheat leaf (**A**) with its segmented area (**B**, green overlay), and an infected leaf (**C**) with its segmented are (**D**, green overlay), 2 days after inoculation.

To further benefit from OCT fast scanning rate and systematically processing large stacks of OCT images, a bespoke machine learning (ML)-based software for image segmentation was used instead of the manual labelling. The automated analysis is based on the same concept as the previous manual analysis: it aims to segment the apparent gap between the second and third cell layer and compute its averaged thickness. Example of the automated segmentation is shown in Fig. 6.

**Fig. 7:**
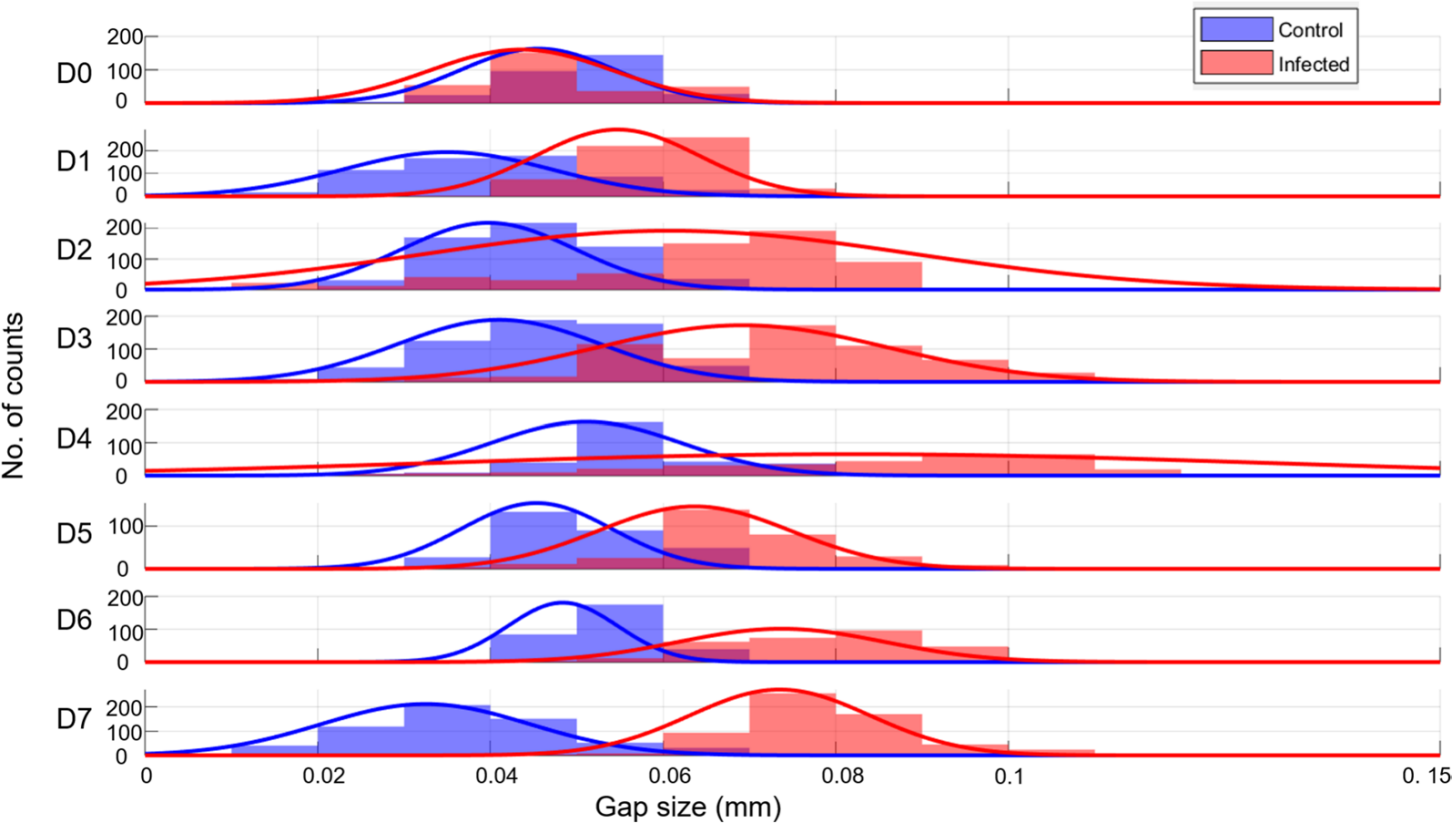
Average gap size distribution in control (blue) and infected (red) wheat leaves extracted from automated segmentation, from day 0 (D0) to day 7 (D7) after inoculation, with superimposed Gaussian fits. The histograms use a bin width of 0.01 mm

In comparison to the previous analysis, for each day and for each of the three OCT volumetric (C-scan) readings on control and infected plants, 200 images are selected (instead of 20 for the manual analysis) and analysed using the ML-based software. The computed average thickness of the segmented gap from each image is used to build the histograms shown in Fig. 7.

The results from the ML-based image analysis appear more scattered compared to the previous manual analysis. This scattering might be a direct consequence in the apparent difficulty in adequately segmenting the OCT images, which is in part due to the uneven leaf structures, as discussed subsequently. Although histograms resulting from the automated segmentation analysis are not as consistent compared to those generated from the manual analysis, a similar pattern nonetheless emerges. Starting from day 1 after inoculation, the apparent gaps in infected leaves are statistically and consistently larger compared to those in intact leaves. And similarly, the widths of the Gaussian fits in infected leaves are also larger compared to those in controlled leaves.

It is also important to note that the ML-based segmentation software was here trained using the AxC 169 variety. The same automated procedure was used on images taken from another variety (AxC 157, same parentage but different genotype) and yielded, to a lesser extent, similar results (i.e. broader Gaussian fit and shifted Gaussian centre toward larger gap size for infected leaves, results shown in SI). One main difference between the two varieties is that intact leaves from cultivars AxC157 already have larger apparent gaps between the second and third cell layers (of ~ 0.06 mm). Therefore, the distinction between infected and control became challenging when considering only the average gap size, as it will be discussed subsequently.

## Discussions

Our objective is to benefit from OCT ability to see through soft tissues to evaluate the state of infection over time. Although various features of the leaves were investigated, such as spacing between cell layers, cell appearances, number and length of trichomes, this study focuses on the former due to the absence of clear trend and difficulties to capture accurate data for the others. This study therefore solely reports on the average gap size within the mesophyll, with the aim of correlating the monitored changes with fungal growth. And while OCT cannot directly visualize fungal hyphae, this study reveals that the apparent spacing between cell layers increases where the fungal hyphae are expected to develop. Indeed, damaged tissues have a lower refractive index compared to healthy cells [71–73]. As a result, it is possible that the damaged tissues scatter less light compared to healthy ones and thus appear darker or dark (i.e. as a gap) on the OCT scans.

This increase in gap thickness and heterogeneity of the gap size is thus expected to be a direct result of the mesophyll colonisation by the hyphae (stage 4). As discussed earlier, colonisation of the mesophyll is initiated by the very first hyphae intrusion through a stoma (stage 3). The fact that we start monitoring an increase in gap thickness as early as 24 h after inoculation (instead of day 3, as per Fantozzi, E., et al. [26]) can be a result of our choice of cultivar. Indeed, AxC 169 was chosen because of its increased vulnerability to *Septoria*. It is also possible that inoculation via spray facilitates entry of the developing hyphae through the stomata. It has been shown that spore germination typically occurs within 12 h after contact with a leaf when humidity levels are high [30]. And while the presence of water droplets is not expected to trigger any direct reaction from the stomata, the overall increased humidity due to the spray implies that most stomata will be open [74], thus facilitating entry of the hyphae within the first few hours. It is important to note that our controls were only water sprayed, in accordance with previously published works, also on wheat, and similarly infected by *Septoria* [75–78]. The spraying of purified water was automatically performed within the chamber to preserve a humidity level of 61%. It is worth highlighting that both control and inoculated plants are regularly sprayed with purified water to maintain the high humidity level. Furthermore, the estimated concentration of Tween in the final spore-loaded solution is about 0.01%, which is significantly less compared to the 0.1% and 1% used in the previous works on wheat infected by *Septoria* in which water-sprayed controls were also used. While we do not expect such a low Tween concentration to trigger a systemic response, we do expect the colonisation by *Septoria* to trigger such a response. Because the response monitored (increase in gap size) seems to reach a maximum by day 2, as illustrated in Fig. 5, and does not change until necrosis happens, we are confident that the effects measured are triggered by the pathogen rather than the surfactant or by the spraying procedure. It is, however, possible that upon the first intrusion event, before full colonisation takes place, the whole plant reacts by modifying its mesophyll structure, which might give rise to the increased in apparent gap thickness. If such is the case, this study still demonstrates that OCT can effectively be used to detect early signs of infection. This work thus demonstrated that monitoring intercellular spacing via OCT enables distinct classification between infected and intact plant from day 2 after inoculation, while visible signs of necrosis only appear on day 7.

In comparison to alternative non-invasive and similarly field-applicable methods, OCT has the advantage of being sensitive to changes that precede any discolouration or alteration of the plant’s spectral signature. Indeed, the remote monitoring of the plant’s pigmentation, fluorescence, temperature or water level often rely on multi- or hyperspectral data analysis [37, 38, 42]. It is difficult to directly compare our results with field applications of multi- and hyperspectral monitoring because the chosen variety is inherently more susceptible to *Spetoria*. The *Septoria* life cycle is consequently shorten compared to that in actual crops. But the fact that we can monitor infection-driven changes before any external signs appear demonstrates that detection via OCT precedes detection via multi- or hyperspectral techniques. As exemplified in Fig. 5 and 7, a distinction between infected and non-infected is monitored at least 5 to 6 days before the plant displays any yellow spots (shown in Table 1, day 7).

We concede that both methods, the manual labelling and the ML-aided labelling, are prone to biases. On the one hand, for example, manual labelling tends to focus on areas where a gap is clearly visible, thus neglecting sections of the mesophyll which may display a relatively smaller gap within the same B-scan. On the other hand, the automated segmentation is challenged with uneven leaf surfaces. The benefit of the ML-based analysis over the manual labelling is evidently its systematicity and its ability to analysed data at high throughput. Overall, whether manual or ML-based, the current analysis, with its unique distinguishing parameter, is already promising in differentiating between infected and intact plants.

We remind that our ML-based model was trained and tested on AxC 169. When the same model was applied to AxC 157, it led to more mitigated conclusions (see SI). Indeed, the fact that intact AxC 157 leaves already display gaps that are similar in size to those monitored in infected plants indicates that a single distinguishing parameter (i.e. gap thickness) might not be sufficient when assessing differing cultivars. To circumvent this limitation, it is planned to pursue such studies to include more parameters, such as to take into account the 3-dimentional shape of the gaps since it is readily available from the OCT volumetric C-scans. Additionally, we hope to improve training model by expanding our study to multiple wheat varieties. Furthermore, while the plants in our study were grown under controlled conditions, we acknowledge that field environments will introduce additional variability, such as fluctuating temperatures, humidity levels, soil composition, and pathogen exposure that cannot be fully replicated in a growth chamber. It is thus necessary to study the impact of these environmental factors onto the mesophyll to further evaluate OCT’s field applicability. Nevertheless, the core physiological and structural responses observed in this work (e.g., mesophyll disruption detectable by OCT as early as two days after infection) are expected to manifest similarly in field-grown plants.

## Conclusions

In this proof-of-concept experiment, we demonstrate low-cost OCT to be ideally suited to monitor minute structural changes within the mesophyll. OCT is here used to effectively detect the increased intercellular spacing induced by the inoculation of pathogens. The monitored changes, taken as direct indication that the leaf is being colonized by the pathogen, demonstrate that OCT can be used to detect infections even before any visible signs appear on the plant. Systematic analysis of several thousands of images acquired, over time and non-invasively, is made possible by using bespoke ML-based analysis. And although the analysis currently relies on a single parameter (apparent gap thickness), statistical differences between controlled and infected leaves is established from day 2 after inoculation. Accordingly, OCT imaging holds the promise for quick and accurate identification of infection, even at an asymptomatic stage. The application of OCT coupled with ML analysis thus presents a valuable opportunity to effectively assess crop health. By harnessing and combining these cutting-edge technologies, this work contributes to advancing agricultural practices by providing with a tool that may enable timely treatments, help reduce crop losses, and achieve global food security.

This work also highlights current limitations of using OCT for detection of infection. OCT cannot directly visualize fungal hyphae, and its penetration depth may be insufficient for imaging thicker plant tissues. Furthermore, ML-based segmentation models require further validation to ensure it is applicable across different plant species and can distinguish between the various biotic and abiotic stressors. Despite these challenges, this research highlights the promise that OCT holds, when combined with ML analysis, as a non-destructive, real-time imaging technique to transform precision agriculture and promote sustainable crop disease management.

## Future work

While the resolution of the OCT images is set by the system optoelectronic components, the current software used for image segmentation can be readily improved. The ML based analysis software was trained specifically on a single wheat variety (AxC 169). Future work will expand the dataset to include multiple wheat varieties and potentially correlate with other plant species to improve the robustness of the model. Further experimental runs are also required to disentangle the morphological responses triggered by environmental factors such as variability in temperature and in humidity, as well as variability in soil and atmospheric composition. Additionally, to differential between multiple stressors, it may become required to incorporate other measurables, such as gap density, gap volume and overall structural heterogeneity of the leaf. Including multiple parameters in the analysis could significantly advance the technique’s precision. Given these necessary improvements, the path to make OCT an integral item in a farmer’s toolbox is still long but is definitively underway.

## Supplementary Information

Supplementary File 1

## Data Availability

Analysed data is provided within the manuscript or supplementary information files. The ML-base segmentation software is accessible via Google Drive: https://drive.google.com/drive/u/0/folders/1DJm3OZHfK-P-XSRXGMtpxgSx51WnVNsF The raw data (OCT B-scans of wheat leaves) is provided upon request.
