# Supplementary material for: Optical coherence tomography for early detection of crop infection
Source: Plant Methods. 2025 Jul 6;21:92. doi: 10.1186/s13007-025-01411-7 (PMC12232840; doi:10.1186/s13007-025-01411-7)
Supplement: Supplementary file 1 [file 13007_2025_1411_MOESM1_ESM.docx]

**Optical Coherence Tomography for Early Detection of Crop Infection -** **Supplementary Information**

Ghada Salem Sasi^1^, Stephen J. Matcher^2^, and Adrien Alexis Paul Chauvet^1,^ *

^1^ School of Mathematic and Physical Sciences, University of Sheffield, Sheffield S3 7HF (UK)

^2^ School of Electrical and Electronic Engineering, The University of Sheffield, 3 Solly Street, Sheffield, S1 4DE (UK)

* Correspondence: [a.chauvet@sheffield.ac.uk](mailto:a.chauvet@sheffield.ac.uk)

This Supplementary information file includes:

- (1) images of the wheat plants after inoculation by *Septoria*, p.2
- (2) table of individual manual measurements for the wheat AxC169 variety, starting p.3
- (3) table of measurements produced by the automated segmentation software for the wheat AxC169 variety, starting p.39
- (4) table of measurements produced by the automated segmentation software for the wheat AxC157 variety, starting p.83, with its corresponding histogram, p.85
- (5) Rational behind the choice of methods used in the segmentation software, p.86
- (6) The Python code of the ML segmentation software, starting p.87

1. **Images of wheat leaves in function of days after inoculation.**

Only one leaf is shown per set of controlled (mock) and infected plants.


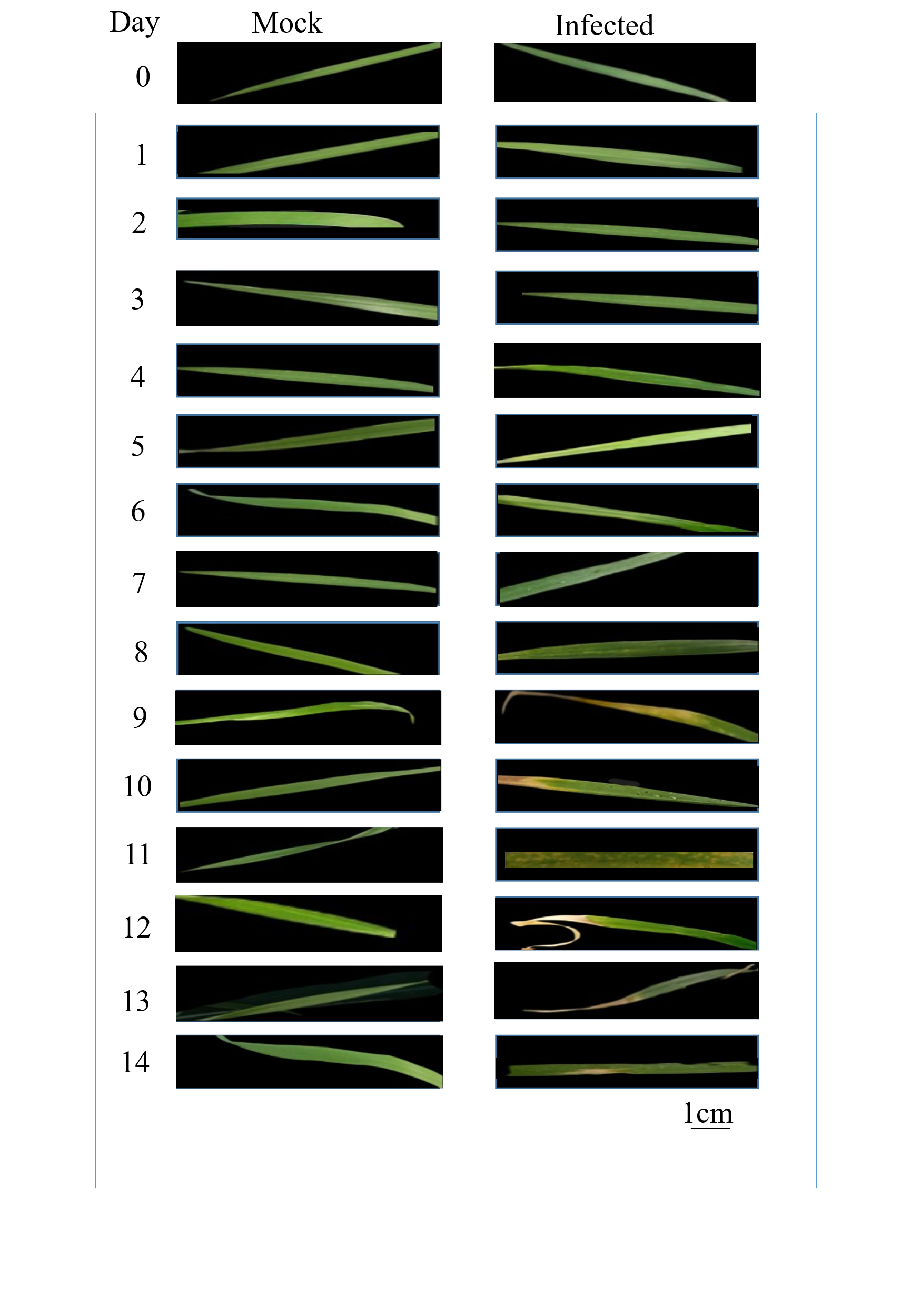


Figure 1: depicts the various stages that wheat leaves, starting at 21-day-old, undergo when exposed to Septoria tritici. Visible yellow spots begin to form on day 8, rapidly increasing until they develop into damaged areas in the later stages of infection, from day 9 to day 14.

1. **Manual gap thickness measurements for AxC169 variety**

The following tables illustrate the total number of OCT images used for manual gap thickness measurements in the AxC169 variety. A total of 250 OCT images were analyzed, with segmentation of the first 2–3 cell layers performed using the Freehand Tool in FIJI. For both control and infected leaves, three readings were taken from Day 0 (D0) to Day 7 (D7).

| Day 0  Before inoculation | | | | | | | Day1 | | | | | |
| --- | --- | --- | --- | --- | --- | --- | --- | --- | --- | --- | --- | --- |
| OCT image | Control | | | Before inoculation | | | Control | | | Infected | | |
|  | 1 | 2 | 3 | 1 | 2 | 3 | 1 | 2 | 3 | 1 | 2 | 3 |
| 1 | 0.08 | 0.08 | 0.084 | 0.06 | 0.05 | 0.052 | 0.031 | 0.061 | 0.031 | 0.051 | 0.05 | 0.022 |
| 2 | 0.071 | 0.05 | 0.08 | 0.02 | 0.057 | 0.042 | 0.048 | 0.039 | 0.055 | 0.05 | 0.051 | 0.034 |
| 3 | 0.05 | 0.042 | 0.07 | 0.033 | 0.033 | 0.03 | 0.061 | 0.034 | 0.038 | 0.055 | 0.055 | 0.039 |
| 4 | 0.07 | 0.04 | 0.098 | 0.03 | 0.06 | 0.052 | 0.027 | 0.038 | 0.027 | 0.05 | 0.055 | 0.052 |
| 5 | 0.091 | 0.04 | 0.01 | 0.06 | 0.04 | 0.05 | 0.058 | 0.027 | 0.07 | 0.039 | 0.05 | 0.039 |
| 6 | 0.081 | 0.02 | 0.042 | 0.075 | 0.04 | 0.04 | 0.026 | 0.039 | 0.039 | 0.059 | 0.055 | 0.013 |
| 7 | 0.057 | 0.03 | 0.091 | 0.04 | 0.048 | 0.05 | 0.039 | 0.027 | 0.055 | 0.055 | 0.057 | 0.038 |
| 8 | 0.07 | 0.033 | 0.08 | 0.07 | 0.024 | 0.061 | 0.033 | 0.033 | 0.059 | 0.044 | 0.05 | 0.049 |
| 9 | 0.057 | 0.02 | 0.048 | 0.057 | 0.057 | 0.05 | 0.037 | 0.016 | 0.028 | 0.055 | 0.056 | 0.049 |
| 10 | 0.04 | 0.04 | 0.071 | 0.05 | 0.033 | 0.033 | 0.039 | 0.044 | 0.064 | 0.067 | 0.05 | 0.038 |
| 11 | 0.05 | 0.052 | 0.052 | 0.07 | 0.061 | 0.04 | 0.023 | 0.039 | 0.023 | 0.053 | 0.054 | 0.034 |
| 12 | 0.061 | 0.024 | 0.09 | 0.06 | 0.04 | 0.03 | 0.049 | 0.034 | 0.048 | 0.049 | 0.056 | 0.062 |
| 13 | 0.071 | 0.075 | 0.052 | 0.057 | 0.05 | 0.05 | 0.049 | 0.044 | 0.045 | 0.038 | 0.056 | 0.034 |
| 14 | 0.084 | 0.057 | 0.06 | 0.03 | 0.04 | 0.05 | 0.039 | 0.033 | 0.044 | 0.05 | 0.05 | 0.028 |
| 15 | 0.129 | 0.04 | 0.06 | 0.08 | 0.042 | 0.07 | 0.033 | 0.054 | 0.05 | 0.045 | 0.055 | 0.045 |
| 16 | 0.04 | 0.04 | 0.05 | 0.02 | 0.05 | 0.052 | 0.027 | 0.031 | 0.033 | 0.054 | 0.06 | 0.055 |
| 17 | 0.066 | 0.06 | 0.03 | 0.061 | 0.05 | 0.02 | 0.071 | 0.031 | 0.038 | 0.045 | 0.053 | 0.057 |
| 18 | 0.02 | 0.033 | 0.042 | 0.04 | 0.05 | 0.042 | 0.038 | 0.028 | 0.049 | 0.036 | 0.055 | 0.022 |
| 19 | 0.052 | 0.042 | 0.07 | 0.071 | 0.04 | 0.03 | 0.044 | 0.04 | 0.027 | 0.055 | 0.042 | 0.04 |
| 20 | 0.04 | 0.033 | 0.03 | 0.05 | 0.04 | 0.05 | 0.031 | 0.057 | 0.045 | 0.058 | 0.051 | 0.041 |
| 21 | 0.033 | 0.02 | 0.06 | 0.06 | 0.02 | 0.06 | 0.036 | 0.062 | 0.049 | 0.058 | 0.059 | 0.064 |
| 22 | 0.084 | 0.04 | 0.071 | 0.061 | 0.052 | 0.052 | 0.049 | 0.045 | 0.044 | 0.04 | 0.056 | 0.066 |
| 23 | 0.04 | 0.04 | 0.048 | 0.061 | 0.05 | 0.052 | 0.011 | 0.044 | 0.041 | 0.06 | 0.06 | 0.075 |
| 24 | 0.071 | 0.04 | 0.057 | 0.04 | 0.04 | 0.05 | 0.028 | 0.016 | 0.05 | 0.057 | 0.055 | 0.045 |
| 25 | 0.061 | 0.066 | 0.06 | 0.048 | 0.061 | 0.091 | 0.05 | 0.041 | 0.05 | 0.046 | 0.06 | 0.026 |
| 26 | 0.033 | 0.06 | 0.05 | 0.066 | 0.02 | 0.052 | 0.044 | 0.084 | 0.022 | 0.044 | 0.043 | 0.071 |
| 27 | 0.033 | 0.03 | 0.05 | 0.033 | 0.07 | 0.061 | 0.07 | 0.034 | 0.027 | 0.044 | 0.061 | 0.027 |
| 28 | 0.061 | 0.03 | 0.05 | 0.03 | 0.04 | 0.066 | 0.034 | 0.057 | 0.027 | 0.045 | 0.061 | 0.033 |
| 29 | 0.057 | 0.03 | 0.04 | 0.052 | 0.061 | 0.05 | 0.062 | 0.035 | 0.018 | 0.04 | 0.06 | 0.044 |
| 30 | 0.042 | 0.061 | 0.04 | 0.07 | 0.05 | 0.066 | 0.045 | 0.028 | 0.054 | 0.028 | 0.054 | 0.049 |
| 31 | 0.024 | 0.071 | 0.05 | 0.03 | 0.03 | 0.06 | 0.055 | 0.041 | 0.057 | 0.049 | 0.04 | 0.023 |
| 32 | 0.03 | 0.05 | 0.042 | 0.024 | 0.04 | 0.07 | 0.038 | 0.033 | 0.033 | 0.046 | 0.061 | 0.049 |
| 33 | 0.052 | 0.05 | 0.048 | 0.06 | 0.02 | 0.05 | 0.041 | 0.016 | 0.049 | 0.027 | 0.064 | 0.027 |
| 34 | 0.05 | 0.033 | 0.061 | 0.03 | 0.042 | 0.06 | 0.031 | 0.028 | 0.049 | 0.063 | 0.065 | 0.061 |
| 35 | 0.061 | 0.03 | 0.042 | 0.05 | 0.05 | 0.048 | 0.072 | 0.022 | 0.055 | 0.06 | 0.06 | 0.041 |
| 36 | 0.052 | 0.04 | 0.04 | 0.06 | 0.03 | 0.033 | 0.038 | 0.034 | 0.028 | 0.055 | 0.061 | 0.04 |
| 37 | 0.091 | 0.071 | 0.07 | 0.03 | 0.033 | 0.052 | 0.049 | 0.05 | 0.057 | 0.06 | 0.06 | 0.049 |
| 38 | 0.02 | 0.042 | 0.052 | 0.057 | 0.03 | 0.03 | 0.06 | 0.049 | 0.034 | 0.062 | 0.052 | 0.055 |
| 39 | 0.03 | 0.042 | 0.057 | 0.06 | 0.04 | 0.048 | 0.044 | 0.034 | 0.027 | 0.068 | 0.061 | 0.049 |
| 40 | 0.04 | 0.075 | 0.05 | 0.024 | 0.04 | 0.03 | 0.05 | 0.027 | 0.027 | 0.05 | 0.054 | 0.035 |
| 41 | 0.04 | 0.05 | 0.05 | 0.03 | 0.03 | 0.042 | 0.06 | 0.034 | 0.023 | 0.066 | 0.051 | 0.072 |
| 42 | 0.06 | 0.052 | 0.033 | 0.07 | 0.02 | 0.033 | 0.078 | 0.016 | 0.034 | 0.055 | 0.061 | 0.062 |
| 43 | 0.061 | 0.052 | 0.05 | 0.04 | 0.066 | 0.04 | 0.049 | 0.038 | 0.049 | 0.063 | 0.065 | 0.088 |
| 44 | 0.061 | 0.03 | 0.06 | 0.04 | 0.03 | 0.05 | 0.027 | 0.034 | 0.045 | 0.06 | 0.065 | 0.023 |
| 45 | 0.07 | 0.042 | 0.07 | 0.042 | 0.042 | 0.05 | 0.033 | 0.034 | 0.034 | 0.028 | 0.06 | 0.045 |
| 46 | 0.03 | 0.033 | 0.07 | 0.033 | 0.033 | 0.048 | 0.022 | 0.039 | 0.027 | 0.054 | 0.063 | 0.067 |
| 47 | 0.071 | 0.03 | 0.071 | 0.01 | 0.02 | 0.071 | 0.044 | 0.049 | 0.022 | 0.045 | 0.056 | 0.05 |
| 48 | 0.05 | 0.024 | 0.03 | 0.033 | 0.042 | 0.08 | 0.039 | 0.044 | 0.027 | 0.033 | 0.056 | 0.053 |
| 49 | 0.04 | 0.042 | 0.04 | 0.06 | 0.02 | 0.04 | 0.055 | 0.054 | 0.023 | 0.031 | 0.05 | 0.039 |
| 50 | 0.024 | 0.06 | 0.052 | 0.02 | 0.03 | 0.02 | 0.045 | 0.027 | 0.075 | 0.024 | 0.057 | 0.048 |
| 51 | 0.052 | 0.01 | 0.04 | 0.052 | 0.06 | 0.052 | 0.018 | 0.027 | 0.05 | 0.018 | 0.056 | 0.018 |
| 52 | 0.07 | 0.052 | 0.04 | 0.06 | 0.061 | 0.06 | 0.049 | 0.038 | 0.038 | 0.011 | 0.06 | 0.022 |
| 53 | 0.06 | 0.05 | 0.052 | 0.042 | 0.05 | 0.05 | 0.044 | 0.048 | 0.05 | 0.049 | 0.065 | 0.028 |
| 54 | 0.061 | 0.04 | 0.081 | 0.02 | 0.04 | 0.081 | 0.041 | 0.07 | 0.034 | 0.05 | 0.05 | 0.034 |
| 55 | 0.05 | 0.042 | 0.04 | 0.052 | 0.033 | 0.04 | 0.011 | 0.04 | 0.011 | 0.056 | 0.054 | 0.044 |
| 56 | 0.05 | 0.052 | 0.057 | 0.06 | 0.03 | 0.042 | 0.039 | 0.038 | 0.033 | 0.06 | 0.056 | 0.071 |
| 57 | 0.052 | 0.052 | 0.071 | 0.04 | 0.04 | 0.03 | 0.077 | 0.023 | 0.038 | 0.062 | 0.06 | 0.038 |
| 58 | 0.052 | 0.01 | 0.052 | 0.05 | 0.052 | 0.061 | 0.06 | 0.054 | 0.027 | 0.034 | 0.061 | 0.053 |
| 59 | 0.02 | 0.05 | 0.05 | 0.02 | 0.06 | 0.017 | 0.045 | 0.044 | 0.057 | 0.038 | 0.051 | 0.052 |
| 60 | 0.05 | 0.02 | 0.071 | 0.03 | 0.042 | 0.084 | 0.055 | 0.033 | 0.027 | 0.054 | 0.061 | 0.088 |
| 61 | 0.071 | 0.04 | 0.07 | 0.05 | 0.05 | 0.061 | 0.052 | 0.088 | 0.033 | 0.052 | 0.06 | 0.033 |
| 62 | 0.071 | 0.03 | 0.091 | 0.052 | 0.052 | 0.07 | 0.044 | 0.049 | 0.06 | 0.061 | 0.061 | 0.059 |
| 63 | 0.04 | 0.04 | 0.052 | 0.033 | 0.02 | 0.052 | 0.055 | 0.052 | 0.044 | 0.041 | 0.06 | 0.061 |
| 64 | 0.061 | 0.061 | 0.08 | 0.03 | 0.042 | 0.042 | 0.022 | 0.033 | 0.05 | 0.062 | 0.052 | 0.057 |
| 65 | 0.084 | 0.06 | 0.061 | 0.03 | 0.033 | 0.06 | 0.038 | 0.061 | 0.039 | 0.061 | 0.062 | 0.027 |
| 66 | 0.1 | 0.052 | 0.05 | 0.04 | 0.06 | 0.07 | 0.033 | 0.052 | 0.05 | 0.067 | 0.042 | 0.05 |
| 67 | 0.04 | 0.02 | 0.03 | 0.05 | 0.071 | 0.052 | 0.044 | 0.034 | 0.022 | 0.062 | 0.05 | 0.05 |
| 68 | 0.071 | 0.03 | 0.02 | 0.07 | 0.03 | 0.03 | 0.038 | 0.022 | 0.049 | 0.058 | 0.054 | 0.072 |
| 69 | 0.033 | 0.05 | 0.033 | 0.07 | 0.057 | 0.06 | 0.022 | 0.033 | 0.039 | 0.064 | 0.06 | 0.05 |
| 70 | 0.06 | 0.03 | 0.03 | 0.024 | 0.057 | 0.06 | 0.034 | 0.033 | 0.039 | 0.062 | 0.031 | 0.044 |
| 71 | 0.07 | 0.04 | 0.033 | 0.05 | 0.05 | 0.06 | 0.039 | 0.041 | 0.052 | 0.05 | 0.051 | 0.028 |
| 72 | 0.05 | 0.03 | 0.05 | 0.052 | 0.042 | 0.06 | 0.022 | 0.013 | 0.044 | 0.036 | 0.054 | 0.027 |
| 73 | 0.061 | 0.05 | 0.05 | 0.03 | 0.052 | 0.042 | 0.027 | 0.044 | 0.027 | 0.041 | 0.06 | 0.066 |
| 74 | 0.071 | 0.05 | 0.04 | 0.071 | 0.052 | 0.05 | 0.027 | 0.034 | 0.058 | 0.053 | 0.051 | 0.062 |
| 75 | 0.091 | 0.04 | 0.03 | 0.04 | 0.05 | 0.07 | 0.023 | 0.023 | 0.039 | 0.039 | 0.03 | 0.04 |
| 76 | 0.091 | 0.03 | 0.048 | 0.024 | 0.04 | 0.05 | 0.028 | 0.028 | 0.034 | 0.066 | 0.05 | 0.046 |
| 77 | 0.091 | 0.03 | 0.04 | 0.033 | 0.071 | 0.071 | 0.039 | 0.028 | 0.034 | 0.039 | 0.048 | 0.028 |
| 78 | 0.05 | 0.04 | 0.071 | 0.08 | 0.03 | 0.07 | 0.06 | 0.027 | 0.054 | 0.04 | 0.009 | 0.041 |
| 79 | 0.071 | 0.04 | 0.04 | 0.05 | 0.091 | 0.05 | 0.041 | 0.034 | 0.049 | 0.039 | 0.011 | 0.033 |
| 80 | 0.061 | 0.05 | 0.05 | 0.061 | 0.04 | 0.06 | 0.034 | 0.054 | 0.038 | 0.066 | 0.017 | 0.052 |
| 81 | 0.04 | 0.04 | 0.05 | 0.04 | 0.052 | 0.052 | 0.035 | 0.033 | 0.018 | 0.055 | 0.044 | 0.053 |
| 82 | 0.071 | 0.05 | 0.04 | 0.048 | 0.05 | 0.05 | 0.028 | 0.05 | 0.044 | 0.062 | 0.05 | 0.06 |
| 83 | 0.03 | 0.05 | 0.091 | 0.05 | 0.045 | 0.02 | 0.022 | 0.038 | 0.04 | 0.034 | 0.051 | 0.062 |
| 84 | 0.04 | 0.033 | 0.03 | 0.04 | 0.073 | 0.073 | 0.06 | 0.052 | 0.013 | 0.044 | 0.06 | 0.044 |
| 85 | 0.04 | 0.07 | 0.05 | 0.05 | 0.05 | 0.024 | 0.038 | 0.033 | 0.064 | 0.033 | 0.056 | 0.05 |
| 86 | 0.05 | 0.03 | 0.01 | 0.05 | 0.042 | 0.042 | 0.053 | 0.022 | 0.013 | 0.028 | 0.057 | 0.049 |
| 87 | 0.1 | 0.04 | 0.057 | 0.06 | 0.03 | 0.084 | 0.034 | 0.023 | 0.062 | 0.011 | 0.065 | 0.044 |
| 88 | 0.04 | 0.061 | 0.045 | 0.052 | 0.042 | 0.042 | 0.039 | 0.022 | 0.044 | 0.057 | 0.06 | 0.065 |
| 89 | 0.05 | 0.06 | 0.03 | 0.033 | 0.06 | 0.03 | 0.022 | 0.033 | 0.028 | 0.062 | 0.061 | 0.061 |
| 90 | 0.05 | 0.03 | 0.03 | 0.03 | 0.02 | 0.07 | 0.038 | 0.045 | 0.066 | 0.057 | 0.064 | 0.05 |
| 91 | 0.03 | 0.042 | 0.05 | 0.05 | 0.05 | 0.033 | 0.044 | 0.035 | 0.033 | 0.049 | 0.051 | 0.057 |
| 92 | 0.066 | 0.05 | 0.066 | 0.04 | 0.03 | 0.03 | 0.034 | 0.06 | 0.038 | 0.064 | 0.064 | 0.053 |
| 93 | 0.066 | 0.042 | 0.061 | 0.03 | 0.06 | 0.05 | 0.033 | 0.028 | 0.049 | 0.046 | 0.063 | 0.052 |
| 94 | 0.052 | 0.033 | 0.07 | 0.02 | 0.03 | 0.05 | 0.06 | 0.054 | 0.06 | 0.08 | 0.065 | 0.028 |
| 95 | 0.04 | 0.03 | 0.03 | 0.08 | 0.04 | 0.03 | 0.045 | 0.085 | 0.075 | 0.016 | 0.065 | 0.044 |
| 96 | 0.04 | 0.03 | 0.02 | 0.04 | 0.033 | 0.017 | 0.059 | 0.023 | 0.027 | 0.038 | 0.057 | 0.033 |
| 97 | 0.042 | 0.04 | 0.05 | 0.03 | 0.03 | 0.04 | 0.049 | 0.055 | 0.045 | 0.045 | 0.06 | 0.055 |
| 98 | 0.061 | 0.024 | 0.03 | 0.05 | 0.06 | 0.071 | 0.062 | 0.023 | 0.045 | 0.044 | 0.065 | 0.057 |
| 99 | 0.042 | 0.05 | 0.04 | 0.028 | 0.05 | 0.04 | 0.049 | 0.011 | 0.041 | 0.049 | 0.03 | 0.059 |
| 100 | 0.042 | 0.033 | 0.061 | 0.04 | 0.07 | 0.07 | 0.05 | 0.038 | 0.039 | 0.06 | 0.04 | 0.044 |
| 101 | 0.05 | 0.05 | 0.04 | 0.05 | 0.075 | 0.017 | 0.066 | 0.033 | 0.044 | 0.045 | 0.041 | 0.055 |
| 102 | 0.02 | 0.03 | 0.05 | 0.04 | 0.042 | 0.05 | 0.041 | 0.044 | 0.027 | 0.066 | 0.04 | 0.06 |
| 103 | 0.06 | 0.07 | 0.042 | 0.057 | 0.05 | 0.042 | 0.059 | 0.049 | 0.045 | 0.052 | 0.057 | 0.055 |
| 104 | 0.04 | 0.042 | 0.048 | 0.04 | 0.02 | 0.02 | 0.083 | 0.028 | 0.027 | 0.066 | 0.055 | 0.065 |
| 105 | 0.04 | 0.05 | 0.04 | 0.033 | 0.052 | 0.052 | 0.071 | 0.039 | 0.052 | 0.022 | 0.06 | 0.06 |
| 106 | 0.052 | 0.02 | 0.05 | 0.061 | 0.03 | 0.042 | 0.033 | 0.066 | 0.045 | 0.027 | 0.053 | 0.033 |
| 107 | 0.04 | 0.02 | 0.03 | 0.03 | 0.04 | 0.02 | 0.073 | 0.039 | 0.061 | 0.04 | 0.06 | 0.078 |
| 108 | 0.04 | 0.03 | 0.066 | 0.05 | 0.052 | 0.052 | 0.066 | 0.068 | 0.044 | 0.06 | 0.05 | 0.033 |
| 109 | 0.04 | 0.04 | 0.05 | 0.052 | 0.061 | 0.05 | 0.035 | 0.055 | 0.055 | 0.044 | 0.055 | 0.067 |
| 110 | 0.075 | 0.066 | 0.024 | 0.04 | 0.05 | 0.048 | 0.041 | 0.044 | 0.071 | 0.053 | 0.065 | 0.044 |
| 111 | 0.075 | 0.052 | 0.02 | 0.04 | 0.042 | 0.09 | 0.038 | 0.034 | 0.039 | 0.041 | 0.057 | 0.049 |
| 112 | 0.075 | 0.071 | 0.05 | 0.03 | 0.04 | 0.061 | 0.06 | 0.038 | 0.05 | 0.022 | 0.04 | 0.044 |
| 113 | 0.052 | 0.09 | 0.066 | 0.05 | 0.06 | 0.05 | 0.05 | 0.039 | 0.066 | 0.04 | 0.06 | 0.067 |
| 114 | 0.061 | 0.061 | 0.052 | 0.03 | 0.03 | 0.084 | 0.055 | 0.044 | 0.033 | 0.039 | 0.052 | 0.028 |
| 115 | 0.05 | 0.07 | 0.061 | 0.061 | 0.042 | 0.081 | 0.096 | 0.049 | 0.027 | 0.065 | 0.04 | 0.073 |
| 116 | 0.052 | 0.052 | 0.05 | 0.064 | 0.071 | 0.061 | 0.045 | 0.038 | 0.033 | 0.06 | 0.04 | 0.055 |
| 117 | 0.04 | 0.06 | 0.07 | 0.05 | 0.04 | 0.05 | 0.066 | 0.033 | 0.066 | 0.052 | 0.03 | 0.094 |
| 118 | 0.042 | 0.05 | 0.02 | 0.048 | 0.061 | 0.06 | 0.044 | 0.055 | 0.055 | 0.023 | 0.046 | 0.066 |
| 119 | 0.1 | 0.033 | 0.02 | 0.033 | 0.03 | 0.017 | 0.038 | 0.044 | 0.038 | 0.055 | 0.05 | 0.034 |
| 120 | 0.066 | 0.05 | 0.071 | 0.06 | 0.048 | 0.075 | 0.044 | 0.046 | 0.027 | 0.049 | 0.052 | 0.046 |
| 121 | 0.07 | 0.05 | 0.03 | 0.04 | 0.061 | 0.05 | 0.05 | 0.033 | 0.027 | 0.049 | 0.057 | 0.06 |
| 122 | 0.071 | 0.05 | 0.03 | 0.061 | 0.04 | 0.05 | 0.05 | 0.016 | 0.022 | 0.049 | 0.055 | 0.049 |
| 123 | 0.07 | 0.066 | 0.04 | 0.042 | 0.052 | 0.048 | 0.049 | 0.039 | 0.072 | 0.058 | 0.04 | 0.066 |
| 124 | 0.061 | 0.06 | 0.052 | 0.042 | 0.061 | 0.052 | 0.022 | 0.027 | 0.046 | 0.033 | 0.06 | 0.06 |
| 125 | 0.057 | 0.04 | 0.071 | 0.04 | 0.01 | 0.03 | 0.083 | 0.033 | 0.055 | 0.046 | 0.06 | 0.06 |
| 126 | 0.07 | 0.033 | 0.04 | 0.066 | 0.091 | 0.05 | 0.049 | 0.034 | 0.027 | 0.062 | 0.051 | 0.06 |
| 127 | 0.07 | 0.06 | 0.03 | 0.05 | 0.03 | 0.05 | 0.049 | 0.044 | 0.052 | 0.036 | 0.05 | 0.061 |
| 128 | 0.08 | 0.064 | 0.052 | 0.042 | 0.048 | 0.04 | 0.044 | 0.016 | 0.026 | 0.025 | 0.052 | 0.055 |
| 129 | 0.052 | 0.071 | 0.04 | 0.042 | 0.052 | 0.033 | 0.06 | 0.072 | 0.016 | 0.045 | 0.06 | 0.055 |
| 130 | 0.052 | 0.04 | 0.042 | 0.03 | 0.05 | 0.1 | 0.082 | 0.055 | 0.062 | 0.026 | 0.055 | 0.068 |
| 131 | 0.06 | 0.075 | 0.03 | 0.07 | 0.05 | 0.05 | 0.044 | 0.027 | 0.046 | 0.059 | 0.061 | 0.066 |
| 132 | 0.06 | 0.033 | 0.05 | 0.07 | 0.073 | 0.02 | 0.06 | 0.044 | 0.049 | 0.057 | 0.06 | 0.049 |
| 133 | 0.05 | 0.03 | 0.033 | 0.04 | 0.05 | 0.03 | 0.049 | 0.027 | 0.05 | 0.031 | 0.056 | 0.038 |
| 134 | 0.075 | 0.04 | 0.061 | 0.048 | 0.048 | 0.091 | 0.039 | 0.038 | 0.034 | 0.055 | 0.06 | 0.061 |
| 135 | 0.06 | 0.033 | 0.061 | 0.052 | 0.057 | 0.05 | 0.038 | 0.046 | 0.033 | 0.044 | 0.051 | 0.062 |
| 136 | 0.06 | 0.05 | 0.052 | 0.033 | 0.07 | 0.052 | 0.041 | 0.027 | 0.077 | 0.062 | 0.05 | 0.06 |
| 137 | 0.052 | 0.04 | 0.04 | 0.06 | 0.03 | 0.048 | 0.036 | 0.027 | 0.016 | 0.04 | 0.06 | 0.055 |
| 138 | 0.101 | 0.04 | 0.07 | 0.03 | 0.02 | 0.052 | 0.033 | 0.033 | 0.038 | 0.049 | 0.061 | 0.044 |
| 139 | 0.048 | 0.071 | 0.08 | 0.04 | 0.04 | 0.03 | 0.045 | 0.072 | 0.034 | 0.06 | 0.042 | 0.038 |
| 140 | 0.06 | 0.02 | 0.05 | 0.052 | 0.081 | 0.07 | 0.055 | 0.033 | 0.028 | 0.064 | 0.06 | 0.044 |
| 141 | 0.05 | 0.052 | 0.02 | 0.07 | 0.061 | 0.05 | 0.027 | 0.066 | 0.039 | 0.039 | 0.061 | 0.039 |
| 142 | 0.066 | 0.071 | 0.048 | 0.061 | 0.07 | 0.05 | 0.057 | 0.049 | 0.05 | 0.039 | 0.06 | 0.034 |
| 143 | 0.061 | 0.05 | 0.052 | 0.07 | 0.064 | 0.033 | 0.058 | 0.059 | 0.034 | 0.06 | 0.061 | 0.062 |
| 144 | 0.07 | 0.04 | 0.07 | 0.052 | 0.05 | 0.042 | 0.061 | 0.022 | 0.028 | 0.049 | 0.053 | 0.055 |
| 145 | 0.061 | 0.03 | 0.06 | 0.03 | 0.06 | 0.04 | 0.046 | 0.033 | 0.027 | 0.055 | 0.06 | 0.036 |
| 146 | 0.03 | 0.04 | 0.02 | 0.06 | 0.052 | 0.033 | 0.041 | 0.044 | 0.033 | 0.055 | 0.06 | 0.057 |
| 147 | 0.04 | 0.03 | 0.03 | 0.06 | 0.042 | 0.03 | 0.027 | 0.022 | 0.027 | 0.06 | 0.064 | 0.065 |
| 148 | 0.072 | 0.042 | 0.03 | 0.042 | 0.081 | 0.02 | 0.034 | 0.028 | 0.061 | 0.049 | 0.063 | 0.039 |
| 149 | 0.071 | 0.03 | 0.05 | 0.05 | 0.04 | 0.05 | 0.01 | 0.033 | 0.034 | 0.054 | 0.06 | 0.038 |
| 150 | 0.075 | 0.03 | 0.04 | 0.04 | 0.048 | 0.052 | 0.037 | 0.038 | 0.027 | 0.062 | 0.05 | 0.057 |
| 151 | 0.052 | 0.042 | 0.05 | 0.081 | 0.096 | 0.033 | 0.037 | 0.058 | 0.049 | 0.028 | 0.065 | 0.072 |
| 152 | 0.052 | 0.02 | 0.04 | 0.05 | 0.052 | 0.071 | 0.037 | 0.022 | 0.033 | 0.054 | 0.06 | 0.082 |
| 153 | 0.03 | 0.03 | 0.052 | 0.024 | 0.04 | 0.052 | 0.037 | 0.031 | 0.046 | 0.034 | 0.065 | 0.053 |
| 154 | 0.04 | 0.03 | 0.042 | 0.024 | 0.042 | 0.04 | 0.037 | 0.049 | 0.031 | 0.041 | 0.061 | 0.053 |
| 155 | 0.048 | 0.04 | 0.042 | 0.03 | 0.06 | 0.042 | 0.044 | 0.033 | 0.036 | 0.046 | 0.062 | 0.052 |
| 156 | 0.04 | 0.07 | 0.04 | 0.02 | 0.03 | 0.05 | 0.044 | 0.028 | 0.016 | 0.036 | 0.063 | 0.061 |
| 157 | 0.061 | 0.02 | 0.06 | 0.02 | 0.04 | 0.066 | 0.052 | 0.05 | 0.062 | 0.049 | 0.058 | 0.066 |
| 158 | 0.052 | 0.061 | 0.03 | 0.03 | 0.02 | 0.06 | 0.059 | 0.028 | 0.027 | 0.061 | 0.062 | 0.065 |
| 159 | 0.052 | 0.04 | 0.06 | 0.04 | 0.052 | 0.04 | 0.059 | 0.027 | 0.049 | 0.064 | 0.065 | 0.033 |
| 160 | 0.057 | 0.05 | 0.06 | 0.061 | 0.04 | 0.02 | 0.066 | 0.018 | 0.049 | 0.061 | 0.05 | 0.027 |
| 161 | 0.042 | 0.061 | 0.04 | 0.04 | 0.052 | 0.024 | 0.066 | 0.022 | 0.04 | 0.033 | 0.051 | 0.061 |
| 162 | 0.075 | 0.04 | 0.052 | 0.066 | 0.05 | 0.052 | 0.066 | 0.057 | 0.051 | 0.052 | 0.033 | 0.05 |
| 163 | 0.05 | 0.081 | 0.01 | 0.052 | 0.05 | 0.04 | 0.067 | 0.033 | 0.027 | 0.055 | 0.04 | 0.051 |
| 164 | 0.042 | 0.033 | 0.03 | 0.05 | 0.04 | 0.04 | 0.067 | 0.016 | 0.028 | 0.055 | 0.066 | 0.052 |
| 165 | 0.06 | 0.05 | 0.061 | 0.033 | 0.052 | 0.033 | 0.07 | 0.039 | 0.061 | 0.055 | 0.06 | 0.054 |
| 166 | 0.101 | 0.04 | 0.03 | 0.06 | 0.081 | 0.08 | 0.027 | 0.046 | 0.052 | 0.044 | 0.065 | 0.045 |
| 167 | 0.071 | 0.052 | 0.03 | 0.052 | 0.028 | 0.04 | 0.044 | 0.045 | 0.062 | 0.05 | 0.066 | 0.052 |
| 168 | 0.06 | 0.03 | 0.03 | 0.04 | 0.024 | 0.07 | 0.038 | 0.044 | 0.045 | 0.044 | 0.059 | 0.06 |
| 169 | 0.04 | 0.061 | 0.02 | 0.06 | 0.042 | 0.066 | 0.066 | 0.034 | 0.033 | 0.062 | 0.053 | 0.044 |
| 170 | 0.04 | 0.08 | 0.06 | 0.04 | 0.06 | 0.04 | 0.054 | 0.034 | 0.044 | 0.066 | 0.06 | 0.038 |
| 171 | 0.06 | 0.033 | 0.042 | 0.05 | 0.06 | 0.03 | 0.027 | 0.045 | 0.022 | 0.048 | 0.04 | 0.022 |
| 172 | 0.081 | 0.05 | 0.03 | 0.08 | 0.05 | 0.04 | 0.057 | 0.022 | 0.027 | 0.035 | 0.04 | 0.022 |
| 173 | 0.081 | 0.052 | 0.03 | 0.1 | 0.033 | 0.02 | 0.049 | 0.038 | 0.038 | 0.016 | 0.055 | 0.033 |
| 174 | 0.061 | 0.048 | 0.03 | 0.042 | 0.042 | 0.033 | 0.045 | 0.036 | 0.044 | 0.055 | 0.061 | 0.044 |
| 175 | 0.04 | 0.071 | 0.03 | 0.03 | 0.02 | 0.07 | 0.045 | 0.054 | 0.022 | 0.033 | 0.06 | 0.066 |
| 176 | 0.04 | 0.04 | 0.042 | 0.061 | 0.05 | 0.042 | 0.066 | 0.038 | 0.028 | 0.055 | 0.054 | 0.035 |
| 177 | 0.081 | 0.042 | 0.052 | 0.024 | 0.04 | 0.04 | 0.033 | 0.028 | 0.016 | 0.022 | 0.06 | 0.034 |
| 178 | 0.061 | 0.03 | 0.061 | 0.042 | 0.05 | 0.06 | 0.044 | 0.028 | 0.034 | 0.026 | 0.061 | 0.028 |
| 179 | 0.071 | 0.03 | 0.057 | 0.05 | 0.03 | 0.06 | 0.049 | 0.055 | 0.078 | 0.052 | 0.062 | 0.041 |
| 180 | 0.07 | 0.05 | 0.04 | 0.03 | 0.033 | 0.07 | 0.046 | 0.022 | 0.036 | 0.06 | 0.05 | 0.048 |
| 181 | 0.05 | 0.04 | 0.04 | 0.04 | 0.03 | 0.081 | 0.055 | 0.027 | 0.05 | 0.063 | 0.064 | 0.05 |
| 182 | 0.03 | 0.052 | 0.05 | 0.03 | 0.061 | 0.033 | 0.055 | 0.016 | 0.031 | 0.06 | 0.064 | 0.05 |
| 183 | 0.05 | 0.042 | 0.03 | 0.05 | 0.042 | 0.04 | 0.038 | 0.016 | 0.039 | 0.06 | 0.057 | 0.044 |
| 184 | 0.052 | 0.07 | 0.05 | 0.04 | 0.042 | 0.061 | 0.038 | 0.034 | 0.044 | 0.033 | 0.056 | 0.061 |
| 185 | 0.03 | 0.03 | 0.02 | 0.02 | 0.033 | 0.066 | 0.049 | 0.016 | 0.04 | 0.063 | 0.05 | 0.044 |
| 186 | 0.033 | 0.05 | 0.042 | 0.024 | 0.052 | 0.052 | 0.044 | 0.039 | 0.018 | 0.061 | 0.06 | 0.066 |
| 187 | 0.04 | 0.052 | 0.03 | 0.024 | 0.04 | 0.061 | 0.033 | 0.068 | 0.016 | 0.062 | 0.061 | 0.068 |
| 188 | 0.075 | 0.08 | 0.04 | 0.04 | 0.04 | 0.06 | 0.05 | 0.038 | 0.033 | 0.058 | 0.061 | 0.061 |
| 189 | 0.052 | 0.06 | 0.024 | 0.033 | 0.057 | 0.03 | 0.07 | 0.022 | 0.044 | 0.064 | 0.057 | 0.055 |
| 190 | 0.05 | 0.04 | 0.03 | 0.04 | 0.04 | 0.04 | 0.036 | 0.023 | 0.039 | 0.05 | 0.05 | 0.061 |
| 191 | 0.061 | 0.09 | 0.042 | 0.052 | 0.042 | 0.024 | 0.041 | 0.016 | 0.025 | 0.048 | 0.061 | 0.061 |
| 192 | 0.061 | 0.06 | 0.033 | 0.03 | 0.042 | 0.066 | 0.031 | 0.049 | 0.027 | 0.062 | 0.06 | 0.055 |
| 193 | 0.052 | 0.052 | 0.042 | 0.04 | 0.05 | 0.04 | 0.055 | 0.041 | 0.043 | 0.061 | 0.065 | 0.071 |
| 194 | 0.052 | 0.042 | 0.04 | 0.024 | 0.042 | 0.061 | 0.046 | 0.033 | 0.035 | 0.052 | 0.064 | 0.068 |
| 195 | 0.05 | 0.04 | 0.033 | 0.042 | 0.052 | 0.04 | 0.038 | 0.05 | 0.035 | 0.062 | 0.061 | 0.044 |
| 196 | 0.072 | 0.05 | 0.052 | 0.057 | 0.061 | 0.042 | 0.033 | 0.072 | 0.059 | 0.046 | 0.062 | 0.055 |
| 197 | 0.01 | 0.061 | 0.05 | 0.02 | 0.052 | 0.033 | 0.066 | 0.057 | 0.044 | 0.059 | 0.065 | 0.053 |
| 198 | 0.071 | 0.05 | 0.05 | 0.033 | 0.052 | 0.06 | 0.052 | 0.022 | 0.031 | 0.065 | 0.064 | 0.055 |
| 199 | 0.04 | 0.052 | 0.042 | 0.024 | 0.04 | 0.052 | 0.044 | 0.022 | 0.039 | 0.05 | 0.05 | 0.058 |
| 200 | 0.02 | 0.042 | 0.06 | 0.057 | 0.02 | 0.05 | 0.044 | 0.016 | 0.061 | 0.037 | 0.053 | 0.06 |
| 201 | 0.042 | 0.04 | 0.061 | 0.033 | 0.06 | 0.057 | 0.033 | 0.018 | 0.025 | 0.049 | 0.058 | 0.061 |
| 202 | 0.061 | 0.075 | 0.06 | 0.048 | 0.03 | 0.033 | 0.027 | 0.05 | 0.044 | 0.055 | 0.06 | 0.037 |
| 203 | 0.057 | 0.081 | 0.052 | 0.042 | 0.08 | 0.052 | 0.044 | 0.044 | 0.066 | 0.045 | 0.054 | 0.044 |
| 204 | 0.06 | 0.05 | 0.07 | 0.02 | 0.05 | 0.052 | 0.06 | 0.016 | 0.045 | 0.028 | 0.06 | 0.06 |
| 205 | 0.05 | 0.075 | 0.071 | 0.03 | 0.06 | 0.05 | 0.048 | 0.022 | 0.044 | 0.049 | 0.055 | 0.053 |
| 206 | 0.02 | 0.033 | 0.052 | 0.02 | 0.04 | 0.05 | 0.038 | 0.057 | 0.044 | 0.038 | 0.06 | 0.049 |
| 207 | 0.05 | 0.042 | 0.04 | 0.02 | 0.042 | 0.061 | 0.038 | 0.038 | 0.044 | 0.049 | 0.061 | 0.052 |
| 208 | 0.052 | 0.06 | 0.061 | 0.02 | 0.02 | 0.052 | 0.058 | 0.038 | 0.022 | 0.061 | 0.066 | 0.022 |
| 209 | 0.06 | 0.07 | 0.05 | 0.05 | 0.048 | 0.05 | 0.033 | 0.016 | 0.027 | 0.065 | 0.057 | 0.034 |
| 210 | 0.084 | 0.052 | 0.033 | 0.03 | 0.06 | 0.05 | 0.023 | 0.027 | 0.028 | 0.061 | 0.058 | 0.038 |
| 211 | 0.03 | 0.052 | 0.048 | 0.052 | 0.024 | 0.072 | 0.033 | 0.007 | 0.034 | 0.062 | 0.051 | 0.031 |
| 212 | 0.052 | 0.03 | 0.08 | 0.033 | 0.057 | 0.03 | 0.071 | 0.013 | 0.044 | 0.038 | 0.06 | 0.04 |
| 213 | 0.057 | 0.033 | 0.072 | 0.03 | 0.048 | 0.033 | 0.049 | 0.075 | 0.036 | 0.028 | 0.051 | 0.062 |
| 214 | 0.05 | 0.04 | 0.06 | 0.05 | 0.057 | 0.06 | 0.044 | 0.039 | 0.033 | 0.044 | 0.052 | 0.057 |
| 215 | 0.048 | 0.033 | 0.03 | 0.04 | 0.071 | 0.033 | 0.031 | 0.022 | 0.05 | 0.028 | 0.057 | 0.062 |
| 216 | 0.03 | 0.06 | 0.061 | 0.04 | 0.06 | 0.05 | 0.038 | 0.011 | 0.05 | 0.055 | 0.051 | 0.061 |
| 217 | 0.06 | 0.061 | 0.03 | 0.057 | 0.06 | 0.096 | 0.023 | 0.022 | 0.05 | 0.059 | 0.05 | 0.049 |
| 218 | 0.024 | 0.06 | 0.04 | 0.042 | 0.075 | 0.064 | 0.033 | 0.033 | 0.077 | 0.044 | 0.041 | 0.06 |
| 219 | 0.05 | 0.05 | 0.05 | 0.066 | 0.04 | 0.02 | 0.045 | 0.011 | 0.062 | 0.023 | 0.045 | 0.061 |
| 220 | 0.066 | 0.066 | 0.06 | 0.05 | 0.094 | 0.04 | 0.044 | 0.06 | 0.022 | 0.036 | 0.043 | 0.06 |
| 221 | 0.052 | 0.042 | 0.064 | 0.033 | 0.04 | 0.04 | 0.045 | 0.06 | 0.072 | 0.022 | 0.049 | 0.062 |
| 222 | 0.05 | 0.04 | 0.04 | 0.048 | 0.03 | 0.052 | 0.033 | 0.044 | 0.062 | 0.01 | 0.052 | 0.062 |
| 223 | 0.08 | 0.071 | 0.03 | 0.042 | 0.07 | 0.04 | 0.066 | 0.038 | 0.035 | 0.063 | 0.06 | 0.06 |
| 224 | 0.07 | 0.05 | 0.02 | 0.02 | 0.04 | 0.05 | 0.033 | 0.027 | 0.054 | 0.055 | 0.061 | 0.055 |
| 225 | 0.052 | 0.05 | 0.06 | 0.04 | 0.04 | 0.06 | 0.026 | 0.016 | 0.033 | 0.058 | 0.06 | 0.055 |
| 226 | 0.05 | 0.05 | 0.042 | 0.067 | 0.033 | 0.04 | 0.027 | 0.055 | 0.055 | 0.061 | 0.05 | 0.061 |
| 227 | 0.07 | 0.04 | 0.06 | 0.04 | 0.033 | 0.08 | 0.038 | 0.016 | 0.071 | 0.06 | 0.051 | 0.071 |
| 228 | 0.03 | 0.061 | 0.057 | 0.03 | 0.042 | 0.052 | 0.033 | 0.028 | 0.035 | 0.049 | 0.054 | 0.07 |
| 229 | 0.042 | 0.081 | 0.042 | 0.071 | 0.04 | 0.071 | 0.033 | 0.027 | 0.039 | 0.066 | 0.053 | 0.048 |
| 230 | 0.033 | 0.08 | 0.05 | 0.04 | 0.07 | 0.057 | 0.038 | 0.033 | 0.055 | 0.055 | 0.06 | 0.022 |
| 231 | 0.05 | 0.033 | 0.061 | 0.05 | 0.042 | 0.061 | 0.066 | 0.028 | 0.061 | 0.055 | 0.053 | 0.055 |
| 232 | 0.01 | 0.048 | 0.02 | 0.07 | 0.071 | 0.06 | 0.023 | 0.055 | 0.033 | 0.062 | 0.055 | 0.027 |
| 233 | 0.052 | 0.071 | 0.04 | 0.024 | 0.033 | 0.07 | 0.028 | 0.049 | 0.044 | 0.067 | 0.054 | 0.011 |
| 234 | 0.06 | 0.04 | 0.04 | 0.06 | 0.057 | 0.057 | 0.033 | 0.045 | 0.031 | 0.062 | 0.052 | 0.027 |
| 235 | 0.03 | 0.02 | 0.057 | 0.033 | 0.05 | 0.06 | 0.045 | 0.027 | 0.041 | 0.056 | 0.051 | 0.057 |
| 236 | 0.052 | 0.05 | 0.081 | 0.06 | 0.03 | 0.05 | 0.044 | 0.05 | 0.016 | 0.061 | 0.05 | 0.022 |
| 237 | 0.061 | 0.061 | 0.052 | 0.052 | 0.052 | 0.07 | 0.026 | 0.06 | 0.035 | 0.055 | 0.04 | 0.039 |
| 238 | 0.042 | 0.06 | 0.01 | 0.03 | 0.033 | 0.02 | 0.027 | 0.066 | 0.055 | 0.045 | 0.04 | 0.044 |
| 239 | 0.061 | 0.033 | 0.04 | 0.03 | 0.042 | 0.061 | 0.027 | 0.038 | 0.044 | 0.073 | 0.055 | 0.033 |
| 240 | 0.081 | 0.05 | 0.052 | 0.06 | 0.03 | 0.04 | 0.034 | 0.033 | 0.055 | 0.055 | 0.059 | 0.071 |
| 241 | 0.02 | 0.04 | 0.042 | 0.06 | 0.061 | 0.042 | 0.041 | 0.044 | 0.034 | 0.069 | 0.05 | 0.07 |
| 242 | 0.01 | 0.06 | 0.066 | 0.03 | 0.03 | 0.033 | 0.033 | 0.023 | 0.035 | 0.09 | 0.055 | 0.07 |
| 243 | 0.052 | 0.07 | 0.048 | 0.04 | 0.033 | 0.04 | 0.053 | 0.011 | 0.055 | 0.061 | 0.045 | 0.055 |
| 244 | 0.03 | 0.04 | 0.06 | 0.05 | 0.071 | 0.024 | 0.027 | 0.026 | 0.06 | 0.04 | 0.04 | 0.035 |
| 245 | 0.052 | 0.05 | 0.03 | 0.052 | 0.03 | 0.03 | 0.034 | 0.039 | 0.045 | 0.046 | 0.049 | 0.066 |
| 246 | 0.07 | 0.042 | 0.067 | 0.04 | 0.033 | 0.02 | 0.011 | 0.023 | 0.041 | 0.039 | 0.04 | 0.06 |
| 247 | 0.084 | 0.03 | 0.042 | 0.03 | 0.061 | 0.05 | 0.022 | 0.055 | 0.038 | 0.061 | 0.03 | 0.062 |
| 248 | 0.04 | 0.033 | 0.057 | 0.061 | 0.01 | 0.07 | 0.039 | 0.044 | 0.039 | 0.04 | 0.042 | 0.058 |
| 249 | 0.08 | 0.052 | 0.042 | 0.03 | 0.06 | 0.064 | 0.018 | 0.05 | 0.035 | 0.044 | 0.04 | 0.05 |
| 250 | 0.061 | 0.061 | 0.033 | 0.052 | 0.03 | 0.024 | 0.049 | 0.052 | 0.031 | 0.098 | 0.031 | 0.058 |
| Mean | 0.054944 | 0.046432 | 0.048164 | 0.044836 | 0.046288 | 0.049404 | 0.043752 | 0.037676 | 0.041152 | 0.04956 | 0.054264 | 0.049892 |
| SD | 0.018334 | 0.0155 | 0.016504 | 0.01544 | 0.015385 | 0.016533 | 0.01461 | 0.014888 | 0.014022 | 0.013575 | 0.009137 | 0.01457 |

|  | Day 2 | | | | | | Day 3 | | | | | |
| --- | --- | --- | --- | --- | --- | --- | --- | --- | --- | --- | --- | --- |
| OCT images | Control | | | Infected | | | Control | | | Infected | | |
|  | 1 | 2 | 3 | 1 | 2 | 3 | 1 | 2 | 3 | 1 | 2 | 3 |
| 1 | 0.044 | 0.046 | 0.05 | 0.066 | 0.04 | 0.06 | 0.02 | 0.033 | 0.04 | 0.033 | 0.091 | 0.072 |
| 2 | 0.033 | 0.059 | 0.011 | 0.071 | 0.071 | 0.105 | 0.04 | 0.07 | 0.05 | 0.061 | 0.06 | 0.071 |
| 3 | 0.055 | 0.033 | 0.066 | 0.052 | 0.081 | 0.07 | 0.05 | 0.03 | 0.024 | 0.071 | 0.033 | 0.061 |
| 4 | 0.016 | 0.033 | 0.039 | 0.03 | 0.02 | 0.04 | 0.033 | 0.04 | 0.05 | 0.07 | 0.103 | 0.06 |
| 5 | 0.011 | 0.016 | 0.028 | 0.042 | 0.101 | 0.05 | 0.05 | 0.03 | 0.04 | 0.05 | 0.071 | 0.052 |
| 6 | 0.039 | 0.041 | 0.057 | 0.052 | 0.066 | 0.12 | 0.052 | 0.04 | 0.03 | 0.071 | 0.052 | 0.052 |
| 7 | 0.038 | 0.052 | 0.022 | 0.066 | 0.048 | 0.02 | 0.07 | 0.042 | 0.04 | 0.08 | 0.05 | 0.071 |
| 8 | 0.041 | 0.038 | 0.044 | 0.02 | 0.052 | 0.024 | 0.071 | 0.03 | 0.04 | 0.091 | 0.042 | 0.071 |
| 9 | 0.045 | 0.049 | 0.031 | 0.06 | 0.042 | 0.061 | 0.061 | 0.01 | 0.04 | 0.1 | 0.06 | 0.07 |
| 10 | 0.038 | 0.023 | 0.039 | 0.03 | 0.04 | 0.05 | 0.04 | 0.01 | 0.05 | 0.09 | 0.071 | 0.1 |
| 11 | 0.034 | 0.055 | 0.022 | 0.081 | 0.042 | 0.033 | 0.06 | 0.024 | 0.061 | 0.05 | 0.08 | 0.113 |
| 12 | 0.044 | 0.027 | 0.044 | 0.03 | 0.03 | 0.101 | 0.061 | 0.045 | 0.07 | 0.06 | 0.06 | 0.042 |
| 13 | 0.045 | 0.022 | 0.036 | 0.01 | 0.061 | 0.061 | 0.05 | 0.03 | 0.01 | 0.04 | 0.081 | 0.131 |
| 14 | 0.045 | 0.041 | 0.055 | 0.017 | 0.03 | 0.071 | 0.061 | 0.04 | 0.042 | 0.04 | 0.12 | 0.081 |
| 15 | 0.038 | 0.027 | 0.044 | 0.03 | 0.04 | 0.06 | 0.052 | 0.02 | 0.03 | 0.04 | 0.111 | 0.081 |
| 16 | 0.028 | 0.028 | 0.023 | 0.042 | 0.042 | 0.05 | 0.04 | 0.03 | 0.04 | 0.04 | 0.05 | 0.113 |
| 17 | 0.038 | 0.036 | 0.038 | 0.03 | 0.06 | 0.042 | 0.042 | 0.033 | 0.064 | 0.061 | 0.042 | 0.08 |
| 18 | 0.027 | 0.045 | 0.034 | 0.052 | 0.04 | 0.071 | 0.04 | 0.02 | 0.057 | 0.05 | 0.05 | 0.094 |
| 19 | 0.028 | 0.044 | 0.016 | 0.04 | 0.03 | 0.05 | 0.024 | 0.024 | 0.04 | 0.02 | 0.057 | 0.06 |
| 20 | 0.049 | 0.033 | 0.027 | 0.07 | 0.013 | 0.071 | 0.04 | 0.042 | 0.042 | 0.05 | 0.081 | 0.071 |
| 21 | 0.071 | 0.044 | 0.034 | 0.04 | 0.033 | 0.06 | 0.04 | 0.02 | 0.042 | 0.057 | 0.07 | 0.057 |
| 22 | 0.044 | 0.062 | 0.072 | 0.072 | 0.071 | 0.06 | 0.03 | 0.02 | 0.05 | 0.06 | 0.042 | 0.05 |
| 23 | 0.049 | 0.046 | 0.033 | 0.07 | 0.02 | 0.07 | 0.061 | 0.04 | 0.033 | 0.09 | 0.12 | 0.071 |
| 24 | 0.033 | 0.055 | 0.038 | 0.108 | 0.04 | 0.05 | 0.042 | 0.03 | 0.05 | 0.101 | 0.136 | 0.052 |
| 25 | 0.039 | 0.055 | 0.068 | 0.073 | 0.066 | 0.06 | 0.05 | 0.04 | 0.04 | 0.07 | 0.126 | 0.06 |
| 26 | 0.025 | 0.022 | 0.016 | 0.07 | 0.052 | 0.07 | 0.052 | 0.03 | 0.057 | 0.052 | 0.06 | 0.111 |
| 27 | 0.062 | 0.06 | 0.054 | 0.07 | 0.04 | 0.048 | 0.066 | 0.02 | 0.033 | 0.04 | 0.09 | 0.042 |
| 28 | 0.066 | 0.049 | 0.023 | 0.04 | 0.071 | 0.05 | 0.052 | 0.03 | 0.04 | 0.066 | 0.071 | 0.07 |
| 29 | 0.044 | 0.04 | 0.033 | 0.04 | 0.07 | 0.05 | 0.02 | 0.033 | 0.048 | 0.052 | 0.081 | 0.05 |
| 30 | 0.06 | 0.028 | 0.045 | 0.017 | 0.06 | 0.071 | 0.01 | 0.048 | 0.024 | 0.05 | 0.094 | 0.091 |
| 31 | 0.016 | 0.064 | 0.022 | 0.08 | 0.091 | 0.081 | 0.061 | 0.04 | 0.04 | 0.06 | 0.111 | 0.09 |
| 32 | 0.055 | 0.038 | 0.049 | 0.033 | 0.091 | 0.08 | 0.05 | 0.042 | 0.03 | 0.08 | 0.05 | 0.091 |
| 33 | 0.038 | 0.022 | 0.061 | 0.052 | 0.07 | 0.06 | 0.071 | 0.03 | 0.048 | 0.042 | 0.06 | 0.05 |
| 34 | 0.033 | 0.022 | 0.028 | 0.07 | 0.071 | 0.03 | 0.05 | 0.033 | 0.052 | 0.024 | 0.091 | 0.09 |
| 35 | 0.027 | 0.023 | 0.039 | 0.101 | 0.081 | 0.06 | 0.02 | 0.033 | 0.03 | 0.04 | 0.06 | 0.08 |
| 36 | 0.04 | 0.05 | 0.039 | 0.04 | 0.052 | 0.05 | 0.04 | 0.05 | 0.033 | 0.033 | 0.081 | 0.052 |
| 37 | 0.055 | 0.026 | 0.064 | 0.05 | 0.07 | 0.05 | 0.04 | 0.042 | 0.02 | 0.05 | 0.071 | 0.05 |
| 38 | 0.055 | 0.027 | 0.055 | 0.07 | 0.05 | 0.08 | 0.042 | 0.04 | 0.08 | 0.01 | 0.061 | 0.081 |
| 39 | 0.044 | 0.075 | 0.062 | 0.02 | 0.071 | 0.07 | 0.03 | 0.04 | 0.02 | 0.061 | 0.066 | 0.098 |
| 40 | 0.033 | 0.031 | 0.022 | 0.06 | 0.042 | 0.042 | 0.04 | 0.03 | 0.042 | 0.08 | 0.048 | 0.09 |
| 41 | 0.036 | 0.038 | 0.049 | 0.12 | 0.042 | 0.042 | 0.03 | 0.03 | 0.05 | 0.1 | 0.121 | 0.06 |
| 42 | 0.034 | 0.046 | 0.034 | 0.09 | 0.04 | 0.04 | 0.04 | 0.013 | 0.04 | 0.13 | 0.1 | 0.08 |
| 43 | 0.016 | 0.027 | 0.06 | 0.042 | 0.04 | 0.061 | 0.01 | 0.033 | 0.05 | 0.1 | 0.08 | 0.08 |
| 44 | 0.034 | 0.057 | 0.038 | 0.04 | 0.03 | 0.072 | 0.042 | 0.061 | 0.04 | 0.08 | 0.052 | 0.061 |
| 45 | 0.027 | 0.027 | 0.06 | 0.061 | 0.05 | 0.071 | 0.02 | 0.052 | 0.03 | 0.091 | 0.08 | 0.06 |
| 46 | 0.039 | 0.038 | 0.049 | 0.071 | 0.06 | 0.096 | 0.02 | 0.02 | 0.05 | 0.05 | 0.057 | 0.071 |
| 47 | 0.028 | 0.059 | 0.052 | 0.033 | 0.042 | 0.066 | 0.02 | 0.02 | 0.04 | 0.07 | 0.07 | 0.081 |
| 48 | 0.075 | 0.066 | 0.045 | 0.042 | 0.04 | 0.042 | 0.04 | 0.042 | 0.061 | 0.05 | 0.09 | 0.072 |
| 49 | 0.023 | 0.062 | 0.06 | 0.081 | 0.052 | 0.05 | 0.04 | 0.02 | 0.04 | 0.03 | 0.06 | 0.057 |
| 50 | 0.016 | 0.022 | 0.034 | 0.081 | 0.061 | 0.07 | 0.052 | 0.024 | 0.04 | 0.05 | 0.05 | 0.061 |
| 51 | 0.027 | 0.061 | 0.062 | 0.024 | 0.05 | 0.091 | 0.05 | 0.03 | 0.04 | 0.03 | 0.061 | 0.042 |
| 52 | 0.059 | 0.078 | 0.036 | 0.03 | 0.09 | 0.075 | 0.03 | 0.042 | 0.033 | 0.042 | 0.061 | 0.111 |
| 53 | 0.018 | 0.06 | 0.049 | 0.03 | 0.084 | 0.091 | 0.04 | 0.04 | 0.06 | 0.06 | 0.06 | 0.1 |
| 54 | 0.022 | 0.028 | 0.034 | 0.03 | 0.052 | 0.071 | 0.05 | 0.04 | 0.04 | 0.061 | 0.101 | 0.06 |
| 55 | 0.033 | 0.059 | 0.038 | 0.061 | 0.091 | 0.057 | 0.03 | 0.042 | 0.03 | 0.072 | 0.071 | 0.07 |
| 56 | 0.011 | 0.038 | 0.06 | 0.061 | 0.07 | 0.03 | 0.04 | 0.071 | 0.042 | 0.05 | 0.06 | 0.07 |
| 57 | 0.038 | 0.039 | 0.013 | 0.04 | 0.061 | 0.04 | 0.01 | 0.05 | 0.06 | 0.033 | 0.061 | 0.09 |
| 58 | 0.044 | 0.016 | 0.066 | 0.03 | 0.03 | 0.052 | 0.04 | 0.052 | 0.04 | 0.09 | 0.06 | 0.06 |
| 59 | 0.016 | 0.034 | 0.018 | 0.024 | 0.066 | 0.11 | 0.03 | 0.04 | 0 | 0.061 | 0.05 | 0.04 |
| 60 | 0.033 | 0.055 | 0.066 | 0.05 | 0.05 | 0.11 | 0.03 | 0.02 | 0.05 | 0.07 | 0.126 | 0.091 |
| 61 | 0.026 | 0.028 | 0.038 | 0.061 | 0.02 | 0.071 | 0.052 | 0.033 | 0.03 | 0.03 | 0.07 | 0.07 |
| 62 | 0.049 | 0.083 | 0.009 | 0.071 | 0.05 | 0.072 | 0.03 | 0.03 | 0.042 | 0.04 | 0.111 | 0.081 |
| 63 | 0.034 | 0.044 | 0.04 | 0.02 | 0.05 | 0.089 | 0.02 | 0.042 | 0.04 | 0.05 | 0.05 | 0.071 |
| 64 | 0.034 | 0.033 | 0.055 | 0.07 | 0.04 | 0.03 | 0.042 | 0.033 | 0.033 | 0.05 | 0.052 | 0.1 |
| 65 | 0.04 | 0.027 | 0.022 | 0.06 | 0.06 | 0.042 | 0.04 | 0.03 | 0.057 | 0.04 | 0.03 | 0.042 |
| 66 | 0.054 | 0.046 | 0.034 | 0.05 | 0.01 | 0.052 | 0.03 | 0.03 | 0.07 | 0.03 | 0.04 | 0.113 |
| 67 | 0.041 | 0.061 | 0.039 | 0.04 | 0.061 | 0.04 | 0.042 | 0.048 | 0.04 | 0.04 | 0.042 | 0.07 |
| 68 | 0.018 | 0.061 | 0.027 | 0.07 | 0.04 | 0.1 | 0.04 | 0.03 | 0.03 | 0.052 | 0.05 | 0.02 |
| 69 | 0.005 | 0.049 | 0.026 | 0.042 | 0.052 | 0.06 | 0.03 | 0.04 | 0.048 | 0.06 | 0.04 | 0.133 |
| 70 | 0.045 | 0.049 | 0.06 | 0.06 | 0.075 | 0.09 | 0.03 | 0.04 | 0.057 | 0.052 | 0.06 | 0.091 |
| 71 | 0.028 | 0.018 | 0.041 | 0.04 | 0.103 | 0.04 | 0.03 | 0.05 | 0.057 | 0.073 | 0.07 | 0.09 |
| 72 | 0.022 | 0.038 | 0.027 | 0.04 | 0.07 | 0.07 | 0.01 | 0.03 | 0.052 | 0.07 | 0.094 | 0.103 |
| 73 | 0.016 | 0.023 | 0.023 | 0.04 | 0.07 | 0.08 | 0.042 | 0.02 | 0.033 | 0.08 | 0.09 | 0.084 |
| 74 | 0.033 | 0.039 | 0.052 | 0.04 | 0.091 | 0.061 | 0.03 | 0.05 | 0.033 | 0.06 | 0.09 | 0.084 |
| 75 | 0.044 | 0.044 | 0.033 | 0.052 | 0.091 | 0.09 | 0.03 | 0.04 | 0.05 | 0.05 | 0.052 | 0.094 |
| 76 | 0.038 | 0.033 | 0.027 | 0.04 | 0.094 | 0.09 | 0.02 | 0.03 | 0.02 | 0.052 | 0.061 | 0.096 |
| 77 | 0.027 | 0.066 | 0.022 | 0.061 | 0.071 | 0.07 | 0.042 | 0.03 | 0.05 | 0.071 | 0.09 | 0.048 |
| 78 | 0.038 | 0.033 | 0.073 | 0.061 | 0.06 | 0.131 | 0.06 | 0.02 | 0.033 | 0.048 | 0.113 | 0.09 |
| 79 | 0.027 | 0.07 | 0.022 | 0.042 | 0.071 | 0.151 | 0.03 | 0.052 | 0.061 | 0.042 | 0.06 | 0.111 |
| 80 | 0.049 | 0.05 | 0.027 | 0.033 | 0.066 | 0.101 | 0.02 | 0.04 | 0.03 | 0.03 | 0.04 | 0.07 |
| 81 | 0.022 | 0.016 | 0.039 | 0.052 | 0.07 | 0.071 | 0.03 | 0.094 | 0.05 | 0.042 | 0.081 | 0.052 |
| 82 | 0.045 | 0.041 | 0.027 | 0.052 | 0.1 | 0.061 | 0.048 | 0.06 | 0.01 | 0.05 | 0.081 | 0.06 |
| 83 | 0.028 | 0.022 | 0.034 | 0.04 | 0.04 | 0.06 | 0.04 | 0.04 | 0.024 | 0.081 | 0.052 | 0.061 |
| 84 | 0.027 | 0.062 | 0.038 | 0.07 | 0.05 | 0.061 | 0.03 | 0.04 | 0.04 | 0.081 | 0.061 | 0.06 |
| 85 | 0.022 | 0.022 | 0.055 | 0.052 | 0.07 | 0.052 | 0.052 | 0.07 | 0.01 | 0.061 | 0.094 | 0.03 |
| 86 | 0.027 | 0.039 | 0.038 | 0.061 | 0.07 | 0.081 | 0.03 | 0.04 | 0.05 | 0.066 | 0.03 | 0.05 |
| 87 | 0.05 | 0.033 | 0.038 | 0.08 | 0.08 | 0.06 | 0.061 | 0.042 | 0.05 | 0.05 | 0.111 | 0.103 |
| 88 | 0.038 | 0.031 | 0.038 | 0.07 | 0.111 | 0.048 | 0.04 | 0.04 | 0.052 | 0.06 | 0.08 | 0.024 |
| 89 | 0.045 | 0.033 | 0.027 | 0.06 | 0.091 | 0.126 | 0.061 | 0.04 | 0.033 | 0.042 | 0.07 | 0.081 |
| 90 | 0.027 | 0.033 | 0.027 | 0.057 | 0.101 | 0.04 | 0.03 | 0.05 | 0.042 | 0.08 | 0.081 | 0.11 |
| 91 | 0.022 | 0.028 | 0.033 | 0.09 | 0.101 | 0.06 | 0.03 | 0.05 | 0.052 | 0.06 | 0.143 | 0.075 |
| 92 | 0.033 | 0.022 | 0.039 | 0.06 | 0.06 | 0.075 | 0.04 | 0.02 | 0.042 | 0.06 | 0.19 | 0.103 |
| 93 | 0.023 | 0.06 | 0.045 | 0.05 | 0.04 | 0.091 | 0.03 | 0.02 | 0.03 | 0.057 | 0.06 | 0.103 |
| 94 | 0.06 | 0.023 | 0.049 | 0.05 | 0.081 | 0.081 | 0.02 | 0.03 | 0.033 | 0.071 | 0.057 | 0.04 |
| 95 | 0.052 | 0.046 | 0.06 | 0.061 | 0.08 | 0.052 | 0.033 | 0.05 | 0.042 | 0.03 | 0.03 | 0.061 |
| 96 | 0.022 | 0.028 | 0.055 | 0.061 | 0.07 | 0.101 | 0.04 | 0.033 | 0.052 | 0.072 | 0.101 | 0.09 |
| 97 | 0.055 | 0.045 | 0.044 | 0.05 | 0.061 | 0.09 | 0.03 | 0.03 | 0.052 | 0.08 | 0.08 | 0.081 |
| 98 | 0.055 | 0.026 | 0.044 | 0.057 | 0.057 | 0.06 | 0.042 | 0.04 | 0.07 | 0.05 | 0.05 | 0.05 |
| 99 | 0.016 | 0.083 | 0.023 | 0.052 | 0.071 | 0.075 | 0.03 | 0.071 | 0.07 | 0.081 | 0.05 | 0.081 |
| 100 | 0.016 | 0.062 | 0.022 | 0.06 | 0.108 | 0.091 | 0.017 | 0.04 | 0.052 | 0.081 | 0.07 | 0.089 |
| 101 | 0.027 | 0.045 | 0.072 | 0.084 | 0.042 | 0.1 | 0.01 | 0.042 | 0.033 | 0.071 | 0.09 | 0.101 |
| 102 | 0.033 | 0.016 | 0.022 | 0.06 | 0.06 | 0.071 | 0.042 | 0.061 | 0.05 | 0.07 | 0.06 | 0.09 |
| 103 | 0.039 | 0.031 | 0.045 | 0.033 | 0.094 | 0.094 | 0.04 | 0.042 | 0.033 | 0.061 | 0.061 | 0.071 |
| 104 | 0.07 | 0.055 | 0.044 | 0.06 | 0.07 | 0.072 | 0.04 | 0.03 | 0.03 | 0.052 | 0.04 | 0.09 |
| 105 | 0.027 | 0.033 | 0.044 | 0.052 | 0.091 | 0.091 | 0.033 | 0.042 | 0.01 | 0.05 | 0.04 | 0.05 |
| 106 | 0.033 | 0.071 | 0.033 | 0.04 | 0.08 | 0.03 | 0.042 | 0.03 | 0.042 | 0.048 | 0.04 | 0.084 |
| 107 | 0.033 | 0.038 | 0.066 | 0.03 | 0.08 | 0.066 | 0.033 | 0.03 | 0.042 | 0.052 | 0.081 | 0.061 |
| 108 | 0.066 | 0.055 | 0.038 | 0.04 | 0.061 | 0.061 | 0.04 | 0.052 | 0.02 | 0.06 | 0.075 | 0.04 |
| 109 | 0.033 | 0.044 | 0.033 | 0.05 | 0.05 | 0.052 | 0.042 | 0.05 | 0.05 | 0.04 | 0.08 | 0.05 |
| 110 | 0.036 | 0.055 | 0.055 | 0.045 | 0.05 | 0.02 | 0.02 | 0.04 | 0.04 | 0.05 | 0.1 | 0.057 |
| 111 | 0.055 | 0.026 | 0.038 | 0.04 | 0.04 | 0.05 | 0.033 | 0.033 | 0.04 | 0.04 | 0.03 | 0.052 |
| 112 | 0.023 | 0.011 | 0.05 | 0.05 | 0.061 | 0.071 | 0.042 | 0.04 | 0.05 | 0.05 | 0.06 | 0.07 |
| 113 | 0.039 | 0.055 | 0.034 | 0.04 | 0.04 | 0.113 | 0.03 | 0.033 | 0.04 | 0.06 | 0.091 | 0.07 |
| 114 | 0.055 | 0.033 | 0.033 | 0.05 | 0.04 | 0.08 | 0.05 | 0.052 | 0.04 | 0.071 | 0.07 | 0.111 |
| 115 | 0.025 | 0.022 | 0.033 | 0.091 | 0.04 | 0.08 | 0.02 | 0.04 | 0.04 | 0.075 | 0.042 | 0.08 |
| 116 | 0.041 | 0.039 | 0.027 | 0.04 | 0.05 | 0.07 | 0.03 | 0.05 | 0.04 | 0.11 | 0.07 | 0.07 |
| 117 | 0.033 | 0.033 | 0.034 | 0.057 | 0.052 | 0.04 | 0.03 | 0.033 | 0.033 | 0.03 | 0.06 | 0.09 |
| 118 | 0.044 | 0.041 | 0.044 | 0.02 | 0.075 | 0.042 | 0.02 | 0.04 | 0.03 | 0.06 | 0.111 | 0.08 |
| 119 | 0.044 | 0.044 | 0.033 | 0.07 | 0.05 | 0.052 | 0.042 | 0.03 | 0.02 | 0.052 | 0.1 | 0.061 |
| 120 | 0.045 | 0.022 | 0.049 | 0.024 | 0.131 | 0.06 | 0.03 | 0.04 | 0.02 | 0.04 | 0.081 | 0.052 |
| 121 | 0.038 | 0.055 | 0.055 | 0.048 | 0.066 | 0.05 | 0.02 | 0.04 | 0.05 | 0.042 | 0.13 | 0.07 |
| 122 | 0.038 | 0.044 | 0.022 | 0.066 | 0.081 | 0.061 | 0.03 | 0.03 | 0.03 | 0.089 | 0.075 | 0.04 |
| 123 | 0.038 | 0.034 | 0.022 | 0.052 | 0.103 | 0.07 | 0.03 | 0.04 | 0.033 | 0.04 | 0.061 | 0.061 |
| 124 | 0.038 | 0.055 | 0.045 | 0.061 | 0.03 | 0.06 | 0.02 | 0.01 | 0.03 | 0.071 | 0.042 | 0.06 |
| 125 | 0.033 | 0.066 | 0.034 | 0.052 | 0.057 | 0.071 | 0.04 | 0.033 | 0.04 | 0.061 | 0.15 | 0.06 |
| 126 | 0.038 | 0.045 | 0.022 | 0.042 | 0.066 | 0.03 | 0.033 | 0.03 | 0.03 | 0.101 | 0.121 | 0.084 |
| 127 | 0.055 | 0.022 | 0.033 | 0.033 | 0.075 | 0.042 | 0.04 | 0.02 | 0.033 | 0.075 | 0.08 | 0.09 |
| 128 | 0.022 | 0.061 | 0.044 | 0.033 | 0.071 | 0.04 | 0.04 | 0.04 | 0.042 | 0.06 | 0.052 | 0.061 |
| 129 | 0.033 | 0.028 | 0.022 | 0.052 | 0.09 | 0.084 | 0.05 | 0.02 | 0.04 | 0.06 | 0.12 | 0.1 |
| 130 | 0.052 | 0.028 | 0.061 | 0.033 | 0.03 | 0.05 | 0.042 | 0.042 | 0.033 | 0.071 | 0.064 | 0.09 |
| 131 | 0.033 | 0.046 | 0.027 | 0.04 | 0.024 | 0.05 | 0.03 | 0.04 | 0.042 | 0.061 | 0.057 | 0.081 |
| 132 | 0.022 | 0.06 | 0.027 | 0.057 | 0.042 | 0.09 | 0.05 | 0.052 | 0.042 | 0.1 | 0.08 | 0.143 |
| 133 | 0.044 | 0.049 | 0.044 | 0.052 | 0.1 | 0.06 | 0.052 | 0.042 | 0.03 | 0.02 | 0.061 | 0.07 |
| 134 | 0.05 | 0.044 | 0.028 | 0.075 | 0.03 | 0.07 | 0.05 | 0.03 | 0.06 | 0.07 | 0.064 | 0.071 |
| 135 | 0.038 | 0.059 | 0.016 | 0.04 | 0.05 | 0.091 | 0.03 | 0.06 | 0.04 | 0.12 | 0.066 | 0.05 |
| 136 | 0.068 | 0.031 | 0.033 | 0.052 | 0.061 | 0.07 | 0.02 | 0.042 | 0.061 | 0.091 | 0.057 | 0.08 |
| 137 | 0.031 | 0.038 | 0.039 | 0.042 | 0.094 | 0.04 | 0.03 | 0.052 | 0.033 | 0.08 | 0.04 | 0.07 |
| 138 | 0.038 | 0.044 | 0.027 | 0.05 | 0.04 | 0.03 | 0.06 | 0.03 | 0.03 | 0.024 | 0.052 | 0.061 |
| 139 | 0.046 | 0.049 | 0.061 | 0.05 | 0.057 | 0.04 | 0.052 | 0.033 | 0.02 | 0.05 | 0.09 | 0.071 |
| 140 | 0.027 | 0.034 | 0.046 | 0.03 | 0.06 | 0.024 | 0.052 | 0.033 | 0.04 | 0.05 | 0.061 | 0.07 |
| 141 | 0.031 | 0.044 | 0.055 | 0.06 | 0.04 | 0.071 | 0.052 | 0.02 | 0.05 | 0.08 | 0.07 | 0.07 |
| 142 | 0.027 | 0.039 | 0.049 | 0.05 | 0.04 | 0.07 | 0.03 | 0.033 | 0.03 | 0.081 | 0.101 | 0.07 |
| 143 | 0.023 | 0.023 | 0.022 | 0.052 | 0.06 | 0.141 | 0.03 | 0.03 | 0.05 | 0.052 | 0.13 | 0.111 |
| 144 | 0.044 | 0.055 | 0.055 | 0.033 | 0.05 | 0.101 | 0.05 | 0.02 | 0.05 | 0.06 | 0.09 | 0.101 |
| 145 | 0.061 | 0.038 | 0.034 | 0.033 | 0.091 | 0.052 | 0.033 | 0.05 | 0.081 | 0.071 | 0.05 | 0.09 |
| 146 | 0.039 | 0.055 | 0.046 | 0.05 | 0.108 | 0.061 | 0.04 | 0.02 | 0.04 | 0.05 | 0.061 | 0.03 |
| 147 | 0.044 | 0.062 | 0.038 | 0.072 | 0.07 | 0.057 | 0.03 | 0.033 | 0.04 | 0.06 | 0.1 | 0.06 |
| 148 | 0.031 | 0.045 | 0.027 | 0.057 | 0.094 | 0.05 | 0.03 | 0.017 | 0.03 | 0.06 | 0.07 | 0.05 |
| 149 | 0.062 | 0.045 | 0.06 | 0.057 | 0.071 | 0.1 | 0.03 | 0.033 | 0.05 | 0.05 | 0.05 | 0.042 |
| 150 | 0.033 | 0.034 | 0.049 | 0.071 | 0.06 | 0.081 | 0.033 | 0.052 | 0.07 | 0.061 | 0.057 | 0.05 |
| 151 | 0.031 | 0.034 | 0.049 | 0.06 | 0.05 | 0.101 | 0.052 | 0.042 | 0.061 | 0.06 | 0.07 | 0.08 |
| 152 | 0.045 | 0.052 | 0.028 | 0.04 | 0.075 | 0.081 | 0.01 | 0.03 | 0.06 | 0.09 | 0.091 | 0.08 |
| 153 | 0.062 | 0.044 | 0.05 | 0.06 | 0.098 | 0.101 | 0.017 | 0.02 | 0.04 | 0.103 | 0.04 | 0.071 |
| 154 | 0.038 | 0.034 | 0.034 | 0.06 | 0.113 | 0.08 | 0.052 | 0.03 | 0.03 | 0.101 | 0.052 | 0.081 |
| 155 | 0.011 | 0.046 | 0.028 | 0.06 | 0.042 | 0.08 | 0.05 | 0.033 | 0.02 | 0.061 | 0.117 | 0.06 |
| 156 | 0.023 | 0.039 | 0.039 | 0.05 | 0.052 | 0.061 | 0.03 | 0.03 | 0.03 | 0.07 | 0.1 | 0.09 |
| 157 | 0.033 | 0.044 | 0.068 | 0.05 | 0.075 | 0.052 | 0.033 | 0.04 | 0.042 | 0.06 | 0.09 | 0.07 |
| 158 | 0.033 | 0.028 | 0.044 | 0.06 | 0.057 | 0.094 | 0.01 | 0.04 | 0.052 | 0.06 | 0.06 | 0.06 |
| 159 | 0.062 | 0.057 | 0.082 | 0.105 | 0.061 | 0.06 | 0.04 | 0.042 | 0.03 | 0.057 | 0.1 | 0.111 |
| 160 | 0.044 | 0.06 | 0.033 | 0.052 | 0.052 | 0.066 | 0.04 | 0.05 | 0.024 | 0.08 | 0.08 | 0.05 |
| 161 | 0.027 | 0.049 | 0.018 | 0.052 | 0.075 | 0.04 | 0.033 | 0.06 | 0.024 | 0.071 | 0.07 | 0.07 |
| 162 | 0.038 | 0.027 | 0.055 | 0.08 | 0.089 | 0.094 | 0.04 | 0.066 | 0.042 | 0.05 | 0.08 | 0.081 |
| 163 | 0.035 | 0.038 | 0.038 | 0.08 | 0.08 | 0.094 | 0.04 | 0.033 | 0.048 | 0.06 | 0.05 | 0.091 |
| 164 | 0.027 | 0.055 | 0.034 | 0.04 | 0.096 | 0.084 | 0.042 | 0.06 | 0.042 | 0.12 | 0.05 | 0.1 |
| 165 | 0.038 | 0.062 | 0.039 | 0.061 | 0.071 | 0.113 | 0.04 | 0.042 | 0.042 | 0.091 | 0.05 | 0.061 |
| 166 | 0.075 | 0.026 | 0.055 | 0.05 | 0.094 | 0.09 | 0.033 | 0.05 | 0.042 | 0.03 | 0.04 | 0.1 |
| 167 | 0.052 | 0.055 | 0.055 | 0.081 | 0.084 | 0.042 | 0.042 | 0.04 | 0.042 | 0.05 | 0.081 | 0.081 |
| 168 | 0.038 | 0.05 | 0.038 | 0.042 | 0.05 | 0.08 | 0.024 | 0.03 | 0.057 | 0.042 | 0.091 | 0.071 |
| 169 | 0.033 | 0.038 | 0.057 | 0.052 | 0.101 | 0.09 | 0.04 | 0.04 | 0.04 | 0.04 | 0.08 | 0.072 |
| 170 | 0.033 | 0.06 | 0.066 | 0.05 | 0.061 | 0.05 | 0.05 | 0.04 | 0.033 | 0.061 | 0.11 | 0.042 |
| 171 | 0.049 | 0.034 | 0.027 | 0.042 | 0.061 | 0.042 | 0.02 | 0.05 | 0.06 | 0.061 | 0.066 | 0.113 |
| 172 | 0.022 | 0.082 | 0.018 | 0.03 | 0.084 | 0.08 | 0.024 | 0.05 | 0.05 | 0.07 | 0.05 | 0.141 |
| 173 | 0.049 | 0.022 | 0.041 | 0.03 | 0.05 | 0.09 | 0.04 | 0.04 | 0.033 | 0.05 | 0.05 | 0.07 |
| 174 | 0.061 | 0.055 | 0.05 | 0.085 | 0.06 | 0.05 | 0.05 | 0.042 | 0.02 | 0.08 | 0.042 | 0.071 |
| 175 | 0.031 | 0.016 | 0.061 | 0.052 | 0.075 | 0.07 | 0.04 | 0.02 | 0.033 | 0.08 | 0.06 | 0.08 |
| 176 | 0.039 | 0.038 | 0.055 | 0.06 | 0.088 | 0.081 | 0.03 | 0.05 | 0.02 | 0.042 | 0.113 | 0.071 |
| 177 | 0.041 | 0.049 | 0.049 | 0.03 | 0.06 | 0.033 | 0.042 | 0.042 | 0.05 | 0.101 | 0.06 | 0.101 |
| 178 | 0.023 | 0.038 | 0.031 | 0.03 | 0.094 | 0.071 | 0.057 | 0.057 | 0.03 | 0.05 | 0.04 | 0.1 |
| 179 | 0.023 | 0.054 | 0.062 | 0.042 | 0.098 | 0.1 | 0.033 | 0.05 | 0.033 | 0.08 | 0.061 | 0.161 |
| 180 | 0.055 | 0.049 | 0.022 | 0.04 | 0.071 | 0.052 | 0.06 | 0.02 | 0.042 | 0.084 | 0.091 | 0.101 |
| 181 | 0.061 | 0.049 | 0.036 | 0.042 | 0.089 | 0.052 | 0.024 | 0.05 | 0.05 | 0.084 | 0.08 | 0.091 |
| 182 | 0.034 | 0.044 | 0.038 | 0.03 | 0.084 | 0.04 | 0.03 | 0.042 | 0.052 | 0.081 | 0.06 | 0.111 |
| 183 | 0.044 | 0.054 | 0.027 | 0.042 | 0.033 | 0.052 | 0.024 | 0.03 | 0.05 | 0.081 | 0.081 | 0.05 |
| 184 | 0.038 | 0.038 | 0.022 | 0.05 | 0.071 | 0.03 | 0.052 | 0.033 | 0.03 | 0.071 | 0.075 | 0.08 |
| 185 | 0.022 | 0.028 | 0.035 | 0.033 | 0.061 | 0.052 | 0.04 | 0.03 | 0.042 | 0.061 | 0.052 | 0.061 |
| 186 | 0.045 | 0.052 | 0.027 | 0.02 | 0.12 | 0.052 | 0.04 | 0.042 | 0.04 | 0.111 | 0.06 | 0.07 |
| 187 | 0.057 | 0.039 | 0.016 | 0.02 | 0.108 | 0.05 | 0.052 | 0.04 | 0.05 | 0.05 | 0.048 | 0.06 |
| 188 | 0.022 | 0.045 | 0.055 | 0.042 | 0.091 | 0.033 | 0.017 | 0.075 | 0.06 | 0.1 | 0.04 | 0.052 |
| 189 | 0.05 | 0.044 | 0.06 | 0.03 | 0.061 | 0.05 | 0.033 | 0.052 | 0.04 | 0.071 | 0.057 | 0.08 |
| 190 | 0.028 | 0.05 | 0.055 | 0.02 | 0.121 | 0.061 | 0.05 | 0.02 | 0.03 | 0.101 | 0.04 | 0.084 |
| 191 | 0.033 | 0.018 | 0.055 | 0.061 | 0.06 | 0.05 | 0.03 | 0.04 | 0.06 | 0.075 | 0.052 | 0.113 |
| 192 | 0.06 | 0.049 | 0.034 | 0.03 | 0.061 | 0.1 | 0.024 | 0.02 | 0.042 | 0.07 | 0.1 | 0.11 |
| 193 | 0.033 | 0.072 | 0.055 | 0.04 | 0.108 | 0.133 | 0.02 | 0.042 | 0.048 | 0.066 | 0.113 | 0.123 |
| 194 | 0.034 | 0.046 | 0.039 | 0.03 | 0.088 | 0.111 | 0.04 | 0.02 | 0.05 | 0.098 | 0.09 | 0.117 |
| 195 | 0.038 | 0.044 | 0.027 | 0.04 | 0.08 | 0.033 | 0.05 | 0.03 | 0.05 | 0.04 | 0.061 | 0.104 |
| 196 | 0.016 | 0.044 | 0.044 | 0.07 | 0.05 | 0.04 | 0.03 | 0.033 | 0.06 | 0.071 | 0.105 | 0.081 |
| 197 | 0.061 | 0.052 | 0.039 | 0.05 | 0.075 | 0.042 | 0.06 | 0.03 | 0.06 | 0.081 | 0.05 | 0.081 |
| 198 | 0.027 | 0.041 | 0.038 | 0.02 | 0.071 | 0.061 | 0.03 | 0.042 | 0.04 | 0.111 | 0.108 | 0.101 |
| 199 | 0.07 | 0.04 | 0.048 | 0.07 | 0.06 | 0.05 | 0.05 | 0.024 | 0.04 | 0.111 | 0.144 | 0.136 |
| 200 | 0.066 | 0.038 | 0.066 | 0.07 | 0.07 | 0.06 | 0.03 | 0.05 | 0.06 | 0.117 | 0.064 | 0.094 |
| 201 | 0.044 | 0.023 | 0.034 | 0.075 | 0.05 | 0.02 | 0.052 | 0.04 | 0.05 | 0.1 | 0.048 | 0.066 |
| 202 | 0.052 | 0.033 | 0.039 | 0.104 | 0.071 | 0.075 | 0.03 | 0.03 | 0.061 | 0.081 | 0.14 | 0.075 |
| 203 | 0.039 | 0.027 | 0.044 | 0.048 | 0.081 | 0.05 | 0.04 | 0.04 | 0.081 | 0.057 | 0.13 | 0.071 |
| 204 | 0.052 | 0.045 | 0.044 | 0.057 | 0.052 | 0.06 | 0.052 | 0.052 | 0.05 | 0.09 | 0.071 | 0.11 |
| 205 | 0.052 | 0.044 | 0.027 | 0.071 | 0.071 | 0.03 | 0.071 | 0.042 | 0.04 | 0.05 | 0.113 | 0.155 |
| 206 | 0.044 | 0.05 | 0.057 | 0.072 | 0.075 | 0.042 | 0.08 | 0.04 | 0.04 | 0.094 | 0.033 | 0.1 |
| 207 | 0.066 | 0.038 | 0.044 | 0.066 | 0.075 | 0.071 | 0.052 | 0.033 | 0.03 | 0.05 | 0.081 | 0.133 |
| 208 | 0.059 | 0.038 | 0.055 | 0.04 | 0.06 | 0.04 | 0.052 | 0.033 | 0.04 | 0.081 | 0.075 | 0.081 |
| 209 | 0.052 | 0.033 | 0.06 | 0.06 | 0.05 | 0.05 | 0.033 | 0.042 | 0.07 | 0.06 | 0.12 | 0.07 |
| 210 | 0.084 | 0.028 | 0.049 | 0.061 | 0.042 | 0.08 | 0.033 | 0.04 | 0.05 | 0.06 | 0.07 | 0.08 |
| 211 | 0.044 | 0.033 | 0.06 | 0.02 | 0.057 | 0.073 | 0.061 | 0.05 | 0.03 | 0.081 | 0.081 | 0.05 |
| 212 | 0.034 | 0.033 | 0.05 | 0.05 | 0.042 | 0.033 | 0.07 | 0.07 | 0.024 | 0.143 | 0.033 | 0.081 |
| 213 | 0.049 | 0.027 | 0.027 | 0.04 | 0.071 | 0.071 | 0.042 | 0.061 | 0.05 | 0.113 | 0.111 | 0.108 |
| 214 | 0.027 | 0.022 | 0.038 | 0.04 | 0.057 | 0.06 | 0.06 | 0.052 | 0.03 | 0.071 | 0.108 | 0.09 |
| 215 | 0.058 | 0.028 | 0.044 | 0.05 | 0.108 | 0.048 | 0.05 | 0.05 | 0.028 | 0.061 | 0.09 | 0.08 |
| 216 | 0.06 | 0.026 | 0.044 | 0.08 | 0.052 | 0.061 | 0.024 | 0.03 | 0.071 | 0.123 | 0.09 | 0.1 |
| 217 | 0.055 | 0.05 | 0.071 | 0.03 | 0.081 | 0.071 | 0.05 | 0.03 | 0.052 | 0.126 | 0.084 | 0.084 |
| 218 | 0.023 | 0.045 | 0.088 | 0.033 | 0.066 | 0.052 | 0.033 | 0.04 | 0.06 | 0.121 | 0.131 | 0.1 |
| 219 | 0.033 | 0.052 | 0.091 | 0.072 | 0.052 | 0.052 | 0.01 | 0.04 | 0.02 | 0.081 | 0.088 | 0.075 |
| 220 | 0.066 | 0.058 | 0.062 | 0.07 | 0.048 | 0.081 | 0.052 | 0.033 | 0.03 | 0.11 | 0.143 | 0.08 |
| 221 | 0.055 | 0.049 | 0.07 | 0.05 | 0.094 | 0.075 | 0.06 | 0.04 | 0.08 | 0.1 | 0.108 | 0.152 |
| 222 | 0.044 | 0.055 | 0.031 | 0.08 | 0.103 | 0.061 | 0.033 | 0.04 | 0.042 | 0.05 | 0.094 | 0.113 |
| 223 | 0.06 | 0.033 | 0.05 | 0.05 | 0.071 | 0.08 | 0.06 | 0.033 | 0.04 | 0.071 | 0.108 | 0.136 |
| 224 | 0.027 | 0.038 | 0.055 | 0.07 | 0.06 | 0.061 | 0.03 | 0.04 | 0.07 | 0.08 | 0.094 | 0.1 |
| 225 | 0.028 | 0.049 | 0.045 | 0.03 | 0.08 | 0.075 | 0.04 | 0.042 | 0.064 | 0.071 | 0.084 | 0.081 |
| 226 | 0.023 | 0.027 | 0.06 | 0.06 | 0.081 | 0.169 | 0.061 | 0.04 | 0.06 | 0.103 | 0.075 | 0.207 |
| 227 | 0.044 | 0.049 | 0.045 | 0.052 | 0.101 | 0.081 | 0.052 | 0.06 | 0.05 | 0.06 | 0.096 | 0.081 |
| 228 | 0.066 | 0.044 | 0.057 | 0.061 | 0.123 | 0.061 | 0.02 | 0.04 | 0.04 | 0.094 | 0.06 | 0.071 |
| 229 | 0.049 | 0.044 | 0.052 | 0.057 | 0.071 | 0.09 | 0.042 | 0.02 | 0.04 | 0.111 | 0.05 | 0.108 |
| 230 | 0.044 | 0.033 | 0.049 | 0.061 | 0.123 | 0.05 | 0.04 | 0.042 | 0.033 | 0.098 | 0.111 | 0.071 |
| 231 | 0.055 | 0.022 | 0.022 | 0.03 | 0.071 | 0.071 | 0.06 | 0.06 | 0.06 | 0.081 | 0.081 | 0.151 |
| 232 | 0.045 | 0.038 | 0.044 | 0.024 | 0.075 | 0.06 | 0.042 | 0.05 | 0.03 | 0.162 | 0.04 | 0.16 |
| 233 | 0.018 | 0.033 | 0.06 | 0.042 | 0.111 | 0.06 | 0.04 | 0.03 | 0.03 | 0.081 | 0.028 | 0.101 |
| 234 | 0.039 | 0.038 | 0.082 | 0.064 | 0.033 | 0.081 | 0.05 | 0.04 | 0.05 | 0.098 | 0.136 | 0.14 |
| 235 | 0.055 | 0.033 | 0.078 | 0.052 | 0.15 | 0.136 | 0.04 | 0.064 | 0.02 | 0.066 | 0.05 | 0.101 |
| 236 | 0.038 | 0.033 | 0.071 | 0.061 | 0.081 | 0.08 | 0.024 | 0.06 | 0.04 | 0.08 | 0.1 | 0.108 |
| 237 | 0.049 | 0.026 | 0.066 | 0.01 | 0.061 | 0.094 | 0.024 | 0.06 | 0.05 | 0.05 | 0.081 | 0.064 |
| 238 | 0.027 | 0.033 | 0.057 | 0.05 | 0.066 | 0.12 | 0.017 | 0.042 | 0.042 | 0.05 | 0.1 | 0.081 |
| 239 | 0.059 | 0.028 | 0.038 | 0.06 | 0.129 | 0.05 | 0.024 | 0.06 | 0.052 | 0.08 | 0.07 | 0.09 |
| 240 | 0.011 | 0.038 | 0.033 | 0.042 | 0.081 | 0.06 | 0.02 | 0.042 | 0.05 | 0.07 | 0.09 | 0.12 |
| 241 | 0.034 | 0.084 | 0.06 | 0.04 | 0.07 | 0.05 | 0.04 | 0.04 | 0.052 | 0.075 | 0.05 | 0.075 |
| 242 | 0.059 | 0.06 | 0.059 | 0.024 | 0.042 | 0.061 | 0.017 | 0.02 | 0.07 | 0.1 | 0.052 | 0.143 |
| 243 | 0.028 | 0.027 | 0.045 | 0.03 | 0.165 | 0.075 | 0.057 | 0.01 | 0.042 | 0.05 | 0.091 | 0.151 |
| 244 | 0.052 | 0.044 | 0.064 | 0.03 | 0.05 | 0.075 | 0.048 | 0.04 | 0.03 | 0.02 | 0.084 | 0.1 |
| 245 | 0.055 | 0.061 | 0.09 | 0.042 | 0.103 | 0.084 | 0.033 | 0.03 | 0.042 | 0.101 | 0.075 | 0.081 |
| 246 | 0.038 | 0.023 | 0.057 | 0.03 | 0.13 | 0.07 | 0.03 | 0.057 | 0.03 | 0.061 | 0.091 | 0.091 |
| 247 | 0.045 | 0.022 | 0.05 | 0.084 | 0.131 | 0.075 | 0.024 | 0.06 | 0.071 | 0.1 | 0.071 | 0.071 |
| 248 | 0.033 | 0.027 | 0.046 | 0.064 | 0.101 | 0.042 | 0.061 | 0.05 | 0.02 | 0.103 | 0.06 | 0.159 |
| 249 | 0.064 | 0.022 | 0.049 | 0.081 | 0.084 | 0.07 | 0.02 | 0.03 | 0.042 | 0.09 | 0.07 | 0.131 |
| 250 | 0.034 | 0.023 | 0.062 | 0.089 | 0.14 | 0.101 | 0.052 | 0.033 | 0.03 | 0.042 | 0.04 | 0.108 |
| Mean | 0.039464 | 0.041356 | 0.04306 | 0.051408 | 0.068032 | 0.066956 | 0.038552 | 0.038552 | 0.042352 | 0.067364 | 0.075124 | 0.082024 |
| SD | 0.014338 | 0.014403 | 0.015639 | 0.01887 | 0.025509 | 0.024352 | 0.013775 | 0.012903 | 0.013805 | 0.024287 | 0.027394 | 0.027534 |

|  | Day 4 | | | | | | Day 5 | | | | | |
| --- | --- | --- | --- | --- | --- | --- | --- | --- | --- | --- | --- | --- |
| OCT images | Control | | | Infected | | | Control | | | Infected | | |
|  | 1 | 2 | 3 | 1 | 2 | 3 | 1 | 2 | 3 | 1 | 2 | 3 |
| 1 | 0.08 | 0.052 | 0.052 | 0.081 | 0.111 | 0.052 | 0.033 | 0.017 | 0.052 | 0.052 | 0.08 | 0.066 |
| 2 | 0.07 | 0.033 | 0.057 | 0.06 | 0.09 | 0.05 | 0.061 | 0.04 | 0.04 | 0.091 | 0.08 | 0.131 |
| 3 | 0.02 | 0.033 | 0.04 | 0.06 | 0.08 | 0.09 | 0.045 | 0.04 | 0.061 | 0.08 | 0.071 | 0.101 |
| 4 | 0.042 | 0.042 | 0.03 | 0.071 | 0.05 | 0.05 | 0.048 | 0.07 | 0.061 | 0.071 | 0.06 | 0.1 |
| 5 | 0.01 | 0.042 | 0.06 | 0.04 | 0.07 | 0.06 | 0.04 | 0.03 | 0.05 | 0.07 | 0.09 | 0.05 |
| 6 | 0.057 | 0.033 | 0.061 | 0.12 | 0.136 | 0.052 | 0.042 | 0.04 | 0.04 | 0.042 | 0.081 | 0.081 |
| 7 | 0.04 | 0.06 | 0.01 | 0.09 | 0.121 | 0.042 | 0.024 | 0.042 | 0.052 | 0.05 | 0.05 | 0.05 |
| 8 | 0.024 | 0.05 | 0.04 | 0.05 | 0.08 | 0.052 | 0.052 | 0.06 | 0.05 | 0.072 | 0.07 | 0.08 |
| 9 | 0.03 | 0.04 | 0.03 | 0.052 | 0.09 | 0.066 | 0.04 | 0.091 | 0.06 | 0.057 | 0.03 | 0.08 |
| 10 | 0.05 | 0.03 | 0.033 | 0.07 | 0.123 | 0.05 | 0.073 | 0.028 | 0.04 | 0.081 | 0.091 | 0.05 |
| 11 | 0.03 | 0.033 | 0.04 | 0.06 | 0.071 | 0.061 | 0.066 | 0.04 | 0.061 | 0.066 | 0.061 | 0.042 |
| 12 | 0.05 | 0.017 | 0.02 | 0.07 | 0.04 | 0.042 | 0.06 | 0.05 | 0.06 | 0.081 | 0.06 | 0.071 |
| 13 | 0.03 | 0.02 | 0.05 | 0.06 | 0.08 | 0.06 | 0.042 | 0.05 | 0.02 | 0.091 | 0.101 | 0.113 |
| 14 | 0.03 | 0.071 | 0.042 | 0.075 | 0.061 | 0.094 | 0.03 | 0.042 | 0.02 | 0.042 | 0.06 | 0.08 |
| 15 | 0.05 | 0.033 | 0.033 | 0.08 | 0.052 | 0.061 | 0.033 | 0.066 | 0.033 | 0.04 | 0.09 | 0.081 |
| 16 | 0.02 | 0.052 | 0.061 | 0.081 | 0.02 | 0.07 | 0.03 | 0.066 | 0.042 | 0.05 | 0.1 | 0.04 |
| 17 | 0.04 | 0.04 | 0.02 | 0.103 | 0.071 | 0.05 | 0.03 | 0.04 | 0.04 | 0.04 | 0.09 | 0.07 |
| 18 | 0.075 | 0.03 | 0.017 | 0.094 | 0.08 | 0.061 | 0.052 | 0.052 | 0.03 | 0.06 | 0.06 | 0.091 |
| 19 | 0.052 | 0.024 | 0.03 | 0.084 | 0.126 | 0.05 | 0.03 | 0.05 | 0.06 | 0.091 | 0.089 | 0.084 |
| 20 | 0.04 | 0.033 | 0.02 | 0.09 | 0.13 | 0.061 | 0.03 | 0.052 | 0.04 | 0.094 | 0.04 | 0.048 |
| 21 | 0.057 | 0.033 | 0.04 | 0.141 | 0.091 | 0.07 | 0.024 | 0.04 | 0.05 | 0.108 | 0.024 | 0.15 |
| 22 | 0.03 | 0.048 | 0.052 | 0.1 | 0.081 | 0.061 | 0.052 | 0.042 | 0.03 | 0.061 | 0.071 | 0.09 |
| 23 | 0.061 | 0.057 | 0.04 | 0.081 | 0.09 | 0.061 | 0.052 | 0.04 | 0.052 | 0.06 | 0.091 | 0.08 |
| 24 | 0.042 | 0.057 | 0.04 | 0.091 | 0.03 | 0.081 | 0.042 | 0.061 | 0.081 | 0.075 | 0.05 | 0.075 |
| 25 | 0.057 | 0.033 | 0.052 | 0.091 | 0.07 | 0.09 | 0.04 | 0.03 | 0.04 | 0.05 | 0.061 | 0.052 |
| 26 | 0.066 | 0.033 | 0.05 | 0.075 | 0.06 | 0.07 | 0.042 | 0.03 | 0.03 | 0.02 | 0.101 | 0.071 |
| 27 | 0.033 | 0.042 | 0.02 | 0.06 | 0.071 | 0.042 | 0.05 | 0.04 | 0.06 | 0.052 | 0.06 | 0.103 |
| 28 | 0.04 | 0.052 | 0.04 | 0.06 | 0.08 | 0.123 | 0.042 | 0.02 | 0.05 | 0.052 | 0.06 | 0.071 |
| 29 | 0.057 | 0.057 | 0.03 | 0.103 | 0.04 | 0.04 | 0.06 | 0.042 | 0.02 | 0.081 | 0.071 | 0.07 |
| 30 | 0.01 | 0.061 | 0.042 | 0.05 | 0.061 | 0.02 | 0.03 | 0.02 | 0.042 | 0.06 | 0.06 | 0.081 |
| 31 | 0.057 | 0.033 | 0.02 | 0.06 | 0.06 | 0.03 | 0.042 | 0.066 | 0.103 | 0.091 | 0.08 | 0.066 |
| 32 | 0.042 | 0.03 | 0.03 | 0.033 | 0.081 | 0.08 | 0.04 | 0.01 | 0.03 | 0.071 | 0.081 | 0.06 |
| 33 | 0.05 | 0.03 | 0.071 | 0.094 | 0.108 | 0.066 | 0.06 | 0.071 | 0.042 | 0.101 | 0.071 | 0.08 |
| 34 | 0.033 | 0.03 | 0.04 | 0.07 | 0.09 | 0.111 | 0.052 | 0.05 | 0.06 | 0.07 | 0.1 | 0.061 |
| 35 | 0.04 | 0.04 | 0.048 | 0.02 | 0.08 | 0.097 | 0.02 | 0.052 | 0.06 | 0.066 | 0.09 | 0.071 |
| 36 | 0.02 | 0.05 | 0.052 | 0.05 | 0.08 | 0.11 | 0.04 | 0.08 | 0.05 | 0.07 | 0.06 | 0.042 |
| 37 | 0.03 | 0.064 | 0.05 | 0.094 | 0.061 | 0.07 | 0.07 | 0.08 | 0.052 | 0.03 | 0.07 | 0.152 |
| 38 | 0.03 | 0.04 | 0.02 | 0.091 | 0.101 | 0.071 | 0.042 | 0.03 | 0.03 | 0.061 | 0.03 | 0.081 |
| 39 | 0.04 | 0.071 | 0.042 | 0.081 | 0.04 | 0.143 | 0.04 | 0.05 | 0.06 | 0.06 | 0.066 | 0.071 |
| 40 | 0.03 | 0.04 | 0.04 | 0.094 | 0.061 | 0.07 | 0.04 | 0.017 | 0.045 | 0.042 | 0.061 | 0.08 |
| 41 | 0.033 | 0.07 | 0.057 | 0.02 | 0.11 | 0.081 | 0.05 | 0.09 | 0.081 | 0.09 | 0.061 | 0.111 |
| 42 | 0.05 | 0.067 | 0.042 | 0.057 | 0.066 | 0.01 | 0.03 | 0.01 | 0.05 | 0.07 | 0.042 | 0.121 |
| 43 | 0.03 | 0.01 | 0.052 | 0.066 | 0.09 | 0.041 | 0.017 | 0.03 | 0.042 | 0.113 | 0.05 | 0.05 |
| 44 | 0.02 | 0.05 | 0.04 | 0.033 | 0.146 | 0.064 | 0.052 | 0.024 | 0.033 | 0.091 | 0.05 | 0.094 |
| 45 | 0.061 | 0.04 | 0.033 | 0.06 | 0.133 | 0.057 | 0.03 | 0.03 | 0.04 | 0.091 | 0.061 | 0.07 |
| 46 | 0.052 | 0.04 | 0.048 | 0.121 | 0.061 | 0.09 | 0.03 | 0.017 | 0.01 | 0.071 | 0.08 | 0.071 |
| 47 | 0.02 | 0.06 | 0.057 | 0.075 | 0.089 | 0.061 | 0.033 | 0.06 | 0.03 | 0.06 | 0.04 | 0.071 |
| 48 | 0.04 | 0.024 | 0.052 | 0.06 | 0.121 | 0.05 | 0.04 | 0.042 | 0.033 | 0.103 | 0.09 | 0.08 |
| 49 | 0.024 | 0.052 | 0.04 | 0.071 | 0.06 | 0.101 | 0.04 | 0.05 | 0.071 | 0.06 | 0.08 | 0.101 |
| 50 | 0.033 | 0.061 | 0.024 | 0.08 | 0.143 | 0.123 | 0.042 | 0.03 | 0.061 | 0.121 | 0.06 | 0.03 |
| 51 | 0.04 | 0.05 | 0.033 | 0.113 | 0.061 | 0.103 | 0.033 | 0.048 | 0.071 | 0.072 | 0.06 | 0.04 |
| 52 | 0.04 | 0.03 | 0.042 | 0.06 | 0.071 | 0.121 | 0.042 | 0.05 | 0.052 | 0.057 | 0.07 | 0.08 |
| 53 | 0.04 | 0.066 | 0.042 | 0.1 | 0.071 | 0.071 | 0.05 | 0.02 | 0.101 | 0.103 | 0.06 | 0.071 |
| 54 | 0.03 | 0.04 | 0.05 | 0.05 | 0.103 | 0.12 | 0.04 | 0.01 | 0.052 | 0.042 | 0.04 | 0.094 |
| 55 | 0.03 | 0.042 | 0.05 | 0.052 | 0.075 | 0.081 | 0.052 | 0.04 | 0.03 | 0.101 | 0.061 | 0.071 |
| 56 | 0.03 | 0.04 | 0.024 | 0.042 | 0.05 | 0.081 | 0.06 | 0.07 | 0.01 | 0.09 | 0.066 | 0.08 |
| 57 | 0.024 | 0.04 | 0.05 | 0.04 | 0.071 | 0.091 | 0.05 | 0.04 | 0.024 | 0.07 | 0.06 | 0.121 |
| 58 | 0.024 | 0.05 | 0.042 | 0.06 | 0.091 | 0.111 | 0.06 | 0.052 | 0.042 | 0.071 | 0.07 | 0.11 |
| 59 | 0.03 | 0.042 | 0.04 | 0.04 | 0.075 | 0.05 | 0.04 | 0.061 | 0.03 | 0.094 | 0.04 | 0.101 |
| 60 | 0.02 | 0.06 | 0.033 | 0.042 | 0.071 | 0.07 | 0.052 | 0.02 | 0.048 | 0.042 | 0.06 | 0.09 |
| 61 | 0.033 | 0.02 | 0.06 | 0.061 | 0.052 | 0.05 | 0.05 | 0.024 | 0.091 | 0.07 | 0.03 | 0.071 |
| 62 | 0.02 | 0.04 | 0.05 | 0.071 | 0.052 | 0.052 | 0.024 | 0.033 | 0.07 | 0.061 | 0.052 | 0.091 |
| 63 | 0.033 | 0.06 | 0.03 | 0.071 | 0.052 | 0.066 | 0.052 | 0.052 | 0.07 | 0.07 | 0.084 | 0.081 |
| 64 | 0.01 | 0.03 | 0.06 | 0.04 | 0.052 | 0.091 | 0.07 | 0.04 | 0.06 | 0.061 | 0.04 | 0.123 |
| 65 | 0.03 | 0.03 | 0.04 | 0.05 | 0.096 | 0.117 | 0.05 | 0.04 | 0.033 | 0.033 | 0.08 | 0.02 |
| 66 | 0.04 | 0.04 | 0.042 | 0.05 | 0.07 | 0.061 | 0.042 | 0.042 | 0.081 | 0.091 | 0.06 | 0.103 |
| 67 | 0.05 | 0.061 | 0.04 | 0.081 | 0.03 | 0.081 | 0.03 | 0.042 | 0.066 | 0.094 | 0.04 | 0.089 |
| 68 | 0.03 | 0.048 | 0.05 | 0.071 | 0.121 | 0.094 | 0.05 | 0.057 | 0.04 | 0.09 | 0.02 | 0.11 |
| 69 | 0.042 | 0.061 | 0.04 | 0.061 | 0.05 | 0.07 | 0.033 | 0.05 | 0.04 | 0.04 | 0.09 | 0.061 |
| 70 | 0.052 | 0.042 | 0.04 | 0.06 | 0.071 | 0.1 | 0.02 | 0.08 | 0.09 | 0.084 | 0.1 | 0.061 |
| 71 | 0.052 | 0.042 | 0.04 | 0.052 | 0.052 | 0.131 | 0.091 | 0.06 | 0.075 | 0.04 | 0.1 | 0.042 |
| 72 | 0.02 | 0.06 | 0.042 | 0.072 | 0.052 | 0.08 | 0.061 | 0.061 | 0.042 | 0.05 | 0.101 | 0.05 |
| 73 | 0.06 | 0.06 | 0.05 | 0.081 | 0.033 | 0.126 | 0.06 | 0.05 | 0.05 | 0.066 | 0.081 | 0.061 |
| 74 | 0.033 | 0.04 | 0.05 | 0.108 | 0.06 | 0.033 | 0.05 | 0.05 | 0.094 | 0.052 | 0.084 | 0.165 |
| 75 | 0.052 | 0.06 | 0.033 | 0.081 | 0.071 | 0.05 | 0.052 | 0.04 | 0.071 | 0.01 | 0.057 | 0.1 |
| 76 | 0.033 | 0.042 | 0.072 | 0.071 | 0.09 | 0.066 | 0.081 | 0.05 | 0.052 | 0.071 | 0.048 | 0.113 |
| 77 | 0.042 | 0.061 | 0.057 | 0.094 | 0.07 | 0.05 | 0.075 | 0.052 | 0.057 | 0.08 | 0.08 | 0.06 |
| 78 | 0.057 | 0.07 | 0.04 | 0.057 | 0.052 | 0.06 | 0.04 | 0.024 | 0.09 | 0.06 | 0.04 | 0.101 |
| 79 | 0.01 | 0.05 | 0.033 | 0.071 | 0.07 | 0.08 | 0.052 | 0.081 | 0.08 | 0.03 | 0.04 | 0.071 |
| 80 | 0.024 | 0.083 | 0.048 | 0.075 | 0.057 | 0.071 | 0.075 | 0.06 | 0.03 | 0.06 | 0.066 | 0.081 |
| 81 | 0.033 | 0.08 | 0.052 | 0.07 | 0.061 | 0.09 | 0.06 | 0.06 | 0.06 | 0.084 | 0.05 | 0.09 |
| 82 | 0.06 | 0.061 | 0.042 | 0.101 | 0.05 | 0.08 | 0.04 | 0.03 | 0.084 | 0.061 | 0.09 | 0.05 |
| 83 | 0.048 | 0.05 | 0.08 | 0.101 | 0.048 | 0.04 | 0.042 | 0.05 | 0.02 | 0.081 | 0.071 | 0.12 |
| 84 | 0.052 | 0.042 | 0.03 | 0.103 | 0.081 | 0.131 | 0.071 | 0.07 | 0.02 | 0.04 | 0.07 | 0.081 |
| 85 | 0.042 | 0.05 | 0.042 | 0.091 | 0.11 | 0.06 | 0.05 | 0.033 | 0.024 | 0.05 | 0.05 | 0.1 |
| 86 | 0.03 | 0.05 | 0.06 | 0.06 | 0.09 | 0.04 | 0.033 | 0.03 | 0.033 | 0.04 | 0.06 | 0.08 |
| 87 | 0.03 | 0.075 | 0.048 | 0.11 | 0.03 | 0.084 | 0.04 | 0.05 | 0.03 | 0.091 | 0.052 | 0.061 |
| 88 | 0.048 | 0.057 | 0.05 | 0.103 | 0.15 | 0.075 | 0.033 | 0.04 | 0.02 | 0.033 | 0.03 | 0.11 |
| 89 | 0.066 | 0.071 | 0.066 | 0.081 | 0.075 | 0.061 | 0.05 | 0.02 | 0.06 | 0.06 | 0.06 | 0.071 |
| 90 | 0.033 | 0.09 | 0.06 | 0.04 | 0.081 | 0.075 | 0.042 | 0.06 | 0.052 | 0.06 | 0.07 | 0.061 |
| 91 | 0.02 | 0.057 | 0.06 | 0.09 | 0.108 | 0.04 | 0.042 | 0.02 | 0.05 | 0.052 | 0.081 | 0.08 |
| 92 | 0.042 | 0.06 | 0.04 | 0.042 | 0.101 | 0.071 | 0.033 | 0.03 | 0.05 | 0.111 | 0.07 | 0.05 |
| 93 | 0.033 | 0.07 | 0.05 | 0.057 | 0.08 | 0.07 | 0.03 | 0.03 | 0.07 | 0.091 | 0.06 | 0.061 |
| 94 | 0.033 | 0.033 | 0.05 | 0.07 | 0.101 | 0.091 | 0.07 | 0.02 | 0.045 | 0.1 | 0.08 | 0.07 |
| 95 | 0.02 | 0.05 | 0.04 | 0.06 | 0.121 | 0.07 | 0.071 | 0.033 | 0.02 | 0.07 | 0.071 | 0.081 |
| 96 | 0.042 | 0.04 | 0.052 | 0.066 | 0.1 | 0.1 | 0.04 | 0.033 | 0.071 | 0.081 | 0.04 | 0.111 |
| 97 | 0.052 | 0.04 | 0.04 | 0.06 | 0.11 | 0.05 | 0.103 | 0.052 | 0.05 | 0.052 | 0.06 | 0.131 |
| 98 | 0.033 | 0.05 | 0.05 | 0.089 | 0.131 | 0.08 | 0.033 | 0.02 | 0.071 | 0.09 | 0.103 | 0.08 |
| 99 | 0.02 | 0.05 | 0.04 | 0.042 | 0.091 | 0.045 | 0.024 | 0.048 | 0.05 | 0.03 | 0.024 | 0.12 |
| 100 | 0.07 | 0.05 | 0.05 | 0.108 | 0.081 | 0.052 | 0.04 | 0.052 | 0.061 | 0.061 | 0.06 | 0.101 |
| 101 | 0.071 | 0.03 | 0.024 | 0.05 | 0.084 | 0.05 | 0.061 | 0.033 | 0.01 | 0.024 | 0.03 | 0.057 |
| 102 | 0.048 | 0.042 | 0.057 | 0.066 | 0.11 | 0.04 | 0.03 | 0.02 | 0.089 | 0.066 | 0.071 | 0.091 |
| 103 | 0.04 | 0.04 | 0.052 | 0.1 | 0.075 | 0.05 | 0.04 | 0.02 | 0.042 | 0.08 | 0.09 | 0.09 |
| 104 | 0.052 | 0.042 | 0.061 | 0.1 | 0.081 | 0.09 | 0.03 | 0.04 | 0.06 | 0.101 | 0.061 | 0.081 |
| 105 | 0.033 | 0.052 | 0.06 | 0.071 | 0.08 | 0.08 | 0.06 | 0.02 | 0.052 | 0.061 | 0.072 | 0.05 |
| 106 | 0.05 | 0.05 | 0.03 | 0.11 | 0.111 | 0.101 | 0.05 | 0.05 | 0.042 | 0.07 | 0.052 | 0.071 |
| 107 | 0.05 | 0.052 | 0.04 | 0.07 | 0.09 | 0.05 | 0.07 | 0.042 | 0.02 | 0.061 | 0.098 | 0.042 |
| 108 | 0.04 | 0.042 | 0.06 | 0.16 | 0.06 | 0.04 | 0.033 | 0.04 | 0.024 | 0.05 | 0.113 | 0.07 |
| 109 | 0.05 | 0.033 | 0.052 | 0.1 | 0.081 | 0.042 | 0.048 | 0.048 | 0.061 | 0.071 | 0.05 | 0.06 |
| 110 | 0.078 | 0.042 | 0.033 | 0.14 | 0.07 | 0.05 | 0.066 | 0.033 | 0.07 | 0.06 | 0.071 | 0.05 |
| 111 | 0.08 | 0.071 | 0.071 | 0.101 | 0.113 | 0.06 | 0.061 | 0.052 | 0.052 | 0.061 | 0.05 | 0.108 |
| 112 | 0.06 | 0.033 | 0.052 | 0.08 | 0.121 | 0.091 | 0.033 | 0.06 | 0.052 | 0.03 | 0.04 | 0.17 |
| 113 | 0.071 | 0.061 | 0.052 | 0.066 | 0.11 | 0.06 | 0.061 | 0.061 | 0.042 | 0.1 | 0.05 | 0.06 |
| 114 | 0.033 | 0.08 | 0.052 | 0.07 | 0.1 | 0.04 | 0.06 | 0.02 | 0.081 | 0.052 | 0.07 | 0.108 |
| 115 | 0.024 | 0.061 | 0.048 | 0.061 | 0.08 | 0.03 | 0.04 | 0.02 | 0.04 | 0.123 | 0.057 | 0.052 |
| 116 | 0.061 | 0.061 | 0.042 | 0.08 | 0.1 | 0.052 | 0.084 | 0.04 | 0.03 | 0.042 | 0.06 | 0.071 |
| 117 | 0.057 | 0.07 | 0.071 | 0.08 | 0.071 | 0.06 | 0.04 | 0.082 | 0.05 | 0.066 | 0.06 | 0.08 |
| 118 | 0.07 | 0.06 | 0.05 | 0.1 | 0.101 | 0.081 | 0.033 | 0.033 | 0.061 | 0.061 | 0.06 | 0.05 |
| 119 | 0.07 | 0.042 | 0.04 | 0.03 | 0.141 | 0.091 | 0.052 | 0.03 | 0.05 | 0.057 | 0.06 | 0.13 |
| 120 | 0.04 | 0.033 | 0.048 | 0.075 | 0.103 | 0.11 | 0.052 | 0.061 | 0.06 | 0.075 | 0.07 | 0.05 |
| 121 | 0.03 | 0.07 | 0.05 | 0.061 | 0.081 | 0.081 | 0.06 | 0.07 | 0.05 | 0.1 | 0.042 | 0.07 |
| 122 | 0.042 | 0.072 | 0.071 | 0.06 | 0.08 | 0.084 | 0.07 | 0.071 | 0.06 | 0.091 | 0.061 | 0.06 |
| 123 | 0.052 | 0.05 | 0.02 | 0.09 | 0.103 | 0.06 | 0.07 | 0.057 | 0.042 | 0.091 | 0.081 | 0.06 |
| 124 | 0.033 | 0.048 | 0.048 | 0.121 | 0.081 | 0.061 | 0.061 | 0.02 | 0.052 | 0.081 | 0.061 | 0.061 |
| 125 | 0.033 | 0.06 | 0.04 | 0.075 | 0.14 | 0.033 | 0.05 | 0.057 | 0.04 | 0.05 | 0.113 | 0.07 |
| 126 | 0.04 | 0.03 | 0.057 | 0.075 | 0.1 | 0.061 | 0.06 | 0.01 | 0.033 | 0.141 | 0.05 | 0.081 |
| 127 | 0.061 | 0.02 | 0.04 | 0.08 | 0.091 | 0.091 | 0.033 | 0.03 | 0.033 | 0.081 | 0.052 | 0.101 |
| 128 | 0.05 | 0.042 | 0.024 | 0.07 | 0.06 | 0.075 | 0.08 | 0.075 | 0.06 | 0.06 | 0.05 | 0.07 |
| 129 | 0.033 | 0.052 | 0.052 | 0.08 | 0.143 | 0.084 | 0.01 | 0.081 | 0.057 | 0.057 | 0.071 | 0.08 |
| 130 | 0.04 | 0.03 | 0.05 | 0.081 | 0.1 | 0.123 | 0.04 | 0.05 | 0.066 | 0.061 | 0.033 | 0.08 |
| 131 | 0.06 | 0.071 | 0.052 | 0.13 | 0.08 | 0.1 | 0.04 | 0.03 | 0.02 | 0.05 | 0.05 | 0.075 |
| 132 | 0.024 | 0.052 | 0.052 | 0.09 | 0.1 | 0.08 | 0.042 | 0.057 | 0.071 | 0.04 | 0.061 | 0.061 |
| 133 | 0.04 | 0.066 | 0.061 | 0.066 | 0.05 | 0.081 | 0.075 | 0.061 | 0.075 | 0.1 | 0.04 | 0.081 |
| 134 | 0.04 | 0.04 | 0.05 | 0.061 | 0.12 | 0.066 | 0.04 | 0.04 | 0.04 | 0.06 | 0.01 | 0.103 |
| 135 | 0.03 | 0.05 | 0.052 | 0.07 | 0.03 | 0.103 | 0.03 | 0.067 | 0.061 | 0.033 | 0.042 | 0.057 |
| 136 | 0.052 | 0.03 | 0.04 | 0.042 | 0.05 | 0.057 | 0.052 | 0.04 | 0.06 | 0.061 | 0.071 | 0.1 |
| 137 | 0.033 | 0.061 | 0.042 | 0.04 | 0.08 | 0.06 | 0.04 | 0.04 | 0.04 | 0.071 | 0.024 | 0.064 |
| 138 | 0.03 | 0.06 | 0.033 | 0.091 | 0.094 | 0.101 | 0.04 | 0.04 | 0.061 | 0.052 | 0.024 | 0.1 |
| 139 | 0.04 | 0.061 | 0.04 | 0.052 | 0.084 | 0.04 | 0.033 | 0.04 | 0.061 | 0.06 | 0.05 | 0.072 |
| 140 | 0.04 | 0.033 | 0.04 | 0.061 | 0.061 | 0.09 | 0.02 | 0.061 | 0.042 | 0.07 | 0.052 | 0.07 |
| 141 | 0.05 | 0.061 | 0.05 | 0.061 | 0.172 | 0.071 | 0.052 | 0.052 | 0.066 | 0.05 | 0.028 | 0.09 |
| 142 | 0.02 | 0.06 | 0.042 | 0.071 | 0.09 | 0.07 | 0.01 | 0.03 | 0.081 | 0.061 | 0.06 | 0.075 |
| 143 | 0.057 | 0.071 | 0.075 | 0.066 | 0.075 | 0.091 | 0.033 | 0.061 | 0.08 | 0.061 | 0.02 | 0.08 |
| 144 | 0.03 | 0.081 | 0.061 | 0.05 | 0.05 | 0.128 | 0.042 | 0.091 | 0.04 | 0.1 | 0.101 | 0.057 |
| 145 | 0.04 | 0.052 | 0.075 | 0.07 | 0.07 | 0.081 | 0.066 | 0.04 | 0.052 | 0.1 | 0.06 | 0.08 |
| 146 | 0.1 | 0.08 | 0.061 | 0.101 | 0.05 | 0.06 | 0.08 | 0.04 | 0.02 | 0.141 | 0.081 | 0.08 |
| 147 | 0.061 | 0.05 | 0.064 | 0.1 | 0.091 | 0.09 | 0.084 | 0.04 | 0.057 | 0.071 | 0.05 | 0.094 |
| 148 | 0.066 | 0.05 | 0.052 | 0.081 | 0.08 | 0.08 | 0.05 | 0.05 | 0.06 | 0.081 | 0.061 | 0.075 |
| 149 | 0.03 | 0.06 | 0.05 | 0.1 | 0.117 | 0.081 | 0.061 | 0.02 | 0.04 | 0.091 | 0.07 | 0.042 |
| 150 | 0.01 | 0.042 | 0.057 | 0.071 | 0.111 | 0.1 | 0.06 | 0.04 | 0.061 | 0.08 | 0.081 | 0.08 |
| 151 | 0.05 | 0.08 | 0.024 | 0.06 | 0.19 | 0.12 | 0.075 | 0.01 | 0.033 | 0.071 | 0.1 | 0.06 |
| 152 | 0.05 | 0.057 | 0.061 | 0.11 | 0.133 | 0.061 | 0.04 | 0.06 | 0.066 | 0.089 | 0.091 | 0.131 |
| 153 | 0.03 | 0.06 | 0.02 | 0.09 | 0.091 | 0.04 | 0.052 | 0.066 | 0.06 | 0.098 | 0.09 | 0.084 |
| 154 | 0.042 | 0.06 | 0.052 | 0.07 | 0.08 | 0.08 | 0.042 | 0.05 | 0.066 | 0.103 | 0.094 | 0.072 |
| 155 | 0.05 | 0.066 | 0.06 | 0.121 | 0.07 | 0.057 | 0.042 | 0.057 | 0.121 | 0.075 | 0.052 | 0.08 |
| 156 | 0.052 | 0.04 | 0.052 | 0.1 | 0.111 | 0.117 | 0.1 | 0.057 | 0.072 | 0.071 | 0.03 | 0.071 |
| 157 | 0.024 | 0.061 | 0.05 | 0.042 | 0.091 | 0.091 | 0.061 | 0.06 | 0.073 | 0.084 | 0.05 | 0.061 |
| 158 | 0.05 | 0.081 | 0.06 | 0.05 | 0.103 | 0.081 | 0.05 | 0.06 | 0.042 | 0.08 | 0.02 | 0.094 |
| 159 | 0.048 | 0.03 | 0.04 | 0.108 | 0.161 | 0.084 | 0.081 | 0.057 | 0.066 | 0.07 | 0.052 | 0.101 |
| 160 | 0.04 | 0.03 | 0.04 | 0.08 | 0.121 | 0.042 | 0.072 | 0.066 | 0.06 | 0.091 | 0.033 | 0.152 |
| 161 | 0.02 | 0.03 | 0.04 | 0.05 | 0.111 | 0.05 | 0.04 | 0.07 | 0.07 | 0.081 | 0.048 | 0.084 |
| 162 | 0.042 | 0.02 | 0.05 | 0.09 | 0.04 | 0.11 | 0.033 | 0.057 | 0.052 | 0.084 | 0.071 | 0.04 |
| 163 | 0.033 | 0.06 | 0.05 | 0.07 | 0.061 | 0.07 | 0.061 | 0.066 | 0.033 | 0.108 | 0.04 | 0.081 |
| 164 | 0.033 | 0.033 | 0.033 | 0.101 | 0.081 | 0.075 | 0.04 | 0.061 | 0.061 | 0.03 | 0.024 | 0.075 |
| 165 | 0.033 | 0.04 | 0.04 | 0.084 | 0.061 | 0.071 | 0.06 | 0.08 | 0.07 | 0.04 | 0.03 | 0.07 |
| 166 | 0.05 | 0.05 | 0.03 | 0.09 | 0.133 | 0.081 | 0.057 | 0.06 | 0.052 | 0.03 | 0.042 | 0.111 |
| 167 | 0.05 | 0.042 | 0.06 | 0.113 | 0.131 | 0.091 | 0.042 | 0.06 | 0.081 | 0.075 | 0.04 | 0.06 |
| 168 | 0.04 | 0.05 | 0.071 | 0.17 | 0.105 | 0.061 | 0.061 | 0.04 | 0.052 | 0.02 | 0.075 | 0.07 |
| 169 | 0.042 | 0.057 | 0.06 | 0.052 | 0.081 | 0.07 | 0.048 | 0.05 | 0.084 | 0.04 | 0.033 | 0.08 |
| 170 | 0.02 | 0.04 | 0.042 | 0.081 | 0.071 | 0.081 | 0.04 | 0.064 | 0.1 | 0.05 | 0.07 | 0.1 |
| 171 | 0.02 | 0.04 | 0.05 | 0.05 | 0.071 | 0.066 | 0.091 | 0.052 | 0.05 | 0.081 | 0.05 | 0.06 |
| 172 | 0.05 | 0.071 | 0.042 | 0.081 | 0.071 | 0.04 | 0.033 | 0.042 | 0.04 | 0.024 | 0.06 | 0.081 |
| 173 | 0.04 | 0.061 | 0.057 | 0.141 | 0.091 | 0.061 | 0.08 | 0.067 | 0.057 | 0.04 | 0.06 | 0.061 |
| 174 | 0.033 | 0.04 | 0.052 | 0.09 | 0.07 | 0.06 | 0.06 | 0.048 | 0.07 | 0.081 | 0.052 | 0.05 |
| 175 | 0.033 | 0.061 | 0.06 | 0.081 | 0.04 | 0.066 | 0.07 | 0.04 | 0.07 | 0.071 | 0.057 | 0.07 |
| 176 | 0.042 | 0.06 | 0.03 | 0.098 | 0.094 | 0.117 | 0.05 | 0.048 | 0.06 | 0.04 | 0.04 | 0.042 |
| 177 | 0.048 | 0.042 | 0.04 | 0.08 | 0.061 | 0.08 | 0.05 | 0.033 | 0.042 | 0.07 | 0.05 | 0.061 |
| 178 | 0.033 | 0.071 | 0.04 | 0.113 | 0.06 | 0.07 | 0.066 | 0.081 | 0.042 | 0.07 | 0.071 | 0.071 |
| 179 | 0.042 | 0.05 | 0.04 | 0.084 | 0.121 | 0.061 | 0.06 | 0.075 | 0.05 | 0.06 | 0.07 | 0.08 |
| 180 | 0.05 | 0.033 | 0.04 | 0.09 | 0.071 | 0.11 | 0.064 | 0.052 | 0.075 | 0.066 | 0.06 | 0.052 |
| 181 | 0.03 | 0.05 | 0.052 | 0.03 | 0.133 | 0.05 | 0.02 | 0.071 | 0.057 | 0.042 | 0.066 | 0.084 |
| 182 | 0.05 | 0.05 | 0.052 | 0.061 | 0.108 | 0.08 | 0.033 | 0.05 | 0.04 | 0.07 | 0.042 | 0.09 |
| 183 | 0.05 | 0.052 | 0.05 | 0.105 | 0.052 | 0.05 | 0.05 | 0.075 | 0.066 | 0.1 | 0.05 | 0.03 |
| 184 | 0.052 | 0.05 | 0.05 | 0.071 | 0.075 | 0.05 | 0.024 | 0.04 | 0.064 | 0.052 | 0.042 | 0.04 |
| 185 | 0.042 | 0.057 | 0.04 | 0.1 | 0.07 | 0.091 | 0.07 | 0.101 | 0.071 | 0.061 | 0.113 | 0.06 |
| 186 | 0.061 | 0.064 | 0.04 | 0.121 | 0.091 | 0.08 | 0.066 | 0.06 | 0.089 | 0.091 | 0.094 | 0.111 |
| 187 | 0.048 | 0.048 | 0.03 | 0.09 | 0.111 | 0.057 | 0.081 | 0.091 | 0.081 | 0.066 | 0.075 | 0.042 |
| 188 | 0.04 | 0.04 | 0.061 | 0.052 | 0.048 | 0.1 | 0.03 | 0.042 | 0.071 | 0.071 | 0.101 | 0.131 |
| 189 | 0.04 | 0.066 | 0.04 | 0.04 | 0.081 | 0.042 | 0.03 | 0.07 | 0.04 | 0.052 | 0.084 | 0.11 |
| 190 | 0.042 | 0.06 | 0.052 | 0.091 | 0.071 | 0.091 | 0.024 | 0.033 | 0.06 | 0.096 | 0.033 | 0.071 |
| 191 | 0.033 | 0.05 | 0.052 | 0.08 | 0.07 | 0.152 | 0.05 | 0.02 | 0.08 | 0.112 | 0.09 | 0.081 |
| 192 | 0.042 | 0.03 | 0.05 | 0.024 | 0.061 | 0.03 | 0.024 | 0.066 | 0.071 | 0.05 | 0.108 | 0.07 |
| 193 | 0.03 | 0.052 | 0.075 | 0.05 | 0.03 | 0.101 | 0.05 | 0.075 | 0.052 | 0.033 | 0.09 | 0.071 |
| 194 | 0.033 | 0.03 | 0.04 | 0.071 | 0.07 | 0.081 | 0.033 | 0.05 | 0.03 | 0.075 | 0.101 | 0.101 |
| 195 | 0.03 | 0.09 | 0.052 | 0.06 | 0.13 | 0.084 | 0.06 | 0.052 | 0.05 | 0.033 | 0.081 | 0.103 |
| 196 | 0.042 | 0.06 | 0.06 | 0.066 | 0.151 | 0.061 | 0.09 | 0.03 | 0.061 | 0.075 | 0.06 | 0.133 |
| 197 | 0.042 | 0.05 | 0.03 | 0.084 | 0.094 | 0.057 | 0.03 | 0.01 | 0.05 | 0.048 | 0.04 | 0.071 |
| 198 | 0.03 | 0.042 | 0.033 | 0.07 | 0.094 | 0.075 | 0.06 | 0.084 | 0.01 | 0.072 | 0.081 | 0.07 |
| 199 | 0.033 | 0.052 | 0.066 | 0.06 | 0.098 | 0.066 | 0.03 | 0.08 | 0.06 | 0.072 | 0.146 | 0.094 |
| 200 | 0.042 | 0.052 | 0.03 | 0.06 | 0.06 | 0.081 | 0.042 | 0.081 | 0.057 | 0.084 | 0.091 | 0.081 |
| 201 | 0.033 | 0.04 | 0.071 | 0.061 | 0.06 | 0.061 | 0.05 | 0.08 | 0.073 | 0.07 | 0.071 | 0.084 |
| 202 | 0.05 | 0.052 | 0.03 | 0.052 | 0.08 | 0.066 | 0.04 | 0.052 | 0.06 | 0.084 | 0.061 | 0.084 |
| 203 | 0.042 | 0.05 | 0.02 | 0.07 | 0.08 | 0.089 | 0.071 | 0.09 | 0.03 | 0.071 | 0.075 | 0.05 |
| 204 | 0.05 | 0.04 | 0.07 | 0.081 | 0.052 | 0.072 | 0.04 | 0.07 | 0.101 | 0.07 | 0.112 | 0.12 |
| 205 | 0.05 | 0.06 | 0.04 | 0.042 | 0.08 | 0.066 | 0.042 | 0.06 | 0.052 | 0.081 | 0.066 | 0.08 |
| 206 | 0.06 | 0.02 | 0.057 | 0.057 | 0.061 | 0.084 | 0.04 | 0.05 | 0.061 | 0.081 | 0.123 | 0.066 |
| 207 | 0.061 | 0.061 | 0.05 | 0.066 | 0.089 | 0.057 | 0.04 | 0.05 | 0.052 | 0.07 | 0.071 | 0.07 |
| 208 | 0.02 | 0.02 | 0.05 | 0.052 | 0.091 | 0.096 | 0.06 | 0.061 | 0.04 | 0.08 | 0.141 | 0.103 |
| 209 | 0.03 | 0.05 | 0.03 | 0.08 | 0.04 | 0.064 | 0.08 | 0.075 | 0.071 | 0.121 | 0.091 | 0.06 |
| 210 | 0.033 | 0.061 | 0.033 | 0.084 | 0.14 | 0.057 | 0.07 | 0.03 | 0.05 | 0.071 | 0.101 | 0.094 |
| 211 | 0.061 | 0.052 | 0.03 | 0.052 | 0.12 | 0.05 | 0.03 | 0.042 | 0.07 | 0.061 | 0.081 | 0.084 |
| 212 | 0.03 | 0.071 | 0.04 | 0.126 | 0.081 | 0.081 | 0.03 | 0.11 | 0.08 | 0.04 | 0.052 | 0.113 |
| 213 | 0.05 | 0.04 | 0.05 | 0.061 | 0.101 | 0.03 | 0.02 | 0.04 | 0.111 | 0.11 | 0.071 | 0.143 |
| 214 | 0.03 | 0.04 | 0.052 | 0.061 | 0.08 | 0.052 | 0.02 | 0.052 | 0.02 | 0.052 | 0.075 | 0.071 |
| 215 | 0.057 | 0.061 | 0.03 | 0.113 | 0.08 | 0.08 | 0.07 | 0.02 | 0.04 | 0.04 | 0.09 | 0.12 |
| 216 | 0.05 | 0.03 | 0.02 | 0.04 | 0.05 | 0.04 | 0.03 | 0.048 | 0.04 | 0.061 | 0.08 | 0.103 |
| 217 | 0.02 | 0.071 | 0.03 | 0.09 | 0.03 | 0.06 | 0.042 | 0.052 | 0.052 | 0.052 | 0.081 | 0.09 |
| 218 | 0.033 | 0.03 | 0.057 | 0.081 | 0.081 | 0.03 | 0.01 | 0.06 | 0.052 | 0.05 | 0.121 | 0.121 |
| 219 | 0.04 | 0.057 | 0.064 | 0.04 | 0.052 | 0.1 | 0.06 | 0.061 | 0.091 | 0.057 | 0.06 | 0.162 |
| 220 | 0.03 | 0.05 | 0.071 | 0.071 | 0.03 | 0.08 | 0.04 | 0.11 | 0.03 | 0.042 | 0.141 | 0.131 |
| 221 | 0.042 | 0.04 | 0.061 | 0.05 | 0.057 | 0.103 | 0.04 | 0.04 | 0.057 | 0.057 | 0.06 | 0.121 |
| 222 | 0.052 | 0.05 | 0.052 | 0.1 | 0.07 | 0.091 | 0.052 | 0.052 | 0.05 | 0.084 | 0.071 | 0.07 |
| 223 | 0.052 | 0.052 | 0.05 | 0.09 | 0.081 | 0.052 | 0.06 | 0.06 | 0.1 | 0.08 | 0.071 | 0.081 |
| 224 | 0.05 | 0.02 | 0.05 | 0.1 | 0.1 | 0.13 | 0.04 | 0.08 | 0.05 | 0.084 | 0.066 | 0.141 |
| 225 | 0.04 | 0.03 | 0.033 | 0.101 | 0.08 | 0.105 | 0.05 | 0.08 | 0.061 | 0.089 | 0.101 | 0.071 |
| 226 | 0.03 | 0.03 | 0.042 | 0.094 | 0.088 | 0.066 | 0.061 | 0.042 | 0.042 | 0.061 | 0.1 | 0.081 |
| 227 | 0.061 | 0.04 | 0.052 | 0.091 | 0.057 | 0.1 | 0.02 | 0.05 | 0.06 | 0.072 | 0.09 | 0.14 |
| 228 | 0.03 | 0.03 | 0.03 | 0.089 | 0.052 | 0.061 | 0.03 | 0.052 | 0.071 | 0.131 | 0.09 | 0.081 |
| 229 | 0.052 | 0.042 | 0.06 | 0.09 | 0.066 | 0.075 | 0.06 | 0.05 | 0.071 | 0.033 | 0.094 | 0.111 |
| 230 | 0.05 | 0.04 | 0.042 | 0.081 | 0.057 | 0.094 | 0.04 | 0.05 | 0.061 | 0.057 | 0.04 | 0.067 |
| 231 | 0.052 | 0.02 | 0.03 | 0.05 | 0.033 | 0.084 | 0.06 | 0.02 | 0.103 | 0.024 | 0.14 | 0.091 |
| 232 | 0.03 | 0.052 | 0.03 | 0.061 | 0.075 | 0.08 | 0.052 | 0.01 | 0.033 | 0.05 | 0.121 | 0.14 |
| 233 | 0.052 | 0.02 | 0.06 | 0.101 | 0.096 | 0.06 | 0.07 | 0.05 | 0.04 | 0.024 | 0.121 | 0.09 |
| 234 | 0.033 | 0.075 | 0.042 | 0.081 | 0.091 | 0.08 | 0.052 | 0.071 | 0.03 | 0.07 | 0.048 | 0.091 |
| 235 | 0.03 | 0.042 | 0.03 | 0.071 | 0.06 | 0.06 | 0.03 | 0.061 | 0.08 | 0.05 | 0.113 | 0.101 |
| 236 | 0.052 | 0.06 | 0.05 | 0.052 | 0.07 | 0.071 | 0.052 | 0.045 | 0.052 | 0.07 | 0.11 | 0.162 |
| 237 | 0.07 | 0.052 | 0.04 | 0.09 | 0.06 | 0.101 | 0.033 | 0.042 | 0.04 | 0.131 | 0.071 | 0.07 |
| 238 | 0.04 | 0.04 | 0.024 | 0.052 | 0.091 | 0.06 | 0.057 | 0.04 | 0.024 | 0.06 | 0.08 | 0.143 |
| 239 | 0.057 | 0.05 | 0.066 | 0.071 | 0.12 | 0.06 | 0.061 | 0.1 | 0.061 | 0.09 | 0.061 | 0.08 |
| 240 | 0.071 | 0.04 | 0.01 | 0.066 | 0.101 | 0.052 | 0.02 | 0.017 | 0.048 | 0.071 | 0.07 | 0.151 |
| 241 | 0.066 | 0.03 | 0.042 | 0.061 | 0.052 | 0.071 | 0.05 | 0.075 | 0.064 | 0.101 | 0.091 | 0.071 |
| 242 | 0.033 | 0.05 | 0.04 | 0.066 | 0.101 | 0.05 | 0.03 | 0.05 | 0.07 | 0.081 | 0.081 | 0.111 |
| 243 | 0.042 | 0.03 | 0.042 | 0.091 | 0.066 | 0.07 | 0.042 | 0.06 | 0.06 | 0.111 | 0.101 | 0.094 |
| 244 | 0.033 | 0.04 | 0.02 | 0.06 | 0.09 | 0.07 | 0.071 | 0.052 | 0.04 | 0.042 | 0.08 | 0.15 |
| 245 | 0.03 | 0.03 | 0.03 | 0.072 | 0.04 | 0.075 | 0.052 | 0.066 | 0.03 | 0.06 | 0.169 | 0.06 |
| 246 | 0.04 | 0.042 | 0.05 | 0.071 | 0.083 | 0.08 | 0.09 | 0.042 | 0.06 | 0.048 | 0.101 | 0.042 |
| 247 | 0.042 | 0.052 | 0.06 | 0.071 | 0.071 | 0.07 | 0.081 | 0.033 | 0.07 | 0.04 | 0.17 | 0.091 |
| 248 | 0.04 | 0.05 | 0.05 | 0.061 | 0.04 | 0.071 | 0.052 | 0.04 | 0.033 | 0.05 | 0.098 | 0.09 |
| 249 | 0.048 | 0.061 | 0.072 | 0.06 | 0.05 | 0.078 | 0.08 | 0.048 | 0.048 | 0.061 | 0.1 | 0.05 |
| 250 | 0.048 | 0.071 | 0.072 | 0.11 | 0.06 | 0.09 | 0.06 | 0.03 | 0.05 | 0.061 | 0.12 | 0.07 |
| Mean | 0.041644 | 0.048516 | 0.045596 | 0.075524 | 0.08222 | 0.07284 | 0.048512 | 0.049028 | 0.053868 | 0.068308 | 0.069316 | 0.083024 |
| SD | 0.0145 | 0.015029 | 0.013368 | 0.024143 | 0.028821 | 0.023843 | 0.017391 | 0.019727 | 0.01986 | 0.023365 | 0.026394 | 0.027543 |

|  | Day 6 | | | | | | Day 7 | | | | | |
| --- | --- | --- | --- | --- | --- | --- | --- | --- | --- | --- | --- | --- |
| OCT images | Control | | | Infected | | | Control | | | Infected | | |
|  | 1 | 2 | 3 | 1 | 2 | 3 | 1 | 2 | 3 | 1 | 2 | 3 |
| 1 | 0.071 | 0.042 | 0.042 | 0.07 | 0.052 | 0.03 | 0.048 | 0.033 | 0.06 | 0.055 | 0.06 | 0.066 |
| 2 | 0.042 | 0.033 | 0.04 | 0.08 | 0.09 | 0.03 | 0.01 | 0.03 | 0.06 | 0.071 | 0.108 | 0.071 |
| 3 | 0.06 | 0.05 | 0.05 | 0.05 | 0.042 | 0.091 | 0.05 | 0.02 | 0.052 | 0.06 | 0.071 | 0.073 |
| 4 | 0.04 | 0.02 | 0.03 | 0.07 | 0.052 | 0.07 | 0.07 | 0.052 | 0.04 | 0.044 | 0.09 | 0.061 |
| 5 | 0.04 | 0.05 | 0.02 | 0.05 | 0.075 | 0.07 | 0.02 | 0.01 | 0.033 | 0.033 | 0.1 | 0.093 |
| 6 | 0.06 | 0.071 | 0.05 | 0.04 | 0.061 | 0.042 | 0.033 | 0.052 | 0.066 | 0.093 | 0.098 | 0.077 |
| 7 | 0.05 | 0.081 | 0.061 | 0.06 | 0.05 | 0.02 | 0.04 | 0.02 | 0.05 | 0.077 | 0.07 | 0.057 |
| 8 | 0.06 | 0.05 | 0.081 | 0.06 | 0.1 | 0.061 | 0.03 | 0.048 | 0.03 | 0.049 | 0.103 | 0.077 |
| 9 | 0.02 | 0.07 | 0.061 | 0.03 | 0.07 | 0.042 | 0.05 | 0.03 | 0.04 | 0.057 | 0.07 | 0.088 |
| 10 | 0.042 | 0.06 | 0.071 | 0.052 | 0.071 | 0.071 | 0.061 | 0.03 | 0.057 | 0.038 | 0.05 | 0.149 |
| 11 | 0.04 | 0.071 | 0.05 | 0.042 | 0.07 | 0.061 | 0.042 | 0.061 | 0.048 | 0.049 | 0.084 | 0.082 |
| 12 | 0.03 | 0.05 | 0.052 | 0.057 | 0.052 | 0.05 | 0.05 | 0.04 | 0.04 | 0.072 | 0.113 | 0.054 |
| 13 | 0.05 | 0.06 | 0.07 | 0.03 | 0.08 | 0.05 | 0.04 | 0.05 | 0.02 | 0.066 | 0.06 | 0.11 |
| 14 | 0.057 | 0.01 | 0.1 | 0.04 | 0.05 | 0.03 | 0.05 | 0.01 | 0.03 | 0.041 | 0.05 | 0.13 |
| 15 | 0.03 | 0.024 | 0.081 | 0.03 | 0.06 | 0.06 | 0.042 | 0.02 | 0.03 | 0.077 | 0.07 | 0.121 |
| 16 | 0.033 | 0.03 | 0.04 | 0.061 | 0.07 | 0.105 | 0.071 | 0.02 | 0.03 | 0.105 | 0.11 | 0.1 |
| 17 | 0.06 | 0.052 | 0.052 | 0.05 | 0.03 | 0.089 | 0.02 | 0.042 | 0.03 | 0.093 | 0.07 | 0.091 |
| 18 | 0.052 | 0.03 | 0.066 | 0.084 | 0.05 | 0.057 | 0.033 | 0.04 | 0.066 | 0.11 | 0.16 | 0.071 |
| 19 | 0.11 | 0.05 | 0.02 | 0.05 | 0.07 | 0.033 | 0.052 | 0.05 | 0.05 | 0.093 | 0.042 | 0.049 |
| 20 | 0.048 | 0.017 | 0.02 | 0.05 | 0.131 | 0.101 | 0.052 | 0.06 | 0.064 | 0.066 | 0.071 | 0.134 |
| 21 | 0.04 | 0.052 | 0.06 | 0.05 | 0.05 | 0.11 | 0.033 | 0.042 | 0.052 | 0.078 | 0.159 | 0.049 |
| 22 | 0.04 | 0.052 | 0.05 | 0.04 | 0.05 | 0.09 | 0.05 | 0.02 | 0.06 | 0.083 | 0.091 | 0.044 |
| 23 | 0.02 | 0.05 | 0.061 | 0.1 | 0.12 | 0.101 | 0.042 | 0.091 | 0.052 | 0.055 | 0.131 | 0.066 |
| 24 | 0.06 | 0.03 | 0.06 | 0.08 | 0.06 | 0.09 | 0.06 | 0.05 | 0.06 | 0.075 | 0.09 | 0.096 |
| 25 | 0.03 | 0.04 | 0.05 | 0.08 | 0.06 | 0.09 | 0.02 | 0.05 | 0.03 | 0.072 | 0.11 | 0.122 |
| 26 | 0.03 | 0.04 | 0.042 | 0.052 | 0.061 | 0.066 | 0.03 | 0.04 | 0.02 | 0.088 | 0.033 | 0.073 |
| 27 | 0.052 | 0.061 | 0.07 | 0.07 | 0.05 | 0.061 | 0.06 | 0.024 | 0.061 | 0.091 | 0.075 | 0.121 |
| 28 | 0.04 | 0.03 | 0.048 | 0.08 | 0.06 | 0.084 | 0.05 | 0.061 | 0.05 | 0.072 | 0.061 | 0.099 |
| 29 | 0.061 | 0.02 | 0.057 | 0.05 | 0.042 | 0.05 | 0.05 | 0.05 | 0.057 | 0.078 | 0.111 | 0.094 |
| 30 | 0.06 | 0.02 | 0.052 | 0.03 | 0.042 | 0.08 | 0.048 | 0.061 | 0.04 | 0.055 | 0.094 | 0.104 |
| 31 | 0.081 | 0.05 | 0.033 | 0.04 | 0.048 | 0.042 | 0.04 | 0.04 | 0.07 | 0.062 | 0.04 | 0.049 |
| 32 | 0.04 | 0.061 | 0.03 | 0.081 | 0.042 | 0.05 | 0.042 | 0.02 | 0.05 | 0.055 | 0.061 | 0.061 |
| 33 | 0.05 | 0.05 | 0.02 | 0.094 | 0.04 | 0.081 | 0.08 | 0.05 | 0.09 | 0.073 | 0.084 | 0.049 |
| 34 | 0.042 | 0.071 | 0.03 | 0.089 | 0.08 | 0.09 | 0.07 | 0.033 | 0.03 | 0.066 | 0.094 | 0.073 |
| 35 | 0.02 | 0.05 | 0.03 | 0.084 | 0.06 | 0.121 | 0.061 | 0.042 | 0.03 | 0.061 | 0.06 | 0.11 |
| 36 | 0.061 | 0.05 | 0.05 | 0.052 | 0.08 | 0.103 | 0.052 | 0.061 | 0.03 | 0.104 | 0.07 | 0.122 |
| 37 | 0.061 | 0.052 | 0.048 | 0.111 | 0.01 | 0.06 | 0.02 | 0.02 | 0.05 | 0.075 | 0.08 | 0.055 |
| 38 | 0.071 | 0.02 | 0.04 | 0.066 | 0.061 | 0.05 | 0.05 | 0.02 | 0.01 | 0.089 | 0.052 | 0.027 |
| 39 | 0.042 | 0.08 | 0.05 | 0.081 | 0.081 | 0.042 | 0.02 | 0.033 | 0.01 | 0.082 | 0.071 | 0.099 |
| 40 | 0.052 | 0.061 | 0.05 | 0.042 | 0.094 | 0.02 | 0.06 | 0.06 | 0.02 | 0.111 | 0.098 | 0.038 |
| 41 | 0.052 | 0.04 | 0.07 | 0.02 | 0.07 | 0.05 | 0.07 | 0.033 | 0.033 | 0.084 | 0.057 | 0.071 |
| 42 | 0.06 | 0.042 | 0.03 | 0.03 | 0.05 | 0.13 | 0.052 | 0.05 | 0.024 | 0.11 | 0.091 | 0.077 |
| 43 | 0.07 | 0.05 | 0.061 | 0.12 | 0.03 | 0.09 | 0.04 | 0.042 | 0.07 | 0.088 | 0.071 | 0.061 |
| 44 | 0.04 | 0.05 | 0.04 | 0.03 | 0.03 | 0.101 | 0.03 | 0.04 | 0.02 | 0.115 | 0.071 | 0.08 |
| 45 | 0.072 | 0.052 | 0.03 | 0.06 | 0.061 | 0.06 | 0.02 | 0.04 | 0.07 | 0.082 | 0.06 | 0.061 |
| 46 | 0.04 | 0.05 | 0.02 | 0.108 | 0.133 | 0.071 | 0.02 | 0.03 | 0.07 | 0.066 | 0.081 | 0.033 |
| 47 | 0.071 | 0.061 | 0.061 | 0.101 | 0.091 | 0.098 | 0.017 | 0.04 | 0.04 | 0.044 | 0.081 | 0.094 |
| 48 | 0.05 | 0.071 | 0.04 | 0.094 | 0.091 | 0.081 | 0.052 | 0.04 | 0.07 | 0.083 | 0.071 | 0.104 |
| 49 | 0.071 | 0.05 | 0.03 | 0.14 | 0.06 | 0.071 | 0.04 | 0.061 | 0.071 | 0.11 | 0.1 | 0.052 |
| 50 | 0.042 | 0.061 | 0.02 | 0.04 | 0.06 | 0.033 | 0.081 | 0.01 | 0.04 | 0.066 | 0.08 | 0.028 |
| 51 | 0.05 | 0.06 | 0.02 | 0.081 | 0.04 | 0.061 | 0.04 | 0.03 | 0.05 | 0.11 | 0.091 | 0.06 |
| 52 | 0.02 | 0.061 | 0.061 | 0.1 | 0.05 | 0.06 | 0.02 | 0.042 | 0.04 | 0.06 | 0.081 | 0.049 |
| 53 | 0.071 | 0.05 | 0.04 | 0.101 | 0.033 | 0.07 | 0.03 | 0.03 | 0.05 | 0.05 | 0.07 | 0.049 |
| 54 | 0.03 | 0.04 | 0.04 | 0.131 | 0.07 | 0.07 | 0.05 | 0.04 | 0.052 | 0.055 | 0.05 | 0.038 |
| 55 | 0.01 | 0.057 | 0.05 | 0.1 | 0.057 | 0.05 | 0 | 0.02 | 0.052 | 0.044 | 0.075 | 0.127 |
| 56 | 0.081 | 0.03 | 0.05 | 0.131 | 0.07 | 0.05 | 0.091 | 0.01 | 0.07 | 0.057 | 0.081 | 0.121 |
| 57 | 0.07 | 0.06 | 0.05 | 0.141 | 0.05 | 0.04 | 0.033 | 0.03 | 0.052 | 0.085 | 0.098 | 0.049 |
| 58 | 0.042 | 0.01 | 0.052 | 0.09 | 0.07 | 0.1 | 0.024 | 0.052 | 0.03 | 0.073 | 0.101 | 0.112 |
| 59 | 0.042 | 0.024 | 0.05 | 0.141 | 0.06 | 0.06 | 0.03 | 0.02 | 0.04 | 0.027 | 0.151 | 0.08 |
| 60 | 0.061 | 0.084 | 0.03 | 0.07 | 0.071 | 0.08 | 0.05 | 0.03 | 0.042 | 0.072 | 0.061 | 0.066 |
| 61 | 0.048 | 0.066 | 0.02 | 0.084 | 0.05 | 0.05 | 0.04 | 0.02 | 0.04 | 0.027 | 0.08 | 0.077 |
| 62 | 0.052 | 0.04 | 0.07 | 0.071 | 0.121 | 0.08 | 0.075 | 0.04 | 0.05 | 0.055 | 0.075 | 0.073 |
| 63 | 0.03 | 0.05 | 0.04 | 0.06 | 0.08 | 0.06 | 0.042 | 0.033 | 0.05 | 0.062 | 0.101 | 0.05 |
| 64 | 0.03 | 0.06 | 0.091 | 0.01 | 0.05 | 0.02 | 0.03 | 0.03 | 0.05 | 0.095 | 0.052 | 0.044 |
| 65 | 0.04 | 0.057 | 0.03 | 0.061 | 0.05 | 0.04 | 0.03 | 0.02 | 0.07 | 0.038 | 0.081 | 0.068 |
| 66 | 0.02 | 0.033 | 0.04 | 0.08 | 0.052 | 0.11 | 0.052 | 0.033 | 0.07 | 0.077 | 0.042 | 0.066 |
| 67 | 0.07 | 0.04 | 0.075 | 0.084 | 0.05 | 0.101 | 0.02 | 0.042 | 0.08 | 0.049 | 0.07 | 0.075 |
| 68 | 0.061 | 0.033 | 0.052 | 0.07 | 0.07 | 0.08 | 0.01 | 0.02 | 0.03 | 0.088 | 0.061 | 0.055 |
| 69 | 0.061 | 0.042 | 0.042 | 0.094 | 0.02 | 0.01 | 0.042 | 0.052 | 0.07 | 0.078 | 0.08 | 0.049 |
| 70 | 0.03 | 0.024 | 0.057 | 0.097 | 0.071 | 0.033 | 0.04 | 0.066 | 0.071 | 0.038 | 0.131 | 0.038 |
| 71 | 0.05 | 0.03 | 0.06 | 0.122 | 0.06 | 0.02 | 0.04 | 0.05 | 0.03 | 0.068 | 0.071 | 0.06 |
| 72 | 0.06 | 0.04 | 0.02 | 0.101 | 0.094 | 0.033 | 0.033 | 0.042 | 0.066 | 0.078 | 0.084 | 0.084 |
| 73 | 0.05 | 0.01 | 0.061 | 0.091 | 0.1 | 0.048 | 0.02 | 0.033 | 0.07 | 0.05 | 0.06 | 0.1 |
| 74 | 0.052 | 0.06 | 0.048 | 0.084 | 0.061 | 0.052 | 0.01 | 0.071 | 0.03 | 0.055 | 0.111 | 0.049 |
| 75 | 0.04 | 0.03 | 0.05 | 0.131 | 0.091 | 0.103 | 0.066 | 0.04 | 0.057 | 0.044 | 0.113 | 0.071 |
| 76 | 0.05 | 0.033 | 0.02 | 0.075 | 0.09 | 0.07 | 0.096 | 0.01 | 0.06 | 0.111 | 0.05 | 0.1 |
| 77 | 0.03 | 0.05 | 0.024 | 0.08 | 0.06 | 0.05 | 0.02 | 0.01 | 0.04 | 0.099 | 0.048 | 0.055 |
| 78 | 0.06 | 0.061 | 0.05 | 0.094 | 0.06 | 0.042 | 0.06 | 0.033 | 0.113 | 0.06 | 0.101 | 0.077 |
| 79 | 0.06 | 0.04 | 0.05 | 0.1 | 0.081 | 0.101 | 0.05 | 0.052 | 0.09 | 0.095 | 0.11 | 0.095 |
| 80 | 0.06 | 0.03 | 0.01 | 0.091 | 0.057 | 0.05 | 0.04 | 0.07 | 0.066 | 0.082 | 0.117 | 0.075 |
| 81 | 0.05 | 0.04 | 0.042 | 0.04 | 0.05 | 0.07 | 0.042 | 0.08 | 0.05 | 0.089 | 0.084 | 0.064 |
| 82 | 0.05 | 0.024 | 0.04 | 0.13 | 0.091 | 0.03 | 0.02 | 0.05 | 0.02 | 0.077 | 0.123 | 0.073 |
| 83 | 0.04 | 0.04 | 0.03 | 0.09 | 0.098 | 0.05 | 0.05 | 0.052 | 0.07 | 0.028 | 0.091 | 0.079 |
| 84 | 0.04 | 0.04 | 0.05 | 0.18 | 0.03 | 0.052 | 0.042 | 0.04 | 0.042 | 0.055 | 0.08 | 0.064 |
| 85 | 0.06 | 0.04 | 0.048 | 0.05 | 0.05 | 0.03 | 0.024 | 0.071 | 0.09 | 0.116 | 0.03 | 0.075 |
| 86 | 0.064 | 0.05 | 0 | 0.084 | 0.07 | 0.08 | 0.03 | 0.071 | 0.084 | 0.068 | 0.057 | 0.044 |
| 87 | 0.042 | 0.033 | 0.03 | 0.05 | 0.04 | 0.081 | 0.033 | 0.091 | 0.052 | 0.07 | 0.071 | 0.052 |
| 88 | 0.03 | 0.02 | 0.05 | 0.101 | 0.06 | 0.03 | 0.04 | 0.05 | 0.05 | 0.064 | 0.06 | 0.055 |
| 89 | 0.042 | 0.061 | 0.01 | 0.04 | 0.061 | 0.094 | 0.04 | 0.03 | 0.07 | 0.055 | 0.061 | 0.071 |
| 90 | 0.04 | 0.024 | 0.061 | 0.081 | 0.08 | 0.042 | 0.071 | 0.07 | 0.084 | 0.068 | 0.084 | 0.071 |
| 91 | 0.03 | 0.04 | 0.05 | 0.07 | 0.066 | 0.05 | 0.07 | 0.03 | 0.05 | 0.077 | 0.081 | 0.077 |
| 92 | 0.048 | 0.042 | 0.04 | 0.091 | 0.075 | 0.06 | 0.057 | 0.04 | 0.024 | 0.016 | 0.094 | 0.093 |
| 93 | 0.061 | 0.02 | 0.048 | 0.1 | 0.08 | 0.071 | 0.04 | 0.03 | 0.05 | 0.055 | 0.073 | 0.034 |
| 94 | 0.033 | 0.075 | 0.04 | 0.091 | 0.042 | 0.081 | 0.042 | 0.04 | 0.07 | 0.06 | 0.081 | 0.099 |
| 95 | 0.01 | 0.03 | 0.04 | 0.103 | 0.06 | 0.064 | 0.03 | 0.042 | 0.03 | 0.071 | 0.081 | 0.093 |
| 96 | 0.057 | 0.03 | 0.052 | 0.07 | 0.042 | 0.091 | 0.02 | 0.03 | 0.02 | 0.073 | 0.081 | 0.103 |
| 97 | 0.05 | 0.05 | 0.04 | 0.11 | 0.06 | 0.05 | 0.04 | 0.05 | 0.033 | 0.073 | 0.088 | 0.077 |
| 98 | 0.024 | 0.02 | 0.06 | 0.024 | 0.081 | 0.081 | 0.06 | 0.05 | 0.03 | 0.1 | 0.075 | 0.066 |
| 99 | 0.08 | 0.03 | 0.04 | 0.01 | 0.07 | 0.071 | 0.066 | 0.05 | 0.06 | 0.119 | 0.03 | 0.066 |
| 100 | 0.04 | 0.03 | 0.04 | 0.05 | 0.06 | 0.075 | 0.04 | 0.07 | 0.05 | 0.093 | 0.08 | 0.071 |
| 101 | 0.04 | 0.02 | 0.048 | 0.07 | 0.033 | 0.066 | 0.066 | 0.06 | 0.02 | 0.11 | 0.081 | 0.038 |
| 102 | 0.033 | 0.05 | 0.04 | 0.08 | 0.05 | 0.057 | 0.01 | 0.05 | 0.05 | 0.089 | 0.14 | 0.055 |
| 103 | 0.02 | 0.042 | 0.052 | 0.04 | 0.03 | 0.064 | 0.03 | 0.033 | 0.02 | 0.116 | 0.057 | 0.06 |
| 104 | 0.045 | 0.042 | 0.05 | 0.06 | 0.024 | 0.091 | 0.052 | 0.052 | 0.04 | 0.055 | 0.07 | 0.049 |
| 105 | 0.05 | 0.03 | 0.071 | 0.101 | 0.042 | 0.052 | 0.05 | 0.05 | 0.04 | 0.104 | 0.146 | 0.044 |
| 106 | 0.06 | 0.066 | 0.03 | 0.072 | 0.05 | 0.1 | 0.06 | 0.02 | 0.052 | 0.045 | 0.113 | 0.045 |
| 107 | 0.05 | 0.052 | 0.07 | 0.057 | 0.06 | 0.1 | 0.061 | 0.052 | 0.071 | 0.064 | 0.08 | 0.083 |
| 108 | 0.084 | 0.06 | 0.094 | 0.071 | 0.06 | 0.057 | 0.07 | 0.041 | 0.02 | 0.13 | 0.105 | 0.049 |
| 109 | 0.057 | 0.052 | 0.048 | 0.12 | 0.042 | 0.061 | 0.05 | 0.024 | 0.05 | 0.064 | 0.15 | 0.077 |
| 110 | 0.024 | 0.06 | 0.061 | 0.091 | 0.066 | 0.06 | 0.061 | 0.04 | 0.057 | 0.071 | 0.071 | 0.057 |
| 111 | 0.04 | 0.05 | 0.1 | 0.052 | 0.09 | 0.075 | 0.052 | 0.048 | 0.042 | 0.052 | 0.081 | 0.077 |
| 112 | 0.01 | 0.05 | 0.061 | 0.04 | 0.05 | 0.042 | 0.071 | 0.042 | 0.033 | 0.077 | 0.04 | 0.06 |
| 113 | 0.057 | 0.04 | 0.042 | 0.08 | 0.075 | 0.066 | 0.05 | 0.071 | 0.033 | 0.072 | 0.103 | 0.064 |
| 114 | 0.042 | 0.057 | 0.07 | 0.066 | 0.052 | 0.04 | 0.052 | 0.042 | 0.033 | 0.044 | 0.052 | 0.077 |
| 115 | 0.03 | 0.02 | 0.04 | 0.11 | 0.05 | 0.06 | 0.05 | 0.05 | 0.05 | 0.062 | 0.071 | 0.033 |
| 116 | 0.045 | 0.061 | 0.07 | 0.11 | 0.06 | 0.066 | 0.05 | 0.03 | 0.03 | 0.001 | 0.04 | 0.071 |
| 117 | 0.04 | 0.048 | 0.08 | 0.091 | 0.101 | 0.084 | 0.02 | 0.02 | 0.03 | 0.055 | 0.103 | 0.071 |
| 118 | 0.03 | 0.071 | 0.042 | 0.071 | 0.1 | 0.07 | 0.052 | 0.06 | 0.02 | 0.05 | 0.06 | 0.088 |
| 119 | 0.052 | 0.05 | 0.03 | 0.061 | 0.084 | 0.09 | 0.04 | 0.03 | 0.06 | 0.084 | 0.05 | 0.082 |
| 120 | 0.061 | 0.05 | 0.02 | 0.052 | 0.121 | 0.101 | 0.089 | 0.02 | 0.05 | 0.106 | 0.052 | 0.061 |
| 121 | 0.03 | 0.061 | 0.1 | 0.05 | 0.042 | 0.04 | 0.052 | 0.04 | 0.07 | 0.055 | 0.075 | 0.06 |
| 122 | 0.02 | 0.081 | 0.03 | 0.057 | 0.042 | 0.081 | 0.033 | 0.03 | 0.061 | 0.049 | 0.081 | 0.05 |
| 123 | 0.05 | 0.04 | 0.05 | 0.064 | 0.024 | 0.08 | 0.081 | 0.04 | 0.052 | 0.033 | 0.04 | 0.039 |
| 124 | 0.06 | 0.04 | 0.07 | 0.05 | 0.042 | 0.061 | 0.04 | 0.02 | 0.04 | 0.05 | 0.08 | 0.066 |
| 125 | 0.033 | 0.04 | 0.042 | 0.1 | 0.052 | 0.08 | 0.06 | 0.052 | 0.045 | 0.094 | 0.071 | 0.095 |
| 126 | 0.04 | 0.042 | 0.04 | 0.03 | 0.048 | 0.06 | 0.05 | 0.033 | 0.057 | 0.075 | 0.07 | 0.156 |
| 127 | 0.06 | 0.061 | 0.06 | 0.052 | 0.02 | 0.061 | 0.052 | 0.08 | 0.057 | 0.102 | 0.1 | 0.147 |
| 128 | 0.052 | 0.04 | 0.04 | 0.071 | 0.101 | 0.052 | 0.05 | 0.033 | 0.05 | 0.111 | 0.052 | 0.103 |
| 129 | 0.06 | 0.05 | 0.024 | 0.06 | 0.061 | 0.1 | 0.06 | 0.06 | 0.064 | 0.05 | 0.08 | 0.084 |
| 130 | 0.042 | 0.033 | 0.066 | 0.143 | 0.05 | 0.033 | 0.052 | 0.04 | 0.048 | 0.088 | 0.08 | 0.06 |
| 131 | 0.05 | 0.01 | 0.08 | 0.111 | 0.06 | 0.06 | 0.04 | 0.03 | 0.08 | 0.072 | 0.081 | 0.121 |
| 132 | 0.061 | 0.071 | 0.06 | 0.05 | 0.09 | 0.06 | 0.04 | 0.024 | 0.057 | 0.088 | 0.08 | 0.088 |
| 133 | 0.03 | 0.042 | 0.06 | 0.07 | 0.121 | 0.06 | 0.04 | 0.04 | 0.064 | 0.093 | 0.07 | 0.06 |
| 134 | 0.07 | 0.111 | 0.07 | 0.02 | 0.061 | 0.07 | 0.06 | 0.03 | 0.064 | 0.066 | 0.091 | 0.077 |
| 135 | 0.066 | 0.07 | 0.081 | 0.03 | 0.03 | 0.061 | 0.04 | 0.02 | 0.052 | 0.093 | 0.1 | 0.099 |
| 136 | 0.07 | 0.052 | 0.101 | 0.123 | 0.05 | 0.05 | 0.072 | 0 | 0.033 | 0.093 | 0.05 | 0.094 |
| 137 | 0.05 | 0.08 | 0.05 | 0.066 | 0.048 | 0.042 | 0.05 | 0.02 | 0.094 | 0.088 | 0.06 | 0.094 |
| 138 | 0.01 | 0.09 | 0.04 | 0.08 | 0.03 | 0.05 | 0.06 | 0.04 | 0.013 | 0.104 | 0.091 | 0.088 |
| 139 | 0.06 | 0.071 | 0.071 | 0.07 | 0.024 | 0.052 | 0.03 | 0.03 | 0.057 | 0.093 | 0.042 | 0.049 |
| 140 | 0.02 | 0.04 | 0.042 | 0.123 | 0.042 | 0.042 | 0.01 | 0.017 | 0.042 | 0.094 | 0.06 | 0.083 |
| 141 | 0.061 | 0.08 | 0.07 | 0.04 | 0.07 | 0.042 | 0.05 | 0.02 | 0.071 | 0.059 | 0.061 | 0.077 |
| 142 | 0.05 | 0.04 | 0.052 | 0.05 | 0.04 | 0.06 | 0.03 | 0.02 | 0.04 | 0.099 | 0.06 | 0.064 |
| 143 | 0.05 | 0.024 | 0.061 | 0.081 | 0.075 | 0.07 | 0.05 | 0.05 | 0.06 | 0.099 | 0.061 | 0.072 |
| 144 | 0.04 | 0.04 | 0.042 | 0.121 | 0.066 | 0.06 | 0.05 | 0.03 | 0.04 | 0.066 | 0.103 | 0.08 |
| 145 | 0.04 | 0.075 | 0.061 | 0.03 | 0.042 | 0.05 | 0.061 | 0.071 | 0.03 | 0.06 | 0.101 | 0.077 |
| 146 | 0.048 | 0.091 | 0.07 | 0.06 | 0.04 | 0.071 | 0.02 | 0.04 | 0.04 | 0.121 | 0.066 | 0.111 |
| 147 | 0.066 | 0.075 | 0.042 | 0.042 | 0.05 | 0.06 | 0.03 | 0.04 | 0.03 | 0.072 | 0.111 | 0.082 |
| 148 | 0.04 | 0.071 | 0.033 | 0.084 | 0.08 | 0.052 | 0.052 | 0.02 | 0.02 | 0.077 | 0.113 | 0.066 |
| 149 | 0.05 | 0.033 | 0.03 | 0.136 | 0.12 | 0.09 | 0.06 | 0.024 | 0.02 | 0.122 | 0.06 | 0.072 |
| 150 | 0.033 | 0.04 | 0.071 | 0.08 | 0.121 | 0.08 | 0.04 | 0.02 | 0.033 | 0.073 | 0.05 | 0.066 |
| 151 | 0.05 | 0.03 | 0.04 | 0.084 | 0.06 | 0.09 | 0.052 | 0.03 | 0.06 | 0.044 | 0.07 | 0.131 |
| 152 | 0.042 | 0.04 | 0.09 | 0.113 | 0.061 | 0.07 | 0.084 | 0.04 | 0.02 | 0.116 | 0.066 | 0.052 |
| 153 | 0.061 | 0.03 | 0.04 | 0.07 | 0.05 | 0.06 | 0.07 | 0.033 | 0.052 | 0.148 | 0.075 | 0.082 |
| 154 | 0.07 | 0.103 | 0.081 | 0.081 | 0.06 | 0.05 | 0.094 | 0.017 | 0.071 | 0.088 | 0.081 | 0.077 |
| 155 | 0.052 | 0.03 | 0.07 | 0.103 | 0.06 | 0.05 | 0.04 | 0.057 | 0.033 | 0.057 | 0.066 | 0.08 |
| 156 | 0.04 | 0.01 | 0.061 | 0.061 | 0.052 | 0.03 | 0.061 | 0.033 | 0.05 | 0.022 | 0.073 | 0.09 |
| 157 | 0.04 | 0.04 | 0.06 | 0.09 | 0.07 | 0.071 | 0.061 | 0.048 | 0.057 | 0.11 | 0.088 | 0.109 |
| 158 | 0.02 | 0.04 | 0.052 | 0.057 | 0.09 | 0.05 | 0.048 | 0.05 | 0.064 | 0.072 | 0.105 | 0.111 |
| 159 | 0.06 | 0.01 | 0.02 | 0.179 | 0.07 | 0.061 | 0.04 | 0.02 | 0.03 | 0.077 | 0.071 | 0.055 |
| 160 | 0.05 | 0.091 | 0.04 | 0.121 | 0.08 | 0.08 | 0.066 | 0.05 | 0.05 | 0.044 | 0.05 | 0.038 |
| 161 | 0.033 | 0.071 | 0.061 | 0.1 | 0.052 | 0.111 | 0.04 | 0.03 | 0 | 0.077 | 0.05 | 0.101 |
| 162 | 0.042 | 0.03 | 0.048 | 0.09 | 0.06 | 0.14 | 0.05 | 0.06 | 0.04 | 0.071 | 0.033 | 0.064 |
| 163 | 0.07 | 0.05 | 0.066 | 0.113 | 0.05 | 0.057 | 0.02 | 0.052 | 0.024 | 0.077 | 0.04 | 0.077 |
| 164 | 0.071 | 0.042 | 0.08 | 0.113 | 0.101 | 0.04 | 0.024 | 0.04 | 0.057 | 0.066 | 0.066 | 0.055 |
| 165 | 0.042 | 0.071 | 0.104 | 0.071 | 0.066 | 0.075 | 0.06 | 0.03 | 0.052 | 0.066 | 0.126 | 0.077 |
| 166 | 0.05 | 0.033 | 0.072 | 0.071 | 0.141 | 0.052 | 0.04 | 0.04 | 0.061 | 0.094 | 0.165 | 0.071 |
| 167 | 0.033 | 0.042 | 0.064 | 0.05 | 0.03 | 0.042 | 0.07 | 0.052 | 0.03 | 0.066 | 0.066 | 0.08 |
| 168 | 0.05 | 0.052 | 0.064 | 0.061 | 0.06 | 0.03 | 0.05 | 0.01 | 0.042 | 0.121 | 0.089 | 0.115 |
| 169 | 0.042 | 0.04 | 0.096 | 0.1 | 0.05 | 0.05 | 0.05 | 0.03 | 0.04 | 0.049 | 0.113 | 0.077 |
| 170 | 0.03 | 0.042 | 0.08 | 0.03 | 0.081 | 0.07 | 0.042 | 0.04 | 0.042 | 0.088 | 0.108 | 0.055 |
| 171 | 0.066 | 0.02 | 0.072 | 0.08 | 0.061 | 0.1 | 0.04 | 0.03 | 0.01 | 0.055 | 0.098 | 0.114 |
| 172 | 0.06 | 0.04 | 0.052 | 0.07 | 0.075 | 0.098 | 0.07 | 0.02 | 0.03 | 0.121 | 0.084 | 0.044 |
| 173 | 0.06 | 0.042 | 0.04 | 0.141 | 0.066 | 0.08 | 0.03 | 0.03 | 0.03 | 0.055 | 0.075 | 0.049 |
| 174 | 0.07 | 0.033 | 0.08 | 0.141 | 0.091 | 0.08 | 0.04 | 0.024 | 0.04 | 0.083 | 0.066 | 0.038 |
| 175 | 0.05 | 0.05 | 0.048 | 0.13 | 0.1 | 0.061 | 0.061 | 0.04 | 0.024 | 0.143 | 0.07 | 0.116 |
| 176 | 0.05 | 0.033 | 0.094 | 0.131 | 0.07 | 0.03 | 0.04 | 0.024 | 0.01 | 0.082 | 0.094 | 0.055 |
| 177 | 0.08 | 0.08 | 0.08 | 0.143 | 0.07 | 0.081 | 0.04 | 0.042 | 0.05 | 0.045 | 0.081 | 0.055 |
| 178 | 0.071 | 0.04 | 0.06 | 0.141 | 0.02 | 0.052 | 0.052 | 0.02 | 0.05 | 0.077 | 0.091 | 0.066 |
| 179 | 0.042 | 0.05 | 0.061 | 0.11 | 0.06 | 0.06 | 0.03 | 0.04 | 0.042 | 0.045 | 0.081 | 0.096 |
| 180 | 0.04 | 0.02 | 0.04 | 0.094 | 0.061 | 0.05 | 0.03 | 0.04 | 0.04 | 0.061 | 0.05 | 0.094 |
| 181 | 0.06 | 0.061 | 0.03 | 0.081 | 0.061 | 0.11 | 0.03 | 0.04 | 0.033 | 0.06 | 0.094 | 0.071 |
| 182 | 0.06 | 0.05 | 0.033 | 0.057 | 0.06 | 0.071 | 0.03 | 0.071 | 0.057 | 0.05 | 0.084 | 0.066 |
| 183 | 0.06 | 0.03 | 0.088 | 0.052 | 0.03 | 0.103 | 0.08 | 0.052 | 0.033 | 0.071 | 0.097 | 0.088 |
| 184 | 0.05 | 0.05 | 0.06 | 0.05 | 0.071 | 0.09 | 0.04 | 0.061 | 0.048 | 0.049 | 0.071 | 0.068 |
| 185 | 0.06 | 0.042 | 0.04 | 0.06 | 0.061 | 0.06 | 0.05 | 0.05 | 0.052 | 0.09 | 0.133 | 0.044 |
| 186 | 0.042 | 0.057 | 0.03 | 0.101 | 0.05 | 0.091 | 0.042 | 0.08 | 0.03 | 0.107 | 0.1 | 0.066 |
| 187 | 0.05 | 0.04 | 0.05 | 0.1 | 0.08 | 0.081 | 0.02 | 0.05 | 0.04 | 0.066 | 0.091 | 0.077 |
| 188 | 0.075 | 0.042 | 0.052 | 0.2 | 0.091 | 0.071 | 0.033 | 0.08 | 0.017 | 0.082 | 0.081 | 0.049 |
| 189 | 0.081 | 0.061 | 0.04 | 0.04 | 0.133 | 0.081 | 0.033 | 0.02 | 0.06 | 0.072 | 0.071 | 0.088 |
| 190 | 0.075 | 0.052 | 0.091 | 0.103 | 0.048 | 0.06 | 0.061 | 0.01 | 0.052 | 0.084 | 0.1 | 0.094 |
| 191 | 0.042 | 0.06 | 0.09 | 0.091 | 0.111 | 0.042 | 0.05 | 0.052 | 0.06 | 0.104 | 0.081 | 0.066 |
| 192 | 0.03 | 0.024 | 0.033 | 0.121 | 0.061 | 0.081 | 0.06 | 0.04 | 0.1 | 0.044 | 0.07 | 0.049 |
| 193 | 0.08 | 0.03 | 0.04 | 0.09 | 0.07 | 0.05 | 0.061 | 0.052 | 0.05 | 0.055 | 0.07 | 0.062 |
| 194 | 0.04 | 0.042 | 0.06 | 0.09 | 0.1 | 0.04 | 0.042 | 0.03 | 0.06 | 0.055 | 0.084 | 0.061 |
| 195 | 0.033 | 0.017 | 0.081 | 0.113 | 0.06 | 0.07 | 0.03 | 0.02 | 0.071 | 0.049 | 0.081 | 0.066 |
| 196 | 0.042 | 0.05 | 0.071 | 0.08 | 0.091 | 0.06 | 0.02 | 0.04 | 0.04 | 0.027 | 0.07 | 0.071 |
| 197 | 0.05 | 0.075 | 0.06 | 0.08 | 0.04 | 0.071 | 0.05 | 0.042 | 0.05 | 0.045 | 0.072 | 0.082 |
| 198 | 0.04 | 0.057 | 0.071 | 0.081 | 0.042 | 0.098 | 0.06 | 0.04 | 0.03 | 0.033 | 0.07 | 0.033 |
| 199 | 0.04 | 0.071 | 0.052 | 0.06 | 0.081 | 0.111 | 0.02 | 0.061 | 0.061 | 0.055 | 0.05 | 0.088 |
| 200 | 0.05 | 0.02 | 0.111 | 0.04 | 0.042 | 0.123 | 0.03 | 0.061 | 0.05 | 0.055 | 0.133 | 0.044 |
| 201 | 0.07 | 0.042 | 0.06 | 0.07 | 0.05 | 0.103 | 0.02 | 0.06 | 0.04 | 0.038 | 0.121 | 0.061 |
| 202 | 0.05 | 0.042 | 0.05 | 0.081 | 0.061 | 0.06 | 0.02 | 0.05 | 0.05 | 0.072 | 0.075 | 0.077 |
| 203 | 0.042 | 0.03 | 0.05 | 0.17 | 0.08 | 0.071 | 0.03 | 0.05 | 0.03 | 0.083 | 0.094 | 0.066 |
| 204 | 0.033 | 0.042 | 0.042 | 0.111 | 0.042 | 0.075 | 0.02 | 0.02 | 0.024 | 0.066 | 0.126 | 0.055 |
| 205 | 0.07 | 0.05 | 0.04 | 0.057 | 0.04 | 0.048 | 0.06 | 0.042 | 0.05 | 0.082 | 0.09 | 0.082 |
| 206 | 0.02 | 0.052 | 0.052 | 0.061 | 0.081 | 0.052 | 0.042 | 0.066 | 0.01 | 0.104 | 0.08 | 0.049 |
| 207 | 0.04 | 0.08 | 0.05 | 0.071 | 0.057 | 0.081 | 0.06 | 0.024 | 0.052 | 0.088 | 0.094 | 0.055 |
| 208 | 0.03 | 0.06 | 0.061 | 0.042 | 0.057 | 0.05 | 0.05 | 0.05 | 0.042 | 0.066 | 0.066 | 0.082 |
| 209 | 0.07 | 0.06 | 0.08 | 0.09 | 0.075 | 0.071 | 0.061 | 0.042 | 0.071 | 0.107 | 0.117 | 0.071 |
| 210 | 0.066 | 0.06 | 0.094 | 0.121 | 0.075 | 0.06 | 0.04 | 0.071 | 0.04 | 0.088 | 0.088 | 0.073 |
| 211 | 0.07 | 0.05 | 0.05 | 0.101 | 0.045 | 0.042 | 0.03 | 0.024 | 0.02 | 0.055 | 0.081 | 0.077 |
| 212 | 0.05 | 0.02 | 0.09 | 0.101 | 0.057 | 0.052 | 0.061 | 0.04 | 0.02 | 0.094 | 0.09 | 0.039 |
| 213 | 0.04 | 0.01 | 0.091 | 0.207 | 0.057 | 0.094 | 0.024 | 0.052 | 0.024 | 0.099 | 0.111 | 0.093 |
| 214 | 0.057 | 0.03 | 0.08 | 0.131 | 0.052 | 0.094 | 0.033 | 0.033 | 0.02 | 0.08 | 0.12 | 0.11 |
| 215 | 0.071 | 0.03 | 0.101 | 0.161 | 0.048 | 0.061 | 0.07 | 0.06 | 0.05 | 0.071 | 0.111 | 0.071 |
| 216 | 0.061 | 0.013 | 0.07 | 0.081 | 0.033 | 0.071 | 0.066 | 0.042 | 0.03 | 0.049 | 0.11 | 0.084 |
| 217 | 0.052 | 0.075 | 0.04 | 0.06 | 0.08 | 0.108 | 0.04 | 0.04 | 0.052 | 0.055 | 0.06 | 0.044 |
| 218 | 0.057 | 0.048 | 0.03 | 0.07 | 0.064 | 0.08 | 0.03 | 0.02 | 0.03 | 0.06 | 0.101 | 0.062 |
| 219 | 0.04 | 0.042 | 0.05 | 0.16 | 0.052 | 0.071 | 0.02 | 0.04 | 0.042 | 0.088 | 0.141 | 0.079 |
| 220 | 0.03 | 0.02 | 0.03 | 0.18 | 0.104 | 0.103 | 0.04 | 0.07 | 0.05 | 0.046 | 0.103 | 0.061 |
| 221 | 0.02 | 0.052 | 0.05 | 0.091 | 0.103 | 0.08 | 0.017 | 0.05 | 0.02 | 0.062 | 0.09 | 0.05 |
| 222 | 0.033 | 0.033 | 0.07 | 0.05 | 0.072 | 0.113 | 0.061 | 0.03 | 0.03 | 0.06 | 0.052 | 0.1 |
| 223 | 0.071 | 0.042 | 0.042 | 0.061 | 0.066 | 0.075 | 0.04 | 0.061 | 0.06 | 0.028 | 0.06 | 0.084 |
| 224 | 0.081 | 0.042 | 0.052 | 0.06 | 0.1 | 0.088 | 0.04 | 0.03 | 0.03 | 0.071 | 0.061 | 0.101 |
| 225 | 0.07 | 0.042 | 0.03 | 0.1 | 0.048 | 0.081 | 0.04 | 0.061 | 0.052 | 0.1 | 0.09 | 0.033 |
| 226 | 0.061 | 0.06 | 0.06 | 0.071 | 0.048 | 0.075 | 0.05 | 0.04 | 0.033 | 0.044 | 0.113 | 0.068 |
| 227 | 0.075 | 0.066 | 0.071 | 0.192 | 0.07 | 0.12 | 0.06 | 0.02 | 0.02 | 0.049 | 0.141 | 0.044 |
| 228 | 0.081 | 0.03 | 0.02 | 0.192 | 0.08 | 0.042 | 0.02 | 0.052 | 0.042 | 0.077 | 0.136 | 0.128 |
| 229 | 0.097 | 0.03 | 0.09 | 0.1 | 0.071 | 0.052 | 0.061 | 0.06 | 0.04 | 0.073 | 0.133 | 0.027 |
| 230 | 0.061 | 0.04 | 0.071 | 0.14 | 0.03 | 0.07 | 0.04 | 0.04 | 0.017 | 0.049 | 0.09 | 0.046 |
| 231 | 0.05 | 0.052 | 0.04 | 0.131 | 0.075 | 0.111 | 0.05 | 0.05 | 0.04 | 0.078 | 0.103 | 0.064 |
| 232 | 0.04 | 0.033 | 0.03 | 0.131 | 0.162 | 0.052 | 0.06 | 0.07 | 0.06 | 0.088 | 0.09 | 0.055 |
| 233 | 0.052 | 0.024 | 0.061 | 0.1 | 0.02 | 0.042 | 0.03 | 0.081 | 0.03 | 0.055 | 0.143 | 0.083 |
| 234 | 0.061 | 0.052 | 0.1 | 0.1 | 0.05 | 0.11 | 0.03 | 0.052 | 0.071 | 0.066 | 0.12 | 0.116 |
| 235 | 0.04 | 0.07 | 0.06 | 0.091 | 0.052 | 0.042 | 0.033 | 0.06 | 0.04 | 0.05 | 0.061 | 0.066 |
| 236 | 0.091 | 0.05 | 0.052 | 0.03 | 0.081 | 0.07 | 0.02 | 0.052 | 0.03 | 0.066 | 0.09 | 0.127 |
| 237 | 0.042 | 0.04 | 0.04 | 0.04 | 0.05 | 0.04 | 0.06 | 0.01 | 0.05 | 0.072 | 0.094 | 0.104 |
| 238 | 0.066 | 0.05 | 0.042 | 0.06 | 0.03 | 0.071 | 0.04 | 0.052 | 0.05 | 0.058 | 0.104 | 0.077 |
| 239 | 0.084 | 0.04 | 0.05 | 0.06 | 0 | 0.042 | 0.04 | 0.042 | 0.04 | 0.072 | 0.07 | 0.044 |
| 240 | 0.05 | 0.071 | 0.06 | 0.081 | 0.03 | 0.04 | 0.02 | 0.061 | 0.07 | 0.071 | 0.101 | 0.062 |
| 241 | 0.033 | 0.05 | 0.04 | 0.094 | 0.03 | 0.048 | 0.033 | 0.05 | 0.03 | 0.077 | 0.07 | 0.073 |
| 242 | 0.052 | 0.052 | 0.075 | 0.05 | 0.03 | 0.033 | 0.05 | 0.05 | 0.052 | 0.093 | 0.07 | 0.107 |
| 243 | 0.083 | 0.057 | 0.05 | 0.033 | 0.033 | 0.02 | 0.05 | 0.02 | 0.033 | 0.11 | 0.081 | 0.093 |
| 244 | 0.04 | 0.06 | 0.075 | 0.094 | 0.042 | 0.042 | 0.033 | 0.04 | 0.052 | 0.099 | 0.11 | 0.098 |
| 245 | 0.06 | 0.04 | 0.061 | 0.057 | 0.06 | 0.024 | 0.04 | 0.057 | 0.033 | 0.039 | 0.061 | 0.09 |
| 246 | 0.04 | 0.052 | 0.033 | 0.061 | 0.06 | 0.05 | 0.042 | 0.03 | 0.03 | 0.088 | 0.101 | 0.06 |
| 247 | 0.08 | 0.071 | 0.02 | 0.07 | 0.07 | 0.091 | 0.02 | 0.033 | 0.03 | 0.062 | 0.075 | 0.07 |
| 248 | 0.052 | 0.06 | 0.05 | 0.033 | 0.052 | 0.061 | 0.042 | 0.04 | 0.024 | 0.072 | 0.042 | 0.05 |
| 249 | 0.042 | 0.07 | 0.03 | 0.091 | 0.07 | 0.071 | 0.08 | 0.081 | 0.042 | 0.059 | 0.084 | 0.099 |
| 250 | 0.03 | 0.071 | 0.06 | 0.096 | 0.071 | 0.06 | 0.033 | 0.03 | 0.017 | 0.066 | 0.131 | 0.071 |
| Mean | 0.049896 | 0.046764 | 0.053108 | 0.081824 | 0.062988 | 0.066424 | 0.04442 | 0.041016 | 0.045524 | 0.072556 | 0.0833 | 0.074256 |
| SD | 0.016858 | 0.018157 | 0.020395 | 0.035977 | 0.024304 | 0.023748 | 0.017363 | 0.016998 | 0.018366 | 0.023735 | 0.025888 | 0.024478 |

**2. Automated gap thickness measurements for AxC169 variety**

The following tables show the total number of OCT images used for manual gap thickness measurements in the AxC169 variety. A total of 200 OCT images were analyzed, with segmentation of the first 2–3 cell layers performed using the OCT Analyzer software. For both control and infected leaves, three measurements were taken daily from day 0 (D0) to day 7 (D7). The software provided values for the maximum (Max), minimum (Min), and average gap thickness. The average value being the most relevant for our study.

| Control (mm) Infected (mm)  Day 0 R1 R2 R3 R1 R2 R3  Max Min Average Max Min Average Max Min Average Max Min Average Max Min Average Max Min Average  1 0.04 0 0.02 0.09 0 0.03 0.08 0 0.03 0.07 0 0.03 0.08 0 0.03 0.07 0 0.03  2 0.05 0 0.03 0.06 0 0.04 0.06 0 0.04 0.07 0 0.04 0.1 0 0.04 0.07 0 0.03  3 0.05 0 0.02 0.07 0 0.03 0.08 0 0.04 0.07 0 0.04 0.09 0 0.04 0.09 0 0.04  4 0.05 0 0.01 0.08 0 0.04 0.07 0 0.04 0.07 0 0.04 0.09 0 0.04 0.08 0 0.03  5 0.04 0 0.02 0.08 0 0.04 0.08 0 0.04 0.08 0 0.04 0.08 0 0.04 0.07 0 0.03  6 0.04 0 0.01 0.08 0 0.04 0.07 0 0.04 0.07 0 0.03 0.07 0 0.04 0.08 0 0.03  7 0.02 0 0.01 0.07 0 0.04 0.09 0 0.04 0.08 0 0.04 0.07 0 0.04 0.08 0 0.03  8 0.02 0 0.01 0.07 0 0.04 0.07 0 0.04 0.07 0 0.04 0.08 0 0.04 0.08 0 0.04  9 0.02 0 0.01 0.08 0 0.04 0.08 0 0.04 0.07 0 0.04 0.07 0 0.03 0.08 0 0.03  10 0.04 0 0.02 0.09 0 0.04 0.08 0 0.04 0.08 0 0.04 0.07 0 0.04 0.08 0 0.04  11 0.05 0 0.03 0.08 0 0.04 0.07 0 0.04 0.09 0 0.04 0.08 0 0.04 0.08 0 0.04  12 0.06 0 0.04 0.1 0 0.05 0.08 0 0.04 0.09 0.01 0.04 0.08 0 0.04 0.08 0 0.04  13 0.06 0 0.04 0.09 0 0.04 0.08 0 0.05 0.08 0 0.04 0.08 0 0.04 0.08 0 0.03  14 0.06 0.01 0.04 0.09 0 0.04 0.08 0 0.04 0.07 0 0.04 0.08 0 0.03 0.08 0 0.03  15 0.06 0 0.04 0.1 0 0.04 0.07 0 0.04 0.07 0 0.04 0.08 0 0.04 0.09 0 0.04  16 0.07 0 0.05 0.09 0 0.04 0.08 0 0.04 0.06 0 0.04 0.08 0 0.04 0.09 0 0.04  17 0.06 0.03 0.05 0.08 0 0.04 0.08 0 0.04 0.08 0 0.04 0.08 0 0.04 0.08 0 0.04  18 0.07 0 0.05 0.09 0 0.05 0.07 0 0.04 0.07 0.01 0.04 0.08 0 0.04 0.09 0 0.04  19 0.07 0 0.04 0.09 0.01 0.05 0.08 0 0.05 0.09 0 0.04 0.08 0 0.04 0.08 0 0.04  20 0.07 0 0.05 0.09 0 0.05 0.08 0 0.04 0.09 0 0.04 0.07 0 0.04 0.08 0 0.04  21 0.07 0.03 0.06 0.08 0 0.05 0.1 0 0.05 0.08 0 0.04 0.07 0 0.05 0.09 0 0.03  22 0.08 0 0.05 0.08 0 0.04 0.09 0 0.05 0.09 0 0.04 0.08 0 0.05 0.1 0 0.03  23 0.08 0 0.05 0.08 0.02 0.06 0.08 0 0.05 0.09 0 0.04 0.08 0 0.05 0.08 0 0.03  24 0.08 0.04 0.06 0.07 0 0.06 0.08 0 0.05 0.09 0 0.04 0.09 0 0.04 0.08 0 0.03  25 0.08 0.04 0.06 0.08 0 0.06 0.08 0 0.04 0.07 0 0.04 0.09 0 0.04 0.08 0 0.04  26 0.07 0 0.05 0.08 0 0.04 0.08 0 0.05 0.1 0 0.04 0.09 0 0.04 0.08 0 0.03  27 0.07 0 0.04 0.08 0 0.04 0.09 0 0.05 0.07 0 0.04 0.08 0 0.04 0.08 0 0.04  28 0.06 0 0.03 0.07 0 0.04 0.09 0 0.05 0.08 0 0.05 0.1 0 0.04 0.08 0 0.03  29 0.05 0 0.03 0.07 0 0.04 0.09 0 0.05 0.09 0 0.04 0.09 0 0.03 0.09 0 0.03  30 0.05 0 0.03 0.09 0 0.04 0.11 0 0.06 0.08 0 0.05 0.09 0 0.03 0.08 0 0.03  31 0.05 0 0.03 0.07 0 0.04 0.09 0.03 0.06 0.07 0 0.04 0.08 0 0.03 0.09 0 0.03  32 0.05 0 0.03 0.07 0 0.04 0.08 0 0.04 0.07 0 0.04 0.1 0 0.04 0.09 0 0.03  33 0.04 0 0.03 0.08 0 0.04 0.08 0 0.04 0.07 0 0.04 0.09 0 0.04 0.09 0 0.04  34 0.04 0 0.03 0.08 0 0.04 0.1 0 0.05 0.08 0 0.04 0.1 0 0.05 0.08 0 0.04  35 0.05 0 0.03 0.07 0 0.04 0.08 0 0.05 0.06 0 0.04 0.11 0 0.05 0.09 0 0.04  36 0.04 0 0.03 0.1 0 0.04 0.08 0.08 0.06 0.07 0 0.04 0.09 0 0.05 0.08 0 0.03  37 0.04 0 0.03 0.09 0 0.05 0.08 0 0.05 0.07 0 0.04 0.09 0 0.05 0.07 0 0.03  38 0.05 0 0.03 0.08 0 0.04 0.08 0 0.05 0.08 0 0.04 0.08 0 0.03 0.06 0 0.03  39 0.06 0 0.04 0.08 0 0.05 0.09 0 0.05 0.07 0 0.04 0.09 0 0.04 0.08 0 0.03  40 0.06 0 0.04 0.08 0 0.05 0.09 0.03 0.05 0.09 0 0.04 0.09 0 0.03 0.08 0 0.03  41 0.06 0 0.04 0.09 0 0.05 0.08 0 0.05 0.08 0 0.04 0.08 0 0.03 0.08 0 0.03  42 0.07 0 0.04 0.07 0 0.05 0.1 0 0.05 0.07 0 0.04 0.09 0 0.03 0.08 0 0.03  43 0.08 0 0.04 0.08 0 0.05 0.09 0.02 0.05 0.08 0 0.04 0.08 0 0.03 0.08 0 0.03  44 0.07 0 0.04 0.1 0 0.04 0.06 0 0.04 0.07 0 0.04 0.1 0 0.05 0.08 0 0.03  45 0.08 0 0.05 0.09 0 0.05 0.08 0 0.04 0.09 0 0.04 0.08 0 0.06 0.08 0 0.03  46 0.08 0 0.05 0.09 0 0.05 0.12 0 0.04 0.1 0 0.04 0.1 0 0.06 0.1 0 0.03  47 0.06 0 0.04 0.09 0.02 0.05 0.1 0 0.04 0.09 0 0.04 0.09 0 0.06 0.08 0 0.03  48 0.08 0.02 0.05 0.09 0.01 0.05 0.08 0 0.04 0.1 0 0.04 0.09 0 0.06 0.08 0 0.04  49 0.07 0 0.04 0.1 0.03 0.05 0.11 0 0.04 0.08 0 0.04 0.11 0 0.06 0.09 0 0.02  50 0.06 0 0.04 0.1 0 0.05 0.09 0.02 0.05 0.09 0 0.04 0.09 0 0.06 0.09 0 0.03  51 0.06 0 0.04 0.1 0 0.06 0.08 0.02 0.05 0.1 0 0.04 0.09 0 0.06 0.09 0 0.03  52 0.07 0 0.04 0.1 0.03 0.06 0.08 0 0.04 0.07 0 0.04 0.09 0 0.06 0.07 0 0.03  53 0.05 0 0.03 0.08 0 0.06 0.1 0.03 0.05 0.1 0 0.04 0.11 0 0.06 0.08 0 0.03  54 0.04 0 0.03 0.1 0 0.06 0.11 0 0.06 0.1 0 0.04 0.11 0 0.06 0.08 0 0.03  55 0.04 0 0.03 0.09 0.02 0.06 0.13 0.03 0.06 0.09 0 0.05 0.09 0 0.06 0.09 0 0.03  56 0.05 0 0.03 0.07 0.02 0.06 0.09 0 0.05 0.08 0 0.04 0.1 0 0.06 0.08 0 0.03  57 0.06 0 0.04 0.08 0.01 0.06 0.09 0 0.04 0.09 0 0.05 0.09 0 0.06 0.08 0 0.03  58 0.06 0 0.04 0.07 0.01 0.06 0.09 0.01 0.05 0.09 0 0.06 0.1 0 0.07 0.09 0 0.03  59 0.06 0 0.04 0.1 0 0.06 0.09 0 0.05 0.08 0 0.06 0.09 0 0.07 0.07 0 0.03  60 0.07 0 0.04 0.11 0.02 0.06 0.09 0 0.05 0.09 0 0.06 0.09 0 0.06 0.09 0 0.03  61 0.08 0 0.04 0.08 0 0.06 0.1 0 0.05 0.09 0 0.06 0.11 0 0.06 0.08 0 0.03  62 0.07 0 0.04 0.09 0 0.06 0.07 0 0.05 0.07 0 0.06 0.1 0 0.06 0.08 0 0.04  63 0.08 0 0.05 0.07 0 0.06 0.07 0 0.05 0.09 0 0.06 0.1 0 0.06 0.08 0 0.03  64 0.08 0 0.05 0.08 0 0.06 0.09 0.01 0.05 0.09 0 0.06 0.1 0 0.06 0.08 0 0.05  65 0.06 0 0.04 0.09 0 0.04 0.1 0 0.05 0.08 0 0.06 0.09 0 0.06 0.08 0 0.05  66 0.08 0.02 0.05 0.1 0 0.05 0.12 0 0.04 0.08 0 0.07 0.08 0 0.06 0.09 0 0.06  67 0.07 0 0.04 0.09 0 0.05 0.12 0 0.05 0.09 0 0.07 0.09 0 0.05 0.08 0 0.06  68 0.06 0 0.04 0.11 0 0.05 0.11 0 0.05 0.08 0 0.07 0.12 0 0.05 0.09 0 0.06  69 0.06 0 0.04 0.11 0 0.05 0.12 0 0.05 0.08 0 0.07 0.11 0 0.05 0.08 0 0.06  70 0.07 0 0.04 0.09 0 0.05 0.11 0 0.04 0.09 0 0.06 0.12 0 0.05 0.1 0 0.06  71 0.08 0 0.04 0.11 0 0.05 0.1 0 0.04 0.11 0 0.06 0.12 0 0.04 0.09 0 0.04  72 0.07 0 0.04 0.11 0 0.06 0.1 0 0.04 0.09 0 0.06 0.11 0 0.04 0.09 0 0.04  73 0.06 0 0.03 0.13 0 0.06 0.1 0 0.05 0.07 0 0.06 0.1 0 0.04 0.08 0 0.03  74 0.06 0 0.03 0.12 0 0.06 0.08 0 0.04 0.09 0 0.06 0.1 0 0.04 0.08 0 0.03  75 0.07 0 0.03 0.12 0.02 0.05 0.09 0 0.04 0.07 0 0.06 0.11 0 0.06 0.09 0 0.03  76 0.09 0 0.03 0.16 0.03 0.05 0.09 0 0.04 0.08 0 0.06 0.09 0 0.07 0.08 0 0.03  77 0.08 0 0.04 0.11 0 0.05 0.1 0 0.05 0.09 0 0.06 0.08 0 0.07 0.09 0 0.03  78 0.08 0.02 0.05 0.11 0 0.05 0.13 0 0.06 0.08 0 0.06 0.09 0 0.08 0.09 0 0.04  79 0.07 0 0.04 0.09 0.03 0.06 0.12 0 0.07 0.07 0 0.07 0.09 0 0.08 0.09 0 0.04  80 0.06 0 0.04 0.09 0 0.05 0.11 0 0.07 0.1 0 0.07 0.09 0 0.08 0.08 0 0.03  81 0.07 0.01 0.05 0.09 0 0.05 0.17 0 0.05 0.09 0 0.06 0.11 0 0.07 0.1 0 0.04  82 0.07 0 0.05 0.1 0 0.05 0.13 0 0.05 0.09 0 0.06 0.1 0 0.04 0.1 0 0.04  83 0.08 0 0.05 0.1 0 0.06 0.13 0 0.05 0.09 0 0.06 0.11 0 0.04 0.1 0 0.04  84 0.08 0 0.05 0.1 0 0.05 0.13 0 0.05 0.09 0 0.06 0.11 0 0.04 0.11 0 0.05  85 0.08 0 0.04 0.12 0 0.05 0.13 0 0.05 0.08 0 0.06 0.11 0 0.05 0.1 0 0.04  86 0.08 0 0.04 0.11 0 0.05 0.09 0 0.05 0.08 0 0.06 0.1 0 0.04 0.09 0 0.04  87 0.06 0 0.04 0.1 0.01 0.05 0.08 0 0.04 0.09 0 0.06 0.09 0 0.04 0.1 0 0.04  88 0.06 0 0.03 0.09 0 0.04 0.1 0 0.05 0.08 0 0.06 0.1 0 0.04 0.09 0 0.04  89 0.06 0 0.03 0.1 0 0.04 0.1 0 0.05 0.08 0 0.06 0.09 0 0.04 0.1 0 0.04  90 0.06 0 0.04 0.12 0 0.04 0.1 0 0.04 0.09 0 0.06 0.09 0 0.05 0.11 0 0.04  91 0.06 0 0.03 0.12 0.02 0.04 0.1 0 0.05 0.08 0 0.06 0.09 0 0.04 0.11 0 0.05  92 0.06 0 0.04 0.13 0 0.05 0.09 0 0.05 0.08 0 0.06 0.09 0 0.04 0.11 0 0.05  93 0.06 0 0.04 0.12 0.01 0.05 0.08 0 0.05 0.08 0 0.06 0.08 0 0.04 0.11 0 0.06  94 0.06 0 0.04 0.11 0 0.05 0.08 0 0.05 0.09 0 0.06 0.09 0 0.04 0.09 0 0.06  95 0.06 0 0.04 0.1 0 0.05 0.14 0 0.06 0.09 0 0.06 0.09 0 0.04 0.1 0 0.06  96 0.06 0.02 0.04 0.11 0.01 0.05 0.13 0 0.05 0.1 0 0.06 0.08 0 0.04 0.1 0 0.04  97 0.06 0 0.04 0.11 0 0.05 0.12 0 0.05 0.08 0 0.07 0.09 0 0.05 0.09 0 0.04  98 0.07 0 0.04 0.14 0 0.05 0.14 0 0.05 0.09 0 0.07 0.1 0 0.05 0.1 0 0.04  99 0.09 0 0.04 0.15 0 0.05 0.13 0 0.05 0.09 0 0.07 0.09 0 0.04 0.1 0 0.04  100 0.11 0 0.05 0.13 0.01 0.06 0.12 0 0.05 0.09 0 0.07 0.08 0 0.04 0.1 0 0.03  101 0.11 0 0.05 0.12 0.02 0.06 0.12 0 0.05 0.11 0 0.06 0.1 0 0.04 0.1 0 0.04  102 0.08 0 0.04 0.12 0 0.05 0.1 0 0.05 0.1 0 0.06 0.09 0 0.05 0.1 0 0.04  103 0.07 0 0.04 0.14 0 0.05 0.1 0 0.05 0.09 0 0.06 0.09 0 0.04 0.11 0 0.04  104 0.08 0 0.04 0.14 0 0.06 0.09 0 0.04 0.08 0 0.06 0.09 0 0.04 0.09 0 0.03  105 0.09 0 0.04 0.13 0 0.06 0.08 0 0.05 0.09 0 0.06 0.08 0 0.04 0.1 0 0.04  106 0.09 0 0.04 0.12 0.02 0.05 0.08 0 0.05 0.09 0 0.06 0.12 0 0.06 0.11 0 0.04  107 0.1 0 0.05 0.1 0 0.05 0.1 0 0.05 0.08 0 0.06 0.1 0 0.04 0.1 0 0.05  108 0.08 0.02 0.05 0.12 0.02 0.05 0.13 0 0.06 0.08 0.01 0.06 0.09 0 0.04 0.09 0 0.04  109 0.09 0 0.05 0.1 0 0.05 0.12 0 0.05 0.09 0 0.06 0.08 0 0.04 0.1 0 0.04  110 0.07 0 0.05 0.12 0 0.05 0.12 0 0.05 0.07 0 0.06 0.09 0 0.04 0.11 0 0.04  111 0.07 0.03 0.05 0.13 0 0.05 0.12 0 0.05 0.08 0 0.06 0.09 0 0.04 0.09 0 0.03  112 0.08 0.02 0.05 0.14 0 0.05 0.11 0 0.05 0.1 0 0.06 0.08 0 0.03 0.09 0 0.04  113 0.08 0 0.05 0.14 0 0.04 0.11 0 0.05 0.1 0 0.06 0.11 0 0.04 0.1 0 0.03  114 0.06 0.01 0.04 0.13 0 0.04 0.09 0 0.06 0.1 0 0.06 0.09 0 0.04 0.11 0 0.04  115 0.07 0 0.04 0.13 0 0.05 0.09 0 0.05 0.07 0 0.04 0.08 0 0.04 0.09 0 0.04  116 0.06 0.01 0.04 0.11 0 0.04 0.09 0 0.05 0.09 0 0.06 0.1 0 0.04 0.1 0 0.03  117 0.07 0.02 0.04 0.12 0 0.05 0.12 0 0.05 0.09 0 0.06 0.1 0 0.04 0.1 0 0.03  118 0.07 0 0.03 0.12 0 0.04 0.11 0 0.05 0.09 0 0.05 0.09 0 0.04 0.09 0 0.04  119 0.06 0 0.04 0.14 0 0.05 0.11 0 0.05 0.09 0 0.05 0.09 0 0.04 0.1 0 0.04  120 0.07 0 0.04 0.12 0 0.05 0.12 0 0.05 0.12 0 0.05 0.1 0 0.04 0.08 0 0.03  121 0.07 0 0.03 0.13 0 0.05 0.1 0 0.05 0.1 0 0.05 0.09 0 0.03 0.09 0 0.03  122 0.06 0 0.03 0.12 0 0.05 0.09 0 0.04 0.1 0 0.05 0.1 0 0.04 0.1 0 0.03  123 0.06 0 0.04 0.08 0 0.05 0.1 0 0.04 0.09 0 0.05 0.1 0 0.04 0.11 0 0.04  124 0.05 0 0.03 0.13 0 0.05 0.08 0 0.04 0.09 0 0.05 0.09 0 0.04 0.09 0 0.04  125 0.05 0 0.02 0.12 0 0.05 0.08 0 0.04 0.1 0 0.05 0.09 0 0.04 0.12 0 0.03  126 0.04 0 0.02 0.1 0 0.05 0.08 0 0.04 0.09 0 0.05 0.08 0 0.03 0.11 0 0.04  127 0.04 0 0.02 0.09 0 0.05 0.08 0 0.05 0.11 0 0.05 0.09 0 0.03 0.09 0 0.04  128 0.04 0 0.02 0.12 0.02 0.05 0.08 0 0.05 0.09 0 0.05 0.08 0 0.04 0.1 0 0.04  129 0.06 0 0.03 0.11 0.02 0.05 0.11 0 0.05 0.1 0 0.05 0.08 0 0.03 0.08 0 0.04  130 0.06 0 0.04 0.13 0 0.05 0.08 0 0.05 0.1 0 0.06 0.09 0 0.04 0.1 0 0.04  131 0.06 0 0.04 0.13 0 0.05 0.09 0 0.06 0.11 0 0.06 0.07 0 0.03 0.11 0 0.05  132 0.06 0 0.04 0.14 0 0.05 0.11 0 0.05 0.1 0 0.06 0.08 0 0.04 0.09 0 0.04  133 0.07 0 0.04 0.1 0 0.05 0.11 0 0.05 0.09 0 0.06 0.09 0 0.03 0.09 0 0.05  134 0.1 0 0.04 0.13 0 0.05 0.1 0 0.05 0.12 0 0.06 0.09 0 0.04 0.1 0 0.04  135 0.1 0 0.06 0.13 0 0.05 0.12 0 0.05 0.08 0 0.06 0.09 0 0.03 0.11 0 0.04  136 0.09 0 0.05 0.12 0 0.05 0.09 0 0.05 0.1 0 0.06 0.08 0 0.03 0.09 0 0.04  137 0.11 0 0.05 0.13 0 0.05 0.1 0 0.05 0.1 0 0.05 0.08 0 0.03 0.09 0 0.04  138 0.12 0 0.05 0.13 0 0.05 0.09 0 0.05 0.1 0 0.05 0.07 0 0.03 0.1 0 0.04  139 0.1 0 0.04 0.14 0 0.05 0.1 0 0.05 0.09 0 0.05 0.06 0 0.03 0.08 0 0.04  140 0.09 0 0.04 0.14 0 0.05 0.09 0 0.05 0.11 0 0.05 0.07 0 0.03 0.11 0 0.04  141 0.08 0 0.05 0.13 0 0.04 0.12 0 0.05 0.09 0 0.05 0.07 0 0.04 0.09 0 0.04  142 0.09 0 0.05 0.15 0 0.05 0.1 0 0.05 0.09 0 0.05 0.07 0 0.03 0.1 0 0.04  143 0.08 0.02 0.05 0.11 0 0.05 0.11 0 0.05 0.09 0 0.05 0.08 0 0.03 0.09 0 0.04  144 0.09 0 0.05 0.13 0.01 0.06 0.12 0 0.05 0.1 0 0.06 0.08 0 0.04 0.1 0 0.04  145 0.09 0 0.05 0.12 0 0.05 0.09 0 0.05 0.11 0 0.06 0.09 0 0.03 0.09 0 0.04  146 0.11 0 0.05 0.13 0 0.05 0.09 0.03 0.05 0.24 0 0.06 0.06 0 0.05 0.11 0 0.04  147 0.09 0 0.05 0.1 0 0.05 0.09 0 0.05 0.18 0 0.06 0.08 0 0.04 0.09 0 0.04  148 0.1 0 0.05 0.09 0 0.05 0.1 0 0.05 0.1 0 0.06 0.08 0 0.04 0.08 0 0.04  149 0.09 0.03 0.05 0.08 0 0.05 0.1 0 0.05 0.09 0 0.06 0.09 0 0.04 0.09 0 0.04  150 0.08 0 0.05 0.1 0 0.05 0.11 0 0.04 0.08 0 0.06 0.1 0 0.04 0.09 0 0.04  151 0.08 0 0.04 0.12 0 0.05 0.11 0 0.03 0.08 0 0.06 0.09 0 0.03 0.1 0 0.04  152 0.07 0 0.04 0.11 0 0.05 0.1 0 0.03 0.07 0 0.06 0.1 0 0.04 0.1 0 0.05  153 0.11 0 0.05 0.11 0.03 0.06 0.11 0 0.04 0.09 0 0.05 9 0 0.04 0.07 0 0.04  154 0.08 0 0.04 0.11 0 0.05 0.11 0 0.04 0.08 0 0.04 0.09 0 0.04 0.1 0 0.04  155 0.07 0 0.03 0.11 0 0.05 0.11 0 0.04 0.08 0 0.04 0.09 0 0.04 0.1 0 0.04  156 0.07 0 0.03 0.09 0 0.05 0.06 0 0.04 0.08 0 0.04 0.08 0 0.03 0.09 0 0.04  157 0.06 0 0.04 0.1 0 0.05 0.07 0 0.04 0.08 0 0.05 0.09 0 0.04 0.1 0 0.05  158 0.06 0 0.04 0.09 0 0.05 0.06 0 0.04 0.09 0 0.05 0.09 0 0.04 0.1 0 0.05  159 0.06 0 0.03 0.09 0 0.05 0.11 0 0.05 0.08 0 0.04 0.08 0 0.03 0.1 0 0.04  160 0.07 0 0.04 0.09 0 0.05 0.11 0 0.06 0.09 0 0.04 0.11 0 0.04 0.09 0 0.04  161 0.08 0 0.03 0.09 0 0.05 0.08 0 0.06 0.09 0 0.03 0.09 0 0.04 0.08 0 0.05  162 0.1 0 0.04 0.1 0 0.04 0.08 0 0.07 0.08 0 0.04 0.08 0 0.04 0.09 0 0.05  163 0.09 0 0.04 0.09 0 0.04 0.12 0 0.06 0.1 0 0.04 0.1 0 0.04 0.11 0 0.05  164 0.1 0 0.05 0.1 0 0.04 0.12 0 0.05 0.08 0 0.05 0.1 0 0.04 0.09 0 0.04  165 0.11 0 0.05 0.09 0 0.04 0.11 0 0.05 0.07 0 0.04 0.09 0 0.04 0.09 0 0.04  166 0.11 0 0.05 0.09 0 0.04 0.1 0 0.05 0.09 0 0.04 0.09 0 0.04 0.08 0 0.04  167 0.09 0 0.05 0.08 0 0.03 0.1 0 0.05 0.1 0 0.04 0.09 0 0.04 0.09 0 0.05  168 0.11 0 0.05 0.11 0 0.04 0.1 0 0.04 0.1 0 0.04 0.09 0 0.05 0.08 0 0.04  169 0.09 0 0.05 0.1 0 0.04 0.1 0 0.05 0.09 0 0.04 0.09 0 0.04 0.09 0 0.04  170 0.09 0 0.06 0.12 0 0.04 0.1 0 0.04 0.1 0 0.05 0.09 0 0.05 0.1 0 0.04  171 0.09 0 0.05 0.13 0 0.05 0.06 0 0.03 0.09 0 0.04 0.09 0 0.04 0.09 0 0.04  172 0.1 0 0.05 0.1 0 0.05 0.09 0 0.04 0.09 0.01 0.06 0.08 0 0.04 0.1 0 0.05  173 0.08 0 0.05 0.09 0.1 0.05 0.11 0 0.05 0.09 0 0.06 0.08 0 0.04 0.09 0 0.04  174 0.09 0.02 0.05 0.09 0 0.05 0.11 0 0.05 0.1 0 0.06 0.07 0 0.04 0.09 0 0.04  175 0.09 0 0.06 0.11 0 0.05 0.09 0 0.04 0.08 0 0.04 0.11 0 0.04 0.09 0 0.03  176 0.1 0 0.05 0.11 0 0.04 0.08 0 0.03 0.09 0 0.04 0.09 0 0.05 0.09 0 0.03  177 0.07 0 0.05 0.1 0 0.05 0.14 0 0.05 0.08 0 0.04 0.1 0 0.04 0.09 0 0.04  178 0.08 0 0.04 0.09 0 0.05 0.08 0.03 0.05 0.11 0 0.04 0.1 0 0.04 0.09 0 0.04  179 0.07 0 0.04 0.08 0 0.05 0.08 0 0.05 0.08 0 0.04 0.1 0 0.04 0.11 0 0.04  180 0.07 0 0.04 0.09 0 0.05 0.09 0.02 0.06 0.1 0 0.04 0.09 0 0.04 0.09 0 0.04  181 0.07 0 0.04 0.08 0 0.05 0.1 0.01 0.05 0.08 0 0.03 0.09 0 0.05 0.08 0 0.04  182 0.07 0 0.03 0.09 0 0.05 0.12 0.02 0.05 0.09 0 0.04 0.09 0 0.04 0.08 0 0.03  183 0.07 0 0.04 0.07 0 0.04 0.12 0.02 0.05 0.1 0 0.04 0.1 0 0.04 0.08 0 0.04  184 0.06 0 0.04 0.08 0 0.04 0.1 0 0.05 0.11 0 0.04 0.09 0 0.05 0.09 0 0.04  185 0.06 0 0.04 0.08 0 0.05 0.12 0.01 0.05 0.1 0 0.04 0.09 0 0.04 0.1 0 0.04  186 0.07 0 0.04 0.08 0 0.05 0.11 0 0.05 0.09 0 0.04 0.1 0 0.04 0.1 0 0.04  187 0.06 0 0.04 0.09 0 0.05 0.09 0.02 0.05 0.1 0 0.05 0.12 0 0.05 0.08 0 0.04  188 0.06 0 0.03 0.09 0 0.05 0.08 0 0.05 0.11 0 0.04 0.12 0 0.04 0.1 0 0.04  189 0.07 0.02 0.04 0.1 0 0.04 0.11 0 0.06 0.12 0 0.05 0.11 0 0.04 0.08 0 0.04  190 0.06 0 0.04 0.09 0 0.04 0.12 0 0.07 0.08 0 0.04 0.1 0 0.04 0.08 0 0.04  191 0.08 0 0.05 0.1 0 0.04 0.1 0 0.06 0.09 0 0.04 0.11 0 0.04 0.09 0 0.04  192 0.11 0 0.06 0.1 0 0.05 0.1 0 0.05 0.1 0 0.04 0.12 0 0.05 0.07 0 0.03  193 0.11 0 0.06 0.13 0 0.05 0.1 0.1 0.05 0.09 0 0.04 0.11 0 0.04 0.09 0 0.04  194 0.09 0 0.05 0.13 0 0.05 0.08 0 0.03 0.1 0 0.04 0.09 0 0.03 0.08 0 0.03  195 0.11 0 0.06 0.12 0 0.05 0.08 0 0.03 0.1 0 0.04 0.09 0 0.03 0.08 0 0.03  196 0.08 0 0.05 0.13 0 0.05 0.1 0 0.04 0.11 0 0.04 0.1 0 0.03 0.08 0 0.03  197 0.09 0 0.05 0.09 0 0.05 0.09 0 0.04 0.09 0 0.04 0.1 0 0.03 0.11 0 0.04  198 0.1 0 0.06 0.09 0 0.05 0.09 0 0.03 0.1 0 0.04 0.09 0 0.04 0.07 0 0.03  199 0.11 0 0.05 0.1 0 0.05 0.08 0 0.03 0.12 0 0.04 0.1 0 0.04 0.08 0 0.03  200 0.1 0.03 0.06 0.1 0 0.04 0.08 0 0.03 0.08 0 0.04 0.1 0 0.05 0.08 0 0.03 |
| --- |

| Control (mm) Infected (mm)  Day 1 R1 R2 R3 R1 R2 R3  Max Min Average Max Min Average Max Min Average Max Min Average Max Min Average Max Min Average  1 0.04 0 0.02 0.06 0 0.03 0.05 0 0.03 0.13 0.02 0.07 0.15 0 0.06 0.09 0 0.05  2 0.04 0 0.01 0.06 0 0.03 0.05 0 0.03 0.12 0 0.06 0.15 0 0.06 0.11 0 0.05  3 0.06 0 0.02 0.06 0 0.02 0.05 0 0.02 0.14 0 0.07 0.14 0 0.06 0.09 0 0.05  4 0.05 0 0.03 0.06 0 0.03 0.06 0 0.03 0.13 0 0.06 0.13 0 0.06 0.11 0 0.05  5 0.05 0 0.03 0.06 0 0.02 0.05 0 0.01 0.14 0 0.06 0.12 0 0.06 0.11 0 0.05  6 0.05 0 0.02 0.07 0 0.02 0.07 0 0.02 0.12 0 0.06 0.12 0 0.06 0.11 0 0.05  7 0.04 0 0.02 0.07 0 0.03 0.07 0 0.02 0.11 0 0.06 0.12 0 0.06 0.1 0 0.05  8 0.04 0 0.02 0.07 0 0.02 0.07 0 0.02 0.11 0 0.06 0.13 0 0.06 0.12 0 0.05  9 0.06 0 0.03 0.06 0 0.02 0.05 0 0.02 0.12 0 0.06 0.12 0 0.07 0.12 0 0.05  10 0.05 0 0.02 0.05 0 0.03 0.04 0 0.02 0.12 0 0.06 0.13 0 0.06 0.11 0 0.06  11 0.07 0 0.02 0.06 0 0.03 0.04 0 0.02 0.12 0 0.06 0.11 0 0.06 0.12 0 0.05  12 0.05 0 0.03 0.06 0 0.02 0.07 0 0.02 0.11 0 0.06 0.11 0 0.06 0.12 0 0.05  13 0.06 0 0.02 0.05 0 0.02 0.04 0 0.02 0.13 0 0.06 0.1 0 0.06 0.12 0 0.06  14 0.05 0 0.02 0.05 0 0.02 0.04 0 0.01 0.12 0 0.05 0.13 0 0.07 0.13 0 0.06  15 0.07 0 0.03 0.04 0 0.02 0.05 0 0.03 0.11 0 0.06 0.1 0 0.06 0.14 0 0.06  16 0.05 0 0.03 0.05 0 0.02 0.07 0 0.03 0.12 0 0.05 0.11 0 0.06 0.11 0 0.06  17 0.09 0 0.02 0.06 0 0.02 0.09 0 0.03 0.11 0 0.05 0.11 0 0.06 0.12 0 0.06  18 0.01 0 0.03 0.06 0 0.02 0.07 0 0.03 0.11 0 0.05 0.1 0 0.05 0.12 0 0.06  19 0.07 0 0.03 0.05 0 0.02 0.15 0 0.03 0.13 0 0.05 0.12 0 0.07 0.13 0 0.06  20 0.04 0 0.02 0.05 0 0.02 0.05 0 0.03 0.11 0 0.05 0.11 0 0.06 0.2 0 0.06  21 0.05 0 0.02 0.04 0 0.03 0.02 0 0.04 0.12 0 0.05 0.1 0 0.06 0.12 0 0.05  22 0.05 0 0.02 0.07 0 0.03 0.19 0 0.03 0.11 0 0.05 0.13 0 0.06 0.12 0 0.05  23 0.05 0 0.02 0.08 0 0.03 0.04 0 0.02 0.13 0 0.06 0.1 0 0.06 0.12 0 0.05  24 0.06 0 0.02 0.06 0 0.02 0.11 0 0.02 0.13 0 0.06 0.12 0 0.05 0.1 0 0.05  25 0.05 0 0.02 0.06 0 0.02 0.13 0 0.02 0.13 0 0.06 0.11 0 0.06 0.12 0 0.06  26 0.05 0 0.03 0.06 0 0.02 0.15 0 0.03 0.16 0 0.06 0.23 0 0.07 0.11 0 0.05  27 0.05 0 0.03 0.07 0 0.03 0.09 0 0.03 0.16 0 0.06 0.1 0 0.06 0.19 0 0.05  28 0.05 0 0.03 0.06 0 0.02 0.17 0 0.03 0.16 0 0.07 0.13 0 0.05 0.22 0 0.06  29 0.07 0 0.03 0.08 0 0.02 0.05 0 0.02 0.15 0 0.06 0.16 0 0.06 0.13 0 0.04  30 0.07 0 0.03 0.05 0 0.03 0.04 0 0.02 0.17 0 0.06 0.09 0 0.05 0.1 0 0.04  31 0.06 0 0.02 0.05 0 0.02 0.05 0 0.03 0.14 0 0.06 0.13 0 0.06 0.13 0 0.05  32 0.07 0 0.02 0.05 0 0.03 0.06 0 0.02 0.13 0 0.06 0.14 0 0.06 0.1 0 0.05  33 0.04 0 0.02 0.06 0 0.03 0.06 0 0.03 0.16 0 0.05 0.13 0 0.06 0.1 0 0.04  34 0.05 0 0.02 0.06 0 0.04 0.07 0 0.03 0.14 0 0.05 0.13 0 0.06 0.13 0 0.06  35 0.05 0 0.02 0.05 0 0.03 0.07 0 0.03 0.13 0 0.05 0.12 0 0.06 0.22 0 0.06  36 0.06 0 0.03 0.05 0 0.03 0.08 0 0.03 0.15 0 0.05 0.11 0 0.05 0.11 0 0.06  37 0.05 0 0.03 0.08 0 0.02 0.08 0 0.03 0.15 0 0.06 0.12 0 0.06 0.14 0 0.06  38 0.05 0 0.03 0.08 0 0.03 0.06 0 0.03 0.13 0 0.05 0.12 0 0.05 0.13 0 0.06  39 0.06 0 0.03 0.08 0 0.03 0.07 0 0.03 0.16 0 0.06 0.11 0 0.05 0.12 0 0.06  40 0.05 0 0.02 0.07 0 0.03 0.06 0 0.03 0.14 0 0.05 0.12 0 0.05 0.12 0 0.07  41 0.06 0 0.03 0.08 0 0.02 0.04 0 0.02 0.15 0 0.05 0.11 0 0.07 0.12 0 0.07  42 0.07 0 0.03 0.07 0 0.03 0.09 0 0.02 0.12 0 0.06 0.12 0 0.06 0.14 0 0.06  43 0.08 0 0.03 0.08 0 0.04 0.05 0 0.02 0.12 0 0.06 0.12 0 0.06 0.14 0 0.06  44 0.06 0 0.03 0.08 0 0.04 0.05 0 0.02 0.13 0 0.06 0.14 0 0.07 0.12 0 0.06  45 0.06 0 0.03 0.07 0 0.03 0.03 0 0.01 0.27 0 0.08 0.12 0 0.06 0.11 0 0.06  46 0.06 0 0.03 0.09 0 0.03 0.04 0 0.03 0.11 0 0.06 0.17 0 0.06 0.11 0 0.06  47 0.06 0 0.03 0.09 0 0.04 0.05 0 0.02 0.11 0 0.06 0.12 0 0.06 0.14 0 0.06  48 0.06 0 0.03 0.09 0 0.03 0.05 0 0.02 0.13 0 0.06 0.15 0 0.06 0.15 0 0.06  49 0.05 0 0.03 0.08 0 0.03 0.05 0 0.02 0.12 0 0.07 0.11 0 0.06 0.14 0 0.05  50 0.06 0 0.03 0.06 0 0.03 0.04 0 0.02 0.13 0 0.07 0.15 0 0.05 0.13 0 0.05  51 0.06 0 0.03 0.06 0 0.03 0.03 0 0.01 0.14 0 0.06 0.12 0 0.05 0.13 0 0.06  52 0.07 0 0.03 0.05 0 0.02 0.03 0 0.01 0.14 0 0.06 0.11 0 0.06 0.11 0 0.06  53 0.06 0 0.03 0.06 0 0.03 0.04 0 0.02 0.12 0 0.06 0.1 0 0.06 0.14 0 0.06  54 0.05 0 0.03 0.06 0 0.03 0.03 0 0.01 0.11 0 0.06 0.15 0 0.06 0.22 0 0.06  55 0.07 0 0.02 0.06 0 0.03 0.04 0 0.02 0.12 0 0.06 0.16 0 0.07 0.14 0 0.06  56 0.08 0 0.02 0.06 0 0.02 0.03 0 0.02 0.12 0 0.06 0.14 0 0.07 0.15 0 0.06  57 0.06 0 0.03 0.07 0 0.03 0.05 0 0.02 0.13 0 0.06 0.23 0 0.06 0.13 0 0.06  58 0.08 0 0.03 0.05 0 0.02 0.08 0 0.04 0.11 0 0.06 0.14 0 0.07 0.18 0 0.05  59 0.07 0 0.02 0.06 0 0.02 0.09 0 0.05 0.12 0 0.06 0.17 0 0.07 0.15 0 0.07  60 0.08 0 0.03 0.05 0 0.02 0.09 0 0.05 0.12 0 0.06 0.15 0 0.06 0.15 0 0.06  61 0.06 0 0.03 0.06 0 0.03 0.08 0 0.04 0.12 0 0.06 0.13 0 0.06 0.13 0 0.07  62 0.08 0 0.03 0.07 0 0.03 0.08 0 0.04 0.12 0 0.06 0.12 0 0.05 0.13 0 0.06  63 0.07 0 0.03 0.08 0 0.03 0.08 0 0.04 0.13 0 0.06 0.11 0 0.06 0.15 0 0.07  64 0.08 0 0.03 0.07 0 0.03 0.07 0 0.04 0.11 0 0.06 0.14 0 0.05 0.12 0 0.07  65 0.08 0 0.04 0.07 0 0.02 0.07 0 0.04 0.1 0 0.06 0.14 0 0.06 0.15 0 0.07  66 0.08 0 0.03 0.07 0 0.02 0.06 0 0.04 0.12 0 0.06 0.14 0 0.06 0.16 0 0.07  67 0.11 0 0.03 0.07 0 0.03 0.07 0 0.04 0.13 0 0.06 0.21 0 0.06 0.27 0 0.07  68 0.11 0 0.04 0.06 0 0.03 0.07 0.02 0.03 0.13 0 0.06 0.13 0 0.05 0.16 0 0.07  69 0.1 0 0.03 0.07 0 0.03 0.06 0 0.03 0.14 0 0.06 0.13 0 0.05 0.14 0.02 0.07  70 0.11 0 0.04 0.06 0 0.02 0.06 0 0.03 0.14 0 0.05 0.14 0 0.06 0.12 0 0.06  71 0.09 0 0.04 0.05 0 0.02 0.05 0 0.03 0.1 0 0.05 0.13 0 0.06 0.12 0 0.06  72 0.09 0 0.04 0.08 0 0.02 0.04 0 0.02 0.13 0 0.05 0.12 0 0.06 0.14 0 0.06  73 0.1 0 0.03 0.07 0 0.03 0.04 0 0.02 0.12 0 0.06 0.11 0 0.06 0.12 0 0.06  74 0.09 0 0.03 0.06 0 0.03 0.04 0 0.01 0.12 0 0.06 0.13 0 0.06 0.12 0 0.06  75 0.09 0 0.04 0.08 0 0.03 0.04 0 0.02 0.11 0 0.06 0.12 0 0.06 0.13 0 0.06  76 0.1 0 0.04 0.08 0 0.03 0.04 0 0.02 0.15 0 0.06 0.11 0 0.05 0.11 0.02 0.06  77 0.09 0 0.03 0.07 0 0.03 0.04 0 0.02 0.15 0 0.06 0.12 0 0.05 0.1 0 0.06  78 0.11 0 0.04 0.08 0 0.03 0.04 0 0.01 0.12 0 0.06 0.12 0 0.05 0.1 0 0.06  79 0.11 0 0.04 0.07 0 0.03 0.06 0 0.02 0.13 0 0.06 0.15 0 0.06 0.13 0 0.05  80 0.09 0 0.04 0.09 0 0.03 0.09 0 0.04 0.11 0 0.06 0.15 0 0.06 0.11 0 0.05  81 0.08 0 0.03 0.08 0 0.03 0.08 0 0.05 0.11 0 0.05 0.76 0 0.13 0.1 0 0.06  82 0.08 0 0.03 0.09 0 0.03 0.09 0 0.04 0.13 0 0.06 0.13 0 0.06 0.11 0 0.06  83 0.09 0 0.03 0.1 0 0.03 0.07 0 0.03 0.15 0 0.07 0.11 0 0.06 0.1 0 0.06  84 0.11 0 0.04 0.09 0 0.03 0.08 0 0.03 0.13 0 0.06 0.12 0 0.05 0.2 0 0.07  85 0.1 0 0.04 0.07 0 0.03 0.09 0 0.03 0.12 0 0.06 0.11 0 0.06 0.12 0.01 0.06  86 0.08 0 0.03 0.07 0 0.03 0.08 0 0.03 0.11 0 0.06 0.12 0 0.06 0.11 0.03 0.06  87 0.09 0 0.03 0.06 0 0.03 0.08 0 0.04 0.11 0 0.06 0.13 0 0.06 0.11 0 0.06  88 0.09 0 0.03 0.07 0 0.03 0.13 0 0.04 0.12 0 0.06 0.12 0 0.06 0.12 0 0.06  89 0.09 0 0.04 0.07 0 0.03 0.09 0 0.03 0.13 0 0.06 0.13 0 0.06 0.13 0 0.05  90 0.09 0 0.04 0.08 0 0.03 0.06 0 0.01 0.12 0 0.06 0.11 0 0.06 0.11 0 0.06  91 0.13 0 0.04 0.07 0 0.04 0.03 0 0.01 0.15 0 0.06 0.11 0 0.06 0.12 0 0.06  92 0.13 0 0.04 0.08 0 0.04 0.04 0 0.02 0.15 0 0.06 0.13 0 0.06 0.12 0 0.06  93 0.14 0 0.05 0.07 0 0.03 0.04 0 0.02 0.14 0 0.06 0.13 0 0.06 0.13 0 0.06  94 0.14 0 0.05 0.06 0 0.03 0.04 0 0.02 0.14 0 0.05 0.11 0 0.06 0.11 0 0.06  95 0.14 0 0.03 0.08 0 0.04 0.03 0 0.01 0.15 0 0.06 0.1 0 0.06 0.26 0 0.07  96 0.12 0 0.04 0.07 0 0.04 0.04 0 0.02 0.12 0 0.06 0.09 0 0.06 0.13 0 0.07  97 0.11 0 0.04 0.11 0 0.06 0.04 0 0.01 0.1 0 0.06 0.1 0 0.06 0.12 0 0.06  98 0.1 0 0.04 0.15 0 0.06 0.03 0 0.02 0.14 0 0.05 0.09 0 0.05 0.14 0 0.06  99 0.09 0 0.04 0.08 0 0.04 0.05 0 0.03 0.11 0 0.06 0.09 0 0.05 0.13 0 0.05  100 0.12 0 0.04 0.08 0 0.03 0.05 0 0.03 0.1 0 0.05 0.25 0 0.06 0.13 0 0.05  101 0.11 0 0.04 0.07 0 0.04 0.06 0 0.03 0.12 0 0.06 0.11 0 0.05 0.11 0 0.06  102 0.12 0 0.04 0.07 0 0.04 0.06 0 0.03 0.11 0 0.05 0.28 0 0.07 0.15 0 0.06  103 0.11 0 0.04 0.08 0 0.04 0.07 0 0.04 0.1 0 0.06 0.12 0 0.06 0.11 0 0.05  104 0.11 0 0.04 0.07 0 0.04 0.07 0 0.05 0.12 0 0.06 0.09 0 0.05 0.11 0 0.05  105 0.11 0 0.05 0.07 0 0.04 0.09 0.02 0.04 0.12 0 0.06 0.14 0 0.06 0.12 0 0.05  106 0.09 0 0.04 0.08 0 0.04 0.06 0.01 0.04 0.14 0 0.06 0.13 0 0.05 0.12 0 0.05  107 0.1 0 0.04 0.09 0 0.04 0.06 0 0.03 0.12 0 0.06 0.12 0 0.05 0.13 0 0.06  108 0.09 0 0.04 0.09 0 0.04 0.07 0.01 0.04 0.11 0 0.06 0.12 0 0.05 0.13 0 0.06  109 0.08 0 0.04 0.08 0 0.04 0.05 0 0.04 0.13 0 0.06 0.11 0 0.05 0.11 0 0.06  110 0.08 0 0.04 0.09 0 0.04 0.08 0 0.04 0.12 0 0.06 0.14 0 0.05 0.14 0 0.06  111 0.09 0 0.04 0.09 0 0.04 0.1 0 0.04 0.12 0 0.05 0.13 0 0.05 0.11 0 0.07  112 0.09 0 0.04 0.09 0 0.04 0.1 0 0.04 0.1 0 0.05 0.13 0 0.05 0.12 0 0.05  113 0.09 0 0.04 0.09 0 0.04 0.7 0.01 0.04 0.13 0 0.06 0.16 0 0.06 0.12 0 0.06  114 0.11 0 0.04 0.09 0 0.04 0.05 0 0.03 0.12 0 0.05 0.12 0 0.06 0.11 0 0.05  115 0.11 0 0.04 0.11 0 0.04 0.06 0 0.02 0.09 0 0.05 0.13 0 0.05 0.11 0 0.05  116 0.1 0 0.04 0.08 0 0.04 0.07 0 0.03 0.11 0 0.05 0.14 0 0.05 0.1 0 0.04  117 0.09 0 0.04 0.09 0 0.04 0.07 0 0.03 0.12 0 0.04 0.11 0 0.06 0.11 0 0.05  118 0.09 0 0.04 0.1 0 0.04 0.05 0 0.02 0.12 0 0.04 0.11 0 0.06 0.1 0 0.05  119 0.09 0 0.05 0.1 0 0.05 0.05 0 0.01 0.11 0 0.05 0.22 0 0.06 0.1 0 0.05  120 0.08 0 0.04 0.09 0 0.04 0.05 0 0.02 0.11 0 0.05 0.1 0 0.05 0.6 0 0.1  121 0.09 0 0.04 0.08 0 0.04 0.06 0 0.03 0.11 0 0.05 0.11 0 0.06 0.13 0 0.06  122 0.1 0 0.05 0.09 0 0.04 0.06 0 0.03 0.14 0 0.05 0.12 0 0.05 0.09 0 0.05  123 0.14 0 0.05 0.09 0 0.04 0.09 0 0.04 0.13 0 0.05 0.11 0 0.05 0.12 0 0.06  124 0.13 0 0.05 0.09 0 0.04 0.12 0 0.05 0.13 0 0.05 0.15 0.01 0.06 0.11 0 0.06  125 0.13 0 0.04 0.1 0 0.04 0.12 0 0.05 0.12 0 0.05 0.12 0 0.06 0.1 0 0.06  126 0.13 0 0.05 0.1 0 0.05 0.11 0 0.04 0.11 0 0.05 0.14 0 0.05 0.11 0 0.06  127 0.12 0 0.05 0.07 0 0.04 0.07 0 0.03 0.12 0 0.05 0.09 0 0.05 0.11 0 0.06  128 0.11 0 0.05 0.09 0 0.04 0.08 0 0.03 0.13 0 0.05 0.09 0 0.05 0.09 0 0.06  129 0.12 0 0.05 0.09 0 0.04 0.07 0 0.04 0.1 0 0.05 0.1 0 0.05 0.11 0 0.05  130 0.12 0 0.05 0.09 0 0.04 0.07 0 0.04 0.12 0 0.05 0.11 0 0.06 0.11 0 0.06  131 0.1 0 0.05 0.09 0 0.04 0.07 0 0.04 0.1 0 0.04 0.09 0 0.06 0.74 0 0.08  132 0.12 0 0.05 0.08 0 0.03 0.04 0 0.03 0.1 0 0.04 0.11 0 0.06 0.1 0 0.06  133 0.1 0 0.05 0.1 0 0.04 0.05 0 0.02 0.08 0 0.04 0.1 0 0.06 0.1 0 0.06  134 0.11 0 0.05 0.09 0 0.04 0.05 0 0.03 0.12 0 0.05 0.1 0 0.06 0.13 0 0.06  135 0.13 0 0.05 0.09 0 0.04 0.05 0 0.04 0.1 0 0.05 0.13 0 0.06 0.1 0 0.05  136 0.12 0 0.05 0.09 0 0.04 0.05 0 0.03 0.22 0 0.05 0.1 0 0.06 0.26 0 0.07  137 0.11 0 0.05 0.11 0 0.04 0.05 0 0.03 0.12 0 0.05 0.1 0 0.05 0.11 0 0.06  138 0.11 0 0.05 0.1 0 0.04 0.04 0 0.02 0.12 0 0.05 0.13 0 0.06 0.11 0 0.05  139 0.1 0 0.04 0.12 0 0.04 0.04 0 0.02 0.28 0 0.08 0.12 0 0.05 0.1 0 0.05  140 0.12 0 0.05 0.11 0 0.04 0.04 0 0.03 0.25 0 0.06 0.11 0 0.05 0.11 0 0.05  141 0.12 0 0.05 0.12 0 0.05 0.04 0 0.02 0.09 0 0.04 0.12 0 0.05 0.09 0 0.05  142 0.11 0 0.05 0.11 0 0.05 0.03 0 0.01 0.09 0 0.05 0.11 0 0.05 0.1 0 0.05  143 0.12 0 0.05 0.1 0 0.04 0.04 0 0.02 0.08 0 0.04 0.11 0 0.05 0.1 0 0.05  144 0.11 0 0.05 0.1 0 0.05 0.04 0 0.02 0.09 0 0.05 0.09 0 0.05 0.11 0 0.05  145 0.12 0 0.06 0.1 0 0.06 0.04 0 0.02 0.11 0 0.05 0.11 0 0.04 0.1 0 0.04  146 0.12 0 0.05 0.15 0 0.06 0.04 0 0.02 0.08 0 0.04 0.12 0 0.04 0.1 0 0.05  147 0.13 0 0.05 0.11 0 0.05 0.06 0 0.03 0.09 0 0.05 0.13 0 0.05 0.1 0 0.06  148 0.11 0 0.05 0.09 0 0.04 0.05 0 0.03 0.08 0 0.04 0.21 0 0.06 0.09 0 0.05  149 0.11 0 0.05 0.11 0 0.04 0.06 0 0.02 0.08 0 0.03 0.09 0 0.04 0.1 0 0.05  150 0.12 0 0.05 0.1 0 0.04 0.07 0 0.02 0.08 0 0.04 0.09 0 0.05 0.81 0 0.06  151 0.12 0 0.05 0.1 0 0.06 0.12 0 0.06 0.09 0 0.04 0.11 0 0.05 0.13 0 0.06  152 0.11 0 0.05 0.1 0 0.06 0.13 0 0.07 0.18 0 0.04 0.12 0 0.04 0.09 0 0.05  153 0.12 0.02 0.06 0.09 0 0.04 0.14 0 0.07 0.11 0 0.04 0.12 0 0.05 0.08 0 0.05  154 0.11 0 0.05 0.09 0 0.04 0.12 0 0.06 0.09 0 0.04 0.08 0 0.04 0.14 0 0.05  155 0.11 0 0.05 0.09 0 0.04 0.11 0 0.06 0.1 0 0.05 0.08 0 0.04 0.1 0 0.05  156 0.1 0 0.05 0.18 0 0.04 0.09 0 0.06 0.1 0 0.05 0.09 0 0.04 0.18 0 0.05  157 0.11 0 0.05 0.09 0 0.04 0.1 0 0.06 0.1 0 0.05 0.1 0 0.04 0.15 0 0.05  158 0.11 0 0.05 0.1 0 0.04 0.08 0 0.06 0.11 0 0.05 0.09 0 0.05 0.11 0 0.05  159 0.11 0 0.05 0.1 0 0.04 0.07 0 0.04 0.11 0 0.05 0.13 0 0.04 0.14 0 0.05  160 0.11 0 0.05 0.11 0 0.04 0.07 0.03 0.04 0.11 0 0.05 0.1 0 0.05 0.1 0 0.05  161 0.12 0 0.04 0.1 0 0.04 0.08 0 0.04 0.1 0 0.04 0.11 0 0.04 0.15 0 0.06  162 0.1 0 0.05 0.1 0 0.04 0.07 0 0.03 0.12 0 0.05 0.1 0 0.04 0.12 0 0.06  163 0.12 0 0.04 0.1 0 0.04 0.06 0 0.03 0.1 0 0.04 0.12 0 0.05 0.11 0 0.06  164 0.12 0 0.05 0.11 0 0.04 0.06 0 0.02 0.1 0 0.05 0.11 0 0.04 0.1 0 0.05  165 0.12 0 0.05 0.09 0 0.04 0.06 0 0.02 0.11 0 0.05 0.12 0 0.05 0.1 0 0.05  166 0.12 0 0.05 0.1 0 0.04 0.06 0 0.03 0.09 0 0.04 0.14 0 0.05 0.1 0 0.05  167 0.12 0 0.05 0.09 0 0.04 0.04 0 0.02 0.9 0 0.13 0.1 0 0.05 0.1 0 0.05  168 0.1 0 0.05 0.1 0 0.04 0.04 0 0.02 0.85 0 0.09 0.1 0 0.05 0.11 0 0.05  169 0.11 0 0.05 0.1 0 0.04 0.05 0 0.03 0.17 0 0.05 0.1 0 0.04 0.1 0 0.05  170 0.1 0 0.05 0.11 0 0.05 0.04 0 0.02 0.17 0 0.06 0.12 0 0.05 0.09 0 0.05  171 0.11 0 0.05 0.12 0 0.05 0.06 0 0.03 0.24 0 0.07 0.1 0 0.04 0.1 0 0.05  172 0.12 0 0.05 0.12 0 0.06 0.09 0 0.04 0.17 0 0.05 0.11 0 0.04 0.1 0 0.04  173 0.11 0 0.05 0.13 0 0.06 0.1 0 0.07 0.17 0 0.05 0.1 0 0.04 0.09 0 0.04  174 0.1 0 0.05 0.12 0 0.06 0.1 0 0.07 0.11 0 0.05 0.11 0 0.04 0.1 0 0.04  175 0.1 0 0.05 0.11 0 0.06 0.11 0 0.05 0.13 0 0.05 0.09 0 0.04 0.09 0 0.04  176 0.09 0 0.04 0.1 0 0.06 0.11 0 0.05 0.11 0 0.05 0.1 0 0.04 0.1 0 0.05  177 0.1 0 0.04 0.15 0 0.06 0.08 0 0.04 0.09 0 0.04 0.08 0 0.04 0.09 0 0.04  178 0.1 0 0.05 0.09 0 0.04 0.07 0 0.04 0.11 0 0.04 0.09 0 0.04 0.22 0 0.04  179 0.1 0 0.05 0.1 0 0.04 0.07 0 0.04 0.1 0 0.05 0.11 0 0.04 0.24 0 0.06  180 0.11 0 0.05 0.11 0 0.05 0.07 0.02 0.05 0.09 0 0.05 0.11 0 0.04 0.08 0 0.04  181 0.09 0 0.05 0.11 0 0.04 0.07 0.01 0.04 0.09 0 0.05 0.09 0 0.03 0.1 0 0.04  182 0.09 0 0.04 0.1 0 0.05 0.07 0 0.04 0.09 0 0.04 0.11 0 0.04 0.09 0 0.04  183 0.1 0 0.05 0.09 0 0.05 0.07 0 0.04 0.08 0 0.05 0.13 0 0.04 0.1 0 0.04  184 0.1 0 0.04 0.09 0 0.04 0.06 0 0.03 0.11 0 0.04 0.12 0 0.05 0.09 0 0.05  185 0.12 0 0.06 0.12 0 0.04 0.05 0 0.02 0.12 0 0.05 0.1 0 0.05 0.09 0 0.05  186 0.11 0 0.06 0.09 0 0.04 0.04 0 0.02 0.1 0 0.05 0.09 0 0.04 0.1 0 0.04  187 0.1 0 0.05 0.12 0 0.04 0.04 0 0.02 0.11 0 0.05 0.12 0 0.05 0.1 0 0.04  188 0.1 0 0.04 0.11 0 0.04 0.04 0 0.01 0.13 0 0.06 0.11 0 0.05 0.12 0 0.04  189 0.04 0 0.04 0.12 0 0.04 0.03 0 0.01 0.13 0 0.05 0.1 0 0.05 0.13 0 0.05  190 0.1 0 0.05 0.11 0 0.04 0.03 0 0.01 0.1 0 0.05 0.12 0 0.06 0.13 0 0.04  191 0.09 0 0.05 0.1 0 0.05 0.06 0 0.03 0.12 0 0.05 0.11 0 0.06 0.13 0 0.04  192 0.1 0 0.04 0.11 0 0.04 0.08 0 0.03 0.12 0 0.05 0.12 0 0.04 0.14 0 0.05  193 0.1 0 0.04 0.12 0 0.05 0.03 0 0.04 0.1 0 0.05 0.14 0 0.05 0.14 0 0.04  194 0.09 0 0.04 0.11 0 0.05 0.07 0 0.04 0.1 0 0.05 0.13 0 0.05 0.12 0 0.04  195 0.09 0 0.05 0.12 0 0.04 0.08 0 0.05 0.09 0 0.05 0.12 0 0.05 0.16 0 0.04  196 0.1 0 0.04 0.12 0 0.05 0.09 0 0.06 0.1 0 0.05 0.14 0 0.05 0.15 0 0.04  197 0.1 0 0.05 0.1 0 0.06 0.1 0.02 0.06 0.12 0 0.06 0.14 0 0.05 0.15 0 0.04  198 0.1 0 0.04 0.15 0 0.06 0.01 0 0.04 0.12 0 0.06 0.18 0 0.06 0.12 0 0.04  199 0.1 0 0.04 0.12 0 0.06 0.09 0 0.04 0.1 0 0.05 0.13 0 0.06 0.09 0 0.04  200 0.09 0 0.04 0.11 0 0.06 0.08 0.02 0.04 0.09 0 0.05 0.13 0 0.05 0.14 0 0.05 |
| --- |

| Control (mm) Infected (mm)  Day 2 R1 R2 R3 R1 R2 R3  Max Min Average Max Min Average Max Min Average Max Min Average Max Min Average Max Min Average  1 0.06 0 0.03 0.06 0 0.02 0.06 0 0.02 0.04 0 0.03 0.12 0 0.06 0.11 0 0.08  2 0.06 0 0.03 0.06 0 0.02 0.04 0 0.03 0.06 0 0.03 0.15 0 0.06 0.12 0 0.07  3 0.07 0 0.03 0.05 0 0.02 0.06 0 0.04 0.04 0 0.02 0.14 0 0.06 0.1 0 0.07  4 0.06 0 0.03 0.04 0 0.02 0.05 0 0.03 0.03 0 0.01 0.14 0 0.06 0.1 0 0.07  5 0.06 0 0.03 0.07 0 0.03 0.04 0 0.02 0.1 0 0.03 0.12 0 0.06 0.11 0 0.07  6 0.06 0 0.03 0.06 0 0.03 0.04 0 0.02 0.03 0 0.01 0.12 0 0.06 0.12 0 0.07  7 0.08 0 0.03 0.06 0 0.03 0.06 0 0.03 0.03 0 0.01 0.14 0 0.06 0.12 0 0.07  8 0.08 0 0.04 0.06 0 0.03 0.05 0 0.02 0.03 0 0.01 0.15 0 0.06 0.11 0 0.05  9 0.07 0 0.04 0.06 0 0.03 0.07 0 0.03 0.03 0 0.01 0.14 0 0.05 0.12 0 0.05  10 0.07 0 0.03 0.05 0 0.03 0.08 0 0.03 0.02 0 0.04 0.13 0 0.06 0.12 0 0.05  11 0.07 0 0.03 0.05 0 0.03 0.08 0 0.04 0.02 0 0.01 0.16 0 0.06 0.1 0 0.05  12 0.08 0 0.04 0.06 0 0.03 0.08 0 0.04 0.02 0 0.05 0.11 0 0.05 0.1 0 0.05  13 0.06 0 0.03 0.07 0 0.02 0.07 0 0.03 0.02 0 0.06 0.11 0 0.05 0.11 0 0.07  14 0.07 0 0.04 0.08 0 0.03 0.07 0 0.03 0.01 0 0.06 0.12 0 0.05 0.1 0 0.07  15 0.08 0 0.04 0.06 0 0.03 0.08 0 0.03 0.02 0 0.06 0.12 0 0.05 0.1 0 0.07  16 0.01 0 0.04 0.06 0 0.03 0.08 0 0.04 0.02 0 0.06 0.11 0 0.05 0.1 0 0.07  17 0.07 0 0.04 0.07 0 0.02 0.09 0 0.03 0.03 0 0.06 0.11 0 0.05 0.11 0 0.07  18 0.07 0 0.03 0.08 0 0.03 0.08 0 0.03 0.05 0 0.06 0.12 0 0.06 0.09 0 0.06  19 0.07 0 0.04 0.08 0 0.03 0.08 0 0.03 0.06 0 0.06 0.11 0 0.05 0.1 0 0.06  20 0.07 0 0.04 0.07 0 0.03 0.06 0 0.03 0.06 0 0.06 0.12 0 0.05 0.11 0 0.06  21 0.07 0 0.03 0.07 0 0.03 0.07 0 0.04 0.07 0 0.06 0.11 0 0.05 0.1 0 0.06  22 0.07 0 0.03 0.06 0 0.03 0.06 0 0.03 0.08 0 0.06 0.12 0 0.07 0.1 0 0.06  23 0.06 0 0.03 0.06 0 0.03 0.06 0 0.02 0.08 0 0.06 0.12 0 0.07 0.1 0 0.06  24 0.06 0 0.03 0.07 0 0.03 0.06 0 0.03 0.07 0 0.03 0.11 0 0.07 0.09 0 0.06  25 0.07 0 0.03 0.08 0 0.04 0.04 0 0.02 0.08 0 0.03 0.12 0 0.06 0.11 0 0.06  26 0.07 0 0.03 0.08 0 0.04 0.06 0 0.03 0.07 0 0.02 0.1 0 0.06 0.12 0 0.06  27 0.06 0 0.04 0.07 0 0.04 0.07 0 0.03 0.08 0 0.03 0.12 0 0.06 0.12 0 0.06  28 0.09 0 0.04 0.07 0 0.03 0.06 0 0.03 0.08 0 0.02 0.12 0 0.06 0.11 0 0.06  29 0.08 0 0.04 0.07 0 0.04 0.07 0 0.03 0.07 0 0.03 0.13 0 0.06 0.11 0 0.06  30 0.08 0 0.04 0.08 0 0.04 0.06 0 0.03 0.08 0 0.06 0.1 0 0.06 0.13 0 0.06  31 0.07 0 0.04 0.07 0 0.04 0.06 0 0.03 0.08 0 0.06 0.1 0 0.06 0.13 0 0.07  32 0.07 0 0.04 0.07 0 0.04 0.07 0 0.03 0.07 0 0.03 0.09 0 0.05 0.13 0 0.07  33 0.07 0 0.03 0.07 0 0.04 0.06 0 0.03 0.07 0 0.04 0.09 0 0.05 0.14 0 0.07  34 0.08 0 0.04 0.07 0 0.04 0.04 0 0.02 0.08 0 0.04 0.1 0 0.06 0.13 0 0.08  35 0.06 0 0.04 0.07 0 0.03 0.05 0 0.02 0.09 0 0.03 0.11 0 0.06 0.12 0.02 0.07  36 0.08 0 0.04 0.07 0 0.03 0.04 0 0.02 0.1 0 0.06 0.1 0 0.05 0.12 0.02 0.07  37 0.07 0 0.04 0.07 0 0.03 0.05 0 0.02 0.09 0.01 0.06 0.11 0 0.05 0.12 0 0.07  38 0.08 0 0.03 0.06 0 0.03 0.05 0 0.02 0.08 0 0.06 0.11 0 0.05 0.11 0.01 0.07  39 0.06 0 0.03 0.06 0 0.03 0.06 0 0.02 0.12 0 0.06 0.1 0 0.05 0.12 0 0.07  40 0.05 0 0.03 0.06 0 0.03 0.05 0 0.03 0.1 0 0.06 0.1 0 0.05 0.12 0 0.07  41 0.08 0 0.04 0.07 0 0.03 0.05 0 0.03 0.09 0 0.04 0.11 0 0.05 0.1 0.02 0.07  42 0.06 0 0.03 0.07 0 0.04 0.04 0 0.03 0.11 0 0.06 0.1 0 0.06 0.12 0 0.07  43 0.07 0 0.04 0.06 0 0.03 0.05 0 0.02 0.12 0 0.06 0.11 0 0.06 0.11 0.01 0.07  44 0.07 0 0.04 0.07 0 0.03 0.06 0 0.03 0.09 0 0.06 0.1 0 0.05 0.12 0.03 0.07  45 0.07 0 0.03 0.08 0 0.04 0.06 0 0.03 0.12 0 0.05 0.09 0 0.05 0.12 0 0.07  46 0.08 0 0.04 0.07 0 0.03 0.06 0 0.03 0.07 0 0.03 0.1 0 0.06 0.11 0 0.07  47 0.07 0 0.04 0.08 0 0.03 0.06 0 0.03 0.06 0 0.03 0.1 0 0.06 0.11 0 0.07  48 0.07 0 0.04 0.07 0 0.03 0.07 0 0.03 0.05 0 0.02 0.1 0 0.07 0.1 0 0.06  49 0.08 0 0.03 0.05 0 0.02 0.06 0 0.03 0.06 0 0.03 0.12 0 0.07 0.11 0 0.06  50 0.08 0 0.03 0.05 0 0.02 0.06 0 0.03 0.08 0 0.02 0.11 0 0.07 0.12 0.02 0.07  51 0.08 0 0.03 0.06 0 0.02 0.07 0 0.03 0.09 0 0.03 0.12 0 0.07 0.11 0 0.06  52 0.07 0 0.03 0.06 0 0.02 0.05 0 0.03 0.08 0 0.03 0.12 0 0.07 0.11 0 0.07  53 0.08 0 0.03 0.05 0 0.03 0.07 0 0.03 0.08 0 0.04 0.11 0 0.06 0.11 0 0.07  54 0.08 0 0.03 0.04 0 0.02 0.07 0 0.03 0.08 0 0.04 0.11 0 0.07 0.11 0 0.06  55 0.07 0 0.03 0.19 0 0.03 0.06 0 0.03 0.08 0 0.03 0.12 0 0.07 0.11 0 0.07  56 0.09 0 0.04 0.2 0 0.03 0.06 0 0.03 0.1 0 0.04 0.13 0 0.07 0.11 0 0.07  57 0.08 0 0.04 0.06 0 0.03 0.06 0 0.02 0.1 0 0.04 0.11 0 0.06 0.14 0 0.07  58 0.09 0 0.04 0.06 0 0.03 0.05 0 0.02 0.09 0 0.04 0.12 0 0.07 0.12 0 0.06  59 0.09 0 0.04 0.05 0 0.03 0.05 0 0.02 0.1 0 0.04 0.1 0 0.07 0.11 0 0.07  60 0.09 0 0.03 0.05 0 0.03 0.08 0 0.03 0.09 0 0.06 0.12 0 0.07 0.11 0 0.07  61 0.09 0 0.04 0.06 0 0.03 0.06 0 0.02 0.09 0 0.04 0.11 0 0.07 0.12 0 0.07  62 0.08 0 0.03 0.08 0 0.03 0.07 0 0.03 0.1 0 0.03 0.12 0 0.07 0.11 0 0.07  63 0.08 0 0.03 0.07 0 0.03 0.08 0 0.03 0.1 0 0.03 0.11 0 0.07 0.13 0 0.07  64 0.08 0 0.03 0.08 0 0.04 0.09 0 0.03 0.08 0 0.03 0.11 0 0.06 0.11 0 0.07  65 0.07 0 0.03 0.07 0 0.03 0.1 0 0.03 0.09 0 0.03 0.11 0 0.06 0.12 0 0.07  66 0.06 0 0.03 0.07 0 0.03 0.09 0 0.04 0.09 0 0.04 0.11 0 0.06 0.12 0 0.07  67 0.07 0 0.04 0.08 0 0.03 0.08 0 0.04 0.08 0 0.04 0.11 0 0.06 0.12 0 0.07  68 0.07 0 0.03 0.07 0 0.03 0.09 0 0.03 0.08 0 0.04 0.1 0 0.05 0.12 0 0.07  69 0.08 0 0.04 0.08 0 0.04 0.07 0 0.03 0.08 0 0.03 0.1 0 0.05 0.12 0 0.07  70 0.09 0 0.04 0.09 0 0.04 0.07 0 0.03 0.09 0 0.05 0.1 0 0.05 0.12 0 0.08  71 0.08 0 0.04 0.08 0 0.03 0.07 0 0.03 0.07 0 0.03 0.12 0 0.05 0.12 0 0.07  72 0.06 0 0.03 0.08 0 0.03 0.06 0 0.03 0.08 0 0.03 0.12 0 0.06 0.12 0 0.07  73 0.07 0 0.03 0.09 0 0.03 0.06 0 0.03 0.15 0 0.06 0.12 0 0.06 0.14 0 0.07  74 0.06 0 0.03 0.09 0 0.03 0.06 0 0.03 0.06 0 0.06 0.11 0 0.06 0.15 0 0.07  75 0.07 0 0.03 0.08 0 0.03 0.07 0 0.03 0.04 0 0.06 0.11 0 0.06 0.15 0 0.07  76 0.06 0 0.02 0.08 0 0.03 0.07 0 0.03 0.05 0 0.06 0.12 0.01 0.06 0.13 0 0.07  77 0.07 0 0.03 0.09 0 0.04 0.07 0 0.04 0.06 0 0.06 0.12 0 0.06 0.15 0 0.07  78 0.08 0 0.04 0.09 0 0.03 0.06 0 0.04 0.09 0 0.06 0.12 0 0.06 0.15 0 0.07  79 0.08 0 0.04 0.09 0 0.04 0.09 0 0.03 0.08 0 0.06 0.11 0 0.06 0.13 0 0.07  80 0.07 0 0.03 0.08 0 0.04 0.07 0 0.03 0.08 0 0.06 0.12 0 0.07 0.15 0.02 0.07  81 0.07 0 0.04 0.09 0 0.05 0.06 0 0.03 0.09 0 0.06 0.11 0 0.07 0.12 0 0.07  82 0.08 0 0.04 0.08 0 0.04 0.05 0 0.03 0.1 0 0.06 0.13 0 0.07 0.11 0 0.07  83 0.07 0 0.04 0.08 0 0.04 0.05 0 0.03 0.1 0 0.06 0.12 0 0.07 0.13 0 0.07  84 0.07 0 0.04 0.07 0 0.04 0.06 0 0.02 0.11 0 0.04 0.12 0 0.08 0.12 0.01 0.07  85 0.07 0 0.04 0.09 0 0.04 0.05 0 0.02 0.09 0 0.04 0.12 0 0.08 0.14 0 0.07  86 0.08 0.01 0.06 0.07 0 0.04 0.07 0 0.03 0.11 0 0.05 0.11 0 0.07 0.11 0 0.05  87 0.08 0 0.04 0.07 0 0.04 0.08 0 0.03 0.09 0 0.04 0.13 0 0.08 0.09 0 0.04  88 0.09 0 0.04 0.07 0 0.04 0.07 0 0.04 0.08 0 0.04 0.14 0 0.07 0.08 0 0.04  89 0.08 0 0.04 0.07 0 0.04 0.08 0 0.03 0.08 0 0.03 0.14 0 0.07 0.14 0 0.07  90 0.09 0 0.04 0.08 0 0.04 0.09 0 0.04 0.12 0 0.04 0.13 0 0.07 0.14 0 0.07  91 0.07 0 0.04 0.07 0 0.04 0.1 0 0.04 0.08 0 0.04 0.13 0 0.07 0.15 0 0.07  92 0.07 0 0.04 0.07 0 0.04 0.09 0 0.04 0.07 0 0.03 0.12 0 0.07 0.15 0.01 0.08  93 0.09 0 0.04 0.08 0 0.04 0.08 0 0.04 0.08 0 0.04 0.12 0 0.07 0.13 0 0.07  94 0.08 0 0.04 0.07 0 0.04 0.09 0 0.04 0.07 0 0.06 0.13 0.03 0.08 0.13 0 0.07  95 0.09 0 0.04 0.07 0 0.03 0.07 0 0.03 0.08 0 0.04 0.13 0 0.07 0.14 0 0.07  96 0.08 0 0.04 0.08 0 0.03 0.09 0.01 0.04 0.07 0 0.03 0.14 0 0.07 0.14 0 0.06  97 0.08 0 0.04 0.08 0 0.03 0.08 0 0.04 0.06 0 0.03 0.14 0 0.07 0.12 0.03 0.07  98 0.09 0 0.04 0.08 0 0.04 0.08 0 0.04 0.07 0 0.03 0.13 0 0.07 0.15 0 0.07  99 0.08 0 0.04 0.07 0 0.03 0.08 0 0.04 0.07 0 0.03 0.13 0 0.06 0.13 0 0.08  100 0.07 0 0.04 0.08 0 0.04 0.07 0 0.04 0.09 0 0.03 0.11 0.03 0.05 0.14 0 0.07  101 0.09 0 0.05 0.08 0 0.04 0.06 0 0.04 0.08 0 0.03 0.12 0.04 0.05 0.13 0 0.07  102 0.09 0 0.04 0.08 0 0.04 0.08 0 0.04 0.08 0 0.03 0.14 0 0.05 0.14 0 0.08  103 0.08 0 0.04 0.07 0 0.04 0.07 0 0.03 0.07 0 0.03 0.15 0 0.05 0.12 0 0.07  104 0.07 0 0.04 0.08 0 0.05 0.06 0 0.03 0.07 0 0.02 0.12 0 0.06 0.12 0 0.08  105 0.09 0 0.04 0.08 0 0.04 0.08 0 0.03 0.07 0 0.04 0.11 0 0.07 0.12 0.03 0.08  106 0.09 0 0.04 0.08 0 0.05 0.08 0 0.03 0.06 0 0.03 0.12 0 0.07 0.12 0 0.07  107 0.07 0 0.04 0.08 0 0.05 0.08 0 0.04 0.08 0 0.03 0.13 0 0.08 0.13 0.04 0.08  108 0.07 0 0.04 0.09 0 0.05 0.08 0 0.04 0.08 0 0.03 0.13 0 0.08 0.13 0.04 0.08  109 0.06 0 0.04 0.07 0 0.04 0.08 0 0.04 0.07 0 0.03 0.14 0 0.07 0.12 0.04 0.08  110 0.07 0 0.04 0.09 0 0.05 0.08 0 0.03 0.08 0 0.04 0.13 0 0.07 0.12 0 0.08  111 0.07 0 0.04 0.09 0.01 0.05 0.06 0 0.03 0.07 0 0.03 0.13 0 0.07 0.12 0.03 0.08  112 0.07 0 0.04 0.08 0 0.05 0.07 0 0.03 0.08 0 0.02 0.08 0 0.05 0.12 0 0.08  113 0.07 0 0.04 0.08 0 0.05 0.08 0 0.04 0.1 0 0.03 0.08 0 0.05 0.12 0.02 0.08  114 0.07 0 0.04 0.08 0.01 0.05 0.09 0 0.04 0.11 0 0.04 0.14 0.03 0.06 0.12 0.03 0.06  115 0.08 0 0.04 0.09 0 0.05 0.09 0 0.05 0.08 0 0.03 0.12 0 0.07 0.12 0.03 0.07  116 0.08 0 0.04 0.07 0 0.04 0.07 0 0.03 0.07 0 0.04 0.12 0 0.07 0.13 0.02 0.07  117 0.07 0 0.03 0.07 0 0.04 0.09 0 0.04 0.1 0 0.04 0.14 0.02 0.08 0.12 0 0.07  118 0.08 0 0.04 0.07 0.01 0.04 0.09 0 0.05 0.12 0 0.05 0.13 0 0.07 0.14 0 0.08  119 0.07 0 0.04 0.07 0 0.04 0.09 0 0.04 0.15 0 0.04 0.12 0 0.07 0.14 0 0.08  120 0.07 0.01 0.04 0.07 0 0.04 0.07 0 0.04 0.09 0 0.03 0.12 0 0.07 0.13 0 0.08  121 0.07 0 0.04 0.09 0.02 0.05 0.07 0 0.04 0.09 0 0.03 0.12 0 0.07 0.14 0 0.08  122 0.07 0 0.04 0.08 0 0.04 0.08 0 0.05 0.11 0 0.04 0.12 0 0.07 0.14 0 0.08  123 0.09 0 0.05 0.07 0.01 0.04 0.08 0 0.04 0.09 0 0.05 0.11 0 0.07 0.14 0 0.08  124 0.1 0 0.05 0.06 0 0.04 0.09 0 0.05 0.09 0 0.05 0.12 0 0.07 0.14 0.03 0.08  125 0.08 0 0.05 0.07 0 0.04 0.07 0 0.05 0.09 0 0.06 0.12 0 0.07 0.14 0 0.08  126 0.08 0 0.05 0.09 0 0.04 0.08 0.01 0.05 0.08 0 0.06 0.14 0 0.07 0.15 0 0.08  127 0.08 0 0.05 0.09 0 0.04 0.07 0 0.04 0.09 0 0.06 0.13 0 0.07 0.13 0.04 0.08  128 0.09 0 0.05 0.08 0 0.04 0.07 0 0.04 0.07 0 0.06 0.12 0 0.07 0.13 0.03 0.08  129 0.08 0 0.05 0.07 0 0.04 0.06 0 0.04 0.09 0 0.06 0.14 0 0.07 0.13 0 0.08  130 0.1 0 0.05 0.08 0 0.04 0.06 0 0.03 0.07 0 0.06 0.15 0.02 0.07 0.15 0 0.08  131 0.11 0 0.05 0.07 0 0.04 0.06 0.01 0.04 0.07 0 0.06 0.15 0 0.07 0.14 0 0.08  132 0.09 0 0.05 0.07 0 0.04 0.07 0 0.04 0.07 0 0.06 0.14 0 0.08 0.14 0 0.08  133 0.09 0 0.05 0.08 0 0.04 0.07 0 0.04 0.05 0 0.06 0.13 0 0.08 0.14 0 0.08  134 0.08 0 0.04 0.08 0 0.04 0.07 0 0.04 0.05 0 0.06 0.14 0 0.08 0.04 0 0.05  135 0.08 0 0.04 0.08 0 0.04 0.1 0 0.05 0.04 0 0.06 0.12 0 0.08 0.03 0 0.05  136 0.07 0 0.05 0.07 0 0.04 0.07 0 0.04 0.03 0 0.01 0.14 0.03 0.09 0.14 0 0.07  137 0.08 0 0.06 0.07 0 0.04 0.08 0 0.04 0.03 0 0.01 0.14 0 0.08 0.14 0 0.08  138 0.08 0 0.05 0.09 0 0.05 0.08 0 0.05 0.03 0 0.01 0.13 0 0.08 0.14 0 0.09  139 0.07 0 0.05 0.08 0 0.05 0.09 0 0.05 0.03 0 0.01 0.13 0 0.08 0.13 0 0.07  140 0.08 0 0.05 0.1 0 0.05 0.1 0 0.05 0.02 0 0.01 0.12 0 0.08 0.13 0 0.07  141 0.08 0 0.05 0.1 0 0.05 0.1 0 0.06 0.04 0 0.01 0.13 0 0.08 0.14 0 0.08  142 0.08 0 0.05 0.1 0.01 0.05 0.09 0 0.05 0.04 0 0.01 0.12 0 0.09 0.13 0 0.07  143 0.08 0.01 0.05 0.09 0 0.05 0.09 0.02 0.06 0.02 0 0.01 0.12 0 0.08 0.14 0 0.07  144 0.08 0 0.05 0.11 0 0.05 0.09 0.01 0.06 0.03 0 0.01 0.12 0 0.08 0.18 0 0.06  145 0.07 0.01 0.04 0.09 0 0.05 0.09 0 0.05 0.04 0 0.02 0.12 0 0.08 0.14 0.02 0.07  146 0.08 0 0.03 0.09 0 0.05 0.09 0 0.05 0.06 0 0.05 0.12 0 0.07 0.15 0 0.07  147 0.09 0 0.04 0.08 0 0.04 0.09 0 0.05 0.12 0 0.06 0.12 0 0.08 0.14 0 0.07  148 0.06 0 0.04 0.09 0 0.04 0.08 0.03 0.06 0.11 0 0.06 0.12 0 0.08 0.15 0 0.07  149 0.07 0 0.04 0.06 0 0.04 0.08 0 0.05 0.11 0 0.06 0.12 0 0.08 0.15 0 0.07  150 0.08 0 0.04 0.08 0 0.04 0.09 0 0.05 0.02 0 0.06 0.12 0 0.08 0.14 0 0.08  151 0.08 0 0.04 0.07 0 0.04 0.09 0 0.05 0.02 0 0.06 0.12 0 0.08 0.16 0 0.07  152 0.1 0 0.05 0.07 0 0.04 0.08 0 0.05 0.01 0 0.6 0.12 0 0.07 0.14 0 0.07  153 0.1 0.01 0.05 0.08 0 0.04 0.1 0 0.05 0.01 0.01 0.06 0.13 0 0.08 0.16 0 0.08  154 0.1 0 0.05 0.08 0 0.04 0.08 0 0.05 0.12 0 0.06 0.09 0 0.05 0.16 0.05 0.07  155 0.09 0 0.04 0.12 0 0.05 0.09 0.03 0.05 0.02 0 0.06 0.09 0 0.05 0.12 0 0.08  156 0.1 0 0.05 0.09 0 0.05 0.09 0 0.05 0.02 0 0.06 0.12 0 0.07 0.14 0 0.08  157 0.09 0 0.04 0.08 0 0.05 0.06 0 0.04 0.04 0 0.06 0.13 0 0.07 0.14 0 0.05  158 0.09 0.01 0.04 0.09 0 0.04 0.07 0 0.04 0.03 0 0.06 0.13 0 0.07 0.16 0 0.05  159 0.09 0 0.06 0.11 0 0.05 0.08 0 0.04 0.01 0 0.06 0.12 0 0.07 0.14 0 0.05  160 0.09 0.01 0.06 0.09 0 0.05 0.06 0 0.04 0.1 0 0.06 0.13 0 0.07 0.14 0.04 0.08  161 0.12 0 0.06 0.1 0 0.05 0.07 0 0.04 0.02 0 0.06 0.12 0 0.07 0.15 0 0.08  162 0.11 0 0.05 0.1 0 0.04 0.08 0 0.04 0.01 0 0.06 0.13 0 0.07 0.15 0 0.08  163 0.1 0.01 0.05 0.11 0 0.05 0.07 0 0.04 0.09 0 0.06 0.13 0 0.07 0.14 0.03 0.08  164 0.1 0 0.05 0.1 0 0.06 0.08 0 0.04 0.01 0 0.06 0.14 0 0.07 0.12 0 0.08  165 0.11 0 0.05 0.09 0 0.06 0.11 0 0.05 0.01 0 0.06 0.12 0 0.07 0.13 0.02 0.08  166 0.09 0 0.05 0.1 0 0.04 0.08 0 0.05 0.009 0 0.06 0.12 0 0.07 0.14 0 0.08  167 0.1 0 0.05 0.09 0 0.04 0.1 0 0.05 0.04 0 0.02 0.12 0 0.07 0.14 0.02 0.08  168 0.1 0 0.05 0.09 0 0.04 0.09 0 0.05 0.06 0 0.02 0.13 0 0.07 0.13 0 0.08  169 0.11 0.02 0.05 0.1 0 0.05 0.12 0 0.05 0.04 0 0.02 0.12 0 0.07 0.13 0 0.07  170 0.1 0.01 0.05 0.11 0 0.05 0.11 0 0.05 0.06 0 0.06 0.13 0 0.07 0.15 0 0.08  171 0.11 0 0.05 0.09 0 0.05 0.12 0 0.05 0.01 0 0.06 0.13 0 0.07 0.15 0.01 0.07  172 0.11 0 0.04 0.1 0 0.05 0.11 0 0.05 0.02 0 0.01 0.13 0 0.07 0.15 0 0.08  173 0.12 0 0.05 0.1 0 0.05 0.11 0 0.06 0.04 0.01 0.02 0.13 0 0.08 0.14 0.02 0.08  174 0.1 0 0.04 0.11 0 0.06 0.1 0 0.05 0.01 0 0.06 0.12 0 0.07 0.14 0.02 0.08  175 0.09 0 0.04 0.1 0 0.05 0.09 0.02 0.06 0.04 0 0.01 0.12 0 0.07 0.14 0 0.08  176 0.1 0 0.04 0.09 0.01 0.05 0.1 0.01 0.05 0.02 0 0.01 0.12 0 0.08 0.15 0 0.07  177 0.1 0 0.05 0.08 0.01 0.05 0.09 0.02 0.06 0.02 0 0.01 0.11 0 0.07 0.15 0 0.07  178 0.09 0 0.04 0.09 0 0.04 0.12 0.03 0.06 0.01 0 0.06 0.12 0.01 0.07 0.15 0 0.07  179 0.09 0 0.04 0.1 0 0.06 0.09 0.02 0.05 0.02 0 0.06 0.11 0 0.07 0.16 0 0.07  180 0.09 0 0.04 0.11 0 0.05 0.1 0 0.06 0.03 0 0.06 0.13 0 0.07 0.15 0 0.08  181 0.08 0 0.05 0.1 0 0.05 0.09 0.03 0.05 0.02 0 0.06 0.13 0 0.07 0.14 0 0.07  182 0.09 0 0.05 0.11 0 0.05 0.1 0 0.05 0.02 0 0.06 0.14 0 0.07 0.14 0 0.08  183 0.09 0 0.06 0.1 0 0.05 0.09 0.01 0.05 0.02 0 0.06 0.13 0 0.07 0.15 0 0.09  184 0.09 0 0.06 0.11 0 0.05 0.09 0 0.05 0.02 0 0.06 0.13 0 0.06 0.15 0 0.08  185 0.09 0 0.05 0.11 0 0.05 0.1 0 0.05 0.03 0 0.01 0.13 0 0.07 0.15 0 0.08  186 0.09 0 0.05 0.11 0 0.05 0.09 0.02 0.05 0.02 0 0.01 0.13 0 0.08 0.15 0 0.08  187 0.1 0 0.05 0.08 0 0.05 0.1 0 0.04 0.04 0 0.01 0.14 0 0.07 0.14 0 0.08  188 0.11 0.02 0.05 0.08 0 0.05 0.1 0 0.04 0.01 0 0.06 0.12 0.01 0.07 0.14 0.02 0.08  189 0.1 0 0.05 0.1 0 0.05 0.09 0 0.04 0.01 0 0.06 0.12 0 0.06 0.14 0 0.08  190 0.09 0 0.05 0.1 0.02 0.06 0.09 0 0.04 0.02 0 0.06 0.12 0 0.07 0.14 0.02 0.08  191 0.09 0 0.05 0.1 0 0.06 0.08 0 0.04 0.01 0 0.06 0.12 0 0.07 0.15 0 0.07  192 0.1 0 0.05 0.1 0 0.06 0.09 0.01 0.05 0.01 0 0.06 0.11 0 0.07 0.15 0 0.08  193 0.1 0 0.05 0.1 0 0.06 0.09 0 0.05 0.02 0 0.06 0.12 0 0.07 0.15 0 0.08  194 0.1 0 0.05 0.09 0 0.06 0.09 0.02 0.06 0.02 0 0.06 0.11 0 0.07 0.15 0 0.08  195 0.12 0 0.05 0.1 0 0.07 0.09 0.02 0.06 0.01 0 0.06 0.12 0 0.07 0.15 0.03 0.07  196 0.09 0 0.06 0.1 0 0.07 0.09 0 0.05 0.01 0 0.06 0.12 0 0.07 0.14 0.01 0.07  197 0.09 0 0.06 0.1 0 0.07 0.09 0 0.05 0.03 0 0.06 0.11 0 0.07 0.14 0 0.07  198 0.09 0 0.06 0.11 0 0.06 0.09 0 0.05 0.02 0 0.06 0.12 0 0.07 0.14 0 0.07  199 0.08 0 0.06 0.13 0 0.06 0.11 0 0.05 0.01 0 0.06 0.08 0 0.05 0.15 0.02 0.07  200 0.1 0 0.06 0.12 0 0.06 0.1 0 0.05 0.02 0 0.06 0.09 0 0.05 0.14 0.03 0.06 |
| --- |

| Control (mm) Infected (mm)  Day 3 R1 R2 R3 R1 R2 R3  Max Min Average Max Min Average Max Min Average Max Min Average Max Min Average Max Min Average  1 0.06 0 0.04 0.05 0 0.02 0.09 0 0.04 0.11 0 0.05 0.12 0 0.05 0.11 0.03 0.07  2 0.06 0 0.04 0.06 0 0.03 0.07 0 0.04 0.11 0 0.05 0.13 0 0.05 0.1 0.04 0.07  3 0.06 0 0.03 0.06 0 0.03 0.07 0 0.03 0.1 0 0.05 0.12 0 0.05 0.1 0 0.07  4 0.06 0 0.03 0.05 0 0.02 0.07 0 0.04 0.1 0.2 0.06 0.12 0 0.05 0.1 0 0.08  5 0.05 0 0.03 0.05 0 0.03 0.08 0 0.04 0.12 0.02 0.06 0.11 0 0.05 0.1 0 0.08  6 0.06 0 0.04 0.05 0 0.03 0.08 0 0.04 0.12 0 0.05 0.11 0 0.05 0.1 0.04 0.08  7 0.06 0 0.03 0.06 0 0.04 0.08 0 0.04 0.09 0 0.05 0.11 0 0.05 0.11 0.02 0.07  8 0.06 0 0.03 0.06 0 0.04 0.08 0 0.04 0.09 0.03 0.05 0.1 0 0.05 0.09 0.03 0.06  9 0.06 0 0.03 0.05 0 0.03 0.08 0 0.04 0.12 0 0.05 0.11 0.01 0.06 0.09 0 0.05  10 0.05 0 0.03 0.06 0 0.04 0.09 0 0.04 0.16 0.03 0.05 0.12 0 0.05 0.09 0.02 0.05  11 0.05 0 0.03 0.06 0 0.03 0.09 0 0.04 0.21 0 0.05 0.13 0 0.05 0.09 0 0.05  12 0.06 0 0.03 0.05 0.02 0.04 0.09 0 0.04 0.2 0.03 0.05 0.11 0 0.05 0.09 0.03 0.05  13 0.05 0 0.03 0.05 0 0.04 0.09 0 0.04 0.19 0 0.05 0.12 0 0.05 0.09 0 0.06  14 0.05 0 0.03 0.05 0 0.04 0.09 0 0.04 0.16 0 0.05 0.12 0 0.05 0.09 0 0.07  15 0.04 0 0.02 0.04 0 0.03 0.08 0 0.04 0.18 0 0.06 0.11 0 0.05 0.09 0.04 0.07  16 0.04 0 0.02 0.05 0 0.03 0.07 0 0.03 0.2 0 0.06 0.12 0 0.05 0.1 0 0.07  17 0.04 0 0.03 0.05 0 0.03 0.09 0 0.03 0.11 0 0.05 0.12 0 0.05 0.1 0.05 0.08  18 0.04 0 0.02 0.05 0 0.03 0.06 0 0.03 0.12 0 0.05 0.11 0 0.05 0.12 0.05 0.08  19 0.05 0 0.02 0.05 0 0.03 0.07 0 0.03 0.11 0 0.05 0.11 0 0.05 0.1 0 0.08  20 0.04 0 0.01 0.05 0 0.02 0.09 0 0.03 0.11 0 0.05 0.11 0 0.05 0.1 0.05 0.08  21 0.08 0 0.03 0.05 0 0.02 0.09 0 0.03 0.11 0 0.05 0.12 0 0.05 0.1 0 0.07  22 0.08 0 0.03 0.05 0 0.02 0.08 0 0.03 0.12 0 0.05 0.21 0 0.07 0.09 0.02 0.07  23 0.08 0 0.03 0.04 0 0.01 0.09 0 0.03 0.11 0 0.05 0.12 0 0.08 0.11 0 0.07  24 0.08 0 0.04 0.07 0 0.02 0.07 0 0.03 0.12 0 0.08 0.11 0 0.07 0.1 0.05 0.08  25 0.08 0 0.04 0.05 0 0.02 0.08 0 0.03 0.12 0 0.07 0.11 0 0.07 0.12 0 0.08  26 0.08 0 0.04 0.06 0 0.03 0.08 0 0.03 0.12 0.01 0.08 0.12 0 0.07 0.12 0 0.09  27 0.09 0 0.04 0.08 0 0.04 0.1 0 0.04 0.11 0 0.08 0.13 0.02 0.08 0.11 0.05 0.09  28 0.08 0 0.04 0.08 0 0.05 0.1 0 0.04 0.15 0 0.08 0.13 0 0.07 0.12 0 0.09  29 0.08 0 0.05 0.07 0 0.04 0.1 0 0.03 0.11 0 0.08 0.11 0.02 0.07 0.12 0 0.09  30 0.08 0 0.05 0.07 0 0.04 0.08 0 0.04 0.1 0 0.08 0.1 0.03 0.07 0.12 0 0.08  31 0.07 0 0.04 0.08 0 0.05 0.08 0 0.04 0.11 0.06 0.08 0.11 0 0.07 0.12 0 0.05  32 0.07 0 0.04 0.08 0 0.05 0.09 0 0.04 0.11 0 0.08 0.11 0 0.07 0.11 0.04 0.07  33 0.07 0 0.03 0.06 0 0.04 0.09 0 0.04 0.12 0 0.08 0.11 0 0.06 0.11 0.01 0.06  34 0.07 0 0.03 0.06 0 0.04 0.08 0 0.04 0.12 0 0.07 0.11 0 0.07 0.11 0 0.05  35 0.06 0.01 0.04 0.07 0 0.04 0.09 0 0.04 0.12 0.03 0.07 0.14 0 0.08 0.1 0 0.05  36 0.06 0 0.04 0.06 0.02 0.04 0.08 0 0.05 0.12 0 0.05 0.12 0 0.06 0.1 0 0.04  37 0.06 0.01 0.03 0.07 0 0.04 0.08 0 0.04 0.12 0 0.05 0.11 0 0.07 0.06 0 0.03  38 0.05 0 0.03 0.07 0.01 0.03 0.09 0 0.04 0.12 0 0.05 0.11 0 0.06 0.08 0 0.03  39 0.05 0 0.03 0.07 0 0.03 0.07 0 0.04 0.11 0 0.07 0.12 0 0.07 0.08 0 0.03  40 0.06 0 0.03 0.06 0 0.03 0.08 0 0.05 0.1 0 0.05 0.11 0 0.06 0.06 0 0.03  41 0.06 0 0.03 0.05 0 0.03 0.08 0 0.04 0.09 0 0.05 0.12 0 0.06 0.07 0 0.04  42 0.07 0 0.03 0.06 0 0.04 0.08 0 0.04 0.09 0 0.05 0.11 0 0.07 0.08 0 0.04  43 0.07 0 0.03 0.06 0 0.04 0.07 0 0.04 0.1 0 0.05 0.11 0 0.06 0.08 0 0.05  44 0.05 0 0.03 0.06 0 0.03 0.08 0 0.04 0.09 0 0.04 0.11 0 0.06 0.08 0 0.05  45 0.06 0 0.03 0.05 0 0.03 0.08 0 0.04 0.09 0 0.04 0.11 0 0.06 0.09 0 0.05  46 0.06 0 0.03 0.05 0 0.03 0.07 0 0.04 0.09 0 0.04 0.11 0 0.06 0.11 0.03 0.07  47 0.06 0 0.03 0.06 0 0.03 0.08 0 0.03 0.09 0 0.04 0.11 0 0.06 0.12 0 0.07  48 0.08 0 0.04 0.06 0 0.03 0.06 0 0.04 0.07 0 0.03 0.11 0 0.07 0.12 0 0.08  49 0.08 0 0.05 0.04 0 0.02 0.08 0 0.04 0.07 0 0.02 0.11 0 0.07 0.12 0 0.08  50 0.09 0 0.04 0.04 0 0.02 0.09 0 0.04 0.09 0 0.04 0.12 0 0.07 0.12 0 0.09  51 0.08 0 0.03 0.04 0 0.02 0.08 0 0.04 0.1 0 0.04 0.12 0 0.07 0.12 0 0.09  52 0.08 0 0.03 0.05 0 0.02 0.08 0 0.04 0.1 0 0.04 0.1 0 0.07 0.13 0 0.1  53 0.08 0 0.04 0.07 0 0.04 0.08 0 0.04 0.11 0 0.05 0.13 0 0.06 0.12 0 0.09  54 0.07 0 0.04 0.08 0 0.04 0.08 0 0.04 0.1 0 0.05 0.11 0 0.07 0.13 0 0.09  55 0.1 0 0.05 0.08 0 0.05 0.1 0 0.04 0.12 0 0.05 0.11 0 0.07 0.13 0 0.09  56 0.08 0 0.04 0.08 0 0.05 0.1 0 0.05 0.11 0 0.07 0.11 0 0.06 0.13 0 0.08  57 0.09 0 0.04 0.06 0 0.04 0.09 0 0.04 0.1 0 0.07 0.1 0 0.07 0.13 0 0.08  58 0.08 0.01 0.04 0.08 0 0.04 0.09 0 0.05 0.11 0 0.08 0.11 0 0.07 0.12 0 0.08  59 0.08 0 0.04 0.07 0 0.04 0.08 0 0.04 0.11 0.01 0.07 0.12 0 0.07 0.12 0 0.09  60 0.08 0 0.04 0.07 0 0.04 0.09 0 0.04 0.11 0 0.07 0.13 0 0.07 0.12 0 0.09  61 0.07 0 0.04 0.07 0 0.04 0.09 0 0.04 0.11 0 0.07 0.11 0 0.07 0.12 0 0.09  62 0.06 0 0.03 0.06 0 0.04 0.08 0 0.04 0.12 0 0.07 0.12 0 0.07 0.12 0.02 0.09  63 0.08 0 0.03 0.07 0.01 0.04 0.06 0 0.04 0.11 0 0.07 0.11 0 0.07 0.14 0 0.1  64 0.06 0 0.03 0.07 0 0.03 0.08 0 0.05 0.11 0.04 0.07 0.13 0 0.07 0.13 0 0.1  65 0.06 0 0.03 0.07 0 0.03 0.1 0 0.05 0.12 0 0.08 0.12 0 0.05 0.13 0 0.1  66 0.08 0 0.03 0.05 0 0.03 0.1 0 0.05 0.11 0 0.07 0.1 0 0.05 0.14 0 0.1  67 0.05 0 0.03 0.06 0 0.03 0.08 0 0.05 0.11 0.05 0.08 0.12 0 0.05 0.14 0 0.09  68 0.07 0 0.03 0.05 0 0.02 0.08 0 0.05 0.11 0 0.08 0.1 0 0.05 0.13 0.06 0.09  69 0.07 0 0.01 0.06 0 0.01 0.11 0 0.05 0.1 0 0.07 0.1 0 0.05 0.12 0 0.08  70 0.04 0 0.02 0.05 0 0.02 0.1 0 0.05 0.1 0.04 0.07 0.09 0 0.05 0.12 0 0.07  71 0.05 0 0.02 0.05 0 0.02 0.1 0 0.05 0.1 0 0.06 0.12 0 0.07 0.13 0.06 0.07  72 0.05 0 0.02 0.05 0 0.02 0.09 0.01 0.05 0.09 0 0.06 0.1 0 0.07 0.12 0 0.07  73 0.05 0 0.02 0.05 0 0.02 0.11 0 0.04 0.1 0 0.06 0.12 0 0.07 0.1 0 0.05  74 0.03 0 0.01 0.05 0 0.02 0.1 0 0.05 0.1 0 0.06 0.11 0 0.07 0.08 0 0.06  75 0.04 0 0.01 0.04 0 0.02 0.08 0.02 0.05 0.11 0 0.06 0.11 0 0.07 0.09 0.04 0.07  76 0.05 0 0.01 0.05 0 0.03 0.08 0 0.05 0.1 0.04 0.06 0.12 0 0.07 0.09 0 0.06  77 0.09 0 0.03 0.04 0 0.02 0.09 0.03 0.05 0.1 0.02 0.06 0.12 0 0.08 0.1 0 0.07  78 0.12 0 0.06 0.04 0 0.02 0.07 0.02 0.05 0.1 0.03 0.06 0.15 0 0.07 0.11 0.03 0.08  79 0.12 0 0.08 0.04 0 0.01 0.13 0 0.05 0.09 0.03 0.06 0.14 0 0.07 0.11 0 0.07  80 0.1 0 0.07 0.03 0 0.01 0.11 0.01 0.05 0.09 0.01 0.06 0.12 0 0.07 0.11 0 0.09  81 0.09 0 0.05 0.06 0 0.02 0.12 0 0.05 0.11 0 0.05 0.12 0 0.07 0.11 0.04 0.09  82 0.1 0 0.04 0.09 0 0.04 0.1 0 0.06 0.12 0.03 0.05 0.13 0 0.07 0.11 0.06 0.09  83 0.11 0.01 0.05 0.11 0 0.07 0.1 0 0.06 0.11 0.03 0.07 0.13 0 0.07 0.11 0 0.08  84 0.09 0 0.05 0.1 0 0.07 0.11 0 0.05 0.12 0.03 0.07 0.11 0 0.07 0.11 0 0.09  85 0.08 0 0.04 0.1 0 0.07 0.12 0 0.05 0.11 0.03 0.07 0.12 0 0.07 0.33 0.05 0.11  86 0.1 0 0.04 0.08 0 0.05 0.11 0 0.05 0.12 0 0.07 0.13 0 0.07 0.12 0.04 0.09  87 0.09 0 0.04 0.1 0 0.05 0.09 0 0.05 0.12 0.03 0.07 0.13 0 0.07 0.11 0 0.09  88 0.09 0 0.04 0.09 0 0.05 0.11 0 0.05 0.11 0.03 0.07 0.12 0 0.07 0.12 0.04 0.09  89 0.08 0 0.04 0.1 0 0.05 0.11 0 0.05 0.12 0.01 0.07 0.13 0 0.07 0.12 0 0.1  90 0.08 0.02 0.05 0.09 0 0.05 0.1 0 0.05 0.15 0.02 0.08 0.12 0 0.07 0.12 0 0.1  91 0.09 0 0.04 0.1 0 0.04 0.11 0 0.06 0.13 0 0.08 0.13 0 0.07 0.12 0.07 0.1  92 0.08 0 0.04 0.1 0 0.04 0.12 0 0.05 0.12 0 0.08 0.13 0 0.07 0.12 0 0.1  93 0.08 0 0.03 0.08 0 0.04 0.1 0 0.05 0.11 0 0.07 0.13 0 0.08 0.13 0 0.09  94 0.07 0 0.03 0.09 0.01 0.04 0.1 0 0.06 0.11 0 0.08 0.15 0 0.07 0.15 0 0.09  95 0.07 0 0.03 0.07 0 0.04 0.12 0 0.06 0.1 0 0.08 0.12 0 0.07 0.12 0 0.08  96 0.09 0 0.03 0.09 0 0.04 0.1 0.02 0.06 0.12 0 0.08 0.18 0 0.07 0.14 0 0.05  97 0.07 0 0.03 0.08 0.01 0.04 0.1 0.04 0.06 0.13 0 0.08 0.11 0 0.07 0.11 0 0.05  98 0.05 0 0.03 0.06 0 0.03 0.1 0.04 0.06 0.12 0 0.07 0.12 0 0.07 0.12 0 0.05  99 0.08 0 0.04 0.06 0 0.03 0.1 0 0.05 0.11 0 0.06 0.12 0 0.06 0.12 0 0.04  100 0.09 0 0.03 0.07 0 0.03 0.12 0 0.05 0.11 0 0.05 0.11 0 0.06 0.11 0 0.03  101 0.1 0 0.03 0.05 0 0.02 0.12 0 0.05 0.1 0 0.06 0.11 0 0.07 0.09 0 0.05  102 0.07 0 0.02 0.06 0 0.02 0.12 0.02 0.05 0.09 0 0.05 0.14 0 0.07 0.09 0 0.03  103 0.1 0 0.04 0.05 0 0.02 0.11 0 0.05 0.12 0 0.05 0.13 0 0.07 0.26 0 0.06  104 0.12 0 0.05 0.05 0 0.03 0.11 0 0.04 0.11 0 0.06 0.12 0 0.06 0.07 0 0.04  105 0.1 0 0.05 0.05 0 0.03 0.11 0 0.04 0.28 0 0.05 0.12 0 0.06 0.11 0 0.05  106 0.08 0 0.04 0.07 0 0.03 0.11 0 0.04 0.3 0 0.05 0.12 0 0.06 0.09 0 0.05  107 0.08 0 0.03 0.08 0 0.03 0.11 0 0.04 0.09 0 0.04 0.11 0 0.06 0.64 0 0.07  108 0.08 0 0.04 0.1 0 0.05 0.1 0 0.04 0.08 0 0.04 0.12 0 0.07 0.1 0 0.05  109 0.08 0 0.04 0.11 0 0.06 0.09 0.01 0.04 0.09 0 0.05 0.12 0 0.07 0.1 0 0.07  110 0.08 0 0.04 0.11 0 0.06 0.09 0 0.04 0.11 0 0.05 0.12 0 0.06 0.12 0 0.07  111 0.07 0 0.04 0.1 0 0.05 0.09 0 0.04 0.11 0 0.06 0.14 0 0.08 0.11 0 0.08  112 0.08 0 0.04 0.09 0 0.04 0.09 0.02 0.05 0.12 0 0.06 0.11 0 0.07 0.12 0 0.08  113 0.07 0 0.04 0.07 0 0.04 0.09 0.02 0.05 0.11 0 0.07 0.11 0 0.07 0.13 0 0.09  114 0.06 0 0.04 0.07 0 0.04 0.08 0 0.04 0.11 0.01 0.07 0.11 0 0.07 0.12 0 0.09  115 0.07 0 0.05 0.07 0 0.03 0.14 0 0.05 0.13 0 0.08 0.11 0 0.08 0.12 0 0.09  116 0.07 0 0.04 0.07 0 0.04 0.13 0 0.05 0.12 0.01 0.08 0.11 0 0.07 0.12 0 0.09  117 0.08 0 0.04 0.06 0.01 0.04 0.1 0 0.04 0.12 0 0.08 0.13 0 0.08 0.11 0 0.09  118 0.08 0 0.04 0.07 0 0.04 0.11 0 0.04 0.12 0 0.08 0.11 0 0.07 0.12 0.04 0.09  119 0.08 0 0.03 0.07 0 0.03 0.11 0 0.05 0.12 0 0.08 0.11 0 0.07 0.12 0 0.09  120 0.06 0 0.04 0.08 0 0.03 0.11 0 0.06 0.11 0 0.08 0.14 0 0.08 0.12 0 0.09  121 0.07 0 0.03 0.06 0 0.02 0.11 0 0.05 0.11 0 0.08 0.15 0 0.08 0.13 0.04 0.09  122 0.06 0 0.03 0.06 0 0.03 0.12 0.02 0.05 0.11 0.04 0.08 0.13 0 0.08 0.13 0 0.1  123 0.06 0 0.02 0.06 0 0.03 0.14 0 0.05 0.11 0 0.08 0.13 0 0.08 0.15 0.08 0.11  124 0.08 0 0.03 0.06 0 0.03 0.16 0 0.05 0.11 0 0.08 0.12 0 0.08 0.15 0 0.09  125 0.07 0 0.02 0.05 0 0.02 0.14 0 0.05 0.12 0 0.08 0.13 0 0.08 0.13 0.03 0.09  126 0.06 0 0.03 0.06 0 0.03 0.14 0 0.06 0.12 0 0.08 0.13 0 0.08 0.12 0 0.08  127 0.06 0 0.03 0.08 0 0.03 0.13 0 0.06 0.12 0 0.08 0.29 0 0.09 0.14 0 0.09  128 0.09 0 0.05 0.06 0 0.03 0.14 0 0.06 0.13 0 0.08 0.14 0 0.08 0.15 0 0.08  129 0.1 0 0.06 0.05 0 0.02 0.12 0 0.05 0.1 0 0.07 0.13 0 0.07 0.14 0 0.08  130 0.12 0 0.05 0.05 0 0.02 0.13 0.01 0.05 0.1 0 0.07 0.11 0 0.07 0.13 0.03 0.07  131 0.11 0 0.05 0.04 0 0.02 0.12 0 0.05 0.1 0 0.06 0.13 0 0.07 0.13 0 0.06  132 0.09 0 0.05 0.06 0 0.03 0.11 0 0.05 0.1 0.02 0.06 0.12 0 0.07 0.14 0 0.06  133 0.07 0 0.04 0.1 0 0.05 0.12 0 0.05 0.1 0 0.06 0.12 0 0.07 0.76 0 0.07  134 0.08 0 0.05 0.1 0 0.05 0.11 0.01 0.05 0.1 0 0.05 0.11 0 0.07 0.74 0 0.07  135 0.1 0 0.04 0.11 0 0.05 0.11 0 0.05 0.13 0 0.05 0.12 0 0.07 0.12 0 0.05  136 0.08 0 0.05 0.11 0 0.05 0.19 0 0.05 0.1 0 0.06 0.12 0 0.07 0.11 0 0.06  137 0.08 0 0.05 0.09 0 0.05 0.11 0 0.06 0.1 0.02 0.07 0.14 0.03 0.08 0.13 0.03 0.06  138 0.09 0 0.05 0.08 0 0.04 0.1 0.02 0.06 0.14 0.03 0.07 0.12 0 0.07 0.67 0 0.14  139 0.1 0 0.05 0.08 0 0.05 0.1 0 0.05 0.12 0 0.08 0.13 0 0.08 0.3 0.05 0.09  140 0.07 0 0.05 0.09 0 0.05 0.11 0 0.05 0.11 0 0.08 0.12 0 0.08 0.11 0 0.09  141 0.06 0.02 0.04 0.08 0 0.05 0.11 0 0.05 0.11 0.03 0.08 0.12 0 0.08 0.12 0 0.09  142 0.06 0 0.04 0.07 0 0.04 0.1 0.01 0.05 0.12 0.04 0.08 0.12 0 0.08 0.59 0.05 0.13  143 0.07 0 0.04 0.07 0 0.05 0.12 0.02 0.06 0.12 0.04 0.08 0.14 0 0.08 0.12 0 0.09  144 0.07 0 0.04 0.07 0 0.04 0.09 0 0.05 0.11 0.04 0.09 0.14 0 0.08 0.13 0 0.09  145 0.07 0 0.04 0.07 0 0.04 0.09 0 0.05 0.13 0.04 0.09 0.16 0 0.08 0.12 0 0.09  146 0.06 0 0.04 0.07 0 0.04 0.11 0 0.06 0.12 0.05 0.09 0.13 0.02 0.08 0.12 0 0.09  147 0.08 0 0.04 0.07 0 0.04 0.1 0 0.05 0.13 0.05 0.09 0.12 0 0.08 0.12 0 0.09  148 0.09 0.02 0.04 0.07 0 0.05 0.14 0 0.06 0.12 0.03 0.09 0.15 0 0.08 0.16 0.05 0.1  149 0.08 0 0.04 0.07 0.02 0.05 0.1 0 0.05 0.12 0.04 0.09 0.15 0 0.08 0.14 0 0.1  150 0.06 0 0.03 0.09 0 0.04 0.11 0 0.05 0.12 0.07 0.09 0.12 0 0.07 0.13 0.07 0.1  151 0.09 0 0.04 0.07 0.02 0.04 0.1 0 0.05 0.12 0.07 0.09 0.13 0 0.08 0.15 0 0.1  152 0.1 0 0.05 0.07 0 0.03 0.11 0 0.05 0.12 0 0.08 0.13 0 0.07 0.13 0.08 0.11  153 0.13 0 0.05 0.07 0 0.03 0.1 0 0.05 0.11 0 0.08 0.14 0 0.07 0.16 0 0.1  154 0.12 0 0.06 0.07 0 0.03 0.1 0 0.06 0.12 0.03 0.07 0.14 0 0.07 0.15 0.06 0.1  155 0.12 0 0.06 0.08 0 0.04 0.1 0 0.05 0.11 0.04 0.07 0.13 0 0.06 0.16 0.05 0.09  156 0.12 0 0.06 0.09 0 0.05 0.1 0 0.05 0.13 0 0.07 0.14 0 0.06 0.14 0 0.09  157 0.1 0.03 0.05 0.09 0 0.06 0.12 0.01 0.05 0.11 0 0.06 0.14 0 0.07 0.14 0 0.07  158 0.09 0 0.06 0.12 0 0.06 0.11 0 0.05 0.13 0.01 0.06 0.14 0 0.07 0.16 0 0.07  159 0.1 0 0.05 0.12 0 0.06 0.1 0 0.05 0.11 0 0.06 0.13 0 0.07 0.18 0.04 0.08  160 0.1 0.02 0.05 0.12 0 0.05 0.11 0 0.05 0.1 0 0.05 0.12 0 0.05 0.14 0 0.07  161 0.1 0 0.05 0.11 0 0.04 0.11 0 0.05 0.1 0 0.05 0.14 0 0.07 0.14 0 0.07  162 0.08 0 0.05 0.12 0 0.05 0.1 0 0.05 0.09 0 0.04 0.14 0 0.07 0.13 0 0.07  163 0.06 0 0.04 0.12 0.01 0.05 0.1 0 0.05 0.09 0 0.03 0.13 0 0.07 0.12 0 0.06  164 0.08 0.03 0.05 0.11 0 0.04 0.08 0.02 0.05 0.1 0 0.03 0.14 0 0.07 0.12 0.03 0.06  165 0.07 0 0.04 0.1 0 0.04 0.07 0 0.05 0.07 0 0.03 0.12 0 0.05 0.12 0 0.06  166 0.07 0 0.04 0.1 0 0.05 0.08 0 0.05 0.05 0 0.03 0.13 0 0.05 0.11 0 0.06  167 0.08 0 0.04 0.08 0 0.05 0.1 0 0.05 0.06 0 0.03 0.12 0 0.05 0.11 0 0.06  168 0.08 0 0.04 0.09 0 0.04 0.1 0 0.05 0.09 0 0.03 0.13 0 0.05 0.1 0.05 0.07  169 0.06 0 0.03 0.07 0 0.04 0.1 0.01 0.05 0.1 0 0.04 0.13 0 0.05 0.1 0.04 0.08  170 0.09 0 0.04 0.06 0 0.04 0.09 0 0.06 0.1 0 0.06 0.13 0 0.05 0.11 0 0.08  171 0.05 0 0.03 0.06 0 0.04 0.12 0 0.06 0.12 0 0.07 0.13 0 0.05 0.14 0 0.09  172 0.07 0 0.03 0.06 0 0.05 0.09 0.02 0.05 0.11 0 0.06 0.12 0 0.05 0.13 0 0.09  173 0.07 0 0.03 0.07 0 0.05 0.09 0 0.05 0.11 0 0.07 0.12 0 0.05 0.13 0 0.09  174 0.06 0 0.03 0.06 0 0.04 0.09 0 0.06 0.11 0 0.07 0.12 0 0.05 0.12 0 0.1  175 0.05 0 0.02 0.06 0 0.03 0.09 0 0.05 0.11 0 0.07 0.13 0 0.05 0.13 0.05 0.1  176 0.06 0 0.03 0.05 0 0.03 0.09 0.03 0.06 0.12 0 0.08 0.15 0 0.05 0.12 0 0.1  177 0.07 0.01 0.04 0.05 0 0.03 0.09 0 0.05 0.11 0 0.08 0.09 0 0.05 0.12 0 0.1  178 0.08 0 0.04 0.05 0 0.03 0.09 0 0.06 0.12 0.01 0.08 0.08 0 0.05 0.13 0 0.1  179 0.08 0 0.05 0.06 0 0.02 0.09 0 0.06 0.11 0 0.08 0.09 0 0.05 0.14 0 0.1  180 0.09 0 0.04 0.05 0 0.02 0.09 0 0.06 0.13 0.01 0.08 0.08 0.03 0.05 0.14 0.04 0.1  181 0.06 0 0.05 0.06 0 0.02 0.09 0 0.05 0.12 0 0.09 0.08 0.02 0.05 0.13 0 0.1  182 0.11 0 0.06 0.06 0 0.03 0.08 0 0.05 0.12 0 0.09 0.15 0 0.05 0.13 0 0.09  183 0.13 0 0.07 0.08 0 0.04 0.09 0 0.05 0.12 0 0.09 0.13 0 0.05 0.13 0 0.1  184 0.13 0 0.06 0.08 0 0.04 0.08 0.03 0.05 0.12 0.01 0.09 0.16 0 0.05 0.14 0.05 0.1  185 0.11 0 0.06 0.08 0 0.04 0.08 0.02 0.05 0.12 0 0.08 0.15 0 0.05 0.13 0 0.09  186 0.11 0 0.06 0.11 0 0.05 0.09 0 0.04 0.12 0 0.08 0.15 0 0.05 0.13 0 0.08  187 0.11 0 0.05 0.11 0 0.06 0.09 0 0.04 0.13 0 0.08 0.14 0 0.08 0.13 0 0.07  188 0.1 0 0.05 0.13 0 0.07 0.09 0 0.05 0.12 0 0.08 0.17 0 0.07 0.15 0.03 0.08  189 0.1 0 0.05 0.14 0 0.08 0.11 0 0.05 0.12 0 0.07 0.18 0 0.07 0.14 0.04 0.07  190 0.09 0 0.04 0.13 0 0.06 0.09 0.02 0.05 0.11 0 0.06 0.18 0 0.07 0.13 0 0.07  191 0.12 0 0.05 0.11 0 0.06 0.08 0 0.05 0.15 0 0.06 0.16 0 0.07 0.14 0 0.07  192 0.12 0 0.05 0.09 0 0.06 0.09 0 0.05 0.11 0 0.06 0.15 0 0.07 0.14 0.03 0.07  193 0.11 0 0.05 0.11 0 0.05 0.09 0 0.05 0.13 0.03 0.06 0.15 0 0.07 0.13 0.02 0.07  194 0.09 0 0.05 0.1 0 0.06 0.08 0 0.05 0.12 0 0.06 0.16 0 0.07 0.13 0 0.08  195 0.08 0.03 0.05 0.1 0 0.06 0.11 0.02 0.06 0.1 0 0.06 0.14 0 0.07 0.12 0.03 0.09  196 0.07 0.02 0.05 0.09 0 0.05 0.1 0 0.05 0.13 0 0.07 0.14 0 0.07 0.92 0 0.13  197 0.08 0 0.04 0.09 0 0.05 0.09 0.02 0.05 0.11 0 0.05 0.18 0 0.07 0.15 0 0.09  198 0.07 0 0.04 0.09 0 0.05 0.11 0.02 0.06 0.12 0 0.05 0.12 0 0.05 0.12 0 0.09  199 0.09 0 0.05 0.11 0 0.05 0.11 0 0.06 0.12 0 0.06 0.15 0 0.05 0.13 0.07 0.1  200 0.08 0.01 0.04 0.09 0 0.04 0.1 0 0.05 0.11 0 0.07 0.13 0 0.05 0.13 0 0.1 |
| --- |

| Control (mm) Infected (mm)  Day 4 R1 R2 R3 R1 R2 R3  Max Min Average Max Min Average Max Min Average Max Min Average Max Min Average Max Min Average  1 0.09 0 0.05 0.1 0 0.05 0.07 0 0.03 0.1 0.04 0.06 0.09 0.01 0.05 0.14 0 0.08  2 0.1 0 0.05 0.11 0 0.05 0.09 0 0.03 0.09 0 0.05 0.1 0.01 0.06 0.14 0 0.08  3 0.1 0 0.05 0.1 0 0.05 0.09 0 0.04 0.1 0 0.05 0.1 0 0.07 0.14 0 0.08  4 0.1 0 0.05 0.1 0 0.05 0.09 0 0.03 0.1 0 0.05 0.1 0 0.07 0.14 0 0.08  5 0.1 0 0.05 0.11 0 0.05 0.09 0 0.04 0.1 0 0.06 0.1 0.03 0.07 0.13 0 0.08  6 0.09 0 0.05 0.11 0 0.05 0.1 0 0.04 0.1 0.01 0.07 0.1 0 0.07 0.13 0 0.09  7 0.09 0 0.04 0.11 0 0.05 0.08 0 0.04 0.11 0 0.07 0.1 0 0.06 0.13 0 0.09  8 0.1 0 0.05 0.11 0 0.05 0.08 0 0.02 0.11 0.01 0.08 0.09 0.03 0.06 0.15 0 0.09  9 0.1 0 0.05 0.1 0 0.05 0.04 0 0.02 0.11 0 0.08 0.09 0 0.05 0.18 0.02 0.1  10 0.09 0 0.04 0.11 0 0.05 0.05 0 0.02 0.11 0 0.08 0.09 0 0.04 0.19 0.05 0.1  11 0.08 0 0.05 0.11 0 0.05 0.05 0 0.01 0.12 0.05 0.08 0.08 0 0.04 0.17 0.06 0.1  12 0.09 0 0.05 0.09 0 0.04 0.02 0 0.01 0.12 0.05 0.08 0.1 0 0.04 0.14 0.04 0.1  13 0.08 0 0.05 0.09 0 0.05 0.02 0 0.01 0.11 0.04 0.08 0.1 0 0.06 0.13 0 0.09  14 0.09 0.01 0.05 0.11 0 0.05 0.04 0 0.02 0.11 0 0.08 0.1 0 0.06 0.14 0 0.1  15 0.08 0 0.05 0.1 0 0.05 0.05 0 0.04 0.11 0.04 0.08 0.1 0 0.07 0.13 0.05 0.1  16 0.09 0.01 0.05 0.08 0 0.05 0.04 0 0.01 0.1 0.05 0.08 0.1 0 0.06 0.13 0.06 0.1  17 0.09 0 0.04 0.12 0 0.05 0.11 0 0.05 0.12 0.05 0.08 0.1 0 0.06 0.12 0 0.09  18 0.09 0 0.05 0.11 0 0.04 0.11 0 0.05 0.11 0.04 0.08 0.11 0 0.07 0.13 0 0.09  19 0.09 0.01 0.05 0.12 0 0.05 0.11 0 0.05 0.12 0.04 0.09 0.11 0 0.08 0.13 0 0.09  20 0.09 0 0.05 0.06 0 0.03 0.12 0 0.05 0.12 0.05 0.1 0.11 0 0.07 0.13 0 0.08  21 0.09 0 0.05 0.06 0 0.02 0.12 0 0.05 0.13 0.06 0.1 0.12 0 0.08 0.12 0 0.07  22 0.09 0.02 0.05 0.08 0 0.04 0.11 0 0.05 0.12 0 0.09 0.1 0 0.07 0.12 0.05 0.08  23 0.1 0 0.05 0.07 0 0.04 0.11 0 0.05 0.12 0.04 0.08 0.11 0.06 0.08 0.14 0.05 0.08  24 0.09 0.02 0.05 0.08 0 0.04 0.1 0 0.05 0.11 0.03 0.07 0.12 0 0.09 0.15 0.04 0.08  25 0.1 0 0.05 0.05 0 0.03 0.11 0 0.05 0.1 0 0.06 0.14 0 0.09 0.2 0 0.08  26 0.1 0 0.05 0.08 0 0.04 0.09 0 0.05 0.19 0.01 0.05 0.12 0.06 0.09 0.18 0.04 0.09  27 0.1 0 0.05 0.1 0 0.05 0.1 0 0.05 0.08 0 0.05 0.12 0 0.09 0.21 0.04 0.09  28 0.09 0 0.05 0.1 0 0.06 0.09 0 0.05 0.17 0 0.04 0.12 0 0.09 0.18 0.04 0.09  29 0.12 0 0.05 0.11 0 0.06 0.11 0 0.05 0.07 0 0.03 0.11 0.05 0.09 0.16 0 0.08  30 0.11 0 0.05 0.1 0 0.06 0.1 0 0.05 0.06 0 0.03 0.12 0 0.08 0.19 0.05 0.08  31 0.11 0 0.05 0.1 0 0.06 0.1 0 0.05 0.07 0 0.03 0.11 0 0.07 0.14 0 0.08  32 0.1 0 0.05 0.11 0 0.06 0.1 0 0.05 0.06 0 0.03 0.1 0.04 0.07 0.16 0 0.08  33 0.11 0 0.05 0.11 0 0.06 0.1 0 0.05 0.05 0 0.03 0.12 0.03 0.06 0.16 0 0.08  34 0.11 0 0.05 0.11 0 0.06 0.09 0 0.05 0.06 0 0.03 0.12 0 0.05 0.15 0.05 0.09  35 0.1 0 0.05 0.09 0 0.06 0.1 0 0.05 0.08 0 0.04 0.09 0.01 0.05 0.16 0 0.09  36 0.1 0 0.05 0.1 0 0.06 0.1 0 0.05 0.08 0.02 0.05 0.09 0.01 0.04 0.24 0 0.09  37 0.1 0 0.05 0.07 0 0.03 0.09 0 0.04 0.08 0 0.05 0.08 0 0.04 0.23 0 0.09  38 0.1 0 0.05 0.06 0 0.04 0.1 0 0.05 0.08 0 0.06 0.05 0 0.02 0.25 0 0.09  39 0.1 0 0.05 0.07 0 0.04 0.09 0 0.05 0.1 0 0.07 0.05 0 0.02 0.24 0 0.09  40 0.09 0 0.05 0.19 0 0.04 0.09 0 0.05 0.12 0.05 0.09 0.04 0 0.02 0.28 0 0.1  41 0.1 0 0.05 0.09 0 0.05 0.11 0 0.05 0.12 0 0.09 0.04 0 0.02 0.29 0 0.1  42 0.08 0 0.05 0.08 0 0.05 0.1 0 0.05 0.12 0.07 0.1 0.19 0 0.03 0.25 0 0.1  43 0.09 0 0.05 0.09 0 0.05 0.11 0 0.05 0.12 0 0.1 0.07 0.01 0.04 0.13 0 0.09  44 0.09 0.01 0.05 0.08 0 0.05 0.11 0 0.05 0.12 0 0.1 0.08 0 0.05 0.13 0 0.1  45 0.09 0 0.05 0.07 0 0.05 0.11 0 0.05 0.14 0 0.09 0.09 0 0.06 0.14 0.08 0.1  46 0.09 0.03 0.05 0.07 0 0.05 0.11 0 0.05 0.11 0 0.09 0.09 0 0.06 0.13 0 0.09  47 0.09 0 0.05 0.08 0 0.05 0.11 0 0.05 0.11 0.06 0.09 0.01 0 0.06 0.12 0 0.1  48 0.09 0 0.05 0.08 0 0.05 0.11 0 0.05 0.12 0.06 0.09 0.11 0 0.07 0.12 0 0.09  49 0.1 0 0.05 0.07 0.01 0.04 0.11 0 0.05 0.14 0.01 0.08 0.12 0 0.09 0.13 0 0.08  50 0.1 0 0.05 0.07 0.02 0.04 0.11 0 0.05 0.12 0 0.08 0.12 0.05 0.09 0.14 0 0.08  51 0.1 0 0.05 0.06 0 0.04 0.12 0 0.05 0.12 0 0.09 0.12 0 0.08 0.12 0 0.07  52 0.09 0 0.05 0.08 0 0.05 0.12 0 0.05 0.12 0.06 0.09 0.12 0 0.1 0.13 0.02 0.07  53 0.09 0 0.05 0.07 0 0.04 0.11 0 0.05 0.12 0.02 0.1 0.14 0 0.1 0.13 0 0.06  54 0.09 0 0.05 0.04 0 0.02 0.1 0 0.05 0.12 0.03 0.1 0.15 0.05 0.1 0.13 0 0.05  55 0.09 0 0.05 0.06 0 0.03 0.13 0 0.06 0.13 0.03 0.1 0.12 0 0.09 0.13 0 0.04  56 0.1 0 0.05 0.04 0 0.01 0.12 0 0.07 0.12 0.01 0.1 0.12 0 0.08 0.12 0 0.06  57 0.11 0 0.05 0.11 0 0.06 0.14 0 0.07 0.13 0 0.1 0.12 0 0.08 0.13 0 0.05  58 0.12 0 0.05 0.1 0 0.06 0.12 0 0.06 0.12 0.06 0.09 0.12 0 0.08 0.1 0 0.04  59 0.1 0 0.05 0.11 0 0.06 0.12 0 0.06 0.12 0 0.08 0.12 0.05 0.08 0.08 0 0.03  60 0.1 0 0.05 0.11 0 0.07 0.11 0 0.05 0.12 0.05 0.08 0.12 0 0.08 0.08 0 0.03  61 0.1 0 0.06 0.1 0 0.06 0.12 0 0.05 0.12 0 0.07 0.12 0 0.09 0.1 0 0.03  62 0.11 0 0.05 0.1 0 0.06 0.09 0 0.05 0.11 0 0.07 0.12 0 0.09 0.07 0 0.04  63 0.1 0 0.05 0.11 0 0.06 0.11 0 0.05 0.13 0 0.07 0.13 0.08 0.1 0.09 0 0.05  64 0.09 0 0.05 0.1 0 0.06 0.12 0 0.05 0.1 0.04 0.07 0.14 0.04 0.11 0.1 0 0.06  65 0.11 0 0.05 0.12 0 0.06 0.12 0 0.05 0.09 0.04 0.06 0.14 0.08 0.11 0.1 0 0.07  66 0.09 0 0.06 0.11 0 0.06 0.11 0 0.05 0.08 0.04 0.06 0.14 0 0.1 0.12 0 0.08  67 0.1 0 0.05 0.12 0 0.05 0.1 0 0.05 0.09 0.03 0.06 0.14 0.08 0.11 0.12 0.02 0.08  68 0.1 0.02 0.05 0.11 0 0.05 0.12 0 0.06 0.09 0 0.06 0.13 0 0.1 0.13 0 0.08  69 0.1 0.02 0.05 0.1 0 0.05 0.13 0.02 0.07 0.09 0 0.07 0.13 0 0.08 0.15 0 0.09  70 0.09 0 0.05 0.11 0 0.05 0.08 0 0.03 0.11 0 0.07 0.11 0 0.08 0.14 0 0.09  71 0.09 0 0.06 0.05 0 0.02 0.11 0 0.05 0.11 0 0.08 0.11 0 0.07 0.12 0.03 0.09  72 0.1 0.02 0.06 0.11 0 0.05 0.1 0 0.05 0.12 0 0.08 0.12 0.04 0.06 0.13 0 0.09  73 0.09 0 0.05 0.1 0 0.05 0.1 0 0.05 0.12 0.05 0.09 0.12 0 0.06 0.14 0 0.09  74 0.09 0.01 0.05 0.11 0 0.05 0.1 0 0.05 0.12 0 0.09 0.11 0.05 0.07 0.12 0.05 0.09  75 0.1 0 0.05 0.12 0 0.05 0.1 0 0.05 0.13 0.05 0.1 0.09 0 0.06 0.12 0 0.09  76 0.09 0 0.05 0.12 0 0.05 0.11 0 0.05 0.12 0.06 0.09 0.08 0 0.06 0.12 0.06 0.09  77 0.1 0 0.05 0.11 0 0.05 0.1 0 0.05 0.12 0 0.09 0.08 0.03 0.06 0.12 0 0.08  78 0.1 0 0.05 0.12 0 0.05 0.1 0 0.05 0.13 0.07 0.1 0.09 0.03 0.06 0.13 0 0.08  79 0.1 0 0.05 0.11 0 0.07 0.1 0 0.05 0.13 0.03 0.09 0.11 0 0.06 0.14 0 0.1  80 0.11 0 0.05 0.1 0.01 0.07 0.1 0 0.05 0.14 0.04 0.1 0.1 0 0.07 0.14 0 0.08  81 0.11 0 0.05 0.09 0 0.06 0.1 0 0.05 0.14 0.06 0.1 0.1 0 0.08 0.13 0.07 0.1  82 0.1 0 0.05 0.11 0 0.06 0.09 0 0.04 0.13 0.05 0.1 0.11 0 0.08 0.15 0 0.09  83 0.09 0 0.05 0.1 0 0.06 0.09 0 0.05 0.16 0.04 0.1 0.12 0 0.09 0.14 0.07 0.11  84 0.12 0 0.05 0.1 0.03 0.06 0.09 0 0.05 0.12 0.06 0.09 0.12 0.07 0.09 0.12 0 0.09  85 0.19 0 0.05 0.11 0 0.06 0.09 0 0.05 0.12 0.07 0.1 0.12 0 0.09 0.13 0 0.09  86 0.1 0.01 0.05 0.11 0 0.06 0.09 0 0.05 0.12 0 0.09 0.12 0 0.09 0.12 0 0.09  87 0.1 0 0.05 0.1 0.01 0.06 0.13 0 0.05 0.12 0 0.09 0.12 0 0.1 0.12 0 0.08  88 0.1 0 0.05 0.12 0 0.05 0.14 0 0.07 0.12 0.07 0.1 0.12 0 0.1 0.13 0 0.07  89 0.1 0 0.05 0.11 0 0.05 0.13 0 0.07 0.12 0.05 0.09 0.12 0.07 0.1 0.14 0 0.06  90 0.1 0 0.06 0.1 0 0.05 0.13 0 0.07 0.11 0.04 0.08 0.15 0.06 0.1 0.13 0 0.06  91 0.1 0 0.06 0.12 0 0.05 0.15 0 0.08 0.11 0 0.07 0.12 0 0.1 0.13 0 0.06  92 0.11 0.02 0.06 0.11 0 0.05 0.15 0.03 0.07 0.12 0 0.06 0.13 0.06 0.1 0.1 0.04 0.07  93 0.1 0 0.05 0.11 0 0.05 0.15 0 0.07 0.11 0 0.05 0.13 0.04 0.1 0.14 0 0.07  94 0.1 0 0.06 0.11 0 0.05 0.14 0 0.07 0.11 0 0.05 0.12 0 0.09 0.12 0 0.07  95 0.11 0 0.06 0.12 0 0.05 0.16 0 0.07 0.1 0 0.04 0.13 0 0.1 0.13 0 0.07  96 0.11 0 0.06 0.12 0 0.05 0.14 0 0.07 0.07 0 0.03 0.12 0.07 0.1 0.12 0 0.08  97 0.11 0 0.06 0.11 0 0.04 0.13 0 0.07 0.07 0 0.03 0.11 0 0.09 0.12 0 0.07  98 0.11 0 0.06 0.12 0 0.05 0.12 0 0.07 0.06 0 0.02 0.12 0 0.09 0.13 0 0.08  99 0.11 0 0.06 0.11 0 0.05 0.15 0 0.07 0.06 0 0.02 0.12 0.05 0.08 0.13 0 0.08  100 0.1 0 0.06 0.1 0 0.05 0.14 0.02 0.07 0.05 0 0.03 0.12 0.03 0.07 0.13 0 0.09  101 0.13 0 0.06 0.11 0 0.05 0.14 0.02 0.08 0.07 0 0.04 0.12 0.03 0.06 0.14 0.04 0.09  102 0.12 0 0.06 0.11 0 0.05 0.14 0 0.07 0.07 0 0.04 0.11 0 0.05 0.14 0 0.09  103 0.11 0 0.06 0.1 0 0.05 0.13 0 0.07 0.08 0 0.05 0.1 0 0.04 0.13 0 0.1  104 0.12 0 0.06 0.1 0 0.05 0.13 0 0.05 0.08 0 0.05 0.06 0 0.02 0.14 0 0.1  105 0.1 0 0.05 0.12 0 0.05 0.09 0 0.05 0.08 0.02 0.05 0.07 0 0.02 0.15 0.04 0.1  106 0.11 0.01 0.06 0.11 0 0.05 0.09 0 0.04 0.09 0.03 0.06 0.04 0 0.02 0.15 0.06 0.1  107 0.11 0.01 0.06 0.11 0 0.05 0.1 0 0.04 0.12 0.03 0.08 0.04 0 0.01 0.14 0.05 0.1  108 0.1 0 0.05 0.12 0 0.05 0.1 0 0.04 0.12 0 0.07 0.04 0 0.02 0.15 0.03 0.1  109 0.11 0 0.05 0.11 0 0.05 0.1 0 0.05 0.12 0 0.08 0.06 0 0.03 0.14 0 0.1  110 0.09 0 0.05 0.11 0 0.05 0.09 0 0.04 0.12 0 0.09 0.07 0 0.04 0.13 0 0.8  111 0.1 0 0.05 0.1 0 0.05 0.08 0 0.04 0.12 0 0.09 0.07 0 0.04 0.14 0.08 0.11  112 0.11 0 0.06 0.12 0 0.05 0.08 0 0.04 0.12 0.05 0.1 0.09 0 0.05 0.14 0.08 0.11  113 0.12 0 0.06 0.1 0 0.05 0.1 0 0.04 0.12 0.06 0.1 0.07 0 0.05 0.13 0 0.1  114 0.12 0.01 0.05 0.1 0 0.05 0.07 0 0.04 0.12 0.02 0.09 0.09 0 0.06 0.14 0 0.08  115 0.1 0 0.06 0.09 0 0.05 0.08 0 0.04 0.12 0.03 0.1 0.09 0 0.07 0.13 0 0.07  116 0.1 0 0.05 0.09 0 0.05 0.11 0 0.04 0.12 0.03 0.1 0.1 0.03 0.07 0.13 0 0.08  117 0.1 0 0.05 0.11 0 0.05 0.09 0 0.04 0.12 0.01 0.1 0.12 0 0.08 0.12 0.05 0.08  118 0.12 0 0.05 0.11 0 0.05 0.09 0 0.05 0.12 0.03 0.09 0.12 0.06 0.09 0.14 0 0.07  119 0.12 0 0.05 0.1 0 0.05 0.1 0 0.04 0.13 0.03 0.1 0.13 0 0.1 0.13 0 0.06  120 0.11 0 0.05 0.1 0 0.05 0.09 0 0.05 0.13 0.06 0.1 0.14 0.05 0.1 0.14 0 0.06  121 0.1 0 0.05 0.11 0 0.04 0.09 0 0.04 0.13 0 0.1 0.13 0 0.1 0.14 0 0.07  122 0.11 0 0.06 0.09 0 0.05 0.1 0 0.05 0.12 0.08 0.1 0.13 0 0.1 0.13 0 0.06  123 0.11 0 0.05 0.1 0 0.05 0.09 0 0.04 0.13 0 0.09 0.13 0.06 0.1 0.12 0.03 0.06  124 0.12 0 0.06 0.11 0 0.04 0.09 0 0.05 0.14 0 0.08 0.12 0.06 0.1 0.11 0 0.06  125 0.11 0 0.06 0.09 0 0.04 0.08 0 0.05 0.13 0.05 0.08 0.12 0.05 0.09 0.1 0 0.07  126 0.1 0 0.06 0.11 0 0.05 0.1 0 0.04 0.13 0 0.8 0.12 0 0.09 0.11 0 0.07  127 0.12 0 0.06 0.1 0 0.05 0.11 0 0.05 0.12 0 0.07 0.12 0.06 0.1 0.12 0 0.07  128 0.12 0 0.06 0.11 0 0.05 0.09 0 0.05 0.11 0 0.07 0.12 0 0.1 0.14 0 0.09  129 0.11 0 0.06 0.1 0 0.05 0.09 0 0.05 0.12 0 0.06 0.13 0 0.1 0.14 0 0.09  130 0.12 0 0.06 0.09 0 0.04 0.1 0 0.05 0.09 0 0.05 0.15 0.08 0.11 0.13 0 0.09  131 0.11 0 0.06 0.1 0 0.05 0.12 0.02 0.07 0.09 0 0.06 0.13 0 0.09 0.14 0 0.09  132 0.12 0 0.05 0.12 0.01 0.07 0.13 0 0.07 0.08 0 0.05 0.14 0.06 0.1 0.16 0 0.1  133 0.11 0 0.06 0.13 0 0.06 0.13 0 0.07 0.08 0 0.05 0.14 0 0.09 0.14 0.03 0.1  134 0.12 0 0.06 0.15 0 0.07 0.12 0.02 0.07 0.08 0 0.05 0.14 0 0.08 0.13 0 0.08  135 0.12 0 0.06 0.15 0 0.07 0.13 0 0.07 0.09 0 0.06 0.12 0 0.08 0.14 0.04 0.1  136 0.12 0 0.06 0.15 0.01 0.07 0.12 0 0.07 0.1 0 0.07 0.11 0 0.07 0.13 0 0.1  137 0.12 0 0.05 0.16 0 0.06 0.14 0.02 0.07 0.12 0 0.08 0.1 0 0.07 0.14 0.07 0.1  138 0.12 0 0.06 0.18 0 0.07 0.13 0.03 0.07 0.12 0 0.09 0.11 0 0.07 0.14 0 0.09  139 0.12 0 0.06 0.16 0 0.06 0.13 0 0.07 0.12 0 0.09 0.11 0.03 0.06 0.14 0 0.11  140 0.12 0 0.06 0.1 0 0.04 0.17 0.02 0.07 0.13 0.06 0.1 0.08 0 0.06 0.14 0 0.1  141 0.11 0 0.05 0.1 0 0.04 0.13 0.01 0.07 0.13 0.05 0.1 0.08 0 0.06 0.14 0 0.11  142 0.12 0 0.06 0.1 0 0.04 0.14 0 0.07 0.12 0 0.1 0.08 0 0.06 0.15 0 0.11  143 0.11 0 0.06 0.09 0 0.05 0.12 0 0.05 0.18 0.07 0.1 0.08 0 0.05 0.13 0 0.09  144 0.12 0 0.05 0.1 0 0.05 0.12 0 0.05 0.13 0 0.1 0.08 0.03 0.06 0.15 0.07 0.11  145 0.12 0 0.05 0.1 0 0.05 0.11 0 0.05 0.13 0 0.1 0.11 0.02 0.07 0.14 0 0.1  146 0.11 0 0.05 0.1 0 0.05 0.1 0 0.05 0.13 0.04 0.1 0.12 0 0.07 0.13 0 0.09  147 0.1 0 0.05 0.09 0 0.05 0.12 0 0.05 0.16 0.07 0.11 0.11 0 0.08 0.13 0.04 0.09  148 0.12 0 0.05 0.09 0 0.04 0.13 0 0.07 0.16 0.06 0.11 0.12 0 0.09 0.14 0.06 0.09  149 0.11 0 0.06 0.09 0 0.05 0.15 0 0.07 0.16 0.07 0.12 0.13 0.06 0.1 0.13 0 0.09  150 0.11 0 0.05 0.08 0 0.04 0.13 0 0.07 0.18 0 0.11 0.13 0.06 0.1 0.13 0 0.09  151 0.12 0 0.06 0.08 0 0.05 0.13 0 0.07 0.16 0.06 0.11 0.13 0 0.11 0.16 0 0.09  152 0.12 0 0.06 0.09 0 0.04 0.14 0 0.07 0.15 0.05 0.1 0.14 0 0.1 0.14 0 0.09  153 0.12 0 0.06 0.08 0 0.04 0.12 0 0.05 0.13 0 0.09 0.13 0 0.1 0.13 0 0.08  154 0.11 0 0.06 0.08 0 0.04 0.12 0 0.05 0.13 0 0.08 0.17 0.07 0.1 0.14 0.04 0.09  155 0.11 0 0.05 0.08 0 0.04 0.11 0 0.05 0.12 0.06 0.09 0.13 0 0.09 0.14 0 0.09  156 0.12 0 0.05 0.08 0 0.04 0.1 0 0.05 0.13 0.05 0.08 0.13 0 0.09 0.16 0.04 0.09  157 0.11 0 0.05 0.08 0 0.04 0.12 0 0.05 0.12 0.04 0.07 0.13 0.08 0.1 0.15 0 0.1  158 0.11 0 0.05 0.09 0 0.04 0.07 0 0.04 0.11 0 0.07 0.14 0 0.1 0.15 0 0.1  159 0.12 0 0.06 0.08 0 0.04 0.19 0 0.04 0.11 0.03 0.06 0.14 0.05 0.11 0.16 0.03 0.1  160 0.11 0 0.05 0.09 0 0.04 0.13 0 0.06 0.1 0.03 0.06 0.14 0 0.11 0.15 0 0.1  161 0.12 0 0.05 0.08 0 0.05 0.13 0 0.07 0.11 0.05 0.06 0.14 0 0.09 0.15 0 0.1  162 0.12 0 0.05 0.13 0 0.05 0.12 0 0.07 0.08 0 0.05 0.14 0.07 0.011 0.14 0 0.1  163 0.12 0 0.05 0.08 0 0.04 0.12 0 0.07 0.07 0.03 0.05 0.16 0.07 0.1 0.15 0.06 0.11  164 0.12 0 0.04 0.09 0 0.03 0.12 0 0.07 0.07 0.02 0.05 0.13 0.06 0.09 0.15 0.07 0.11  165 0.12 0 0.05 0.09 0 0.04 0.13 0 0.07 0.07 0 0.05 0.12 0.04 0.08 0.16 0.05 0.12  166 0.11 0 0.05 0.08 0 0.04 0.13 0 0.07 0.1 0 0.06 0.12 0.05 0.08 0.16 0.07 0.12  167 0.11 0 0.05 0.08 0 0.03 0.13 0 0.07 0.12 0.02 0.07 0.12 0 0.06 0.16 0.05 0.11  168 0.1 0 0.05 0.09 0 0.04 0.12 0 0.07 0.12 0 0.08 0.09 0 0.06 0.15 0.03 0.1  169 0.11 0 0.05 0.09 0 0.04 0.14 0 0.07 0.12 0 0.08 0.22 0.03 0.07 0.14 0 0.1  170 0.11 0 0.05 0.09 0 0.04 0.14 0 0.07 0.14 0 0.09 0.08 0 0.05 0.14 0.05 0.1  171 0.1 0 0.05 0.08 0 0.03 0.13 0 0.07 0.13 0.04 0.1 0.08 0 0.06 0.13 0.06 0.09  172 0.1 0 0.05 0.09 0 0.04 0.12 0 0.07 0.13 0 0.1 0.09 0 0.06 0.15 0 0.09  173 0.12 0 0.05 0.09 0 0.05 0.12 0 0.07 0.14 0.04 0.11 0.08 0 0.05 0.15 0 0.07  174 0.11 0 0.05 0.08 0 0.04 0.13 0 0.07 0.16 0.03 0.11 0.08 0 0.05 0.13 0 0.04  175 0.11 0 0.04 0.08 0 0.03 0.12 0 0.07 0.16 0.07 0.11 0.07 0.04 0.05 0.12 0 0.05  176 0.12 0 0.05 0.09 0 0.04 0.12 0 0.07 0.14 0.02 0.11 0.1 0.02 0.06 0.14 0 0.05  177 0.11 0 0.05 0.09 0 0.04 0.12 0 0.07 0.15 0.07 0.11 0.12 0.01 0.07 0.12 0 0.04  178 0.11 0 0.05 0.08 0 0.04 0.12 0 0.07 0.15 0.04 0.1 0.12 0 0.08 0.14 0 0.05  179 0.1 0 0.05 0.08 0 0.04 0.13 0 0.07 0.15 0.03 0.1 0.11 0 0.08 0.13 0 0.05  180 0.12 0 0.05 0.08 0 0.04 0.12 0 0.07 0.15 0 0.1 0.14 0 0.09 0.09 0 0.05  181 0.1 0 0.05 0.06 0 0.03 0.12 0 0.07 0.14 0.06 0.1 0.14 0.06 0.11 0.08 0 0.04  182 0.1 0 0.05 0.07 0 0.04 0.12 0 0.07 0.12 0.05 0.09 0.13 0 0.1 0.11 0 0.06  183 0.09 0 0.05 0.06 0 0.03 0.14 0 0.07 0.14 0.05 0.09 0.14 0.07 0.11 0.1 0 0.05  184 0.09 0 0.05 0.08 0 0.03 0.14 0 0.07 0.12 0.04 0.09 0.13 0 0.11 0.09 0 0.06  185 0.09 0 0.05 0.06 0 0.02 0.14 0 0.07 0.12 0.02 0.09 0.13 0 0.1 0.11 0 0.07  186 0.11 0 0.05 0.06 0 0.02 0.15 0 0.07 0.14 0.05 0.08 0.13 0.08 0.11 0.14 0.01 0.08  187 0.11 0 0.05 0.07 0 0.03 0.14 0 0.07 0.12 0 0.07 0.13 0.05 0.1 0.13 0 0.08  188 0.1 0 0.05 0.07 0 0.02 0.15 0 0.07 0.12 0 0.07 0.13 0.06 0.1 0.15 0.01 0.09  189 0.1 0 0.05 0.04 0 0.02 0.13 0 0.07 0.12 0.03 0.07 0.13 0.07 0.1 0.13 0 0.1  190 0.1 0 0.04 0.06 0.01 0.03 0.12 0 0.06 0.1 0.03 0.07 0.15 0 0.1 0.14 0.02 0.1  191 0.09 0 0.05 0.09 0 0.03 0.11 0 0.06 0.1 0 0.06 0.13 0.06 0.1 0.17 0 0.11  192 0.1 0 0.05 0.07 0 0.03 0.12 0 0.06 0.11 0.05 0.07 0.12 0.08 0.1 0.15 0 0.1  193 0.11 0 0.04 0.04 0 0.02 0.12 0 0.06 0.11 0 0.07 0.12 0.04 0.1 0.16 0.03 0.1  194 0.09 0 0.04 0.12 0 0.05 0.12 0 0.06 0.12 0.05 0.08 0.13 0.06 0.09 0.18 0 0.11  195 0.11 0 0.05 0.12 0 0.05 0.12 0 0.05 0.12 0 0.08 0.12 0 0.09 0.18 0.05 0.11  196 0.1 0 0.05 0.11 0 0.05 0.12 0 0.05 0.12 0 0.1 0.11 0.05 0.09 0.16 0 0.1  197 0.11 0 0.05 0.1 0 0.05 0.11 0 0.05 0.14 0 0.1 0.01 0.04 0.07 0.16 0 0.11  198 0.1 0 0.05 0.12 0 0.06 0.1 0 0.05 0.15 0 0.1 0.11 0 0.07 0.14 0.06 0.11  199 0.09 0 0.04 0.13 0 0.07 0.12 0 0.05 0.15 0 0.11 0.11 0 0.07 0.15 0.07 0.11  200 0.1 0 0.05 0.13 0.01 0.07 0.12 0 0.05 0.15 0.01 0.11 0.1 0 0.06 0.14 0 0.09 |
| --- |

| Control (mm) Infected (mm)  Day 5 R1 R2 R3 R1 R2 R3  Max Min Average Max Min Average Max Min Average Max Min Average Max Min Average Max Min Average  1 0.08 0 0.04 0.07 0 0.03 0.09 0 0.03 0.1 0 0.06 0.11 0 0.05 0.11 0 0.06  2 0.09 0 0.04 0.07 0.01 0.04 0.09 0 0.03 0.1 0 0.06 0.12 0 0.06 0.12 0 0.07  3 0.08 0 0.04 0.07 0 0.03 0.09 0 0.03 0.1 0 0.06 0.12 0 0.06 0.1 0 0.06  4 0.09 0 0.04 0.09 0 0.04 0.1 0 0.03 0.12 0.02 0.06 0.11 0 0.06 0.11 0 0.07  5 0.09 0 0.04 0.01 0 0.04 0.08 0 0.03 0.11 0 0.06 0.1 0 0.06 0.12 0.02 0.07  6 0.09 0 0.04 0.09 0 0.04 0.08 0 0.03 0.13 0 0.07 0.13 0 0.06 0.11 0 0.06  7 0.07 0 0.03 0.06 0 0.03 0.06 0 0.03 0.15 0 0.07 0.12 0 0.06 0.1 0.02 0.06  8 0.09 0 0.03 0.08 0 0.04 0.07 0 0.02 0.1 0.01 0.06 0.1 0 0.06 0.09 0 0.06  9 0.09 0 0.04 0.08 0 0.04 0.06 0 0.03 0.09 0.02 0.06 0.11 0 0.06 0.09 0.01 0.06  10 0.1 0 0.04 0.07 0 0.03 0.09 0 0.04 0.11 0.01 0.05 0.1 0 0.06 0.1 0 0.06  11 0.09 0 0.04 0.1 0 0.04 0.07 0 0.03 0.1 0 0.06 0.1 0 0.06 0.1 0 0.06  12 0.08 0 0.03 0.07 0 0.03 0.09 0 0.04 0.12 0 0.06 0.18 0 0.06 0.1 0 0.06  13 0.09 0 0.03 0.08 0 0.03 0.09 0 0.04 0.1 0 0.05 0.1 0 0.06 0.1 0 0.06  14 0.1 0 0.04 0.09 0 0.04 0.07 0 0.03 0.1 0 0.05 0.1 0 0.06 0.12 0 0.06  15 0.08 0 0.04 0.08 0 0.03 0.09 0 0.04 0.12 0 0.05 0.1 0 0.06 0.11 0 0.06  16 0.09 0 0.04 0.1 0 0.04 0.09 0 0.04 0.09 0 0.05 0.11 0 0.07 0.11 0 0.06  17 0.09 0 0.04 0.11 0 0.04 0.1 0 0.04 0.09 0 0.05 0.22 0 0.08 0.12 0 0.06  18 0.1 0 0.04 0.09 0 0.04 0.09 0 0.04 0.11 0 0.05 0.13 0 0.07 0.1 0 0.06  19 0.08 0 0.04 0.1 0 0.04 0.08 0 0.04 0.11 0.02 0.06 0.18 0.04 0.07 0.11 0 0.06  20 0.1 0 0.04 0.08 0 0.04 0.09 0 0.04 0.1 0 0.06 0.14 0 0.07 0.12 0 0.06  21 0.11 0 0.04 0.08 0 0.04 0.08 0 0.04 0.18 0 0.06 0.1 0 0.06 0.11 0 0.06  22 0.1 0 0.04 0.08 0.01 0.06 0.09 0 0.03 0.11 0 0.06 0.21 0 0.08 0.11 0 0.06  23 0.08 0 0.04 0.09 0 0.03 0.08 0 0.04 0.11 0 0.06 0.1 0 0.06 0.11 0 0.06  24 0.09 0 0.04 0.1 0 0.04 0.08 0 0.04 0.11 0 0.06 0.1 0 0.06 0.11 0 0.06  25 0.09 0 0.04 0.1 0 0.04 0.09 0 0.03 0.12 0 0.07 0.12 0 0.07 0.12 0 0.07  26 0.08 0 0.04 0.08 0 0.04 0.1 0 0.04 0.09 0 0.07 0.11 0 0.06 0.12 0 0.07  27 0.08 0 0.04 0.08 0 0.04 0.1 0 0.03 0.11 0 0.07 0.1 0 0.06 0.12 0 0.07  28 0.11 0 0.04 0.08 0 0.04 0.08 0 0.03 0.1 0.02 0.06 0.16 0 0.07 0.11 0 0.07  29 0.08 0 0.04 0.08 0 0.04 0.1 0 0.04 0.11 0 0.06 0.12 0 0.07 0.11 0 0.07  30 0.08 0 0.04 0.08 0 0.04 0.1 0 0.04 0.12 0 0.06 0.13 0.03 0.07 0.12 0 0.07  31 0.08 0.01 0.06 0.09 0 0.04 0.08 0 0.03 0.13 0 0.07 0.13 0.01 0.08 0.1 0 0.07  32 0.1 0 0.06 0.09 0 0.03 0.07 0 0.03 0.11 0.02 0.07 0.11 0 0.06 0.11 0.02 0.07  33 0.08 0 0.06 0.1 0 0.04 0.08 0 0.03 0.12 0 0.07 0.12 0.01 0.06 0.12 0 0.06  34 0.08 0 0.06 0.09 0 0.04 0.08 0 0.03 0.12 0 0.06 0.1 0 0.06 0.11 0.02 0.07  35 0.08 0 0.06 0.1 0 0.04 0.08 0 0.03 0.11 0 0.06 0.11 0 0.06 0.12 0.02 0.07  36 0.09 0 0.06 0.09 0 0.04 0.09 0 0.03 0.11 0 0.06 0.13 0 0.06 0.16 0.01 0.08  37 0.09 0 0.06 0.09 0 0.04 0.1 0 0.04 0.11 0 0.05 0.11 0 0.06 0.11 0 0.07  38 0.1 0 0.06 0.09 0 0.04 0.08 0 0.03 0.09 0 0.05 0.1 0 0.06 0.12 0.01 0.08  39 0.1 0 0.06 0.1 0 0.04 0.08 0 0.03 0.09 0 0.05 0.1 0 0.06 0.12 0 0.08  40 0.08 0 0.06 0.1 0 0.04 0.07 0 0.03 0.08 0 0.05 0.1 0 0.06 0.12 0.01 0.08  41 0.11 0 0.06 0.1 0 0.04 0.1 0 0.04 0.09 0 0.04 0.08 0 0.06 0.12 0 0.08  42 0.1 0 0.06 0.11 0 0.03 0.1 0 0.04 0.08 0 0.05 0.09 0 0.06 0.12 0 0.08  43 0.08 0 0.06 0.07 0 0.04 0.1 0 0.04 0.09 0 0.04 0.08 0 0.06 0.12 0.03 0.07  44 0.08 0 0.06 0.1 0 0.04 0.1 0 0.04 0.08 0 0.04 0.08 0 0.06 0.12 0.03 0.07  45 0.1 0 0.06 0.1 0 0.04 0.09 0 0.04 0.09 0 0.05 0.08 0 0.06 0.11 0 0.07  46 0.1 0 0.04 0.1 0 0.04 0.1 0 0.04 0.09 0 0.04 0.09 0 0.06 0.11 0 0.07  47 0.1 0 0.03 0.09 0 0.04 0.08 0 0.04 0.08 0 0.04 0.09 0 0.06 0.12 0.05 0.07  48 0.1 0 0.03 0.09 0 0.05 0.09 0 0.04 0.14 0 0.04 0.1 0.01 0.06 0.11 0 0.08  49 0.11 0 0.03 0.1 0 0.05 0.08 0 0.04 0.09 0 0.05 0.11 0 0.06 0.12 0 0.07  50 0.08 0 0.03 0.1 0.01 0.05 0.08 0 0.04 0.21 0 0.06 0.09 0 0.06 0.14 0.04 0.07  51 0.08 0 0.03 0.09 0.01 0.06 0.08 0 0.04 0.08 0 0.04 0.24 0 0.07 0.12 0.03 0.07  52 0.08 0 0.04 0.09 0 0.06 0.08 0 0.04 0.09 0 0.05 0.11 0 0.06 0.11 0 0.07  53 0.1 0 0.04 0.09 0 0.06 0.08 0 0.04 0.09 0 0.06 0.1 0 0.06 0.1 0.05 0.08  54 0.1 0 0.05 0.1 0 0.06 0.11 0 0.04 0.24 0 0.05 0.1 0 0.06 0.12 0 0.08  55 0.09 0 0.04 0.1 0 0.06 0.07 0 0.04 0.11 0 0.06 0.1 0.01 0.07 0.12 0 0.08  56 0.09 0 0.04 0.11 0 0.05 0.08 0 0.04 0.27 0 0.07 0.1 0.01 0.07 0.12 0 0.08  57 0.1 0 0.05 0.09 0 0.03 0.09 0 0.04 0.11 0 0.07 0.11 0 0.07 0.15 0 0.07  58 0.1 0 0.04 0.08 0 0.04 0.08 0 0.04 0.1 0 0.07 0.11 0 0.06 0.15 0 0.07  59 0.08 0 0.05 0.09 0 0.04 0.07 0 0.04 0.09 0 0.06 0.11 0 0.07 0.14 0 0.07  60 0.1 0 0.05 0.08 0 0.04 0.09 0 0.04 0.1 0 0.06 0.11 0 0.07 0.12 0 0.08  61 0.09 0 0.04 0.08 0 0.04 0.08 0 0.04 0.11 0 0.06 0.13 0 0.06 0.11 0 0.07  62 0.09 0 0.04 0.09 0 0.03 0.08 0 0.04 0.11 0 0.07 0.09 0 0.06 0.11 0.04 0.08  63 0.09 0 0.05 0.09 0 0.04 0.07 0 0.03 0.11 0 0.07 0.11 0 0.07 0.12 0 0.08  64 0.09 0 0.05 0.08 0 0.04 0.12 0 0.04 0.11 0 0.07 0.1 0 0.06 0.14 0 0.08  65 0.1 0 0.05 0.09 0 0.03 0.08 0 0.04 0.1 0.02 0.06 0.09 0 0.06 0.12 0 0.08  66 0.09 0 0.05 0.1 0 0.04 0.09 0 0.03 0.11 0 0.06 0.11 0.03 0.07 0.12 0 0.08  67 0.1 0 0.05 0.09 0 0.04 0.1 0 0.04 0.1 0 0.06 0.1 0 0.06 0.13 0 0.09  68 0.11 0 0.05 0.1 0 0.04 0.1 0 0.04 0.11 0.03 0.06 0.09 0 0.06 0.12 0 0.08  69 0.12 0 0.05 0.1 0 0.03 0.09 0 0.04 0.09 0 0.06 0.11 0.03 0.06 0.12 0 0.07  70 0.09 0 0.04 0.09 0 0.04 0.09 0 0.04 0.08 0 0.05 0.12 0 0.06 0.11 0 0.07  71 0.08 0 0.04 0.1 0 0.04 0.1 0 0.04 0.09 0 0.05 0.11 0 0.07 0.11 0 0.07  72 0.07 0 0.04 0.09 0 0.04 0.1 0 0.04 0.08 0 0.05 0.1 0 0.06 0.13 0 0.06  73 0.08 0 0.04 0.08 0 0.04 0.09 0 0.04 0.1 0 0.05 0.1 0.02 0.07 0.11 0 0.07  74 0.08 0 0.04 0.08 0 0.04 0.09 0 0.04 0.1 0.02 0.05 0.1 0.03 0.07 0.11 0 0.06  75 0.09 0 0.04 0.09 0 0.04 0.08 0 0.04 0.09 0 0.05 0.11 0.03 0.07 0.12 0 0.06  76 0.08 0 0.04 0.1 0 0.05 0.08 0 0.04 0.1 0 0.05 0.09 0 0.06 0.11 0 0.06  77 0.07 0 0.03 0.1 0 0.05 0.08 0 0.04 0.23 0 0.05 0.11 0 0.06 0.3 0 0.06  78 0.06 0 0.03 0.1 0 0.05 0.08 0 0.04 0.08 0 0.05 0.1 0 0.06 0.12 0 0.07  79 0.08 0 0.04 0.1 0 0.05 0.09 0 0.04 0.08 0.02 0.06 0.09 0 0.06 0.1 0 0.06  80 0.09 0 0.04 0.1 0 0.05 0.08 0 0.04 0.11 0.01 0.06 0.09 0 0.06 0.11 0 0.06  81 0.08 0.01 0.04 0.1 0 0.05 0.09 0 0.04 0.1 0 0.05 0.09 0 0.06 0.09 0 0.06  82 0.09 0 0.04 0.1 0 0.05 0.08 0 0.05 0.09 0 0.06 0.11 0 0.06 0.1 0 0.06  83 0.08 0 0.04 0.1 0 0.05 0.1 0 0.05 0.1 0 0.05 0.12 0 0.06 0.1 0 0.05  84 0.09 0 0.05 0.1 0 0.05 0.1 0 0.05 0.1 0 0.06 0.17 0 0.07 0.11 0 0.06  85 0.09 0 0.04 0.09 0 0.05 0.1 0 0.05 0.09 0 0.05 0.13 0 0.07 0.12 0 0.06  86 0.09 0 0.04 0.09 0 0.05 0.09 0 0.06 0.14 0 0.06 0.1 0 0.06 0.11 0 0.07  87 0.08 0 0.04 0.1 0 0.05 0.1 0 0.06 0.1 0 0.06 0.11 0 0.06 0.12 0 0.07  88 0.08 0 0.04 0.09 0 0.05 0.11 0 0.06 0.1 0 0.05 0.08 0 0.06 0.12 0 0.07  89 0.09 0 0.04 0.23 0 0.06 0.12 0 0.06 0.1 0 0.06 0.1 0 0.06 0.11 0 0.07  90 0.09 0 0.05 0.09 0 0.06 0.11 0 0.06 0.1 0 0.06 0.18 0 0.06 0.12 0 0.08  91 0.09 0 0.05 0.09 0 0.06 0.11 0 0.06 0.11 0.01 0.06 0.1 0.01 0.06 0.11 0 0.08  92 0.09 0 0.05 0.09 0 0.06 0.11 0 0.06 0.1 0 0.06 0.19 0 0.06 0.11 0 0.07  93 0.09 0 0.05 0.09 0 0.06 0.11 0 0.06 0.11 0 0.06 0.15 0 0.06 0.11 0 0.08  94 0.1 0.02 0.05 0.1 0 0.04 0.11 0 0.06 0.14 0 0.06 0.19 0 0.06 0.12 0 0.08  95 0.1 0 0.05 0.09 0 0.04 0.1 0 0.06 0.07 0 0.04 0.24 0 0.06 0.11 0 0.08  96 0.09 0 0.05 0.08 0 0.04 0.11 0 0.06 0.07 0 0.04 0.09 0 0.06 0.13 0 0.08  97 0.09 0 0.05 0.08 0 0.04 0.1 0 0.06 0.09 0 0.05 0.13 0 0.06 0.12 0 0.07  98 0.09 0 0.04 0.09 0 0.04 0.1 0 0.06 0.1 0 0.04 0.08 0 0.06 0.12 0 0.08  99 0.09 0 0.04 0.09 0 0.04 0.11 0 0.06 0.09 0 0.04 0.1 0 0.06 0.12 0 0.07  100 0.09 0 0.04 0.08 0 0.04 0.1 0 0.06 0.08 0 0.06 0.1 0 0.06 0.11 0 0.07  101 0.08 0 0.04 0.07 0 0.04 0.1 0 0.06 0.09 0 0.06 0.13 0 0.06 0.12 0.03 0.07  102 0.11 0 0.04 0.08 0 0.03 0.1 0 0.06 0.27 0 0.06 0.1 0 0.06 0.11 0.03 0.07  103 0.09 0 0.05 0.08 0 0.03 0.09 0 0.06 0.1 0 0.06 0.12 0 0.07 0.11 0 0.07  104 0.1 0 0.04 0.08 0 0.04 0.1 0 0.06 0.08 0.01 0.06 0.11 0 0.07 0.13 0 0.06  105 0.09 0 0.04 0.09 0 0.04 0.09 0 0.06 0.09 0 0.06 0.11 0 0.06 0.11 0.02 0.07  106 0.09 0 0.05 0.07 0 0.04 0.09 0 0.06 0.09 0 0.06 0.17 0 0.07 0.11 0 0.06  107 0.09 0 0.05 0.09 0 0.04 0.08 0 0.06 0.09 0 0.06 0.1 0 0.07 0.11 0 0.06  108 0.09 0 0.04 0.07 0 0.04 0.08 0 0.05 0.11 0.01 0.06 0.1 0 0.07 0.11 0 0.07  109 0.09 0 0.03 0.09 0 0.04 0.08 0 0.04 0.1 0 0.06 0.09 0 0.07 0.11 0 0.07  110 0.1 0 0.04 0.08 0 0.04 0.09 0.01 0.06 0.09 0 0.06 0.1 0 0.06 0.11 0 0.07  111 0.11 0 0.04 0.08 0 0.04 0.08 0 0.05 0.09 0 0.06 0.12 0 0.07 0.12 0 0.08  112 0.11 0 0.04 0.08 0 0.04 0.09 0.01 0.06 0.09 0 0.06 0.12 0 0.07 0.14 0 0.07  113 0.1 0 0.04 0.08 0 0.04 0.1 0.01 0.06 0.09 0 0.06 0.1 0.02 0.07 0.11 0 0.08  114 0.1 0 0.04 0.07 0 0.04 0.09 0 0.06 0.09 0 0.06 0.11 0 0.07 0.12 0.03 0.08  115 0.1 0 0.04 0.07 0 0.04 0.09 0 0.06 0.11 0 0.07 0.11 0 0.07 0.12 0.02 0.08  116 0.09 0 0.04 0.08 0 0.04 0.08 0 0.06 0.09 0 0.07 0.11 0 0.07 0.18 0 0.08  117 0.09 0 0.04 0.1 0 0.05 0.08 0 0.05 0.11 0 0.06 0.11 0 0.07 0.16 0 0.07  118 0.08 0 0.04 0.1 0 0.05 0.09 0 0.05 0.11 0 0.06 0.09 0.02 0.06 0.11 0 0.07  119 0.1 0 0.05 0.09 0 0.06 0.09 0 0.04 0.11 0 0.07 0.09 0.02 0.06 0.18 0 0.09  120 0.08 0 0.04 0.09 0 0.06 0.08 0 0.04 0.15 0 0.06 0.09 0 0.06 0.16 0 0.09  121 0.09 0 0.05 0.08 0 0.06 0.09 0 0.04 0.12 0.01 0.06 0.09 0.02 0.05 0.15 0 0.1  122 0.08 0 0.05 0.08 0 0.04 0.08 0 0.04 0.13 0 0.06 0.09 0.03 0.06 0.16 0.03 0.09  123 0.09 0 0.05 0.09 0 0.04 0.09 0 0.04 0.12 0 0.06 0.09 0 0.06 0.13 0 0.08  124 0.09 0.01 0.06 0.09 0 0.04 0.09 0 0.04 0.11 0 0.06 0.1 0 0.06 0.15 0 0.08  125 0.09 0 0.06 0.09 0 0.04 0.08 0 0.04 0.09 0.04 0.06 0.1 0 0.07 0.15 0 0.08  126 0.1 0 0.05 0.09 0 0.04 0.09 0 0.04 0.08 0.02 0.06 0.11 0 0.07 0.12 0 0.06  127 0.09 0 0.05 0.11 0 0.04 0.1 0 0.04 0.08 0 0.06 0.09 0.03 0.07 0.12 0 0.06  128 0.09 0 0.05 0.09 0 0.04 0 0 0.04 0.09 0.03 0.06 0.1 0 0.07 0.14 0 0.06  129 0.09 0 0.05 0.08 0 0.04 0.07 0 0.04 0.09 0 0.06 0.1 0 0.07 0.1 0 0.06  130 0.09 0 0.05 0.09 0 0.05 0.09 0 0.04 0.09 0 0.06 0.1 0 0.07 0.12 0 0.07  131 0.09 0 0.04 0.08 0 0.04 0.09 0 0.04 0.09 0 0.06 0.1 0.02 0.07 0.12 0 0.06  132 0.08 0 0.05 0.08 0 0.04 0.08 0 0.04 0.09 0 0.06 0.11 0.02 0.07 0.12 0 0.07  133 0.08 0 0.05 0.07 0 0.05 0.09 0 0.04 0.1 0 0.07 0.1 0 0.07 0.1 0 0.06  134 0.08 0 0.05 0.08 0 0.06 0.08 0 0.04 0.1 0 0.06 0.12 0 0.07 0.1 0.03 0.06  135 0.08 0 0.05 0.07 0 0.06 0.09 0 0.04 0.09 0 0.07 0.13 0 0.07 0.11 0 0.06  136 0.08 0 0.05 0.09 0 0.04 0.09 0 0.04 0.1 0 0.07 0.12 0.02 0.08 0.11 0.02 0.06  137 0.08 0 0.04 0.08 0 0.04 0.09 0 0.04 0.12 0 0.07 0.12 0.03 0.07 0.13 0 0.07  138 0.09 0 0.04 0.09 0 0.05 0.09 0 0.05 0.12 0.02 0.07 0.13 0.04 0.08 0.11 0 0.06  139 0.08 0.01 0.04 0.09 0 0.05 0.09 0.01 0.06 0.11 0.01 0.07 0.12 0.04 0.08 0.11 0 0.07  140 0.09 0 0.05 0.09 0 0.05 0.1 0 0.06 0.11 0.01 0.08 0.12 0 0.07 0.12 0 0.07  141 0.09 0 0.05 0.09 0 0.05 0.08 0 0.06 0.13 0 0.08 0.11 0 0.07 0.12 0 0.07  142 0.09 0 0.05 0.09 0 0.05 0.09 0 0.06 0.11 0 0.07 0.1 0 0.06 0.11 0 0.07  143 0.08 0 0.05 0.09 0 0.05 0.09 0 0.06 0.12 0 0.06 0.1 0.03 0.06 0.13 0 0.08  144 0.08 0 0.05 0.09 0 0.05 0.08 0 0.06 0.11 0 0.06 0.1 0.02 0.06 0.13 0 0.07  145 0.09 0 0.05 0.1 0 0.05 0.08 0 0.06 0.11 0 0.05 0.11 0 0.05 0.13 0 0.07  146 0.09 0 0.05 0.09 0 0.05 0.08 0 0.06 0.09 0.01 0.05 0.11 0 0.05 0.12 0 0.07  147 0.08 0 0.05 0.1 0 0.05 0.08 0 0.06 0.09 0 0.04 0.12 0 0.05 0.12 0 0.08  148 0.09 0 0.05 0.11 0 0.05 0.08 0 0.06 0.08 0 0.04 0.1 0 0.05 0.11 0 0.08  149 0.09 0 0.06 0.09 0 0.05 0.08 0 0.06 0.08 0 0.03 0.1 0 0.06 0.17 0 0.08  150 0.11 0.01 0.06 0.09 0 0.05 0.07 0 0.04 0.09 0 0.04 0.1 0 0.06 0.13 0 0.09  151 0.12 0.03 0.06 0.09 0 0.04 0.08 0 0.04 0.07 0 0.03 0.1 0 0.04 0.13 0 0.08  152 0.09 0 0.06 0.09 0 0.05 0.08 0 0.04 0.08 0 0.03 0.09 0 0.03 0.12 0 0.08  153 0.1 0 0.06 0.09 0 0.04 0.08 0 0.04 0.12 0 0.03 0.12 0 0.04 0.13 0.03 0.09  154 0.12 0 0.05 0.09 0 0.04 0.08 0 0.04 0.18 0 0.04 0.08 0 0.04 0.12 0.06 0.09  155 0.11 0 0.05 0.11 0 0.04 0.08 0 0.04 0.15 0 0.04 0.08 0 0.03 0.12 0 0.08  156 0.1 0 0.05 0.09 0 0.04 0.08 0 0.04 0.11 0 0.02 0.08 0 0.04 0.13 0 0.07  157 0.09 0 0.06 0.09 0 0.04 0.08 0 0.04 0.19 0 0.03 0.08 0 0.04 0.13 0 0.08  158 0.09 0 0.05 0.09 0 0.04 0.09 0 0.05 0.06 0 0.03 0.08 0 0.04 0.12 0 0.07  159 0.1 0 0.05 0.09 0 0.05 0.08 0.01 0.06 0.08 0 0.04 0.1 0 0.05 0.13 0 0.06  160 0.11 0 0.05 0.09 0 0.05 0.09 0 0.06 0.1 0 0.05 0.11 0 0.05 0.11 0 0.05  161 0.11 0 0.05 0.08 0 0.05 0.08 0 0.04 0.11 0 0.07 0.12 0 0.06 0.11 0.03 0.06  162 0.12 0 0.05 0.09 0 0.05 0.09 0 0.05 0.11 0 0.07 0.11 0 0.06 0.12 0.03 0.07  163 0.12 0 0.05 0.09 0 0.05 0.11 0 0.06 0.11 0 0.07 0.1 0 0.07 0.12 0 0.08  164 0.11 0 0.05 0.09 0.01 0.06 0.09 0 0.05 0.12 0 0.07 0.13 0 0.07 0.12 0 0.08  165 0.1 0.01 0.05 0.1 0.01 0.06 0.1 0 0.05 0.1 0 0.07 0.13 0 0.06 0.13 0.04 0.08  166 0.09 0 0.05 0.09 0.01 0.05 0.1 0 0.05 0.11 0 0.07 0.11 0 0.08 0.12 0.03 0.08  167 0.08 0 0.04 0.1 0 0.05 0.1 0 0.05 0.11 0 0.07 0.12 0.01 0.08 0.12 0 0.09  168 0.09 0 0.04 0.09 0 0.05 0.13 0 0.06 0.12 0 0.07 0.14 0 0.09 0.12 0.04 0.09  169 0.1 0 0.03 0.11 0 0.06 0.1 0 0.06 0.11 0.01 0.07 0.13 0 0.07 0.13 0.04 0.09  170 0.1 0 0.04 0.1 0 0.05 0.1 0 0.06 0.12 0 0.06 0.13 0 0.08 0.13 0.04 0.09  171 0.11 0 0.04 0.1 0 0.05 0.11 0.01 0.06 0.13 0.02 0.07 0.11 0 0.07 0.14 0.04 0.09  172 0.11 0 0.04 0.11 0.02 0.05 0.09 0 0.06 0.12 0 0.07 0.11 0 0.07 0.13 0 0.09  173 0.09 0 0.04 0.1 0.02 0.05 0.09 0 0.06 0.12 0.01 0.07 0.11 0 0.06 0.14 0.03 0.1  174 0.1 0 0.04 0.09 0 0.05 0.1 0 0.06 0.11 0 0.06 0.1 0.01 0.06 0.14 0 0.1  175 0.09 0 0.04 0.1 0 0.05 0.12 0 0.06 0.11 0 0.06 0.1 0 0.06 0.23 0 0.09  176 0.1 0 0.04 0.11 0 0.05 0.11 0 0.06 0.1 0 0.06 0.09 0 0.05 0.14 0 0.09  177 0.1 0 0.04 0.11 0 0.05 0.12 0 0.06 0.1 0 0.07 0.11 0 0.06 0.13 0 0.09  178 0.11 0 0.05 0.1 0 0.05 0.1 0 0.06 0.11 0 0.07 0.11 0 0.06 0.15 0 0.09  179 0.11 0.01 0.05 0.1 0 0.05 0.1 0 0.05 0.09 0 0.06 0.13 0 0.06 0.13 0 0.08  180 0.09 0 0.05 0.09 0 0.05 0.11 0 0.05 0.12 0 0.07 0.11 0 0.06 0.12 0 0.07  181 0.09 0 0.05 0.1 0 0.05 0.11 0 0.05 0.09 0 0.06 0.12 0 0.06 0.11 0.04 0.07  182 0.09 0 0.04 0.09 0 0.05 0.11 0 0.05 0.08 0 0.06 0.09 0 0.06 0.11 0 0.06  183 0.09 0 0.05 0.1 0 0.05 0.12 0 0.05 0.07 0 0.06 0.09 0 0.06 0.12 0 0.06  184 0.09 0 0.05 0.1 0 0.05 0.12 0 0.05 0.07 0 0.06 0.11 0 0.06 0.12 0 0.06  185 0.12 0 0.05 0.12 0 0.05 0.1 0 0.05 0.06 0 0.06 0.1 0 0.06 0.1 0 0.05  186 0.12 0 0.05 0.11 0 0.05 0.1 0 0.05 0.07 0 0.06 0.11 0 0.06 0.12 0 0.05  187 0.09 0 0.05 0.11 0 0.05 0.1 0 0.04 0.09 0 0.06 0.1 0 0.06 0.12 0 0.06  188 0.1 0 0.05 0.1 0 0.05 0.1 0 0.04 0.1 0 0.06 0.1 0 0.06 0.12 0 0.06  189 0.09 0 0.05 0.09 0 0.04 0.11 0 0.05 0.1 0 0.06 0.09 0 0.06 0.11 0 0.06  190 0.09 0 0.05 0.11 0 0.05 0.12 0 0.05 0.09 0 0.06 0.13 0 0.06 0.12 0 0.06  191 0.09 0 0.05 0.09 0 0.04 0.11 0 0.05 0.09 0.01 0.06 0.13 0 0.07 0.13 0 0.06  192 0.1 0 0.05 0.08 0 0.04 0.11 0 0.05 0.1 0 0.06 0.16 0 0.08 0.19 0 0.06  193 0.09 0 0.05 0.09 0 0.04 0.11 0 0.05 0.09 0 0.06 0.13 0 0.07 0.15 0 0.06  194 0.09 0 0.05 0.1 0 0.05 0.11 0 0.05 0.1 0 0.06 0.11 0 0.06 0.12 0 0.06  195 0.1 0 0.06 0.08 0 0.04 0.12 0 0.05 0.13 0 0.06 0.11 0 0.06 0.19 0 0.06  196 0.12 0 0.06 0.1 0 0.05 0.1 0 0.05 0.12 0 0.06 0.12 0 0.05 0.19 0 0.06  197 0.11 0 0.06 0.1 0 0.05 0.12 0 0.05 0.09 0 0.05 0.11 0 0.06 0.19 0 0.06  198 0.1 0 0.05 0.11 0 0.05 0.09 0 0.05 0.09 0 0.06 0.09 0 0.05 0.1 0 0.06  199 0.1 0 0.05 0.09 0 0.05 0.1 0 0.05 0.09 0 0.06 0.08 0 0.05 0.11 0 0.06  200 0.12 0 0.05 0.01 0 0.05 0.1 0 0.04 0.07 0 0.06 0.09 0 0.05 0.1 0 0.07 |
| --- |

| Control (mm) Infected (mm)  Day 6 R1 R2 R3 R1 R2 R3  Max Min Average Max Min Average Max Min Average Max Min Average Max Min Average Max Min Average  1 0.07 0 0.04 0.07 0.01 0.04 0.07 0 0.04 0.13 0 0.06 0.1 0 0.05 0.08 0 0.06  2 0.06 0 0.04 0.08 0 0.04 0.07 0 0.04 0.12 0 0.06 0.14 0 0.06 0.09 0 0.06  3 0.08 0 0.04 0.08 0 0.04 0.07 0 0.04 0.12 0 0.06 0.12 0 0.06 0.1 0 0.06  4 0.06 0 0.04 0.08 0 0.04 0.07 0 0.04 0.11 0 0.06 0.12 0 0.06 0.09 0 0.06  5 0.06 0.01 0.04 0.07 0 0.04 0.07 0 0.04 0.1 0 0.06 0.12 0 0.06 0.09 0 0.06  6 0.07 0 0.05 0.07 0 0.04 0.07 0 0.04 0.11 0 0.06 0.13 0 0.06 0.09 0.02 0.06  7 0.07 0 0.04 0.07 0 0.04 0.07 0 0.04 0.13 0 0.06 0.12 0 0.06 0.09 0 0.06  8 0.07 0 0.04 0.07 0 0.04 0.08 0 0.04 0.13 0 0.06 0.12 0 0.05 0.09 0 0.06  9 0.08 0 0.05 0.06 0 0.04 0.08 0 0.05 0.11 0 0.06 0.12 0 0.06 0.09 0 0.06  10 0.08 0 0.04 0.08 0 0.04 0.09 0 0.04 0.12 0 0.06 0.12 0 0.06 0.08 0 0.06  11 0.08 0 0.05 0.08 0 0.03 0.08 0 0.04 0.12 0 0.07 0.12 0 0.06 0.09 0 0.06  12 0.08 0 0.05 0.08 0 0.04 0.09 0 0.04 0.14 0 0.07 0.12 0 0.07 0.11 0 0.06  13 0.09 0 0.05 0.07 0 0.04 0.09 0 0.04 0.11 0 0.06 0.11 0 0.06 0.1 0 0.06  14 0.09 0 0.05 0.08 0.01 0.04 0.08 0 0.04 0.11 0 0.06 0.13 0 0.07 0.12 0 0.07  15 0.09 0 0.05 0.09 0 0.04 0.08 0 0.04 0.11 0 0.06 0.13 0 0.08 0.12 0 0.07  16 0.09 0 0.05 0.11 0 0.04 0.08 0 0.05 0.11 0 0.06 0.14 0.01 0.08 0.12 0 0.07  17 0.08 0 0.05 0.11 0 0.04 0.09 0 0.05 0.11 0 0.06 0.13 0.01 0.09 0.1 0 0.07  18 0.08 0 0.05 0.11 0 0.04 0.08 0 0.04 0.11 0 0.06 0.12 0 0.09 0.11 0 0.07  19 0.08 0 0.05 0.07 0 0.04 0.09 0 0.04 0.11 0 0.06 0.13 0 0.08 0.1 0 0.07  20 0.08 0 0.05 0.07 0 0.04 0.08 0 0.05 0.11 0 0.06 0.12 0.04 0.08 0.11 0.01 0.07  21 0.08 0 0.05 0.08 0 0.05 0.08 0 0.05 0.11 0 0.06 0.12 0.05 0.08 0.1 0.03 0.07  22 0.09 0 0.04 0.07 0 0.05 0.09 0 0.05 0.11 0 0.06 0.17 0 0.07 0.11 0.04 0.07  23 0.07 0 0.04 0.08 0.02 0.05 0.08 0.03 0.05 0.1 0 0.05 0.13 0 0.07 0.12 0.03 0.07  24 0.09 0 0.05 0.09 0 0.05 0.09 0 0.05 0.12 0 0.05 0.14 0.04 0.08 0.11 0 0.06  25 0.08 0 0.05 0.08 0 0.05 0.09 0 0.05 0.11 0 0.07 0.12 0.04 0.07 0.11 0 0.06  26 0.08 0 0.05 0.09 0.01 0.05 0.1 0 0.05 0.11 0 0.06 0.12 0 0.07 0.1 0 0.06  27 0.08 0 0.05 0.08 0 0.04 0.09 0 0.05 0.12 0 0.06 0.12 0 0.06 0.11 0 0.06  28 0.09 0 0.05 0.08 0 0.05 0.09 0 0.06 0.13 0 0.06 0.11 0 0.07 0.12 0 0.06  29 0.09 0 0.05 0.09 0 0.05 0.09 0.01 0.05 0.12 0 0.06 0.11 0 0.06 0.1 0 0.06  30 0.11 0 0.05 0.08 0 0.05 0.09 0 0.05 0.11 0 0.07 0.12 0 0.06 0.1 0 0.04  31 0.09 0 0.05 0.08 0 0.05 0.08 0.03 0.05 0.11 0 0.06 0.12 0 0.08 0.09 0 0.05  32 0.09 0.01 0.05 0.1 0 0.05 0.08 0 0.05 0.12 0 0.06 0.13 0 0.08 0.11 0 0.04  33 0.09 0 0.06 0.08 0.02 0.05 0.09 0 0.05 0.11 0 0.07 0.12 0 0.08 0.11 0 0.04  34 0.09 0 0.05 0.08 0.02 0.05 0.08 0 0.05 0.12 0.01 0.07 0.13 0 0.08 0.09 0 0.05  35 0.1 0 0.05 0.08 0 0.05 0.08 0 0.05 0.12 0 0.07 0.12 0 0.07 0.08 0.01 0.05  36 0.08 0 0.04 0.09 0.01 0.05 0.08 0 0.05 0.11 0 0.07 0.12 0 0.08 0.12 0 0.05  37 0.08 0 0.04 0.09 0 0.05 0.08 0 0.05 0.11 0.04 0.07 0.11 0.05 0.08 0.12 0 0.05  38 0.08 0 0.04 0.09 0.02 0.05 0.09 0 0.05 0.12 0.03 0.07 0.13 0.05 0.08 0.14 0 0.06  39 0.08 0.02 0.05 0.09 0 0.05 0.09 0 0.05 0.12 0.03 0.07 0.12 0.04 0.08 0.12 0 0.06  40 0.09 0 0.05 0.08 0.01 0.04 0.09 0 0.05 0.11 0 0.07 0.13 0 0.08 0.12 0 0.06  41 0.09 0 0.05 0.08 0.01 0.05 0.08 0 0.05 0.12 0.03 0.07 0.12 0.05 0.09 0.13 0 0.07  42 0.08 0 0.04 0.09 0.01 0.04 0.08 0.01 0.05 0.12 0.03 0.07 0.12 0.05 0.08 0.12 0 0.07  43 0.09 0.02 0.05 0.08 0 0.04 0.07 0.02 0.05 0.12 0.03 0.07 0.12 0.04 0.08 0.13 0 0.07  44 0.09 0 0.05 0.08 0 0.05 0.08 0 0.04 0.11 0.02 0.07 0.12 0.04 0.08 0.16 0 0.07  45 0.09 0 0.04 0.08 0 0.05 0.08 0 0.04 0.14 0.02 0.08 0.12 0.04 0.08 0.14 0.01 0.07  46 0.07 0 0.05 0.08 0.02 0.05 0.09 0 0.04 0.11 0.03 0.07 0.12 0 0.08 0.12 0 0.07  47 0.08 0.03 0.05 0.08 0.03 0.05 0.09 0 0.05 0.12 0.03 0.07 0.12 0 0.08 0.12 0.02 0.07  48 0.07 0 0.04 0.08 0.03 0.05 0.09 0 0.05 0.11 0.03 0.07 0.13 0 0.08 0.12 0.05 0.08  49 0.07 0 0.04 0.09 0.03 0.06 0.09 0.02 0.05 0.11 0 0.07 0.11 0.02 0.07 0.12 0 0.08  50 0.07 0 0.04 0.08 0.03 0.05 0.09 0.02 0.05 0.12 0 0.06 0.11 0 0.07 0.12 0.03 0.08  51 0.07 0 0.05 0.09 0 0.05 0.09 0 0.05 0.1 0 0.06 0.12 0.01 0.07 0.13 0 0.09  52 0.08 0 0.05 0.09 0 0.05 0.11 0 0.06 0.1 0 0.06 0.1 0 0.06 0.12 0 0.09  53 0.07 0 0.05 0.08 0 0.05 0.09 0.03 0.05 0.1 0 0.07 0.11 0 0.06 0.13 0 0.09  54 0.07 0.02 0.05 0.08 0 0.05 0.08 0 0.05 0.1 0 0.06 0.11 0 0.06 0.12 0 0.09  55 0.08 0.02 0.04 0.08 0 0.05 0.08 0 0.05 0.11 0 0.06 0.12 0 0.06 0.12 0 0.08  56 0.08 0 0.05 0.09 0.02 0.05 0.09 0.02 0.05 0.11 0 0.06 0.13 0 0.07 0.14 0 0.09  57 0.09 0.02 0.05 0.08 0 0.05 0.08 0 0.05 0.11 0 0.06 0.11 0 0.06 0.12 0.05 0.08  58 0.1 0 0.05 0.08 0.02 0.05 0.08 0 0.04 0.09 0 0.05 0.11 0 0.06 0.12 0.05 0.08  59 0.09 0 0.05 0.08 0 0.05 0.08 0 0.05 0.1 0 0.05 0.12 0 0.06 0.12 0.04 0.08  60 0.09 0 0.05 0.09 0 0.04 0.09 0.02 0.05 0.1 0 0.05 0.13 0 0.06 0.12 0.03 0.08  61 0.08 0 0.05 0.17 0.02 0.05 0.08 0 0.05 0.09 0 0.05 0.12 0 0.06 0.13 0 0.08  62 0.09 0 0.05 0.09 0.03 0.05 0.08 0 0.05 0.1 0 0.05 0.11 0 0.06 0.12 0 0.08  63 0.09 0 0.06 0.09 0 0.05 0.08 0.03 0.05 0.11 0 0.06 0.13 0 0.06 0.12 0 0.07  64 0.09 0 0.06 0.09 0.01 0.05 0.09 0 0.06 0.11 0 0.06 0.1 0 0.05 0.12 0 0.07  65 0.09 0 0.05 0.09 0 0.04 0.01 0 0.05 0.11 0 0.06 0.12 0 0.06 0.11 0.05 0.07  66 0.08 0 0.05 0.09 0 0.05 0.1 0 0.05 0.11 0 0.06 0.11 0 0.06 0.12 0 0.07  67 0.09 0 0.05 0.08 0 0.04 0.12 0.01 0.06 0.12 0 0.06 0.12 0 0.06 0.12 0 0.07  68 0.1 0 0.05 0.09 0 0.04 0.09 0 0.05 0.12 0 0.07 0.12 0 0.06 0.11 0 0.07  69 0.08 0 0.05 0.08 0 0.04 0.09 0 0.06 0.11 0 0.06 0.11 0 0.06 0.1 0 0.06  70 0.08 0 0.05 0.1 0 0.04 0.08 0 0.05 0.11 0 0.06 0.12 0 0.07 0.12 0 0.06  71 0.08 0 0.04 0.09 0 0.04 0.08 0 0.05 0.12 0.01 0.07 0.12 0 0.07 0.11 0 0.07  72 0.09 0 0.05 0.07 0 0.04 0.08 0 0.05 0.12 0 0.07 0.12 0 0.07 0.13 0 0.08  73 0.08 0 0.04 0.09 0 0.04 0.08 0 0.05 0.12 0 0.07 0.11 0 0.06 0.13 0.02 0.07  74 0.08 0 0.04 0.08 0 0.04 0.09 0 0.05 0.12 0 0.07 0.11 0 0.07 0.12 0.04 0.08  75 0.09 0 0.04 0.09 0 0.04 0.1 0 0.05 0.12 0 0.07 0.12 0 0.08 0.13 0 0.08  76 0.08 0 0.04 0.08 0 0.04 0.08 0 0.04 0.12 0 0.07 0.12 0 0.08 0.13 0.04 0.08  77 0.06 0 0.04 0.09 0 0.04 0.09 0 0.04 0.13 0 0.07 0.13 0 0.08 0.12 0 0.08  78 0.06 0 0.04 0.08 0 0.04 0.09 0 0.05 0.12 0 0.08 0.12 0 0.08 0.13 0 0.08  79 0.07 0 0.04 0.08 0 0.04 0.07 0 0.04 0.12 0 0.08 0.13 0.03 0.08 0.12 0 0.09  80 0.11 0 0.04 0.08 0.01 0.04 0.07 0 0.03 0.12 0 0.08 0.12 0 0.08 0.12 0 0.08  81 0.1 0 0.05 0.08 0 0.04 0.07 0 0.04 0.12 0 0.07 0.13 0.02 0.08 0.13 0 0.08  82 0.09 0 0.05 0.08 0 0.04 0.08 0 0.04 0.11 0 0.07 0.12 0 0.08 0.13 0 0.09  83 0.08 0.02 0.05 0.09 0 0.05 0.11 0 0.04 0.11 0 0.06 0.12 0.04 0.08 0.12 0.07 0.09  84 0.09 0.01 0.05 0.09 0 0.05 0.1 0 0.05 0.11 0 0.06 0.12 0.02 0.08 0.11 0 0.09  85 0.1 0.02 0.05 0.08 0 0.05 0.1 0 0.05 0.11 0 0.06 0.12 0.03 0.08 0.11 0 0.08  86 0.09 0.01 0.04 0.1 0 0.05 0.09 0 0.04 0.11 0 0.06 0.14 0.03 0.08 0.11 0.06 0.08  87 0.09 0 0.05 0.1 0 0.06 0.09 0 0.05 0.11 0.02 0.07 0.14 0.04 0.08 0.13 0 0.08  88 0.08 0 0.04 0.09 0 0.05 0.09 0 0.04 0.11 0 0.07 0.12 0.04 0.08 0.14 0 0.07  89 0.09 0 0.04 0.09 0 0.05 0.09 0 0.05 0.11 0 0.06 0.13 0.03 0.08 0.11 0 0.06  90 0.07 0 0.05 0.08 0 0.04 0.09 0 0.04 0.11 0.03 0.07 0.13 0.05 0.08 0.11 0 0.06  91 0.08 0 0.05 0.08 0 0.04 0.11 0 0.05 0.11 0.03 0.07 0.12 0.04 0.07 0.12 0 0.06  92 0.08 0 0.05 0.08 0 0.05 0.11 0 0.05 0.12 0.03 0.07 0.12 0.03 0.07 0.11 0 0.06  93 0.07 0.01 0.04 0.09 0 0.04 0.1 0 0.05 0.13 0 0.07 0.12 0 0.07 0.11 0 0.06  94 0.06 0.02 0.04 0.09 0 0.04 0.1 0 0.05 0.12 0.4 0.08 0.12 0 0.07 0.12 0 0.05  95 0.08 0 0.04 0.1 0 0.04 0.1 0 0.04 0.12 0.04 0.08 0.13 0 0.07 0.13 0 0.05  96 0.08 0 0.04 0.08 0.01 0.04 0.09 0 0.04 0.13 0 0.08 0.12 0 0.07 0.11 0 0.04  97 0.08 0 0.04 0.09 0 0.04 0.1 0 0.04 0.12 0 0.08 0.11 0 0.06 0.11 0 0.04  98 0.08 0 0.05 0.08 0 0.04 0.09 0 0.04 0.12 0 0.09 0.12 0 0.07 0.11 0 0.05  99 0.08 0.01 0.05 0.08 0 0.04 0.09 0 0.04 0.12 0 0.08 0.14 0 0.07 0.1 0 0.04  100 0.08 0 0.05 0.08 0 0.04 0.1 0 0.04 0.12 0.04 0.08 0.12 0 0.08 0.09 0 0.05  101 0.08 0.01 0.05 0.08 0 0.04 0.11 0 0.04 0.12 0.03 0.08 0.12 0 0.08 0.09 0 0.06  102 0.1 0 0.05 0.09 0 0.04 0.1 0 0.04 0.12 0.04 0.08 0.12 0 0.07 0.12 0.02 0.06  103 0.09 0 0.05 0.09 0.01 0.04 0.1 0 0.04 0.12 0.03 0.08 0.11 0 0.07 0.12 0 0.07  104 0.11 0 0.05 0.09 0 0.04 0.08 0 0.04 0.14 0 0.08 0.12 0 0.07 0.12 0 0.07  105 0.08 0.02 0.04 0.08 0 0.04 0.09 0 0.04 0.14 0.04 0.09 0.12 0 0.08 0.12 0 0.08  106 0.07 0 0.04 0.09 0 0.04 0.08 0.01 0.04 0.15 0.04 0.08 0.13 0.04 0.09 0.12 0 0.08  107 0.09 0 0.05 0.09 0 0.05 0.08 0 0.04 0.14 0.04 0.08 0.15 0 0.09 0.12 0 0.07  108 0.1 0.03 0.05 0.1 0 0.05 0.09 0 0.04 0.15 0 0.08 0.14 0 0.09 0.13 0 0.08  109 0.08 0 0.04 0.09 0 0.05 0.09 0 0.04 0.14 0.03 0.08 0.15 0.03 0.09 0.13 0 0.08  110 0.07 0 0.04 0.09 0 0.04 0.09 0 0.04 0.13 0 0.08 0.14 0 0.09 0.13 0.02 0.08  111 0.07 0 0.04 0.08 0 0.04 0.1 0 0.05 0.12 0.02 0.08 0.14 0 0.09 0.12 0 0.08  112 0.07 0 0.05 0.08 0 0.04 0.1 0 0.05 0.13 0.03 0.08 0.16 0 0.09 0.12 0 0.09  113 0.07 0.02 0.05 0.07 0.01 0.04 0.09 0 0.05 0.12 0 0.07 0.14 0.04 0.09 0.13 0 0.09  114 0.08 0.02 0.04 0.08 0 0.05 0.09 0 0.05 0.13 0 0.07 0.14 0.04 0.09 0.14 0 0.09  115 0.07 0 0.04 0.08 0 0.05 0.1 0 0.05 0.11 0 0.07 0.14 0.05 0.09 0.16 0.06 0.1  116 0.07 0.03 0.05 0.09 0 0.05 0.09 0 0.04 0.11 0 0.07 0.15 0.04 0.09 0.12 0.07 0.1  117 0.08 0 0.05 0.08 0 0.05 0.09 0.01 0.05 0.11 0.03 0.07 0.14 0.04 0.08 0.12 0 0.09  118 0.08 0 0.05 0.09 0 0.05 0.08 0 0.05 0.11 0.01 0.07 0.14 0 0.08 0.12 0 0.08  119 0.07 0 0.05 0.09 0 0.05 0.08 0.01 0.05 0.11 0.03 0.07 0.12 0.04 0.08 0.13 0.04 0.09  120 0.07 0 0.05 0.08 0 0.05 0.08 0 0.05 0.1 0.03 0.07 0.12 0.03 0.07 0.12 0 0.08  121 0.08 0 0.05 0.08 0 0.05 0.08 0 0.05 0.1 0 0.06 0.15 0.03 0.08 0.12 0 0.07  122 0.08 0 0.05 0.08 0 0.05 0.08 0 0.05 0.11 0 0.06 0.14 0 0.07 0.13 0.04 0.08  123 0.07 0 0.04 0.1 0 0.05 0.09 0.02 0.05 0.12 0 0.07 0.13 0.04 0.07 0.12 0.04 0.07  124 0.07 0.01 0.05 0.08 0 0.05 0.08 0.03 0.03 0.12 0.03 0.07 0.13 0.03 0.07 0.11 0 0.07  125 0.07 0 0.05 0.11 0.01 0.05 0.09 0 0.06 0.14 0.03 0.07 0.15 0.03 0.08 0.12 0 0.07  126 0.08 0 0.05 0.1 0 0.05 0.09 0 0.06 0.14 0.02 0.08 0.16 0 0.08 0.14 0.05 0.07  127 0.08 0 0.05 0.1 0 0.05 0.09 0 0.06 0.13 0 0.08 0.15 0 0.08 0.14 0.05 0.08  128 0.08 0 0.05 0.12 0 0.05 0.09 0.01 0.06 0.13 0 0.08 0.17 0 0.08 0.14 0.05 0.08  129 0.08 0 0.05 0.11 0.02 0.05 0.09 0.01 0.06 0.12 0.01 0.08 0.13 0 0.08 0.14 0 0.07  130 0.08 0 0.05 0.08 0 0.04 0.09 0 0.06 0.13 0.03 0.08 0.13 0 0.08 0.13 0 0.07  131 0.09 0 0.05 0.11 0 0.05 0.09 0.01 0.06 0.13 0.02 0.08 0.13 0.04 0.09 0.12 0 0.07  132 0.09 0 0.05 0.08 0 0.05 0.08 0 0.05 0.13 0.02 0.07 0.12 0 0.08 0.11 0.04 0.08  133 0.09 0 0.05 0.08 0 0.04 0.09 0 0.05 0.14 0 0.08 0.13 0 0.08 0.15 0 0.08  134 0.08 0 0.05 0.08 0 0.05 0.1 0.01 0.05 0.13 0.01 0.08 0.12 0 0.08 0.12 0 0.08  135 0.09 0 0.06 0.08 0 0.05 0.09 0.02 0.05 0.12 0 0.08 0.12 0.02 0.08 0.14 0 0.08  136 0.08 0 0.05 0.09 0.03 0.05 0.09 0 0.05 0.12 0 0.08 0.12 0.01 0.08 0.13 0 0.09  137 0.09 0 0.05 0.11 0 0.05 0.1 0 0.05 0.12 0 0.08 0.12 0 0.09 0.13 0 0.08  138 0.08 0 0.06 0.08 0 0.05 0.09 0.03 0.06 0.12 0 0.08 0.13 0 0.09 0.17 0.03 0.09  139 0.08 0 0.05 0.09 0.03 0.06 0.08 0 0.05 0.12 0 0.09 0.14 0 0.08 0.13 0 0.08  140 0.09 0 0.05 0.08 0 0.05 0.09 0.03 0.06 0.12 0 0.09 0.12 0 0.09 0.12 0.05 0.08  141 0.09 0 0.05 0.09 0.02 0.06 0.09 0 0.04 0.12 0 0.08 0.12 0 0.09 0.14 0 0.08  142 0.09 0 0.05 0.08 0 0.06 0.11 0 0.06 0.12 0 0.08 0.14 0.06 0.09 0.13 0.01 0.09  143 0.09 0.02 0.05 0.08 0 0.05 0.1 0 0.06 0.12 0.04 0.08 0.12 0.06 0.09 0.12 0 0.09  144 0.09 0 0.05 0.09 0 0.05 0.1 0 0.06 0.11 0.05 0.08 0.12 0.05 0.09 0.13 0 0.1  145 0.08 0 0.05 0.09 0 0.06 0.09 0 0.06 0.12 0 0.08 0.13 0.06 0.08 0.12 0 0.09  146 0.09 0 0.05 0.11 0 0.06 0.1 0 0.06 0.12 0 0.08 0.12 0.02 0.08 0.13 0 0.09  147 0.09 0 0.06 0.08 0 0.06 0.1 0.02 0.06 0.12 0 0.07 0.12 0 0.08 0.14 0 0.09  148 0.09 0 0.05 0.11 0 0.06 0.1 0 0.06 0.12 0.05 0.09 0.12 0.04 0.08 0.13 0 0.08  149 0.08 0.02 0.05 0.1 0 0.06 0.11 0 0.06 0.14 0.04 0.08 0.11 0 0.08 0.13 0 0.08  150 0.09 0.02 0.05 0.09 0 0.06 0.09 0 0.06 0.13 0.04 0.08 0.13 0.05 0.08 0.15 0 0.08  151 0.09 0 0.05 0.1 0.03 0.06 0.11 0 0.06 0.12 0 0.08 0.13 0 0.07 0.13 0 0.08  152 0.09 0 0.05 0.09 0.03 0.06 0.11 0 0.06 0.13 0.04 0.09 0.13 0 0.08 0.16 0 0.08  153 0.09 0.03 0.06 0.09 0 0.05 0.09 0 0.06 0.13 0.04 0.09 0.13 0 0.08 0.15 0.04 0.08  154 0.09 0 0.05 0.09 0 0.06 0.1 0.02 0.06 0.12 0.04 0.09 0.14 0 0.09 0.16 0.03 0.08  155 0.08 0.02 0.05 0.11 0 0.06 0.09 0 0.05 0.13 0.01 0.09 0.13 0.04 0.09 0.15 0 0.07  156 0.08 0 0.05 0.09 0.02 0.06 0.1 0 0.05 0.14 0.03 0.09 0.13 0 0.09 0.15 0 0.07  157 0.09 0.01 0.05 0.1 0 0.06 0.1 0 0.05 0.14 0.03 0.09 0.13 0 0.09 0.13 0.03 0.07  158 0.09 0 0.05 0.11 0 0.05 0.09 0.03 0.06 0.14 0.05 0.09 0.13 0 0.1 0.13 0 0.07  159 0.09 0.02 0.05 0.1 0 0.05 0.08 0.03 0.05 0.13 0 0.08 0.13 0.04 0.09 0.12 0 0.07  160 0.1 0 0.06 0.1 0.02 0.05 0.1 0.01 0.06 0.13 0.05 0.09 0.13 0 0.09 0.11 0 0.07  161 0.1 0 0.05 0.1 0 0.05 0.09 0 0.04 0.13 0.01 0.09 0.12 0 0.09 0.12 0.04 0.07  162 0.1 0 0.06 0.1 0.02 0.05 0.1 0 0.04 0.14 0.04 0.09 0.13 0.03 0.1 0.14 0 0.08  163 0.1 0 0.05 0.09 0.03 0.05 0.1 0.01 0.05 0.13 0 0.09 0.15 0 0.1 0.12 0 0.08  164 0.09 0 0.05 0.09 0 0.05 0.09 0 0.04 0.14 0 0.09 0.13 0 0.09 0.12 0 0.08  165 0.1 0 0.05 0.11 0 0.05 0.08 0 0.04 0.13 0 0.08 0.14 0 0.09 0.14 0 0.08  166 0.09 0.03 0.05 0.11 0 0.05 0.08 0.02 0.05 0.13 0 0.08 0.14 0 0.09 0.12 0 0.08  167 0.09 0 0.05 0.11 0.02 0.05 0.08 0.01 0.05 0.13 0 0.07 0.14 0.02 0.08 0.11 0 0.07  168 0.09 0.02 0.05 0.11 0 0.05 0.1 0 0.05 0.13 0 0.08 0.15 0 0.08 0.15 0 0.09  169 0.09 0.03 0.05 0.08 0 0.05 0.08 0 0.05 0.12 0.03 0.07 0.13 0 0.08 0.13 0.03 0.09  170 0.09 0 0.05 0.09 0 0.05 0.08 0 0.05 0.12 0 0.07 0.13 0 0.08 0.13 0 0.09  171 0.1 0 0.06 0.08 0.03 0.06 0.09 0 0.05 0.13 0 0.07 0.13 0 0.07 0.13 0 0.09  172 0.1 0 0.06 0.09 0 0.05 0.09 0 0.06 0.13 0 0.07 0.14 0 0.08 0.14 0 0.09  173 0.11 0.02 0.06 0.1 0.03 0.06 0.09 0.03 0.05 0.13 0 0.07 0.13 0 0.07 0.14 0 0.09  174 0.1 0 0.06 0.08 0 0.06 0.09 0.02 0.06 0.13 0 0.07 0.12 0 0.07 0.12 0 0.1  175 0.09 0.02 0.06 0.09 0 0.06 0.09 0 0.05 0.12 0 0.07 0.14 0 0.08 0.12 0 0.1  176 0.07 0 0.05 0.09 0 0.06 0.09 0 0.06 0.12 0 0.06 0.13 0 0.08 0.14 0.04 0.1  177 0.08 0 0.05 0.1 0 0.05 0.09 0.02 0.06 0.11 0 0.06 0.13 0.02 0.08 0.12 0 0.09  178 0.08 0.01 0.05 0.11 0 0.05 0.1 0.02 0.05 0.11 0 0.05 0.12 0 0.06 0.12 0 0.09  179 0.08 0 0.06 0.09 0 0.05 0.11 0 0.06 0.12 0 0.05 0.12 0 0.06 0.14 0.05 0.09  180 0.08 0.01 0.05 0.1 0 0.06 0.1 0 0.06 0.13 0 0.05 0.15 0 0.06 0.12 0 0.08  181 0.1 0.02 0.05 0.1 0 0.05 0.11 0.02 0.06 0.15 0 0.06 0.15 0 0.07 0.13 0 0.08  182 0.08 0.01 0.05 0.1 0 0.05 0.1 0 0.06 0.14 0 0.06 0.13 0 0.07 0.16 0 0.09  183 0.08 0 0.05 0.1 0 0.05 0.12 0 0.06 0.16 0 0.06 0.13 0 0.07 0.15 0.05 0.09  184 0.09 0 0.05 0.1 0 0.05 0.12 0 0.06 0.14 0 0.06 0.11 0 0.05 0.13 0 0.08  185 0.09 0 0.05 0.09 0 0.05 0.13 0 0.06 0.15 0 0.06 0.11 0 0.05 0.12 0 0.08  186 0.1 0 0.04 0.12 0 0.05 0.11 0 0.05 0.16 0 0.07 0.12 0 0.06 0.12 0 0.08  187 0.09 0 0.04 0.1 0 0.05 0.1 0 0.05 0.13 0 0.07 0.13 0 0.06 0.14 0 0.09  188 0.09 0 0.04 0.1 0 0.05 0.12 0 0.05 0.15 0 0.08 0.12 0 0.07 0.14 0 0.08  189 0.09 0 0.04 0.1 0 0.05 0.1 0 0.05 0.14 0 0.06 0.12 0 0.06 0.13 0 0.08  190 0.09 0 0.05 0.1 0 0.05 0.12 0 0.05 0.12 0 0.08 0.11 0 0.06 0.14 0 0.09  191 0.08 0 0.05 0.11 0 0.05 0.12 0 0.05 0.12 0 0.08 0.11 0 0.07 0.12 0 0.09  192 0.08 0 0.04 0.11 0 0.05 0.11 0 0.04 0.12 0 0.07 0.12 0.01 0.07 0.13 0 0.08  193 0.07 0 0.05 0.1 0 0.05 0.1 0 0.05 0.13 0 0.08 0.15 0 0.07 0.13 0 0.08  194 0.08 0 0.04 0.1 0 0.05 0.1 0 0.04 0.13 0 0.09 0.13 0 0.09 0.14 0 0.09  195 0.08 0 0.04 0.1 0.01 0.05 0.11 0 0.05 0.12 0 0.08 0.13 0 0.08 0.15 0 0.09  196 0.1 0 0.05 0.11 0 0.05 0.1 0 0.05 0.12 0 0.08 0.13 0.02 0.09 0.17 0 0.09  197 0.08 0 0.04 0.1 0 0.05 0.12 0 0.05 0.12 0 0.09 0.15 0 0.09 0.16 0 0.1  198 0.08 0 0.05 0.13 0 0.05 0.1 0 0.05 0.12 0 0.08 0.13 0 0.09 0.13 0 0.08  199 0.08 0.02 0.05 0.12 0 0.05 0.09 0.02 0.05 0.12 0.01 0.08 0.14 0 0.08 0.13 0 0.09  200 0.07 0 0.04 0.12 0 0.05 0.1 0 0.05 0.13 0 0.08 0.12 0 0.08 0.14 0 0.09 |
| --- |

| Control (mm) Infected (mm)  Day 7 R1 R2 R3 R1 R2 R3  Max Min Average Max Min Average Max Min Average Max Min Average Max Min Average Max Min Average  1 0.07 0 0.03 0.02 0 0.01 0.05 0 0.02 0.13 0 0.06 0.12 0 0.07 0.23 0 0.08  2 0.06 0 0.03 0.02 0 0.01 0.04 0 0.02 0.13 0 0.07 0.12 0 0.07 0.11 0 0.07  3 0.06 0 0.03 0.03 0 0.02 0.04 0 0.02 0.13 0.01 0.07 0.13 0 0.07 0.39 0 0.07  4 0.06 0 0.03 0.02 0.01 0.01 0.05 0 0.02 0.12 0 0.07 0.12 0 0.06 0.12 0 0.07  5 0.06 0 0.03 0.02 0 0.01 0.04 0 0.02 0.12 0 0.07 0.15 0 0.07 0.14 0 0.07  6 0.05 0 0.02 0.02 0 0.01 0.05 0 0.02 0.14 0.01 0.07 0.16 0 0.05 0.12 0 0.07  7 0.05 0 0.02 0.02 0 0.01 0.03 0 0.01 0.11 0 0.07 0.14 0 0.07 0.11 0 0.07  8 0.04 0 0.01 0.02 0 0.02 0.03 0 0.01 0.12 0 0.07 0.13 0 0.06 0.33 0 0.1  9 0.04 0 0.01 0.02 0 0.01 0.04 0 0.02 0.12 0 0.08 0.11 0 0.04 0.11 0 0.07  10 0.04 0 0.02 0.03 0 0.02 0.05 0 0.02 0.11 0 0.07 0.1 0 0.05 0.11 0 0.07  11 0.05 0 0.03 0.04 0 0.02 0.04 0 0.02 0.11 0 0.08 0.11 0 0.06 0.12 0 0.07  12 0.06 0 0.02 0.04 0 0.02 0.04 0 0.02 0.12 0 0.08 0.1 0 0.05 0.12 0.01 0.07  13 0.04 0 0.03 0.03 0 0.01 0.15 0 0.02 0.11 0 0.07 0.1 0 0.04 0.12 0 0.07  14 0.06 0 0.03 0.03 0 0.01 0.03 0 0.02 0.12 0 0.07 0.12 0 0.06 0.11 0.03 0.07  15 0.06 0 0.03 0.04 0 0.02 0.02 0 0.01 0.1 0 0.07 0.11 0 0.06 0.11 0 0.07  16 0.06 0 0.03 0.05 0 0.03 0.15 0 0.03 0.11 0 0.06 0.12 0 0.07 0.11 0 0.07  17 0.06 0 0.03 0.04 0 0.02 0.03 0 0.01 0.1 0 0.06 0.11 0 0.06 0.11 0 0.07  18 0.06 0 0.03 0.03 0 0.01 0.04 0 0.02 0.11 0.01 0.06 0.12 0 0.07 0.11 0.01 0.06  19 0.06 0 0.03 0.04 0 0.02 0.05 0 0.02 0.11 0 0.07 0.14 0 0.08 0.11 0 0.06  20 0.02 0 0.04 0.03 0 0.02 0.04 0 0.02 0.11 0 0.07 0.13 0 0.08 0.1 0 0.06  21 0.06 0 0.03 0.04 0 0.02 0.05 0 0.02 0.12 0 0.06 0.12 0 0.08 0.11 0 0.06  22 0.06 0 0.03 0.04 0 0.02 0.04 0 0.02 0.11 0 0.05 0.13 0 0.08 0.1 0 0.06  23 0.08 0 0.03 0.04 0 0.02 0.04 0 0.02 0.12 0 0.06 0.13 0 0.08 0.12 0 0.06  24 0.07 0 0.05 0.04 0 0.02 0.04 0 0.01 0.11 0 0.06 0.19 0 0.08 0.11 0 0.06  25 0.07 0 0.05 0.05 0 0.03 0.04 0 0.02 0.2 0 0.07 0.15 0 0.08 0.11 0 0.06  26 0.07 0 0.06 0.05 0 0.03 0.02 0 0.01 0.18 0 0.06 0.15 0 0.09 0.11 0 0.06  27 0.08 0 0.06 0.04 0 0.02 0.05 0 0.02 0.12 0 0.06 0.14 0.02 0.09 0.11 0 0.06  28 0.07 0.01 0.06 0.05 0 0.02 0.06 0 0.03 0.14 0 0.06 0.15 0 0.09 0.12 0 0.06  29 0.07 0 0.06 0.03 0 0.02 0.07 0 0.05 0.12 0 0.06 0.15 0.01 0.09 0.16 0 0.06  30 0.06 0 0.06 0.03 0 0.01 0.06 0 0.04 0.14 0 0.06 0.18 0 0.1 0.13 0 0.06  31 0.06 0 0.06 0.04 0 0.01 0.07 0 0.04 0.14 0 0.06 0.17 0 0.1 0.19 0 0.07  32 0.06 0 0.02 0.04 0 0.02 0.06 0 0.03 0.14 0 0.07 0.15 0 0.1 0.13 0 0.07  33 0.06 0 0.03 0.06 0 0.02 0 0 0.03 0.12 0 0.06 0.17 0.05 0.1 0.21 0 0.07  34 0.06 0 0.03 0.04 0 0.02 0.05 0 0.03 0.13 0 0.06 0.16 0.05 0.09 0.27 0 0.08  35 0.06 0 0.03 0.05 0 0.02 0.06 0.01 0.03 0.12 0 0.06 0.15 0.04 0.09 0.14 0 0.07  36 0.07 0 0.04 0.06 0 0.03 0.07 0 0.03 0.12 0 0.06 0.16 0 0.09 0.13 0 0.07  37 0.06 0 0.03 0.06 0 0.03 0.07 0 0.04 0.12 0 0.07 0.18 0.05 0.09 0.12 0 0.07  38 0.08 0 0.03 0.06 0 0.02 0.07 0 0.03 0.11 0 0.07 0.14 0.04 0.08 0.16 0 0.07  39 0.1 0 0.04 0.07 0 0.03 0.05 0 0.03 0.13 0 0.07 0.17 0 0.08 0.12 0 0.07  40 0.1 0 0.03 0.07 0 0.03 0.05 0 0.02 0.13 0 0.07 0.15 0 0.07 0.13 0 0.07  41 0.08 0 0.03 0.07 0 0.03 0.04 0 0.02 0.12 0 0.08 0.13 0.04 0.07 0.13 0 0.07  42 0.04 0 0.03 0.07 0 0.03 0.04 0 0.02 0.12 0 0.08 0.13 0 0.06 0.12 0 0.07  43 0.05 0 0.03 0.06 0 0.03 0.06 0 0.04 0.12 0 0.08 0.13 0 0.07 0.12 0 0.07  44 0.04 0 0.02 0.07 0 0.04 0.07 0 0.03 0.12 0 0.07 0.16 0 0.06 0.12 0 0.07  45 0.04 0 0.02 0.07 0 0.04 0.06 0 0.03 0.11 0 0.07 0.13 0.01 0.06 0.11 0 0.07  46 0.04 0 0.01 0.07 0.02 0.04 0.06 0 0.03 0.11 0 0.07 0.12 0.01 0.06 0.11 0 0.07  47 0.05 0 0.02 0.06 0 0.04 0.05 0 0.03 0.12 0 0.07 0.1 0 0.06 0.11 0.02 0.07  48 0.05 0 0.02 0.06 0 0.03 0.05 0 0.02 0.12 0 0.07 0.09 0 0.06 0.12 0 0.06  49 0.05 0 0.02 0.05 0.02 0.04 0.05 0 0.02 0.11 0 0.07 0.12 0 0.06 0.13 0 0.06  50 0.06 0 0.04 0.05 0 0.03 0.03 0 0.01 0.11 0 0.07 0.11 0 0.06 0.12 0 0.07  51 0.08 0 0.04 0.06 0 0.03 0.03 0 0.02 0.11 0 0.07 0.14 0 0.07 0.11 0 0.06  52 0.09 0 0.03 0.05 0 0.02 0.04 0 0.02 0.12 0 0.06 0.13 0 0.06 0.12 0 0.07  53 0.08 0 0.03 0.06 0 0.02 0.04 0 0.02 0.11 0 0.06 0.12 0 0.07 0.12 0 0.07  54 0.07 0 0.03 0.06 0 0.02 0.04 0 0.02 0.11 0 0.07 0.12 0 0.07 0.11 0 0.06  55 0.06 0 0.03 0.06 0 0.02 0.04 0 0.02 0.12 0 0.07 0.13 0 0.07 0.12 0 0.07  56 0.06 0 0.03 0.06 0 0.03 0.04 0 0.02 0.12 0 0.07 0.12 0 0.08 0.12 0 0.07  57 0.08 0 0.03 0.05 0 0.02 0.04 0 0.01 0.12 0 0.08 0.13 0.03 0.08 0.11 0 0.07  58 0.06 0 0.03 0.05 0 0.03 0.03 0 0.01 0.12 0 0.07 0.12 0 0.08 0.12 0 0.06  59 0.08 0 0.04 0.06 0 0.03 0.04 0 0.02 0.11 0 0.07 0.14 0 0.09 0.14 0 0.07  60 0.06 0 0.04 0.06 0 0.04 0.05 0 0.02 0.12 0 0.06 0.14 0 0.08 0.12 0 0.07  61 0.07 0 0.03 0.06 0 0.04 0.07 0 0.04 0.12 0 0.07 0.13 0.05 0.09 0.13 0 0.07  62 0.08 0 0.04 0.05 0 0.03 0.07 0 0.04 0.12 0 0.07 0.12 0 0.08 0.13 0 0.07  63 0.06 0 0.04 0.05 0 0.03 0.08 0.01 0.05 0.12 0 0.07 0.13 0 0.08 0.12 0 0.07  64 0.07 0 0.04 0.05 0 0.02 0.07 0 0.04 0.12 0 0.07 0.12 0 0.09 0.12 0 0.07  65 0.05 0 0.03 0.05 0 0.02 0.07 0.02 0.04 0.11 0 0.07 0.12 0.04 0.09 0.12 0.02 0.07  66 0.05 0 0.03 0.06 0 0.04 0.07 0 0.04 0.12 0 0.07 0.12 0.05 0.09 0.12 0 0.07  67 0.06 0 0.03 0.07 0 0.03 0.06 0 0.03 0.12 0 0.07 0.14 0 0.08 0.12 0 0.07  68 0.06 0 0.03 0.07 0 0.03 0.07 0 0.03 0.12 0 0.07 0.13 0.04 0.09 0.13 0 0.07  69 0.06 0 0.03 0.07 0 0.03 0.05 0 0.03 0.13 0 0.08 0.13 0 0.09 0.12 0 0.07  70 0.05 0 0.02 0.08 0 0.03 0.07 0 0.03 0.11 0 0.08 0.14 0.03 0.09 0.12 0 0.08  71 0.07 0 0.03 0.08 0 0.03 0.06 0 0.03 0.11 0 0.07 0.16 0.04 0.08 0.12 0 0.07  72 0.06 0 0.03 0.07 0 0.04 0.05 0 0.03 0.11 0 0.07 0.14 0 0.08 0.13 0 0.07  73 0.06 0 0.03 0.06 0 0.03 0.05 0 0.02 0.12 0 0.07 0.13 0 0.07 0.11 0 0.07  74 0.05 0 0.02 0.06 0 0.03 0.05 0 0.02 0.13 0 0.08 0.12 0 0.08 0.14 0 0.07  75 0.08 0 0.03 0.05 0 0.03 0.06 0 0.02 0.12 0 0.07 0.12 0 0.07 0.12 0 0.07  76 0.07 0 0.03 0.06 0 0.03 0.05 0 0.02 0.12 0 0.07 0.14 0 0.07 0.14 0.01 0.07  77 0.07 0 0.03 0.07 0 0.04 0.04 0 0.02 0.12 0 0.07 0.11 0 0.06 0.14 0 0.07  78 0.06 0 0.03 0.07 0 0.03 0.04 0 0.02 0.13 0 0.07 0.12 0 0.05 0.13 0 0.07  79 0.07 0 0.02 0.06 0 0.03 0.04 0 0.02 0.12 0 0.07 0.11 0 0.06 0.12 0 0.07  80 0.07 0 0.03 0.07 0 0.04 0.03 0 0.01 0.12 0 0.07 0.12 0 0.06 0.12 0 0.07  81 0.06 0 0.03 0.06 0 0.03 0.02 0 0.01 0.12 0 0.07 0.08 0 0.06 0.12 0 0.07  82 0.06 0 0.03 0.06 0 0.03 0.02 0 0.01 0.14 0 0.07 0.1 0 0.06 0.13 0 0.07  83 0.07 0 0.04 0.05 0 0.02 0.01 0.01 0.01 0.12 0 0.07 0.13 0 0.06 0.12 0 0.07  84 0.07 0 0.03 0.04 0 0.02 0.04 0 0.02 0.12 0 0.07 0.11 0.02 0.06 0.13 0 0.07  85 0.06 0 0.03 0.04 0 0.03 0.02 0 0.01 0.12 0 0.06 0.1 0 0.06 0.14 0 0.07  86 0.05 0 0.03 0.04 0 0.02 0.04 0 0.02 0.12 0.03 0.07 0.11 0 0.07 0.14 0 0.07  87 0.06 0 0.03 0.04 0 0.02 0.04 0 0.02 0.14 0 0.07 0.11 0 0.07 0.12 0 0.07  88 0.06 0 0.03 0.03 0 0.02 0.05 0 0.03 0.13 0 0.06 0.12 0 0.07 0.13 0 0.07  89 0.07 0 0.03 0.03 0 0.01 0.05 0 0.03 0.12 0 0.06 0.12 0 0.07 0.12 0 0.07  90 0.06 0 0.03 0.03 0 0.01 0.05 0 0.02 0.12 0 0.06 0.12 0 0.07 0.13 0 0.07  91 0.05 0 0.03 0.04 0 0.02 0.05 0 0.02 0.14 0 0.07 0.12 0 0.07 0.12 0 0.07  92 0.06 0 0.03 0.04 0 0.02 0.03 0 0.01 0.13 0 0.07 0.12 0 0.08 0.2 0.03 0.08  93 0.07 0 0.03 0.04 0 0.02 0.05 0 0.02 0.12 0 0.07 0.12 0 0.08 0.19 0 0.07  94 0.07 0 0.03 0.05 0 0.02 0.06 0 0.03 0.12 0 0.07 0.13 0.04 0.08 0.12 0 0.07  95 0.06 0 0.03 0.05 0 0.02 0.06 0 0.03 0.12 0 0.07 0.13 0 0.09 0.12 0 0.07  96 0.05 0 0.02 0.05 0 0.02 0.06 0 0.04 0.12 0.03 0.07 0.13 0 0.09 0.12 0 0.06  97 0.05 0 0.03 0.08 0 0.03 0.05 0 0.03 0.12 0.04 0.08 0.14 0 0.09 0.12 0 0.07  98 0.06 0 0.03 0.06 0 0.04 0.06 0 0.03 0.12 0 0.07 0.13 0.04 0.09 0.11 0.01 0.07  99 0.07 0 0.03 0.07 0 0.03 0.05 0 0.02 0.11 0 0.07 0.13 0 0.09 0.12 0 0.08  100 0.06 0 0.03 0.06 0 0.03 0.05 0 0.02 0.12 0 0.08 0.14 0 0.09 0.12 0 0.07  101 0.08 0 0.03 0.07 0 0.02 0.06 0 0.03 0.12 0 0.07 0.13 0.04 0.09 0.12 0 0.07  102 0.09 0 0.04 0.08 0 0.03 0.07 0 0.04 0.12 0.03 0.07 0.12 0 0.09 0.12 0 0.08  103 0.08 0 0.04 0.07 0 0.03 0.07 0 0.04 0.2 0 0.08 0.13 0.06 0.09 0.12 0 0.07  104 0.07 0 0.04 0.08 0 0.03 0.06 0 0.03 0.14 0.04 0.08 0.12 0.05 0.08 0.11 0 0.08  105 0.07 0 0.04 0.06 0 0.03 0.06 0 0.03 0.24 0 0.09 0.12 0 0.09 0.12 0.03 0.08  106 0.07 0 0.04 0.05 0 0.03 0.06 0 0.03 0.23 0 0.08 0.13 0.04 0.08 0.12 0 0.07  107 0.07 0 0.04 0.06 0 0.04 0.06 0 0.03 0.26 0 0.08 0.12 0 0.08 0.11 0 0.08  108 0.08 0 0.05 0.06 0 0.04 0.05 0 0.03 0.11 0 0.07 0.12 0.01 0.08 0.2 0 0.08  109 0.08 0 0.05 0.06 0 0.03 0.06 0 0.03 0.12 0 0.07 0.13 0 0.07 0.16 0 0.08  110 0.09 0.02 0.05 0.06 0 0.03 0.04 0 0.02 0.13 0 0.08 0.12 0 0.07 0.21 0 0.08  111 0.09 0 0.05 0.06 0 0.03 0.06 0 0.02 0.11 0 0.07 0.12 0 0.06 0.21 0 0.08  112 0.09 0 0.05 0.06 0 0.04 0.06 0 0.03 0.12 0 0.07 0.12 0.03 0.06 0.22 0 0.08  113 0.09 0 0.05 0.06 0 0.04 0.05 0 0.02 0.11 0 0.06 0.13 0 0.06 0.16 0 0.08  114 0.08 0 0.05 0.06 0 0.04 0.04 0 0.02 0.12 0 0.06 0.12 0 0.06 0.11 0 0.06  115 0.1 0 0.05 0.05 0 0.03 0.04 0 0.01 0.12 0 0.07 0.12 0 0.06 0.11 0 0.07  116 0.08 0 0.05 0.06 0 0.03 0.05 0 0.03 0.12 0 0.07 0.1 0 0.06 0.12 0 0.07  117 0.07 0 0.06 0.05 0 0.03 0.06 0 0.04 0.11 0 0.07 0.12 0 0.06 0.12 0 0.07  118 0.08 0 0.06 0.04 0 0.02 0.06 0 0.03 0.12 0 0.08 0.1 0 0.07 0.12 0 0.08  119 0.08 0 0.06 0.06 0 0.01 0.06 0 0.03 0.11 0 0.07 0.15 0 0.07 0.12 0 0.08  120 0.08 0.01 0.06 0.05 0 0.03 0.06 0 0.03 0.11 0.04 0.07 0.15 0 0.08 0.12 0 0.08  121 0.07 0.01 0.06 0.05 0 0.03 0.06 0 0.03 0.12 0 0.07 0.14 0 0.08 0.13 0 0.08  122 0.09 0 0.06 0.05 0 0.03 0.06 0 0.04 0.12 0 0.08 0.14 0 0.09 0.12 0 0.08  123 0.08 0 0.06 0.06 0 0.03 0.07 0 0.04 0.11 0 0.08 0.13 0 0.08 0.12 0 0.08  124 0.06 0 0.06 0.06 0 0.03 0.07 0 0.04 0.27 0 0.1 0.14 0 0.09 0.15 0 0.08  125 0.08 0 0.06 0.06 0 0.03 0.06 0 0.04 0.12 0 0.08 0.15 0 0.09 0.13 0 0.08  126 0.08 0 0.06 0.06 0 0.03 0.07 0 0.04 0.12 0 0.08 0.14 0 0.09 0.14 0 0.08  127 0.08 0 0.06 0.05 0 0.03 0.07 0 0.04 0.12 0.02 0.08 0.14 0 0.1 0.12 0 0.08  128 0.09 0 0.04 0.06 0 0.03 0.09 0 0.05 0.12 0 0.08 0.14 0 0.09 0.12 0 0.08  129 0.07 0 0.04 0.07 0 0.03 0.08 0.03 0.05 0.13 0 0.08 0.15 0 0.1 0.12 0 0.08  130 0.08 0 0.04 0.06 0 0.03 0.07 0.02 0.05 0.13 0 0.08 0.17 0 0.09 0.12 0 0.08  131 0.08 0 0.04 0.06 0 0.04 0.07 0 0.04 0.12 0 0.08 0.15 0 0.1 0.12 0 0.08  132 0.08 0 0.04 0.06 0 0.03 0.07 0.01 0.04 0.14 0 0.08 0.15 0 0.1 0.12 0.02 0.08  133 0.08 0 0.04 0.07 0 0.03 0.07 0 0.04 0.11 0 0.08 0.14 0 0.1 0.12 0 0.08  134 0.08 0 0.04 0.07 0 0.03 0.07 0 0.03 0.12 0 0.08 0.15 0.06 0.1 0.12 0.01 0.08  135 0.08 0 0.04 0.07 0 0.03 0.07 0 0.03 0.13 0 0.07 0.17 0 0.1 0.12 0 0.08  136 0.06 0 0.03 0.06 0 0.04 0.06 0 0.03 0.13 0 0.07 0.16 0.02 0.1 0.12 0 0.07  137 0.07 0 0.03 0.06 0 0.04 0.06 0 0.02 0.15 0 0.08 0.16 0 0.1 0.12 0.01 0.08  138 0.08 0 0.04 0.07 0 0.03 0.05 0 0.02 0.11 0 0.07 0.15 0 0.09 0.32 0 0.09  139 0.08 0 0.05 0.06 0 0.03 0.05 0 0.02 0.15 0 0.07 0.14 0 0.09 0.13 0 0.07  140 0.08 0 0.04 0.06 0 0.03 0.05 0 0.02 0.12 0 0.07 0.15 0 0.09 0.13 0 0.07  141 0.08 0 0.04 0.06 0 0.03 0.06 0 0.03 0.13 0 0.07 0.15 0 0.08 0.26 0.01 0.08  142 0.08 0 0.04 0.06 0 0.03 0.06 0 0.03 0.13 0 0.07 0.16 0 0.08 0.13 0.02 0.07  143 0.07 0 0.04 0.06 0 0.03 0.06 0 0.04 0.13 0 0.06 0.17 0 0.08 0.11 0 0.07  144 0.06 0 0.04 0.05 0 0.03 0.05 0 0.03 0.14 0 0.07 0.1 0.01 0.08 0.11 0 0.06  145 0.07 0 0.04 0.06 0 0.03 0.05 0 0.03 0.13 0 0.06 0.17 0.01 0.08 0.12 0 0.07  146 0.07 0 0.04 0.05 0 0.03 0.06 0 0.03 0.15 0 0.07 0.14 0 0.08 0.16 0.02 0.07  147 0.08 0 0.04 0.06 0 0.03 0.07 0 0.03 0.12 0 0.07 0.15 0 0.06 0.12 0 0.07  148 0.07 0 0.04 0.06 0 0.03 0.06 0 0.04 0.14 0 0.07 0.15 0 0.07 0.13 0 0.07  149 0.06 0 0.03 0.08 0 0.04 0.07 0 0.03 0.13 0 0.07 0.14 0 0.06 0.12 0 0.07  150 0.08 0 0.03 0.07 0 0.04 0.09 0 0.06 0.13 0 0.07 0.14 0 0.04 0.12 0.01 0.08  151 0.08 0 0.04 0.08 0 0.04 0.1 0 0.06 0.11 0 0.07 0.13 0 0.05 0.11 0 0.07  152 0.06 0 0.03 0.08 0 0.05 0.09 0 0.06 0.12 0 0.07 0.12 0 0.04 0.14 0 0.07  153 0.07 0 0.03 0.08 0 0.04 0.1 0 0.06 0.23 0.02 0.09 0.08 0 0.06 0.14 0 0.08  154 0.1 0 0.03 0.08 0 0.04 0.09 0 0.06 0.13 0 0.07 0.09 0 0.07 0.14 0 0.07  155 0.09 0 0.03 0.09 0 0.04 0.1 0 0.05 0.11 0 0.07 0.1 0 0.07 0.13 0 0.08  156 0.09 0 0.04 0.09 0 0.05 0.1 0 0.04 0.12 0 0.07 0.11 0 0.07 0.14 0.02 0.08  157 0.12 0 0.04 0.08 0 0.05 0.1 0.02 0.05 0.11 0 0.07 0.11 0 0.05 0.14 0 0.08  158 0.13 0 0.04 0.08 0 0.05 0.08 0.03 0.05 0.13 0 0.07 0.11 0 0.05 0.14 0.01 0.08  159 0.12 0 0.05 0.09 0 0.05 0.08 0 0.05 0.12 0 0.07 0.12 0 0.06 0.14 0 0.08  160 0.13 0 0.03 0.08 0 0.05 0.09 0.03 0.06 0.12 0 0.08 0.14 0 0.06 0.12 0.02 0.08  161 0.1 0 0.04 0.07 0 0.04 0.09 0.03 0.06 0.13 0 0.08 0.13 0 0.06 0.13 0.02 0.08  162 0.09 0 0.04 0.07 0 0.05 0.08 0 0.05 0.14 0 0.08 0.15 0 0.07 0.12 0 0.08  163 0.1 0 0.05 0.08 0 0.04 0.06 0 0.04 0.13 0 0.08 0.15 0 0.07 0.13 0 0.08  164 0.09 0 0.05 0.08 0 0.05 0.06 0.01 0.04 0.13 0 0.08 0.13 0 0.08 0.13 0 0.08  165 0.09 0 0.05 0.08 0 0.04 0.06 0 0.04 0.13 0.01 0.08 0.14 0 0.08 0.14 0 0.08  166 0.08 0 0.06 0.09 0 0.04 0.06 0.01 0.04 0.13 0 0.08 0.13 0 0.08 0.14 0.02 0.08  167 0.08 0 0.06 0.08 0 0.04 0.05 0 0.03 0.14 0 0.08 0.14 0 0.08 0.14 0.01 0.08  168 0.07 0 0.06 0.09 0 0.04 0.05 0 0.03 0.13 0 0.07 0.15 0 0.08 0.16 0 0.08  169 0.06 0 0.04 0.08 0.01 0.04 0.04 0 0.02 0.15 0 0.07 0.15 0 0.09 0.14 0 0.07  170 0.08 0 0.04 0.08 0.01 0.04 0.04 0 0.02 0.12 0 0.08 0.13 0 0.09 0.16 0 0.08  171 0.07 0 0.04 0.06 0 0.03 0.05 0 0.02 0.13 0 0.08 0.14 0 0.09 0.14 0 0.07  172 0.09 0 0.04 0.08 0 0.03 0.05 0 0.03 0.12 0 0.08 0.14 0 0.1 0.13 0 0.08  173 0.08 0 0.04 0.07 0 0.04 0.05 0 0.02 0.12 0 0.07 0.14 0 0.1 0.15 0 0.08  174 0.1 0 0.04 0.07 0 0.04 0.06 0 0.03 0.12 0 0.07 0.14 0.05 0.1 0.13 0 0.08  175 0.1 0 0.04 0.07 0 0.03 0.06 0 0.03 0.14 0 0.08 0.14 0.05 0.1 0.13 0.02 0.08  176 0.08 0.01 0.04 0.07 0 0.04 0.07 0 0.04 0.13 0 0.08 0.15 0.04 0.1 0.15 0 0.07  177 0.06 0 0.04 0.07 0 0.04 0.09 0 0.05 0.11 0 0.08 0.15 0.04 0.1 0.12 0 0.07  178 0.08 0 0.04 0.09 0 0.04 0.08 0 0.05 0.12 0 0.08 0.15 0.04 0.1 0.12 0 0.07  179 0.12 0 0.05 0.09 0 0.04 0.09 0 0.05 0.12 0 0.07 0.14 0.05 0.1 0.13 0 0.07  180 0.1 0 0.04 0.09 0 0.04 0.08 0 0.04 0.12 0 0.08 0.15 0.01 0.1 0.12 0.01 0.08  181 0.1 0 0.04 0.09 0 0.04 0.01 0 0.05 0.12 0 0.07 0.14 0.06 0.09 0.12 0 0.07  182 0.11 0.02 0.05 0.08 0 0.04 0.09 0 0.06 0.12 0 0.07 0.14 0 0.09 0.12 0 0.08  183 0.1 0.01 0.05 0.08 0 0.04 0.09 0 0.05 0.12 0.02 0.07 0.14 0 0.08 0.12 0 0.09  184 0.09 0.02 0.05 0.1 0 0.04 0.08 0 0.04 0.12 0 0.08 0.14 0 0.08 0.13 0 0.08  185 0.1 0.02 0.05 0.07 0 0.04 0.07 0 0.04 0.12 0 0.08 0.14 0 0.08 0.13 0.02 0.08  186 0.08 0.01 0.06 0.07 0 0.04 0.08 0 0.04 0.13 0 0.08 0.14 0 0.08 0.13 0 0.08  187 0.09 0.01 0.06 0.07 0 0.04 0.07 0 0.04 0.14 0 0.08 0.15 0.04 0.08 0.14 0 0.08  188 0.09 0 0.06 0.08 0 0.04 0.07 0 0.04 0.27 0 0.09 0.12 0.02 0.07 0.13 0 0.08  189 0.1 0.01 0.05 0.07 0 0.04 0.06 0 0.03 0.12 0 0.07 0.12 0.04 0.07 0.16 0 0.08  190 0.1 0.02 0.05 0.09 0.01 0.04 0.06 0 0.04 0.13 0 0.07 0.12 0 0.07 0.14 0 0.08  191 0.11 0 0.05 0.09 0.01 0.04 0.07 0 0.03 0.12 0.01 0.08 0.12 0 0.06 0.12 0 0.07  192 0.09 0 0.05 0.07 0 0.04 0.06 0 0.03 0.15 0 0.08 0.1 0 0.07 0.14 0.02 0.08  193 0.09 0 0.05 0.07 0.03 0.05 0.05 0 0.02 0.15 0.02 0.08 0.12 0 0.07 0.13 0 0.08  194 0.11 0.01 0.05 0.07 0 0.04 0.03 0 0.02 0.18 0 0.08 0.11 0 0.06 0.14 0 0.08  195 0.09 0 0.04 0.06 0 0.04 0.05 0 0.02 0.13 0 0.07 0.12 0 0.06 0.13 0 0.07  196 0.09 0 0.05 0.06 0 0.04 0.04 0 0.01 0.11 0 0.07 0.1 0.02 0.06 0.12 0.02 0.07  197 0.08 0 0.04 0.06 0 0.03 0.03 0 0.01 0.13 0 0.07 0.12 0 0.06 0.11 0 0.07  198 0.08 0 0.04 0.05 0 0.03 0.03 0 0.01 0.13 0 0.07 0.14 0 0.07 0.11 0 0.07  199 0.07 0 0.04 0.04 0 0.01 0.04 0 0.01 0.14 0 0.06 0.19 0 0.08 0.12 0 0.07  200 0.09 0 0.04 0.03 0 0.01 0.05 0 0.02 0.13 0 0.06 0.2 0 0.08 0.12 0 0.06 |
| --- |

1. **Automated gap thickness measurements for *AxC157* variety**

| Control (mm) Infected (mm)  Day 2 R1 R2 R3 R1 R2 R3  Max Min Average Max Min Average Max Min Average Max Min Average Max Min Average Max Min Average  1 0.16 0.01 0.06 0.13 0 0.07 0.07 0 0.05 0.06 0 0.03 0.11 0.03 0.07 0.05 0 0.06  2 0.17 0 0.06 0.13 0 0.07 0.07 0 0.06 0.04 0 0.02 0.12 0 0.07 0.05 0 0.06  3 0.14 0 0.07 0.13 0.02 0.07 0.07 0.01 0.08 0.05 0 0.03 0.12 0 0.07 0.06 0 0.06  4 0.14 0 0.05 0.1 0 0.07 0.07 0 0.06 0.04 0 0.03 0.05 0 0.02 0.06 0 0.06  5 0.13 0 0.07 0.12 0 0.07 0.08 0.02 0.05 0.03 0 0.06 0.1 0 0.06 0.04 0 0.06  6 0.12 0 0.06 0.06 0 0.03 0.09 0 0.04 0.09 0 0.06 0.08 0 0.05 0.05 0 0.05  7 0.15 0 0.05 0.04 0 0.02 0.09 0 0.05 0.07 0 0.06 0.11 0 0.06 0.04 0 0.03  8 0.13 0 0.05 0.05 0 0.03 0.09 0 0.05 0.08 0.02 0.08 0.1 0 0.1 0.03 0 0.01  9 0.13 0 0.05 0.04 0 0.03 0.11 0 0.05 0.06 0 0.03 0.1 0 0.08 0.09 0 0.06  10 0.12 0 0.05 0.03 0 0.1 0.09 0 0.04 0.05 0 0.05 0.06 0 0.02 0.07 0 0.05  11 0.14 0 0.05 0.09 0 0.06 0.07 0 0.03 0.03 0 0.06 0.07 0 0.03 0.08 0.02 0.04  12 0.3 0.02 0.05 0.07 0 0.04 0.09 0 0.06 0.09 0 0.05 0.05 0 0.05 0.06 0 0.03  13 0.3 0 0.04 0.08 0.02 0.08 0.01 0 0.06 0.1 0 0.05 0.11 0 0.06 0.05 0 0.05  14 0.11 0 0.06 0.06 0 0.03 0.08 0 0.06 0.09 0 0.05 0.05 0 0.03 0.03 0 0.06  15 0.09 0 0.04 0.05 0 0.02 0.07 0 0.06 0.11 0 0.06 0.06 0 0.03 0.03 0 0.06  16 0.1 0 0.05 0.11 0 0.07 0.09 0 0.03 0.12 0 0.06 0.03 0 0.02 0.09 0 0.05  17 0.19 0 0.05 0.13 0.02 0.07 0.08 0 0.03 0.13 0 0.07 0.06 0 0.03 0.13 0.02 0.07  18 0.11 0 0.06 0.11 0.03 0.06 0.06 0 0.06 0.13 0 0.07 0.05 0 0.06 0.11 0 0.07  19 0.12 0 0.05 0.09 0.01 0.07 0.06 0 0.07 0.12 0.02 0.07 0.07 0 0.06 0.11 0.03 0.06  20 0.13 0 0.06 0.07 0 0.08 0.06 0 0.06 0.1 0 0.07 0.06 0 0.06 0.09 0.01 0.07 |
| --- |

| Control (mm) Infected (mm)  Day 4 R1 R2 R3 R1 R2 R3  Max Min Average Max Min Average Max Min Average Max Min Average Max Min Average Max Min Average  1 0.1 0 0.05 0.8 0.01 0.05 0.1 0 0.06 0.12 0 0.08 0.1 0 0.06 0.1 0 0.08  2 0.09 0 0.05 0.08 0 0.06 0.12 0 0.05 0.13 0 0.08 0.1 0 0.05 0.11 0 0.07  3 0.1 0 0.04 0.09 0.01 0.06 0.1 0 0.06 0.12 0.04 0.08 0.1 0 0.05 0.11 0 0.06  4 0.1 0 0.04 0.1 0 0.05 0.1 0 0.06 0.13 0 0.08 0.09 0 0.09 0.1 0 0.07  5 0.09 0 0.04 0.11 0 0.06 0.11 0 0.06 0.17 0 0.08 0.09 0 0.08 0.1 0 0.06  6 0.12 0 0.05 0.11 0 0.05 0.1 0 0.06 0.12 0.02 0.07 0.1 0 0.05 0.11 0 0.06  7 0.11 0 0.05 0.1 0 0.05 0.1 0 0.06 0.12 0.01 0.07 0.1 0 0.06 0.11 0 0.06  8 0.1 0 0.05 0.09 0 0.05 0.12 0 0.07 0.12 0 0.07 0.13 0 0.08 0.09 0 0.05  9 0.1 0 0.06 0.11 0 0.06 0.13 0.03 0.07 0.13 0 0.07 0.13 0.01 0.08 0.09 0 0.09  10 0.1 0 0.06 0.11 0 0.06 0.12 0 0.07 0.12 0.02 0.08 0.13 0.01 0.06 0.1 0 0.06  11 0.11 0 0.05 0.12 0 0.06 0.12 0 0.06 0.14 0.03 0.07 0.14 0 0.08 0.09 0 0.05  12 0.1 0 0.05 0.11 0 0.05 0.13 0.03 0.07 0.17 0.02 0.08 0.15 0 0.07 0.1 0 0.07  13 0.1 0 0.06 0.11 0 0.06 0.13 0 0.07 0.16 0.02 0.07 0.16 0 0.06 0.09 0 0.07  14 0.11 0 0.06 0.12 0.01 0.06 0.12 0 0.06 0.12 0.01 0.07 0.14 0 0.07 0.09 0 0.08  15 0.1 0.01 0.06 0.11 0 0.07 0.12 0 0.06 0.12 0.01 0.07 0.13 0 0.07 0.1 0 0.1  16 0.09 0.02 0.06 0.11 0 0.07 0.12 0 0.06 0.11 0.01 0.06 0.15 0 0.07 0.11 0 0.05  17 0.1 0.01 0.06 0.13 0.03 0.08 0.12 0 0.06 0.1 0 0.06 0.15 0 0.08 0.09 0 0.06  18 0.1 0.01 0.06 0.11 0.03 0.07 0.12 0 0.07 0.11 0 0.05 0.15 0 0.08 0.1 0 0.06  19 0.11 0.02 0.06 0.12 0 0.08 0.12 0 0.07 0.12 0 0.05 0.15 0 0.09 0.09 0 0.05  20 0.1 0 0.05 0.12 0 0.09 0.13 0 0.07 0.12 0 0.05 0.17 0 0.08 0.1 0.01 0.06 |
| --- |

| Control (mm) Infected (mm)  Day 6 R1 R2 R3 R1 R2 R3  Max Min Average Max Min Average Max Min Average Max Min Average Max Min Average Max Min Average  1 0.05 0 0.06 0.11 0 0.07 0.08 0 0.06 0.05 0 0.03 0.04 0 0.1 0.15 0.01 0.09  2 0.06 0 0.06 0.11 0 0.06 0.08 0 0.06 0.08 0 0.04 0.07 0 0.06 0.19 0 0.11  3 0.04 0 0.06 0.17 0 0.07 0.05 0 0.03 0.06 0 0.04 0.12 0 0.07 0.23 0 0.13  4 0.06 0 0.03 0.05 0 0.04 0.05 0 0.06 0.09 0 0.04 0.05 0 0.07 0.11 0 0.06  5 0.06 0 0.03 0.07 0 0.04 0.06 0 0.07 0.08 0 0.03 0.12 0 0.06 0.12 0.03 0.08  6 0.06 0 0.03 0.07 0 0.04 0.07 0 0.07 0.06 0 0.03 0.08 0 0.04 0.23 0 0.12  7 0.07 0 0.05 0.09 0.01 0.05 0.08 0 0.07 0.06 0 0.04 0.11 0 0.04 0.13 0.04 0.09  8 0.08 0 0.05 0.08 0 0.04 0.05 0 0.05 0.06 0 0.03 0.11 0 0.06 0.1 0 0.06  9 0.08 0.02 0.05 0.07 0 0.04 0.05 0 0.06 0.05 0 0.03 0.11 0 0.06 0.14 0 0.07  10 0.07 0.02 0.04 0.08 0 0.05 0.06 0 0.03 0.05 0 0.03 0.12 0.01 0.07 0.08 0 0.06  11 0.08 0.02 0.05 0.09 0 0.05 0.06 0 0.03 0.06 0 0.04 0.11 0 0.08 0.09 0 0.05  12 0.09 0 0.05 0.01 0 0.06 0.07 0 0.07 0.12 0 0.04 0.12 0.04 0.08 0.11 0 0.05  13 0.08 0 0.05 0.11 0 0.07 0.04 0 0.06 0.04 0 0.02 0.11 0 0.06 0.13 0 0.04  14 0.07 0 0.05 0.12 0 0.06 0.06 0 0.1 0.05 0 0.02 0.14 0.01 0.08 0.07 0 0.04  15 0.06 0 0.05 0.15 0 0.07 0.04 0 0.1 0.07 0 0.02 0.12 0 0.05 0.08 0 0.04  16 0.08 0 0.05 0.09 0 0.05 0.05 0 0.06 0.1 0 0.05 0.11 0 0.06 0.06 0 0.05  17 0.09 0.03 0.06 0.08 0 0.05 0.03 0.01 0.08 0.02 0 0.6 0.15 0 0.06 0.07 0 0.06  18 0.08 0 0.06 0.1 0.02 0.05 0.04 0 0.05 0.08 0 0.04 0.05 0 0.03 0.26 0 0.05  19 0.07 0 0.07 0.08 0 0.05 0.06 0 0.06 0.09 0 0.04 0.11 0.03 0.08 0.08 0 0.06  20 0.07 0 0.07 0.08 0 0.04 0.06 0 0.07 0.12 0 0.05 0.108 0 0.1 0.08 0 0.03 |
| --- |


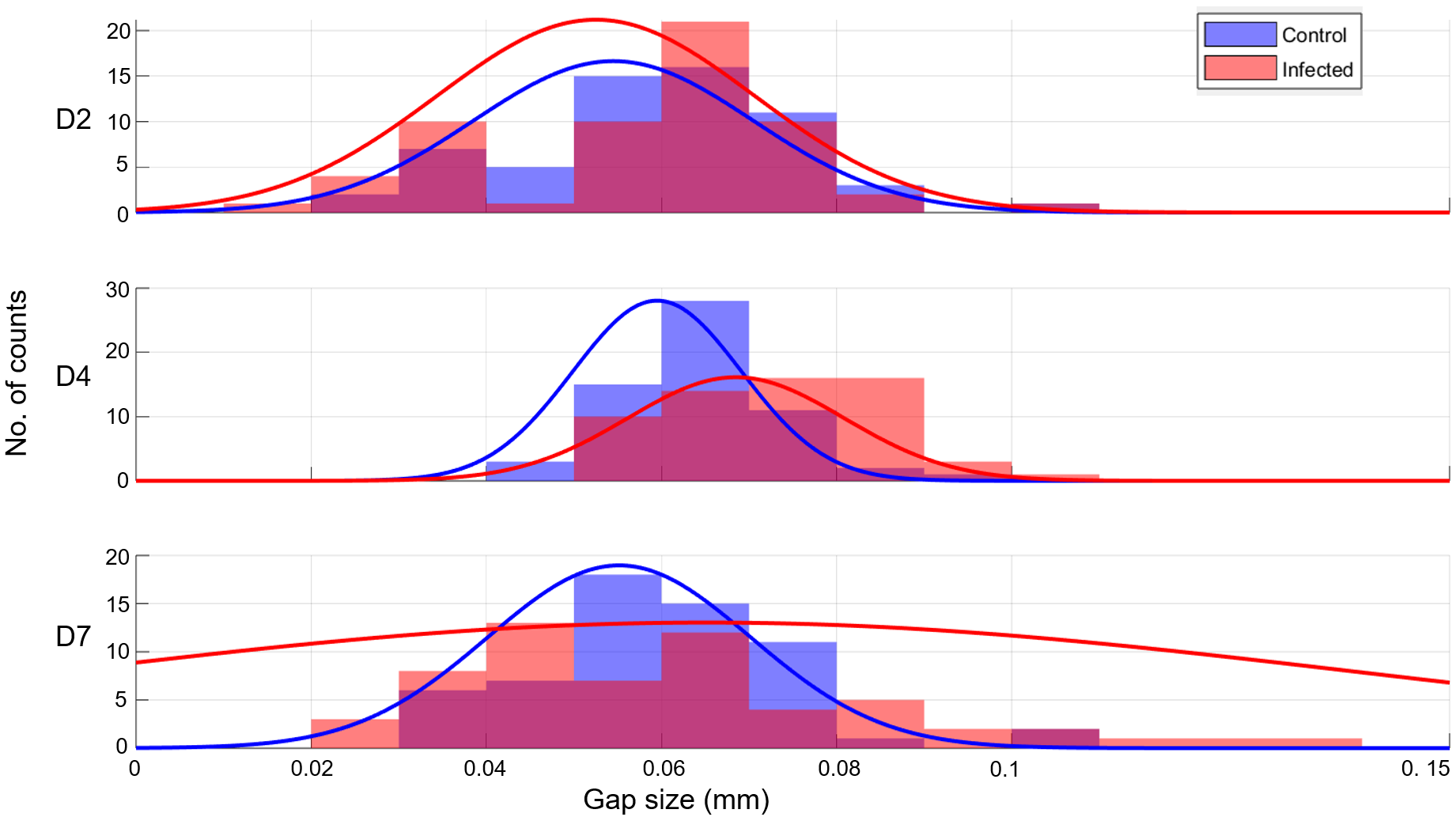


Figure 2: Average gap size distribution in control (blue) and infected (red) wheat leaves AxC157 variety extracted from automated segmentation, for day 2 (D2), day 4 (D4), and day 6 (D6) after inoculation, with superimposed Gaussian fits. The histograms use a bin width of 0.01 mm.

1. **Software rational**

This explains key variables and design decisions in the OCT leaf analyzer application, along with the exact lines of code where they are applied.

1. Image Size: 512 x 512
   - Reason: Standard input size for CNNs like UNet, balancing detail and speed.
   - Code:      self.image = cv2.resize(self.image, (512, 512), interpolation=cv2.INTER_AREA)

2. Segmentation Model: unet_masking3.keras
   - Reason: UNet is well-suited for precise segmentation of leaf regions.
   - Code:      masking_model = load_model('unet_masking3.keras')

3. Classification Model: classification_model.keras
   - Reason: For separating classification from segmentation, this classify the actual label infected/non-infected.
   - Code:      predict_using_thickness_data_model = load_model("classification_model.keras")

4. Classification Threshold: 0.5
   - Reason: Standard threshold for binary classification using sigmoid output.
   - Code:      tag = "Non-Infected" if results[0] > 0.5 else "Infected"

5. Features for Classification: min, max, avg
   - Reason: Capture relevant statistics of segmented thickness for diagnosis.
   - Code:      df = pd.DataFrame(thickness_data)
     details = {"tag": tag, "max": round(df['max'][0],2), "min": round(df['min'][0],2), "avg": round(df['avg'][0],2)}

6. Threading for Masking and Insights
   - Reason: Enables background processing without freezing the GUI.
   - Code:      self.mask_thread = MaskThread(...)
     self.insights_thread = InsightsThread(...)

1. **Python code**

import cv2

import os

import sys

import numpy as np

import pandas as pd

import tensorflow as tf

from PIL import Image

import logging

from PyQt5.QtCore import Qt, QSize, QThread, pyqtSignal

from PyQt5.QtGui import QPixmap, QImage

from PyQt5.QtWidgets import QApplication, QMainWindow, QLabel, QPushButton, QVBoxLayout, QHBoxLayout, QWidget, QFileDialog, QTextEdit, QProgressBar, QProgressDialog

# Configure logging

logging.basicConfig(filename='test.log', level=logging.INFO, format='%(asctime)s - %(levelname)s - %(message)s')

class ProgressDialog(QProgressDialog):

"""

Custom progress dialog that extends QProgressDialog for displaying operation progress.

"""

def __init__(self, parent=None):

super().__init__(parent)

self.setWindowModality(Qt.WindowModal)

self.setWindowTitle("Progress")

self.setMinimum(0)

self.setMaximum(100)

self.setValue(0)

def set_value(self, value):

self.setValue(value)

class MaskThread(QThread):

"""

Thread to handle image masking operations asynchronously to avoid UI freezing.

"""

mask_generated = pyqtSignal(np.ndarray, dict)

progress_updated = pyqtSignal(int)

def __init__(self, image_app_instance, image, masking_model, predict_using_thickness_data_model):

super().__init__()

self.image_app_instance = image_app_instance

self.image = image

self.masking_model = masking_model

self.predict_using_thickness_data_model = predict_using_thickness_data_model

self.is_running = True

def run(self):

mask = self.image_app_instance.generate_mask(self.image)

image_with_boundary = self.image_app_instance.create_boundary(mask, self.image)

filled_mask_area_image = self.image_app_instance.fill_mask(mask, self.image.copy())

straighten_image = self.image_app_instance.straight_images([image_with_boundary])

thickness_data = self.image_app_instance.calculate_thickness(straighten_image)

df = pd.DataFrame(thickness_data)

results = self.predict_using_thickness_data_model.predict(df)

tag = "Non-Infected" if results[0] > 0.5 else "Infected"

details = {"tag": tag, "max": round(df['max'][0],2), "min": round(df['min'][0],2), "avg": round(df['avg'][0],2)}

self.mask_generated.emit(np.array(filled_mask_area_image), details)

def stop(self):

self.is_running = False

class InsightsThread(QThread):

"""

Thread for generating insights across multiple images. Handles bulk processing.

"""

insights_generated = pyqtSignal(dict)

progress_updated = pyqtSignal(int)

def __init__(self, image_app_instance, file_paths, masking_model, predict_using_thickness_data_model):

super().__init__()

self.image_app_instance = image_app_instance

self.file_paths = file_paths

self.masking_model = masking_model

self.predict_using_thickness_data_model = predict_using_thickness_data_model

self.is_running = True

def run(self):

straighten_image = []

total_files = len(self.file_paths)

for idx, file_path in enumerate(self.file_paths):

if not self.is_running:

break

img_for_calculation = cv2.imread(file_path, cv2.IMREAD_COLOR)

if img_for_calculation is not None:

mask = self.image_app_instance.generate_mask(img_for_calculation.copy())

image_with_boundary = self.image_app_instance.create_boundary(mask, img_for_calculation)

straighten_image.append(self.image_app_instance.straight_images([image_with_boundary])[0])

self.progress_updated.emit(int((idx + 1) * 90 / total_files))

if straighten_image:

thickness_data = self.image_app_instance.calculate_thickness(straighten_image)

self.progress_updated.emit(95)

df = pd.DataFrame(thickness_data)

results = self.predict_using_thickness_data_model.predict(df)

mean_df = df.mean(axis=0)

mean_df_for_pred = pd.DataFrame([{'min': mean_df['min'], 'max': mean_df['max'], 'avg': mean_df['avg']}])

mean_result = self.predict_using_thickness_data_model.predict(mean_df_for_pred)

tag = "Non-Infected" if mean_result[0] > 0.5 else "Infected"

details = {

"tag": tag, "max": round(mean_df_for_pred['max'][0], 2),

"min": round(mean_df_for_pred['min'][0], 2),

"avg": round(mean_df_for_pred['avg'][0], 2),

"total images": len(results),

"detected non-infected images": list(results).count(1),

"detected infected images": list(results).count(0)

}

self.insights_generated.emit(details)

self.progress_updated.emit(98)

self.progress_updated.emit(100)

self.finished.emit()

def stop(self):

self.is_running = False

class Label(QLabel):

"""

Custom QLabel with drag-and-drop support for images or image folders.

"""

imageLoaded = pyqtSignal()

def __init__(self, title, parent):

super().__init__(title, parent)

self.setAcceptDrops(True)

self.file_paths = []

def dragEnterEvent(self, e):

if e.mimeData().hasUrls():

e.accept()

else:

e.ignore()

def dropEvent(self, e):

m = e.mimeData()

if m.hasUrls():

for url in m.urls():

if url.isLocalFile():

file_path = url.toLocalFile()

if os.path.isfile(file_path):

self.file_paths = []

self.window().load_image(file_path)

self.window().image_count_label.setText(f"Image 1 of 1")

elif os.path.isdir(file_path):

self.current_index = -1

image_files = [f for f in os.listdir(file_path) if f.endswith(('.png', '.jpg', '.jpeg', '.bmp', '.tif', '.tiff'))]

self.file_paths = [os.path.join(file_path, f) for f in sorted(image_files)]

self.load_next_image()

def load_next_image(self):

if self.file_paths and self.current_index < len(self.file_paths) - 1:

self.current_index += 1

self.window().load_image(self.file_paths[self.current_index])

self.imageLoaded.emit()

def load_previous_image(self):

if self.file_paths and self.current_index > 0:

self.current_index -= 1

self.window().load_image(self.file_paths[self.current_index])

self.imageLoaded.emit()

class ImageApp(QMainWindow):

"""

Main application window for the OCT Leaf Analyzer.

"""

def __init__(self, masking_model, predict_using_thickness_data_model):

super().__init__()

self.masking_model = masking_model

self.predict_using_thickness_data_model = predict_using_thickness_data_model

self.setup_ui()

def setup_ui(self):

self.setWindowTitle("OCT Leaf Analyzer")

self.setGeometry(100, 100, 1000, 600)

central_widget = QWidget(self)

self.setCentralWidget(central_widget)

main_layout = QVBoxLayout(central_widget)

image_container = QWidget()

image_layout = QHBoxLayout(image_container)

self.original_label = Label("Drag and Drop image or folder here", self)

self.original_label.setAlignment(Qt.AlignCenter)

image_layout.addWidget(self.original_label)

self.masked_label = QLabel("", self)

self.masked_label.setAlignment(Qt.AlignCenter)

image_layout.addWidget(self.masked_label)

main_layout.addWidget(image_container)

button_layout = QHBoxLayout()

self.load_button = QPushButton("Load OCT Image", self)

self.load_button.clicked.connect(self.load_image)

button_layout.addWidget(self.load_button)

self.mask_button = QPushButton("Apply Masking", self)

self.mask_button.clicked.connect(self.apply_mask_async)

button_layout.addWidget(self.mask_button)

self.generate_insights_button = QPushButton("Generate Insights for All", self)

self.generate_insights_button.clicked.connect(self.generate_insights_for_all)

button_layout.addWidget(self.generate_insights_button)

self.details_textedit = QTextEdit(self)

self.details_textedit.setReadOnly(True)

main_layout.addLayout(button_layout)

main_layout.addWidget(self.details_textedit)

arrow_layout = QHBoxLayout()

self.prev_button = QPushButton("Previous", self)

self.prev_button.clicked.connect(self.original_label.load_previous_image)

arrow_layout.addWidget(self.prev_button)

self.image_count_label = QLabel("", self)

self.image_count_label.setAlignment(Qt.AlignCenter)

arrow_layout.addWidget(self.image_count_label)

self.next_button = QPushButton("Next", self)

self.next_button.clicked.connect(self.original_label.load_next_image)

arrow_layout.addWidget(self.next_button)

main_layout.addLayout(arrow_layout)

self.setStyleSheet("""

QMainWindow {

background-color: #37474F;

}

QPushButton {

background-color: #FFC107;

color: #37474F;

border-radius: 15px;

padding: 10px;

margin: 6px;

font-size: 14px;

}

QPushButton:hover {

background-color: #FFD54F;

}

QLabel {

color: #FFFFFF;

background-color: #455A64;

padding: 5px;

border-radius: 5px;

font-size: 16px;

font-weight: semi-bold;

}

QTextEdit, QLineEdit {

background-color: #CFD8DC;

color: #37474F;

font-size: 14px;

border: none;

padding: 5px;

border-radius: 5px;

}

""")

self.original_label.imageLoaded.connect(self.update_details)

def load_image(self, file_path=None):

"""

Load an image from the file dialog or from a dropped file.

"""

if not file_path:

options = QFileDialog.Options()

file_path, _ = QFileDialog.getOpenFileName(self, "Select OCT Image", "", "Image Files (*.png *.jpg *.jpeg *.bmp *.tif *.tiff)", options=options)

if file_path:

self.image = cv2.imread(file_path, cv2.IMREAD_COLOR)

self.image = cv2.resize(self.image, (512, 512), interpolation=cv2.INTER_AREA)

if self.image is not None:

self.display_image(self.image, self.original_label)

self.update_details(file_path)

self.masked_label.clear()

def apply_mask_async(self):

"""

Apply mask to the loaded image asynchronously.

"""

if self.image is not None:

self.progress_dialog = ProgressDialog(self)

self.progress_dialog.show()

self.progress_dialog.setLabelText("Applying Masking")

self.mask_thread = MaskThread(self, self.image.copy(), self.masking_model, self.predict_using_thickness_data_model)[Book](https://sciasuedu-my.sharepoint.com/:x:/g/personal/ghadasasi_sci_asu_edu_eg/EfE6pbfLYOJNldPx4_uj108BpFCKZKolHgKPPz-DNctWVQ)[Gap thickness. AxC169. Manual.xls](file:///C:\Users\Adrien\Desktop\Adrien\aapc_Sheffield%20Uni\Students\2021_2025_PhD_Ghada%20Sasi\Gap%20thickness.%20AxC169.%20Manual.xls)

self.mask_thread.mask_generated.connect(self.show_mask_result)

self.mask_thread.progress_updated.connect(self.update_progress_bar)

self.mask_thread.finished.connect(self.progress_dialog.accept)

self.progress_dialog.canceled.connect(self.stop_mask_thread)

self.mask_thread.start()

def stop_mask_thread(self):

"""

Stop the mask thread if it is still running.

"""

if self.mask_thread.isRunning():

self.mask_thread.stop()

def generate_insights_for_all(self):

"""

Generate insights for all images loaded into the application.

"""

if not self.original_label.file_paths:

return # No files loaded, exit the function

self.progress_dialog = ProgressDialog(self)

self.progress_dialog.show()

self.progress_dialog.setLabelText("Generating Insights")

self.insights_thread = InsightsThread(self, self.original_label.file_paths, self.masking_model, self.predict_using_thickness_data_model)

self.insights_thread.insights_generated.connect(self.show_insights)

self.insights_thread.progress_updated.connect(self.update_progress_bar)

self.insights_thread.finished.connect(self.progress_dialog.accept)

self.progress_dialog.canceled.connect(self.stop_insights_thread)

self.insights_thread.start()

def stop_insights_thread(self):

"""

Stop the insights generation thread if it is still running.

"""

if self.insights_thread.isRunning():

self.insights_thread.stop()

def update_progress_bar(self, progress_percent):

"""

Update the progress bar in the UI.

"""

self.progress_dialog.set_value(progress_percent)

def show_mask_result(self, masked_image, details):

"""

Display the masked image and details in the UI.

"""

self.display_image(self.image, self.original_label)

self.display_image(masked_image, self.masked_label)

self.show_details(details)

def show_insights(self, detail):

"""

Display the insights for all processed images in the UI.

"""

details = (f"<b>Total images</b>: {detail['total images']}<br>"

f"<b>Detected Non-Infected images</b>: {detail['detected non-infected images']}<br>"

f"<b>Detected Infected images</b>: {detail['detected infected images']}<br><br>"

f"<b>Mean insights for all images</b> :-<br><br>"

f"<b>Tag</b>: {detail['tag']}<br>"

f"<b>Maximum Thickness</b>: {detail['max']}mm<br>"

f"<b>Minimum Thickness</b>: {detail['min']}mm<br>"

f"<b>Average Thickness</b>: {detail['avg']}mm<br><br>"

f"<b>Note</b>: 100px = 1mm")

self.details_textedit.setHtml(details)

def display_image(self, image, label):

"""

Utility function to display an image on a QLabel.

"""

if image is not None:

if len(image.shape) == 3:

height, width, channel = image.shape

else:

height, width = image.shape

image = cv2.cvtColor(image, cv2.COLOR_BGR2RGB)

bytesPerLine = 3 * width

qImg = QImage(image.data, width, height, bytesPerLine, QImage.Format_RGB888).rgbSwapped()

pixmap = QPixmap.fromImage(qImg)

pixmap = pixmap.scaled(QSize(512, 512), Qt.KeepAspectRatio)

label.setPixmap(pixmap)

def load_model(path):

"""

Load a TensorFlow model from a specified path.

"""

try:

if getattr(sys, 'frozen', False):

# When running as a PyInstaller bundle

base_path = sys._MEIPASS

else:

# When running as a script

base_path = os.path.dirname(os.path.abspath(__file__))

model_path = os.path.join(base_path, path)

model = tf.keras.models.load_model(model_path)

logging.info('Model loaded successfully from main')

return model

except FileNotFoundError:

logging.error('Model file not found. Please verify the file path.')

except Exception as e:

logging.error(f'Failed to load model: {e}')

if __name__ == "__main__":

tf.compat.v1.enable_eager_execution()

predict_using_thickness_data_model = load_model("classification_model.keras")

masking_model = load_model('unet_masking3.keras')

app = QApplication(sys.argv)

window = ImageApp(masking_model, predict_using_thickness_data_model)

window.show()

sys.exit(app.exec_())
